# Supplementary material for: From Sea to Table: The Role of Traceability in Italian Seafood Consumption
Source: Foods. 2025 Oct 11;14(20):3469. doi: 10.3390/foods14203469 (PMC12563374; doi:10.3390/foods14203469)
Supplement: Supplementary file 1 [file foods-14-03469-s001.zip › foods-3766972-supplementary.pdf]

Supplementary material

Contingency Table Analyses and Analyses of Variance (ANOVAs) for the 2022 dataset

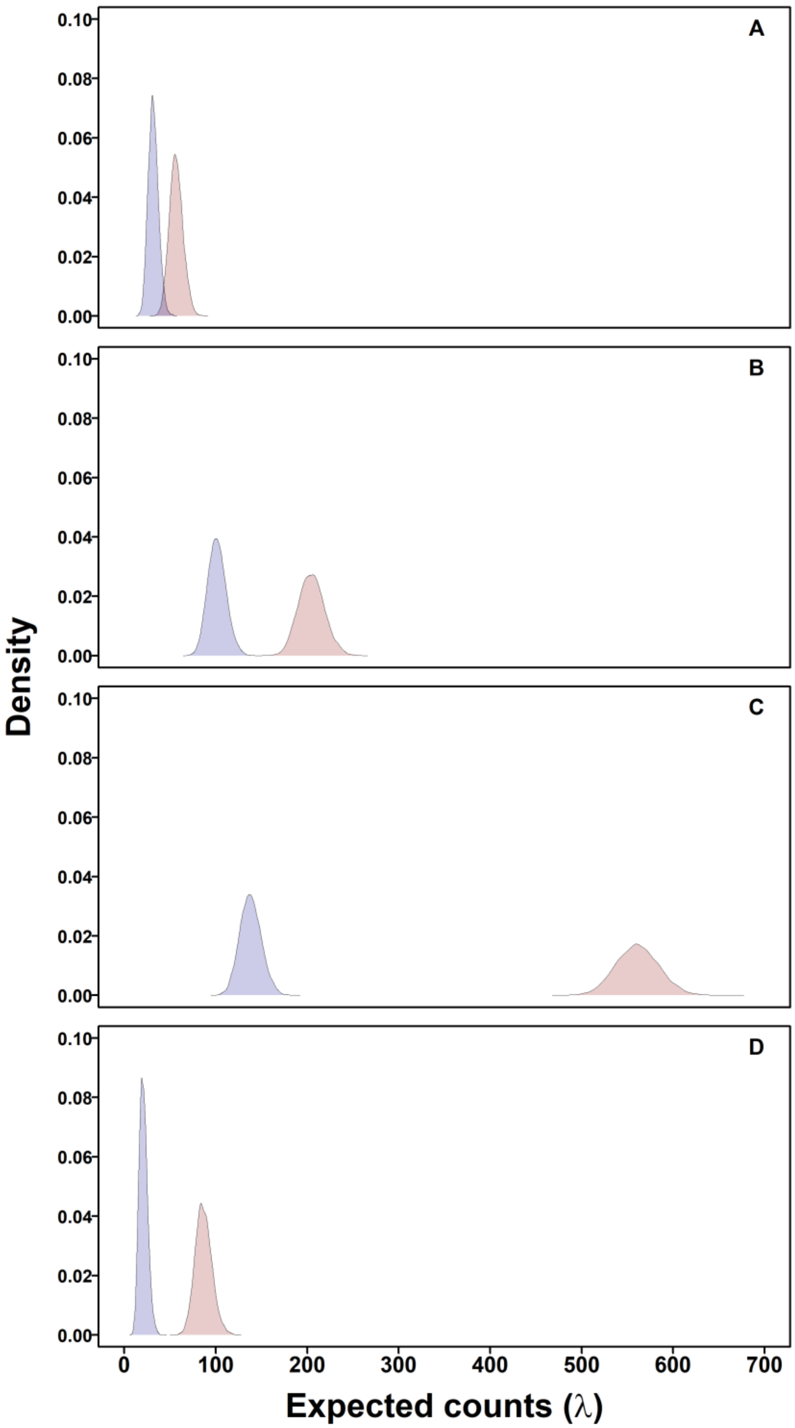

Supplementary Figure S1. Posterior densities of the expected counts (λ) for being informed on seafood origin (Red= "Yes" and Blue= "No") per age range (from A to D: 18–25, 26–40, 41–65, and 66+), extracted after 10^4 MCMC draws.

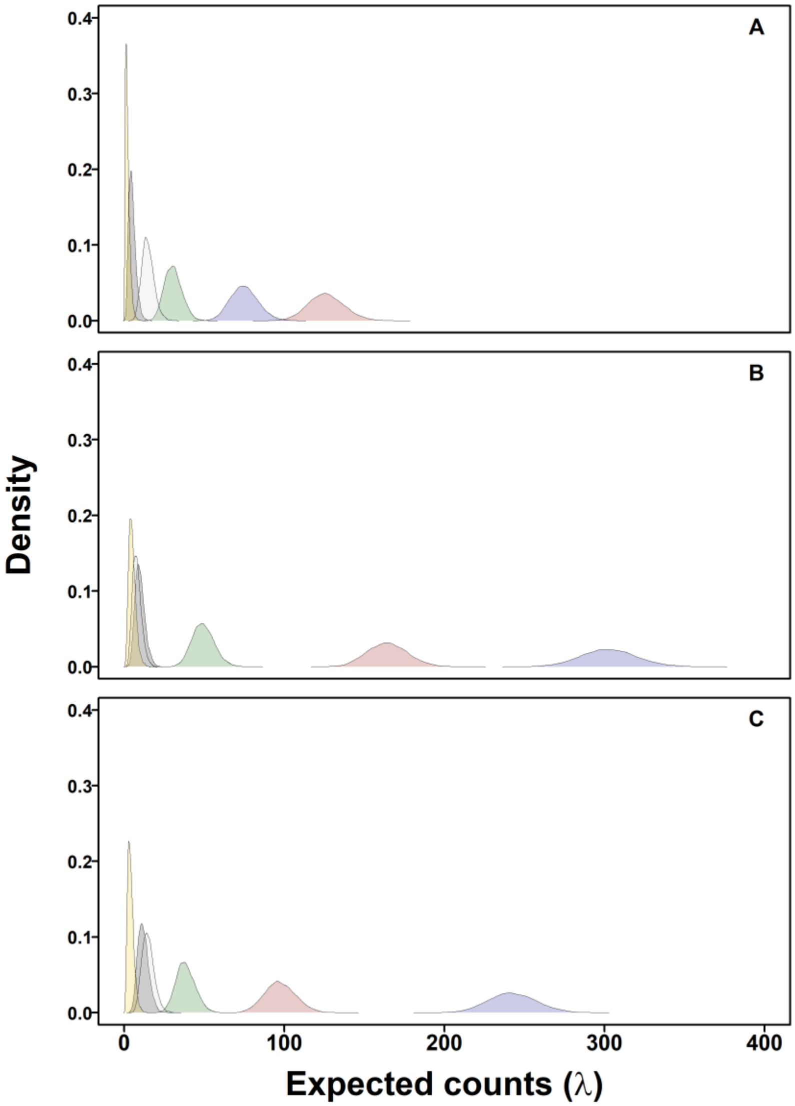

Supplementary Figure S2. Posterior densities of the expected counts (λ) for where consumers buy seafood (Red= "Fish market", Blue= "Large retail", Green= "Local market", Dark gray= "Not buy", Yellow= "Online", and Light gray = "Other") per province (from A to C: "South", "Central", and "North"), extracted after 10^4 MCMC draws.

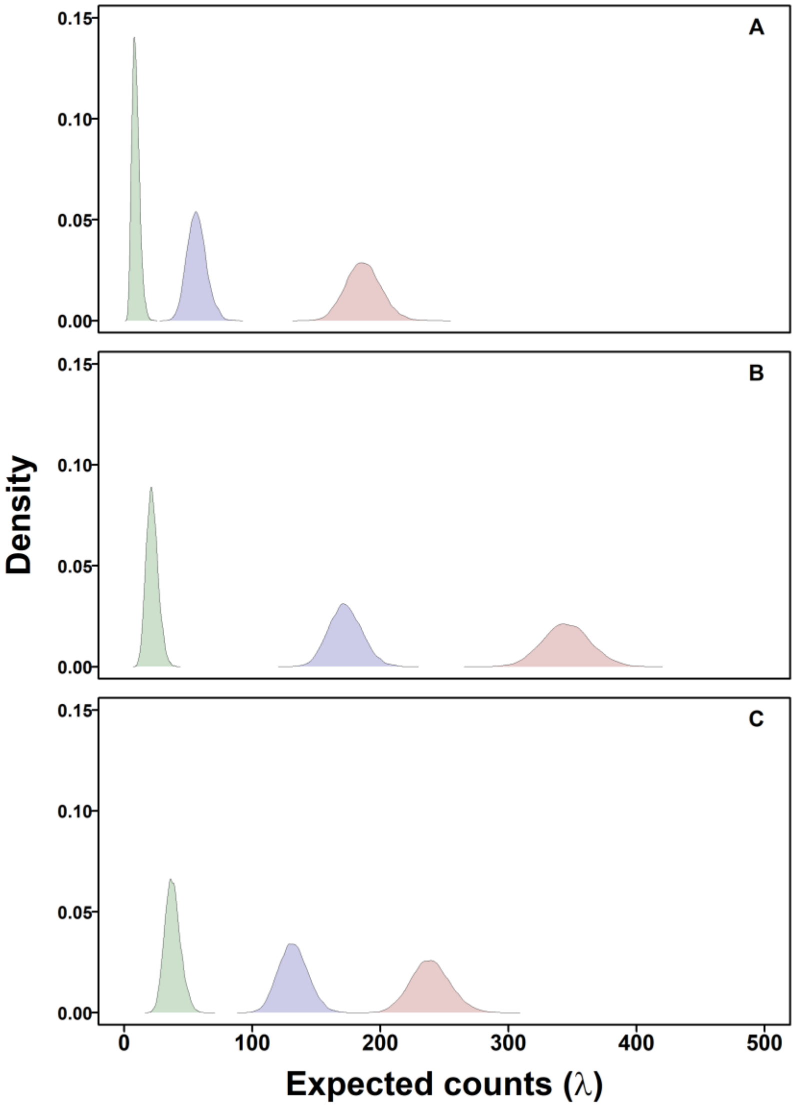

Supplementary Figure S3. Posterior densities of the expected counts (λ) for the degree of seafood processing consumers prefer (Red= "Fresh", Blue= "Frozen", and Green= "Processed") per province (from A to C: "South", "Central", and "North"), extracted after 10^4 MCMC draws.

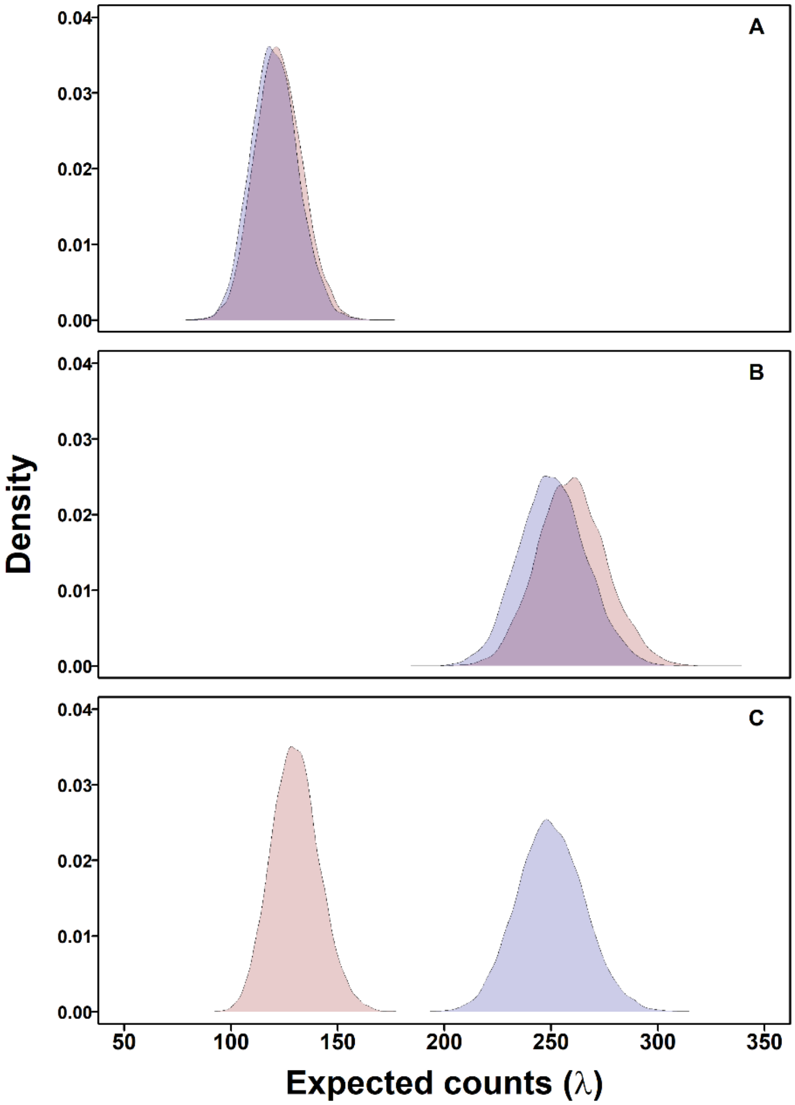

Supplementary Figure S4. Posterior densities of the expected counts (λ) for the consumption of Italian-farmed sea bass (Red= "Yes", and Blue= "No") per province (from A to C: "South", "Central", and "North"), extracted after 10^4 MCMC draws.

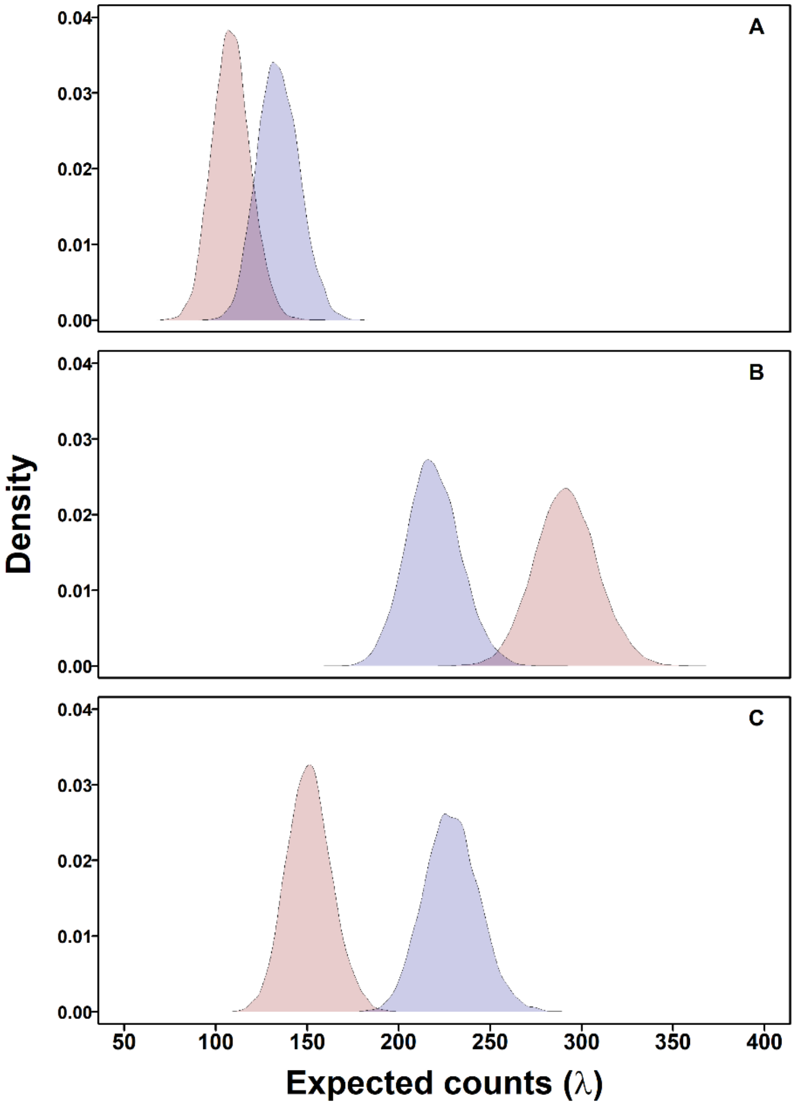

Supplementary Figure S5. Posterior densities of the expected counts (λ) for the consumption of striped venus clams (Red= "Yes", and Blue= "No") per province (from A to C: "South", "Central", and "North"), extracted after 10^4 MCMC draws.

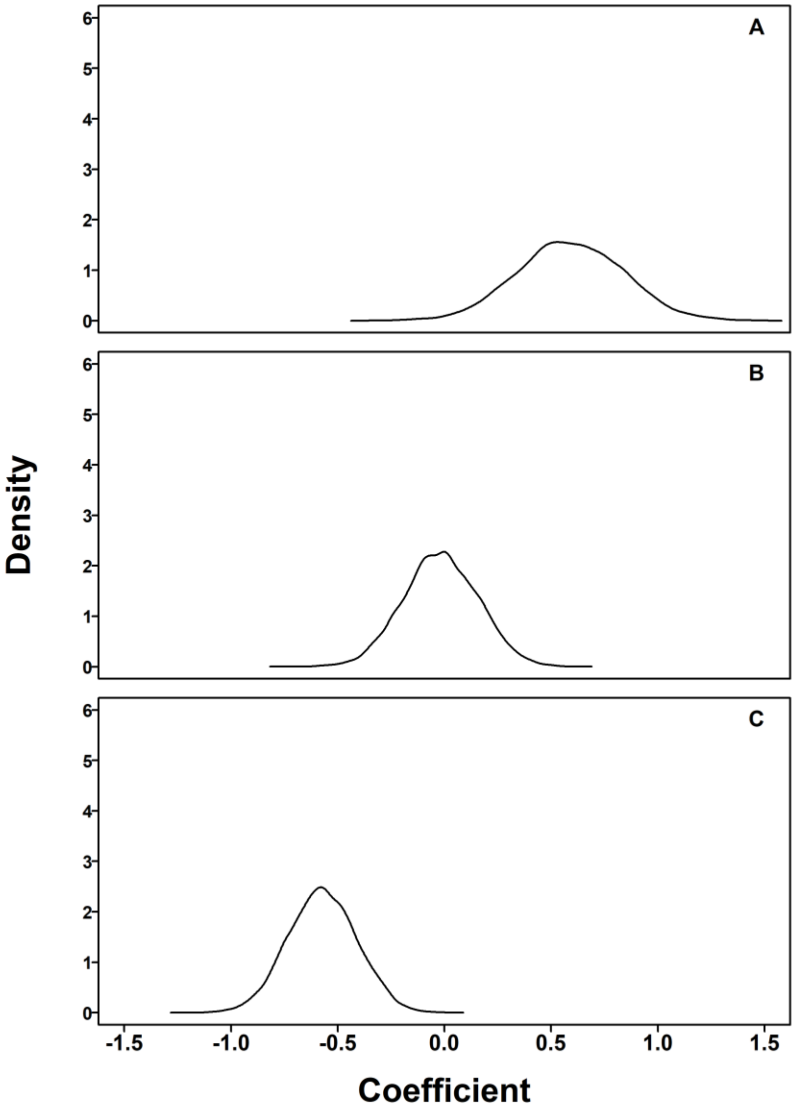

Supplementary Figure S6. Posterior densities of the ANOVA coefficients for the consumers' WTP for striped venus clams per educational level (from A to C: "Middle school degree", "High school degree", and "BSc degree or higher"), extracted after 10^4 MCMC draws.

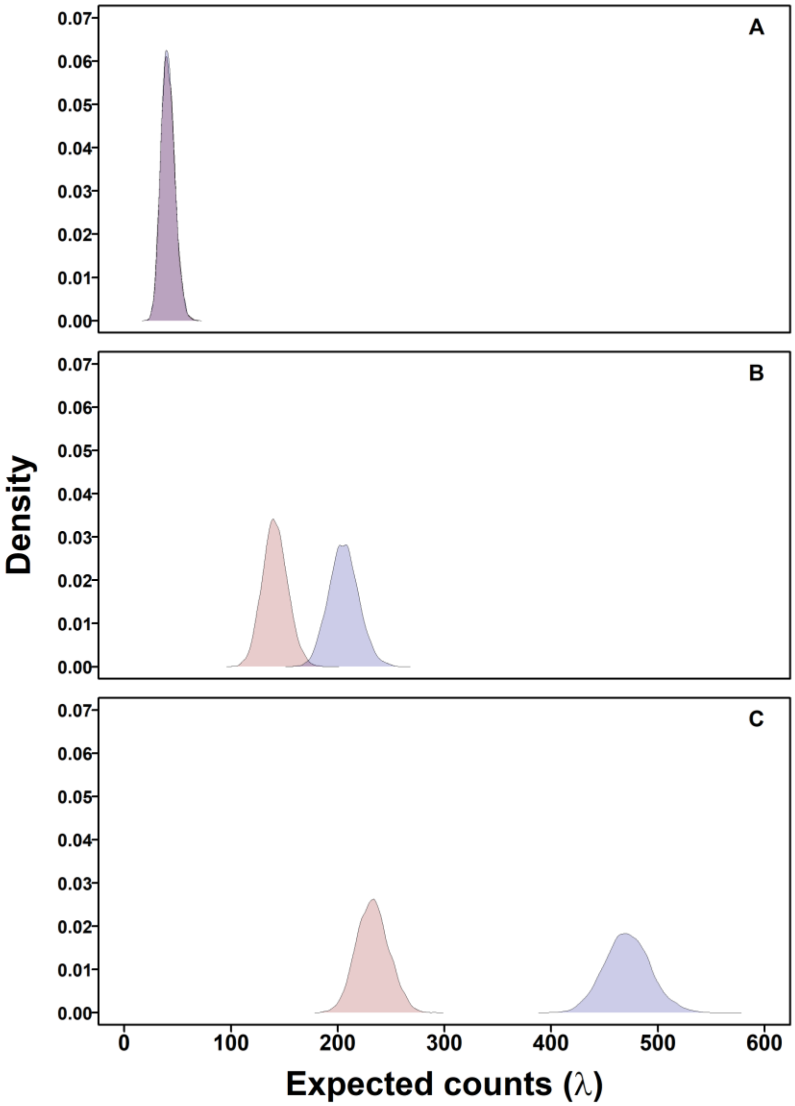

Supplementary Figure S7. Posterior densities of the expected counts (λ) for the consumption of giant red shrimp (Red= “Yes”, and Blue= “No”) per educational level (from A to C: “Middle school degree”, “High school degree”, and “BSc degree or higher”), extracted after 10^4 MCMC draws.

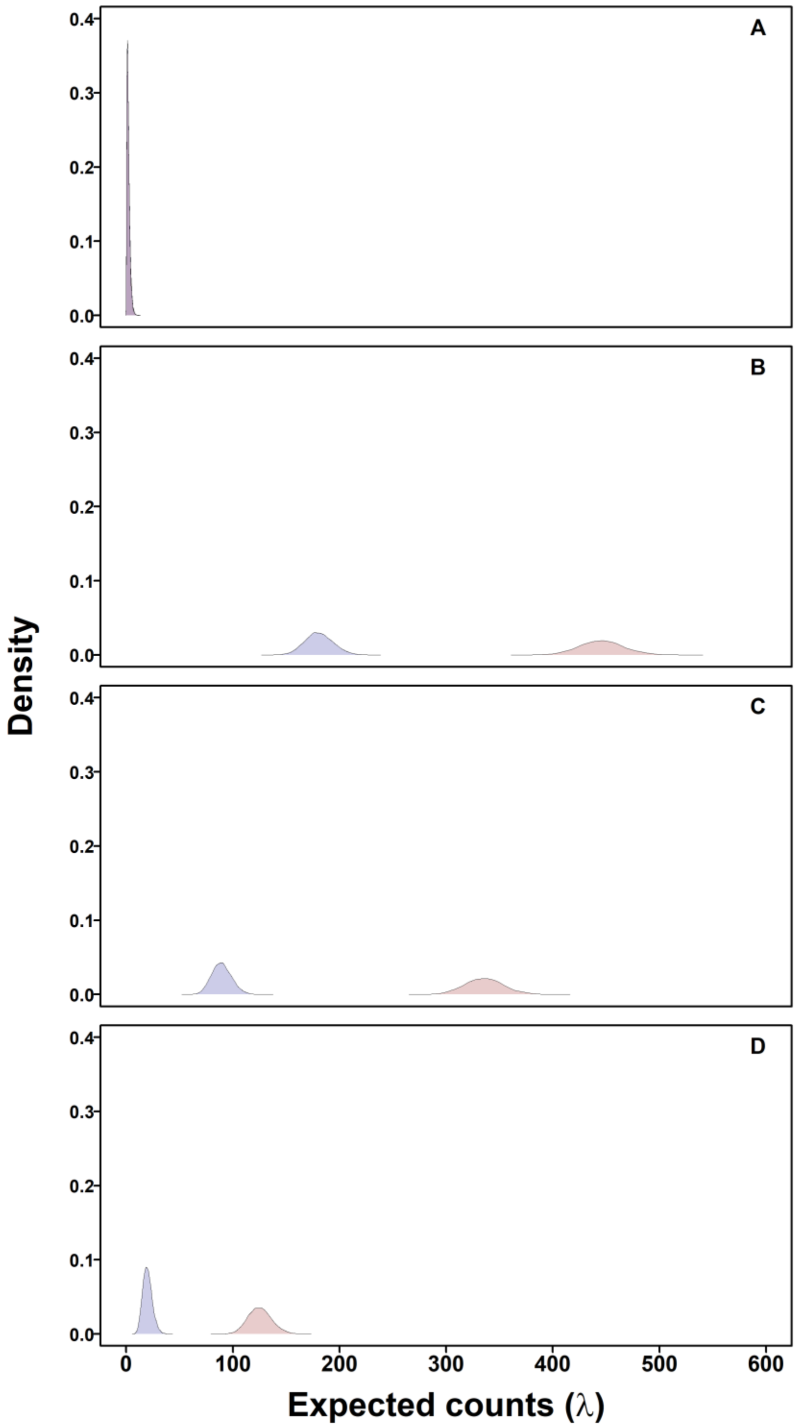

Supplementary Figure S8. Posterior densities of the expected counts (λ) for being informed on seafood origin (Red= "Yes", and Blue= "No") per monthly seafood consumption rate (from A to D: 0, 1–5, 6–10, and 10+), extracted after 10^4 MCMC draws.

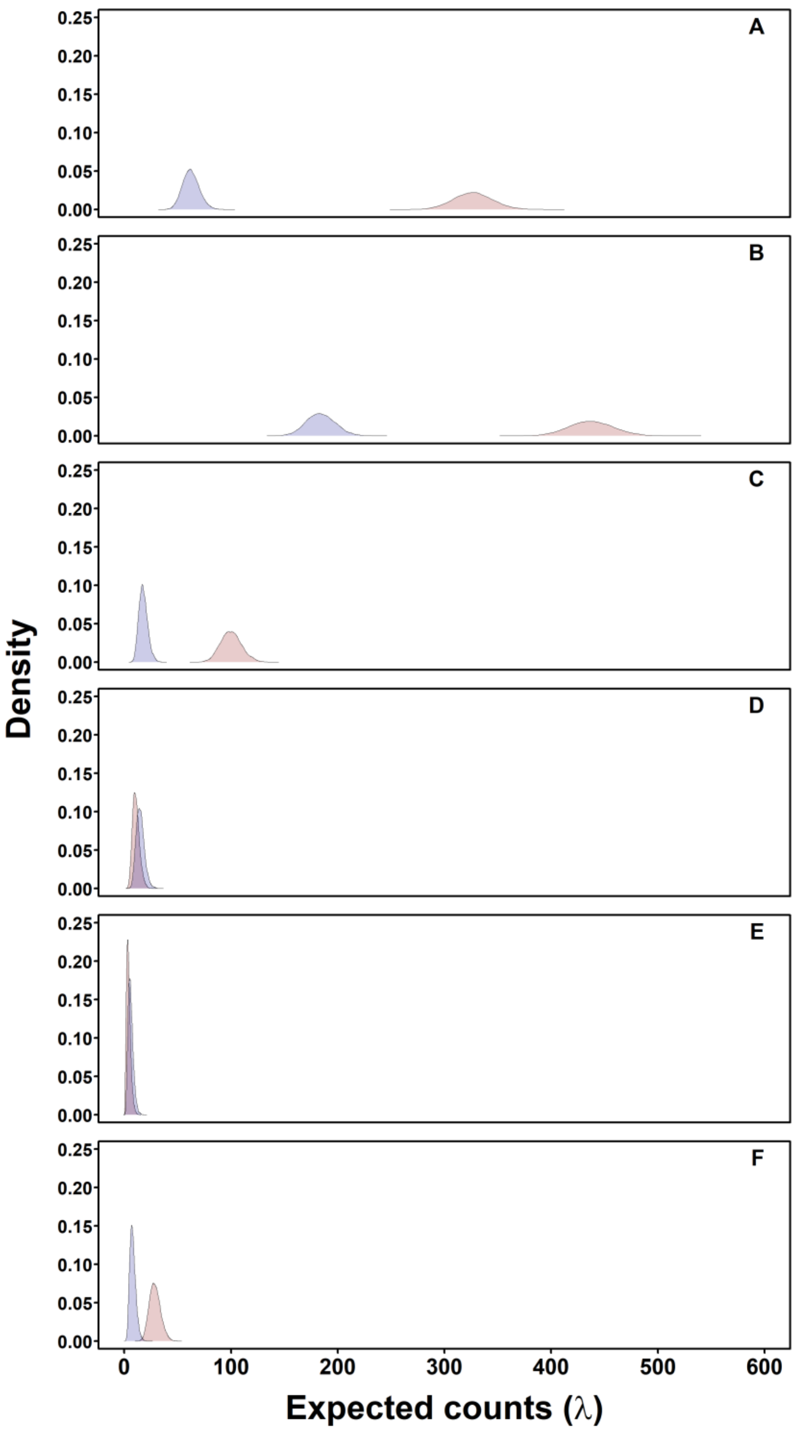

Supplementary Figure S9. Posterior densities of the expected counts (λ) for being informed on seafood origin (Red= "Yes", and Blue= "No") per where consumers buy seafood (from A to F: "Fish market", "Large retail", "Local market", "Not buy", "Online", and "Other"), extracted after 10^4 MCMC draws.

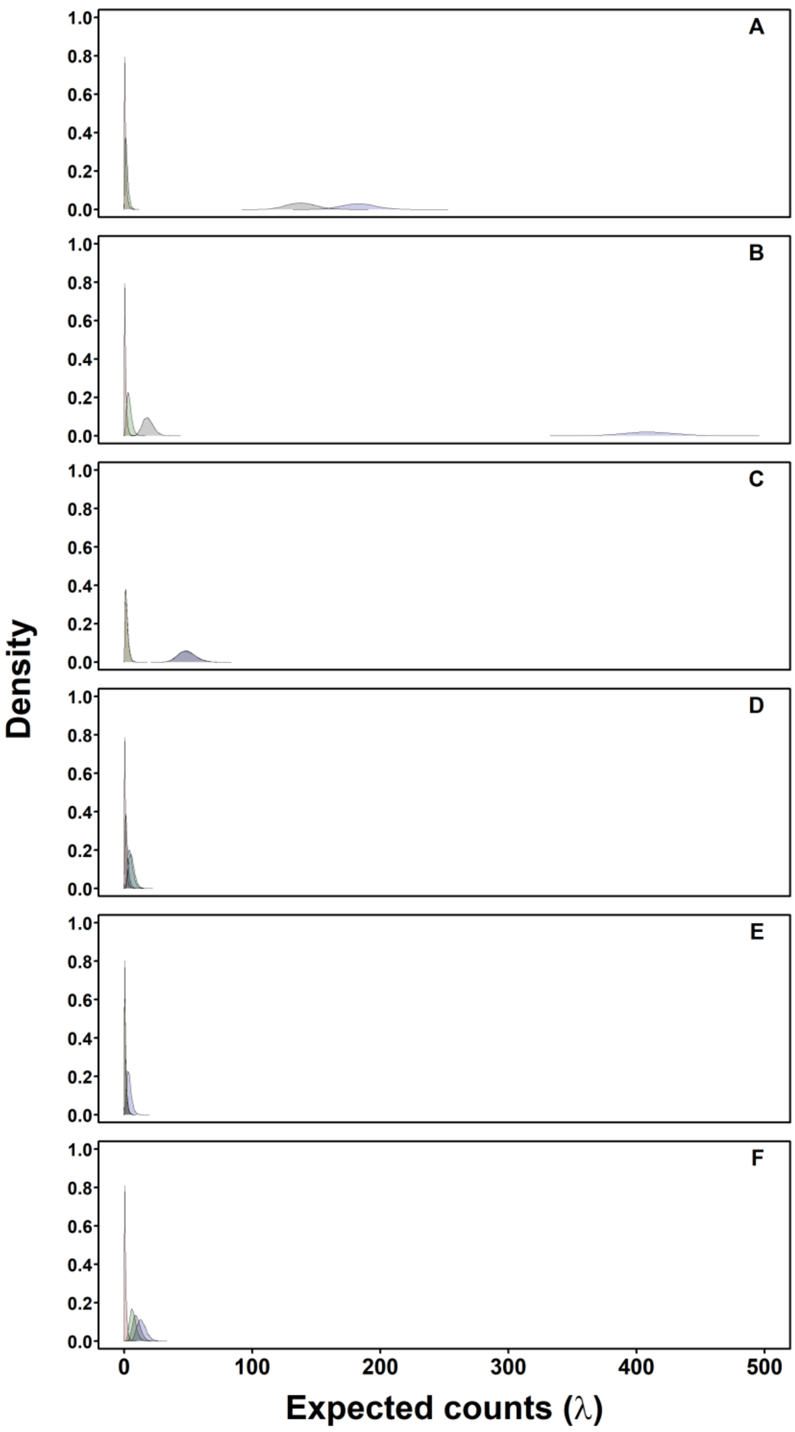

Supplementary Figure S10. Posterior densities of the expected counts (λ) for sources of information on seafood origin (Red= "Ads", Blue= "Label", Green= "Other", and Dark gray= "Retailer") per where consumers buy seafood (from A to F: "Fish market", "Large retail", "Local market", "Not buy", "Online", and "Other"), extracted after 10^4 MCMC draws.

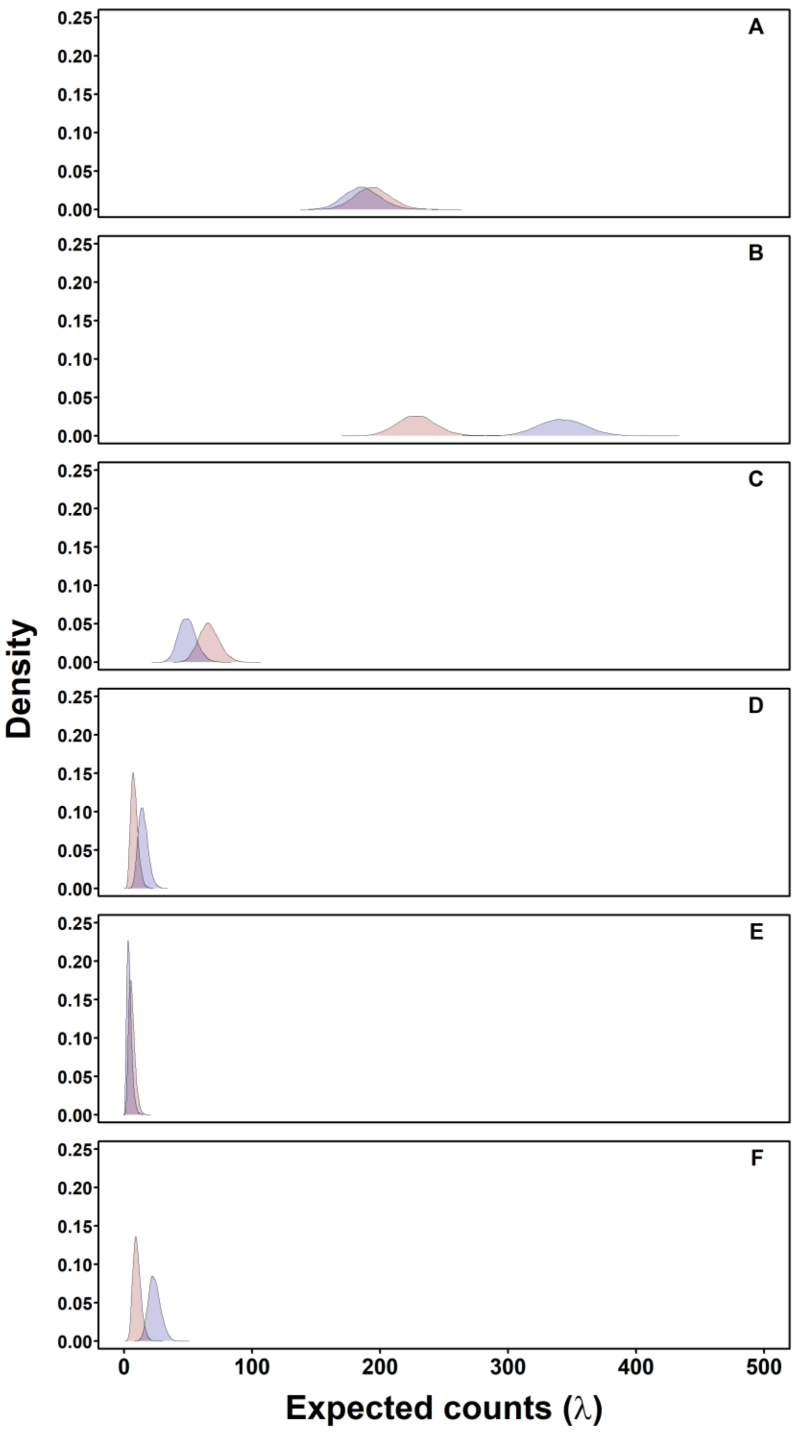

Supplementary Figure S11. Posterior densities of the expected counts (λ) for the consumption of Italian-farmed sea bass (Red= "Yes", and Blue= "No") per where consumers buy seafood (from A to F: "Fish market", "Large retail", "Local market", "Not buy", "Online", and "Other"), extracted after 10^4 MCMC draws.

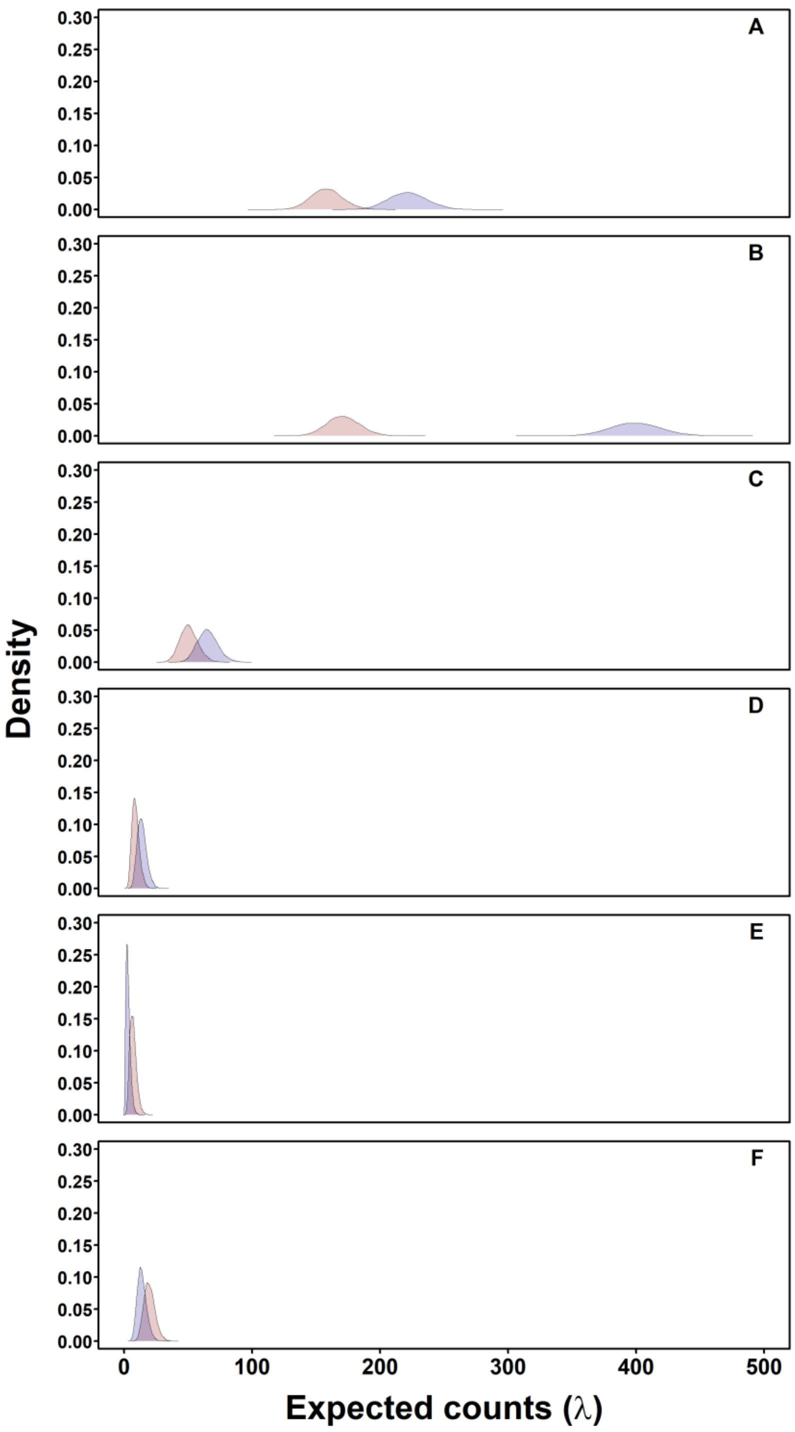

Supplementary Figure S12. Posterior densities of the expected counts (λ) for the consumption of giant red shrimp (Red= “Yes”, and Blue= “No”) per where consumers buy seafood (from A to F: “Fish market”, “Large retail”, “Local market”, “Not buy”, “Online”, and “Other”), extracted after 10^4 MCMC draws.

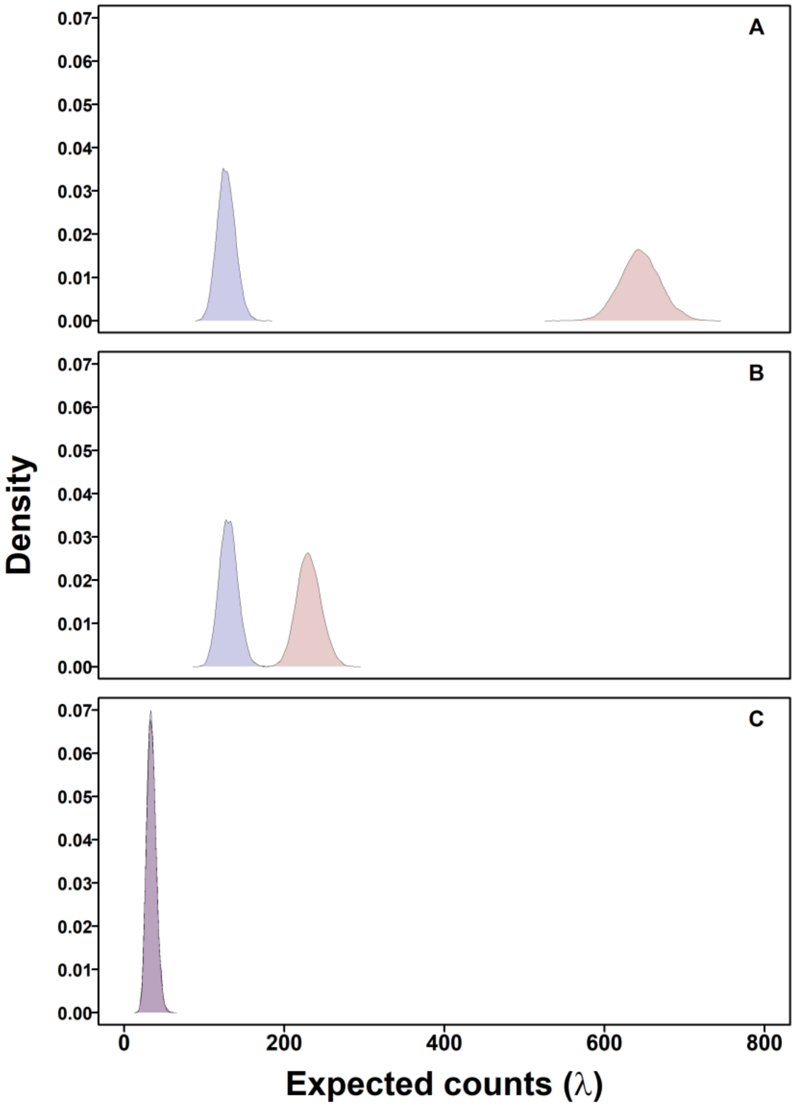

Supplementary Figure S13. Posterior densities of the expected counts (λ) for being informed on seafood origin (Red= "Yes", and Blue= "No") per degree of seafood processing consumers prefer (from A to C: "Fresh" "Frozen", and "Processed"), extracted after 10^4 MCMC draws.

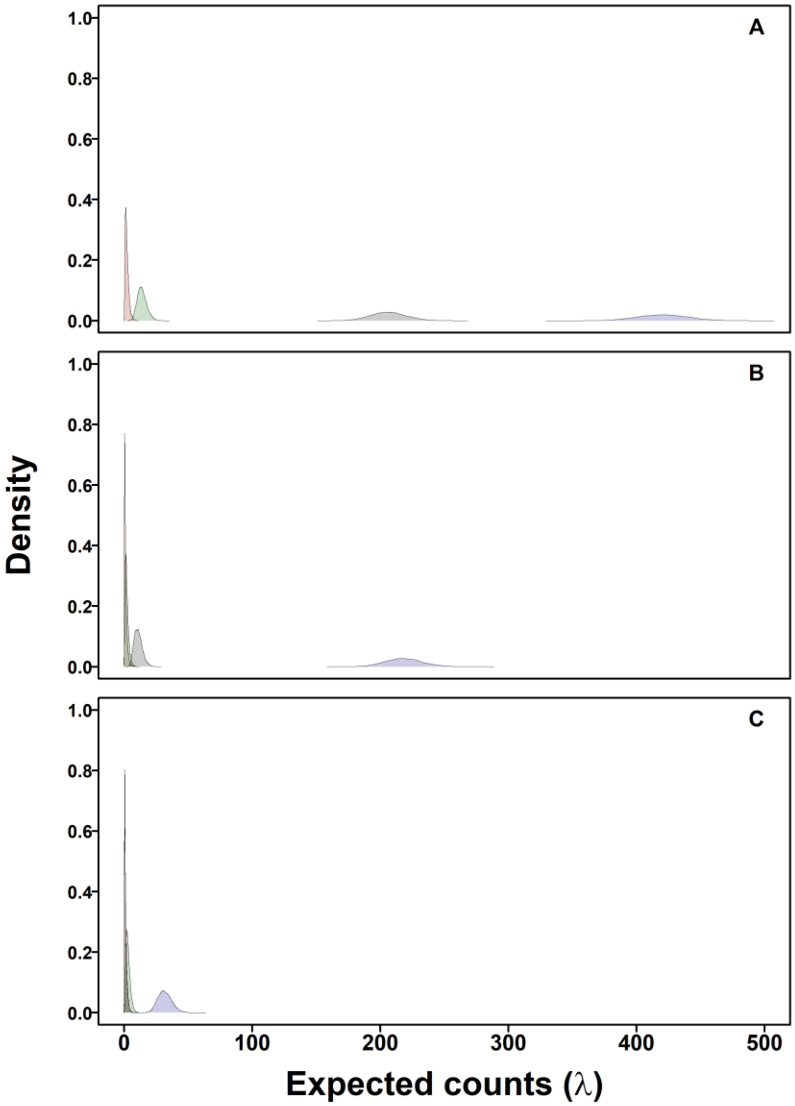

Supplementary Figure S14. Posterior densities of the expected counts (λ) for sources of information on seafood origin (Red= "Ads", Blue= "Label", Green= "Other", and Dark gray= "Retailer") per degree of seafood processing consumers prefer (from A to C: "Fresh" "Frozen", and "Processed"), extracted after 10^4 MCMC draws.

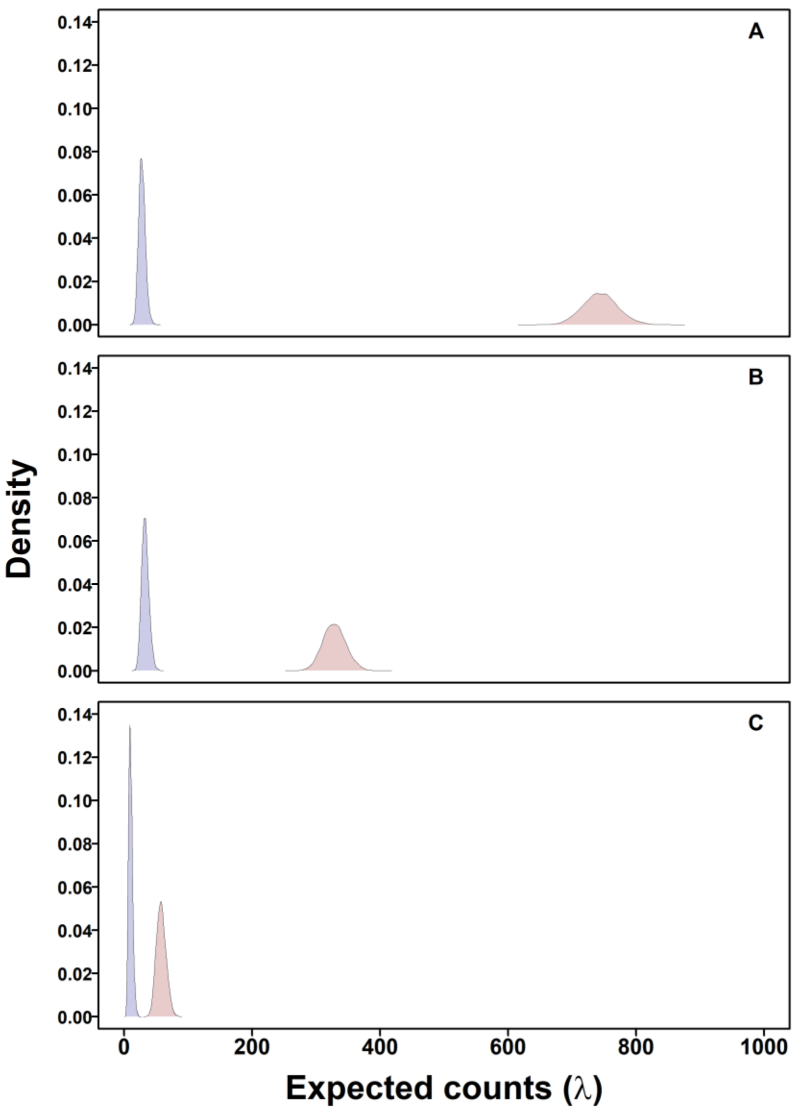

Supplementary Figure S15. Posterior densities of the expected counts (λ) for interest on seafood traceability (Red= "Yes", and Blue= "No") per degree of seafood processing consumers prefer (from A to C: "Fresh" "Frozen", and "Processed"), extracted after 10^4 MCMC draws.

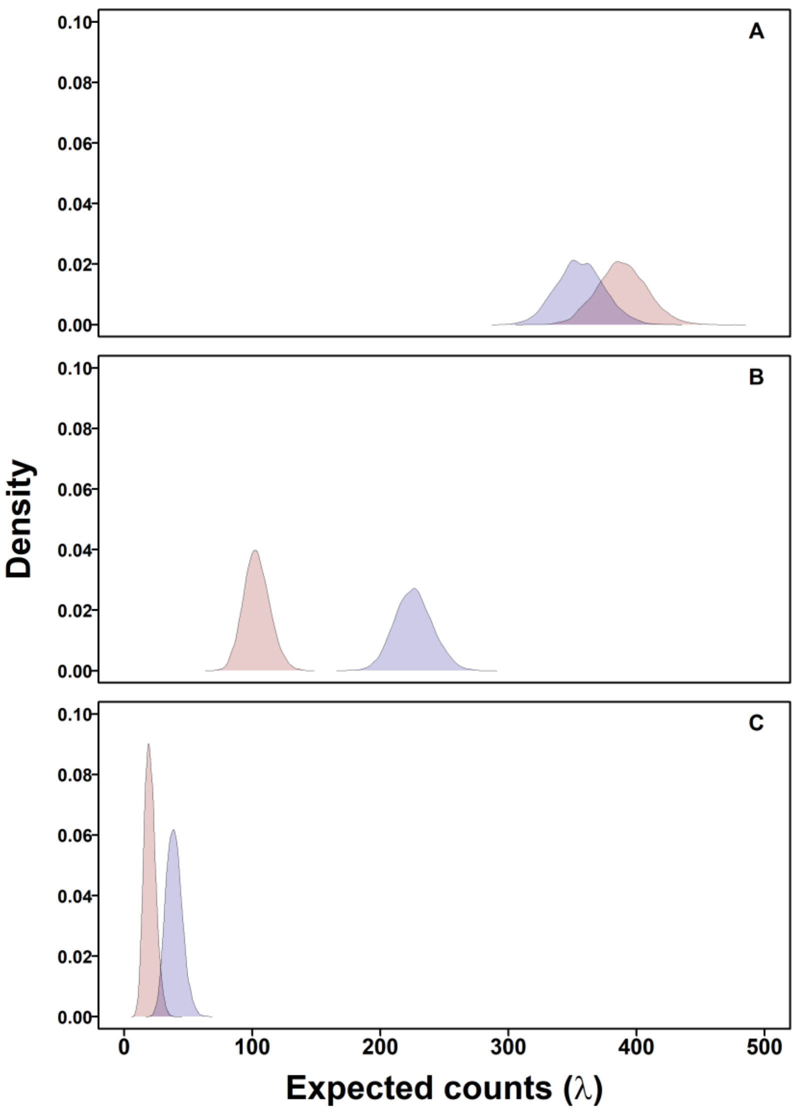

Supplementary Figure S16. Posterior densities of the expected counts (λ) for the consumption of Italian-farmed sea bass (Red= "Yes", and Blue= "No") per degree of seafood processing consumers prefer (from A to C: "Fresh" "Frozen", and "Processed"), extracted after 10^4 MCMC draws.

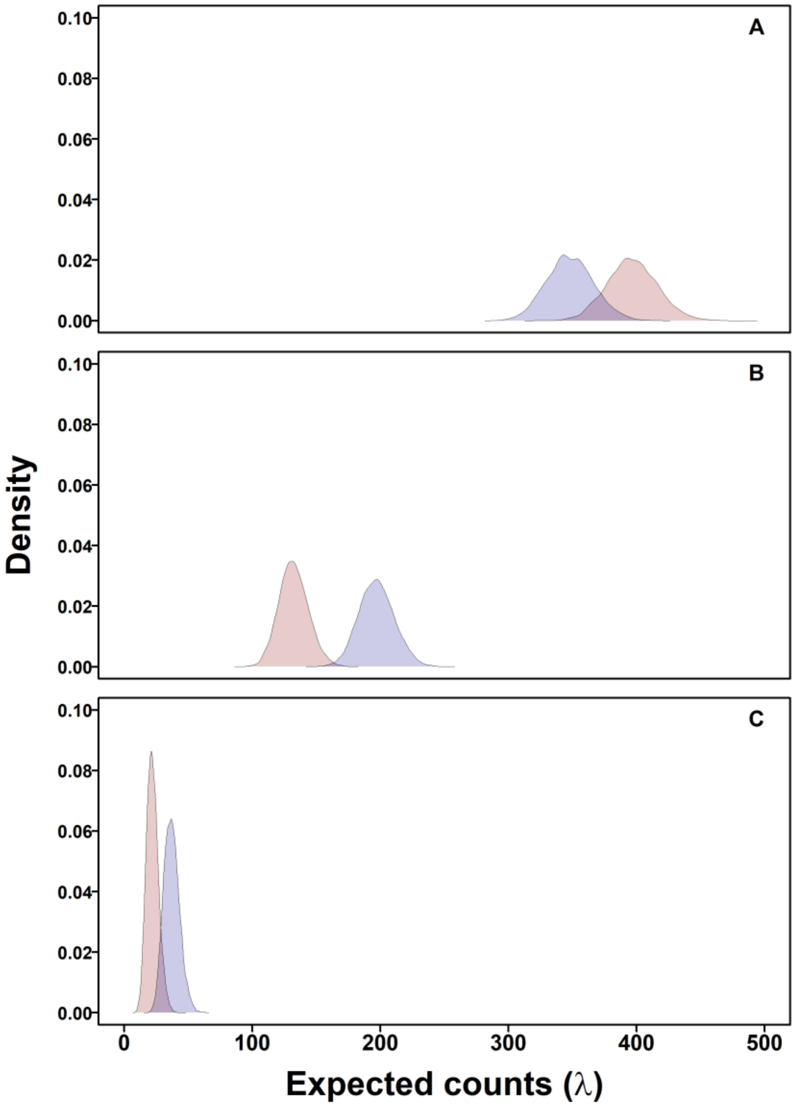

Supplementary Figure S17. Posterior densities of the expected counts (λ) for the consumption of striped venus clams (Red= "Yes", and Blue= "No") per degree of seafood processing consumers prefer (from A to C: "Fresh" "Frozen", and "Processed"), extracted after 10^4 MCMC draws.

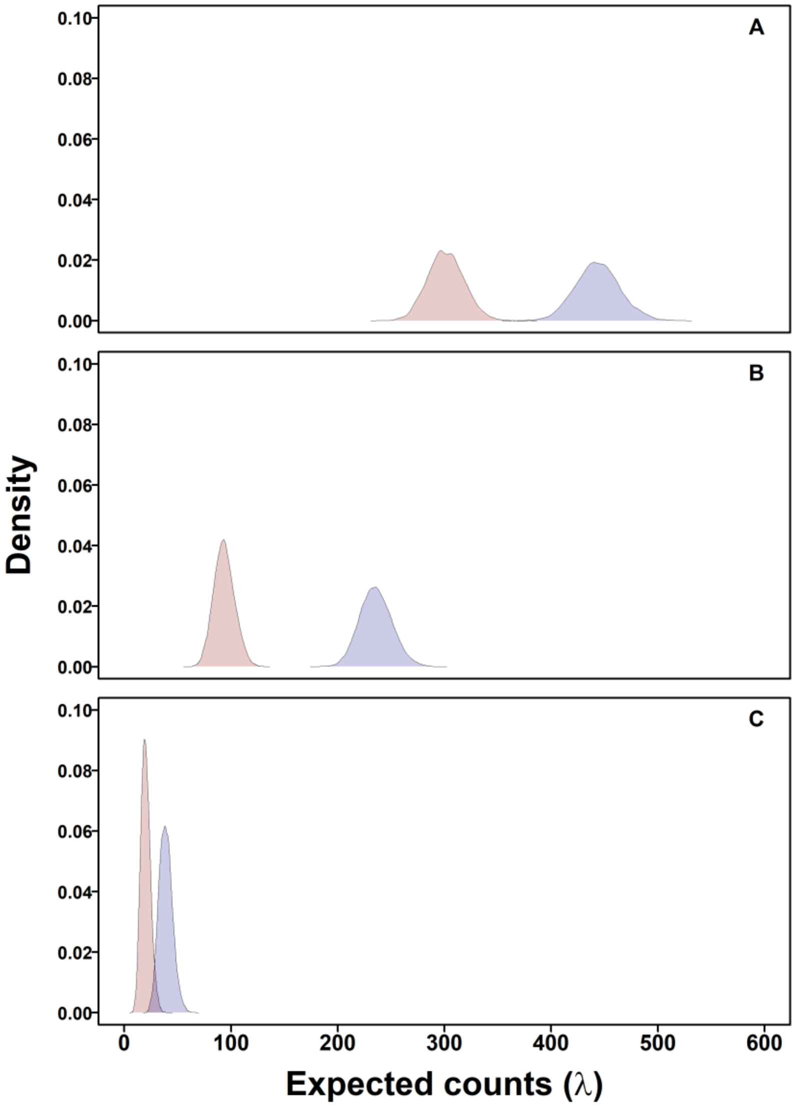

Supplementary Figure S18. Posterior densities of the expected counts (λ) for the consumption of giant red shrimp (Red= "Yes", and Blue= "No") per degree of seafood processing consumers prefer (from A to C: "Fresh" "Frozen", and "Processed"), extracted after 10^4 MCMC draws.

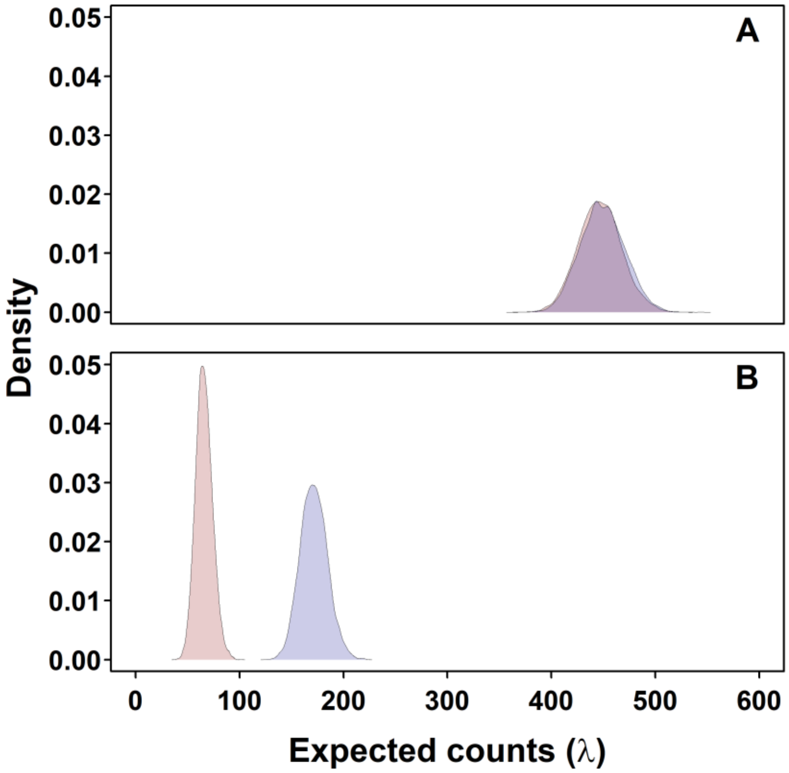

Supplementary Figure S19. Posterior densities of the expected counts (λ) for the consumption of Italian-farmed sea bass (Red= “Yes”, and Blue= “No”) per being informed on seafood origin (from A to B: “Yes”, and “No”), extracted after 10^4 MCMC draws.

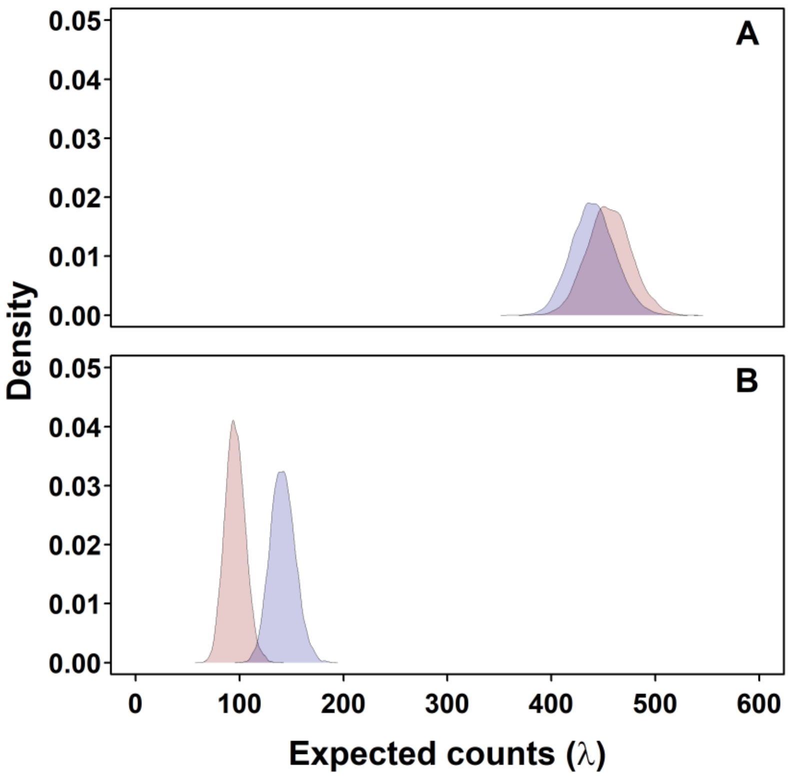

Supplementary Figure S20. Posterior densities of the expected counts (λ) for the consumption of striped venus clams (Red= "Yes", and Blue= "No") per being informed on seafood origin (from A to B: "Yes", and "No"), extracted after 10^4 MCMC draws.

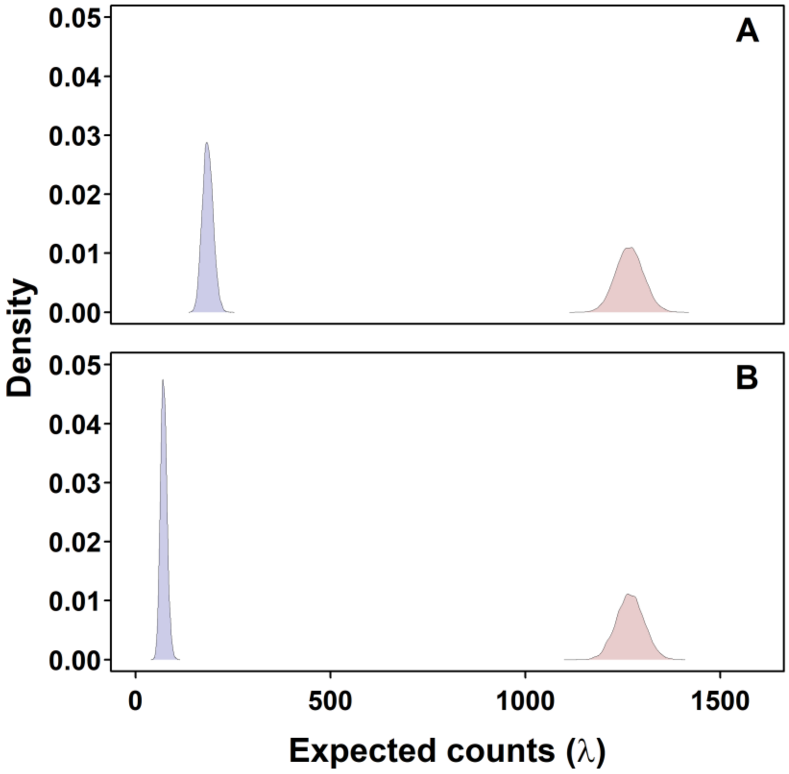

Supplementary Figure S21. Posterior densities of the expected counts (λ) for the consumption of seafood (Red= "Yes", and Blue= "No") per sex (from A to B: "Female", and "Male"), extracted after 10^4 MCMC draws.

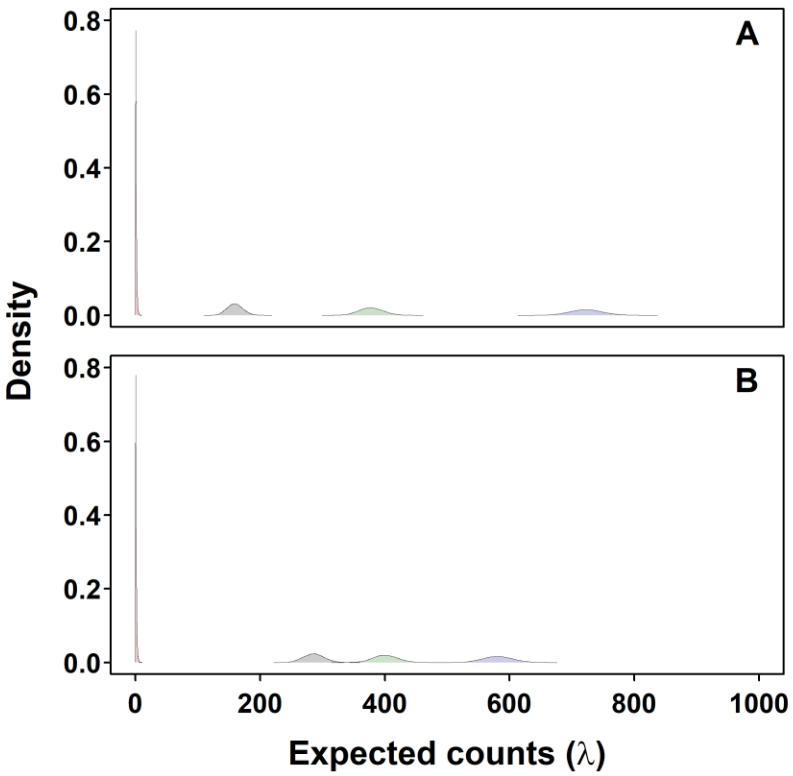

Supplementary Figure S22. Posterior densities of the expected counts (λ) for monthly seafood consumption rate (Red= 0, Blue= 1–5, Green= 6–10, and Dark gray= 10+) per sex (from A to B: “Female”, and “Male”), extracted after 10^4 MCMC draws.

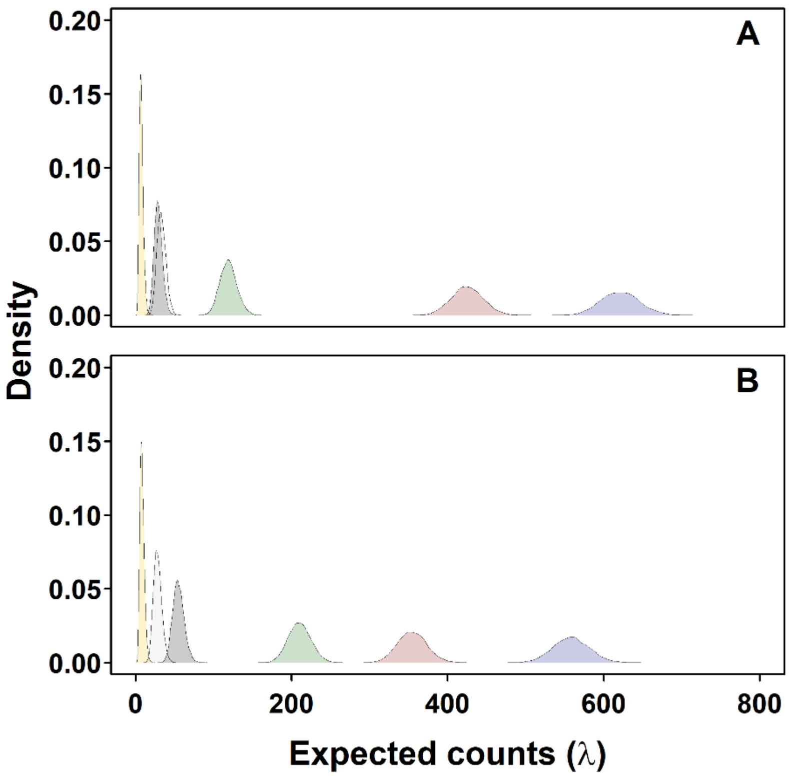

Supplementary Figure S23. Posterior densities of the expected counts (λ) for where consumers buy seafood (Red= "Fish market", Blue= "Large retail", Green= "Local market", Dark gray= "Not buy", Yellow= "Online", and Light gray = "Other") per sex (from A to B: "Female", and "Male"), extracted after 10^4 MCMC draws.

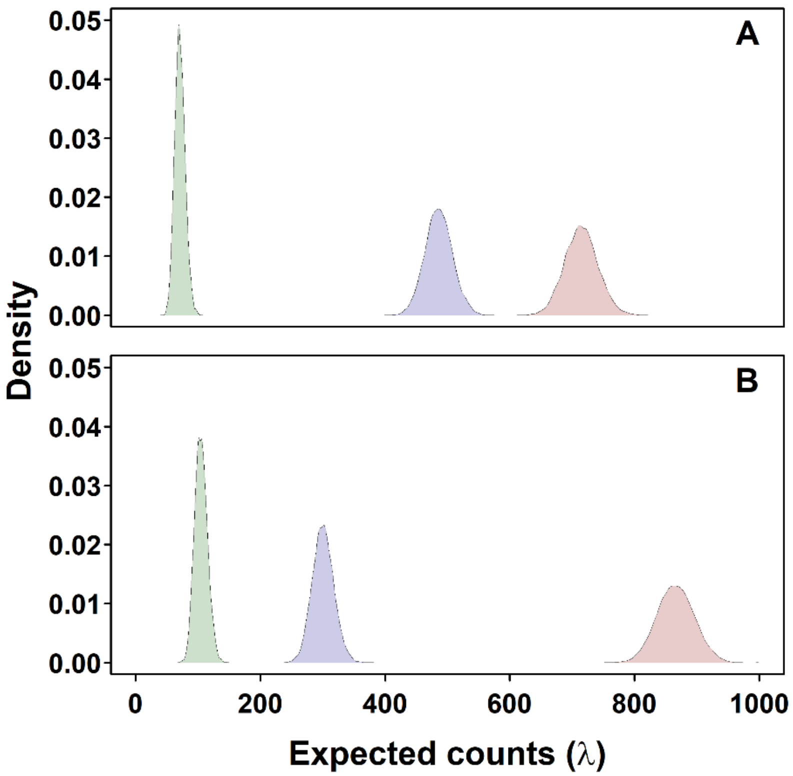

Supplementary Figure S24. Posterior densities of the expected counts (λ) for the degree of seafood processing consumers prefer (Red= "Fresh", Blue= "Frozen", and Green= "Processed") per sex (from A to B: "Female", and "Male"), extracted after 10^4 MCMC draws.

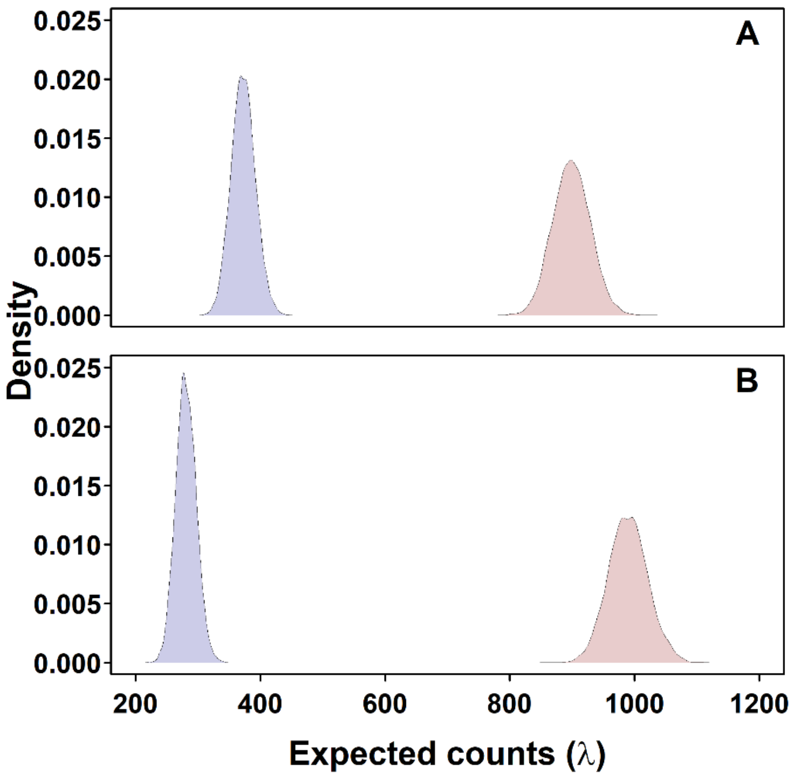

Supplementary Figure S25. Posterior densities of the expected counts (λ) for being informed on seafood origin (Red= "Yes", and Blue= "No") per sex (from A to B: "Female", and "Male"), extracted after 10^4 MCMC draws.

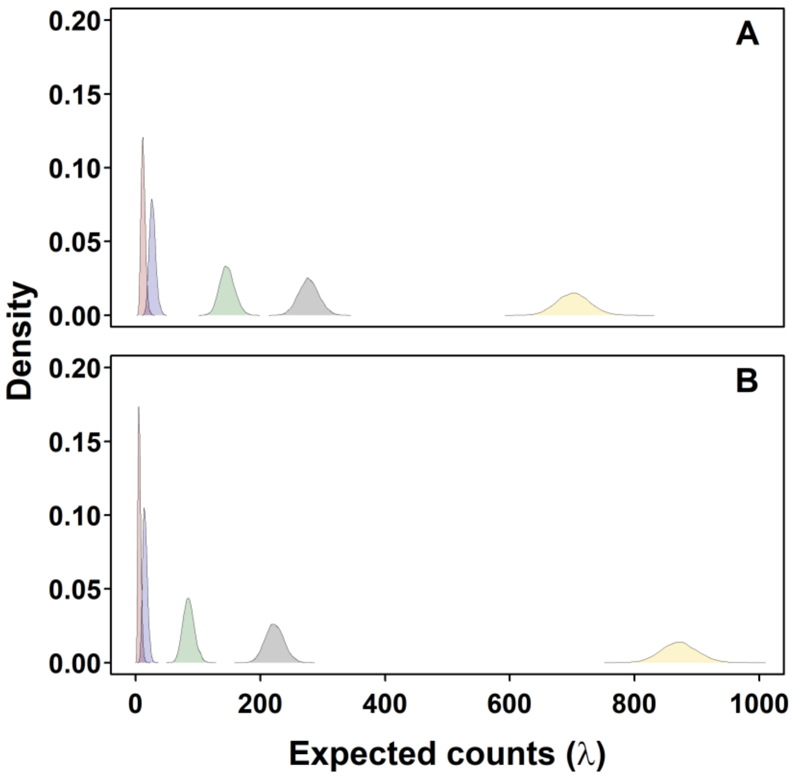

Supplementary Figure S26. Posterior densities of the expected counts (λ) for level of consumers' interest in seafood traceability (Red= 1, Blue= 2, Green= 3, Dark gray= 4, and Yellow= 5) per sex (from A to B: "Female", and "Male"), extracted after 10^4 MCMC draws.

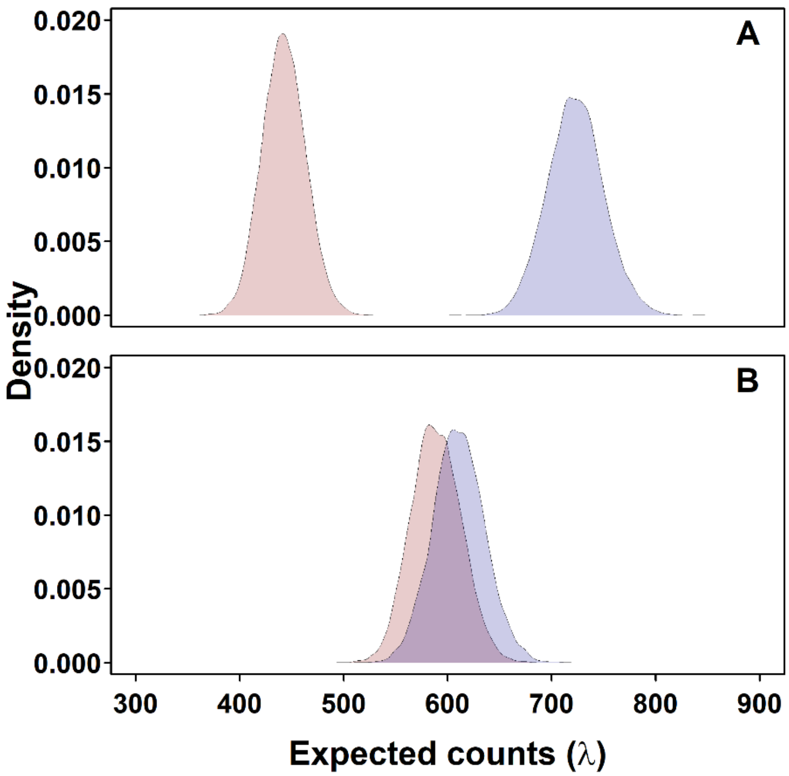

Supplementary Figure S27. Posterior densities of the expected counts (λ) for the consumption of Italian-farmed sea bass (Red= “Yes”, and Blue= “No”) per sex (from A to B: “Female”, and “Male”), extracted after 10^4 MCMC draws.

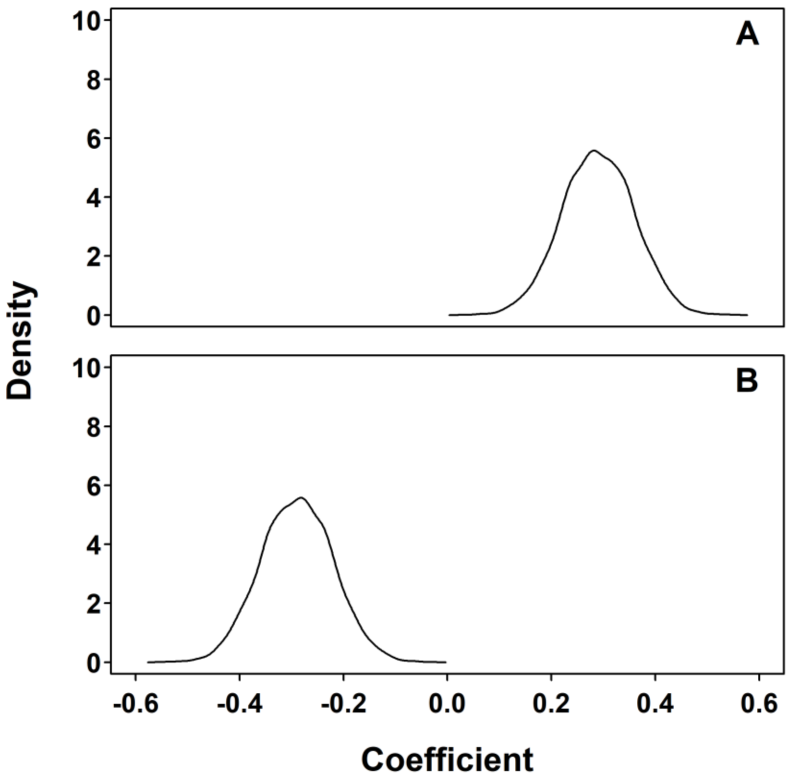

Supplementary Figure S28. Posterior densities of the ANOVA coefficients for the consumers' WTP for Italian-farmed sea bass per sex (from A to B: "Female", and "Male"), extracted after 10^4 MCMC draws.

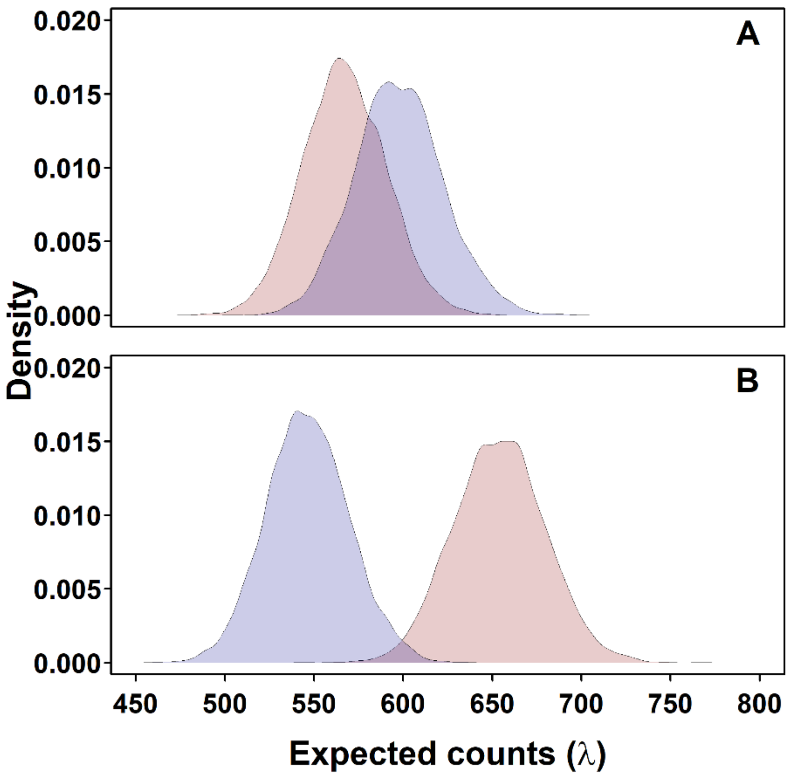

Supplementary Figure S29. Posterior densities of the expected counts (λ) for the consumption of striped venus clams (Red= "Yes", and Blue= "No") per sex (from A to B: "Female", and "Male"), extracted after 10^4 MCMC draws.

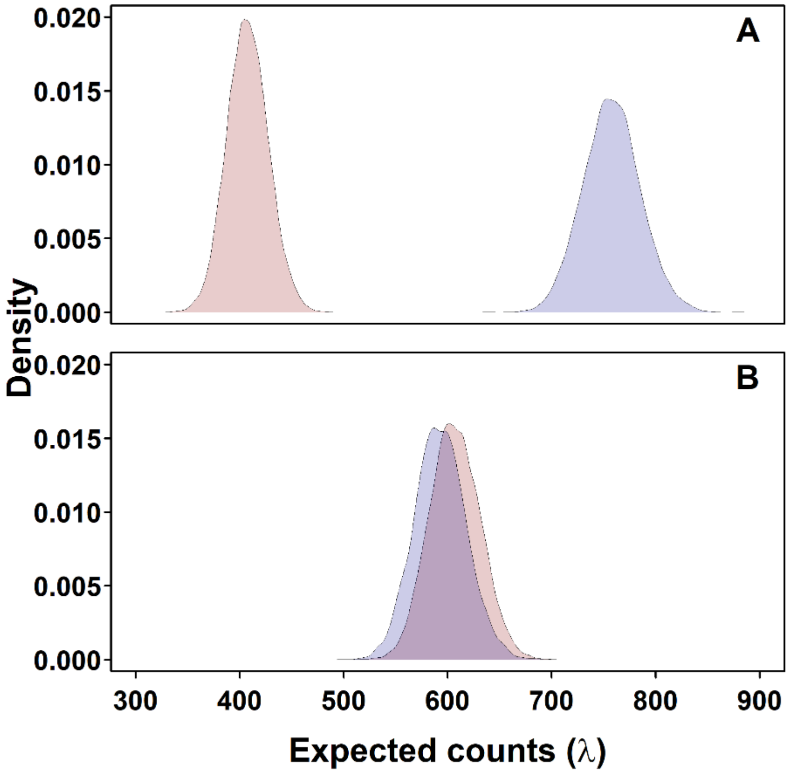

Supplementary Figure S30. Posterior densities of the expected counts (λ) for the consumption of giant red shrimp (Red= "Yes", and Blue= "No") per sex (from A to B: "Female", and "Male"), extracted after 10^4 MCMC draws.

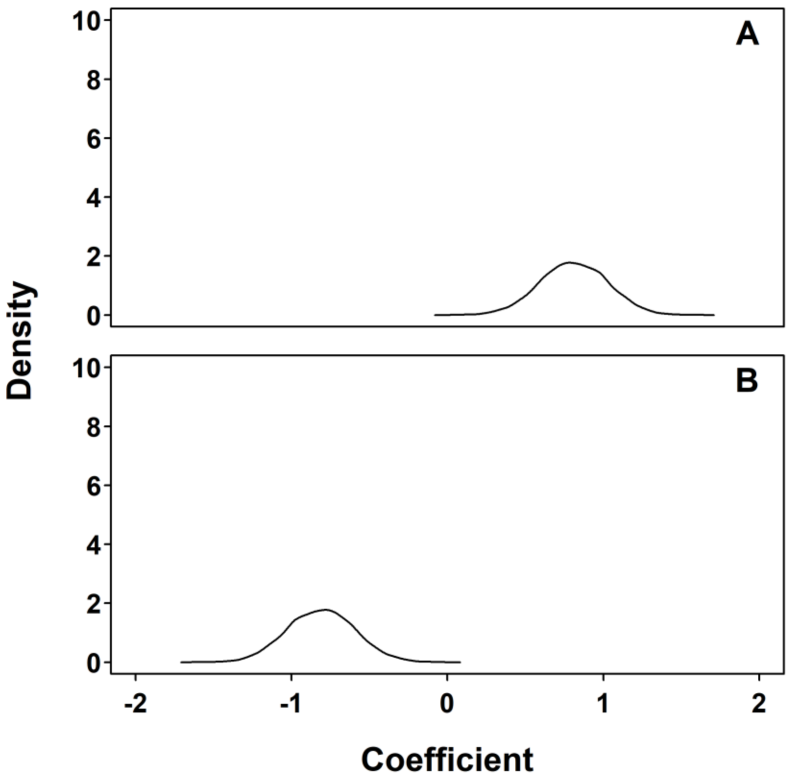

Supplementary Figure S31. Posterior densities of the ANOVA coefficients for the consumers' WTP for red giant shrimp per sex (from A to B: "Female", and "Male"), extracted after 10^4 MCMC draws.

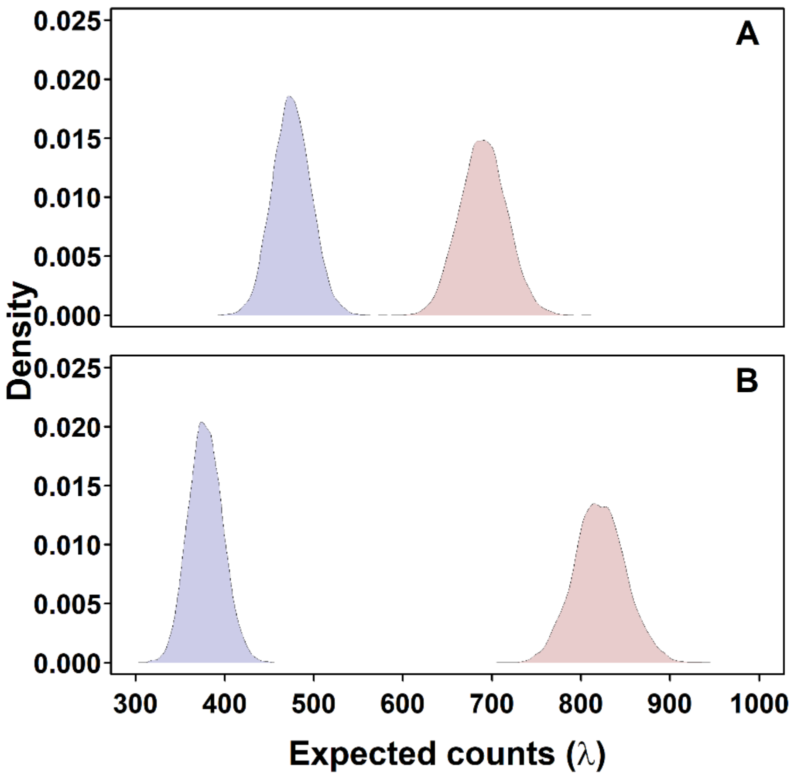

Supplementary Figure S32. Posterior densities of the expected counts (λ) for the consumption of processed albacore tuna (Red= "Yes", and Blue= "No") per sex (from A to B: "Female", and "Male"), extracted after 10^4 MCMC draws.

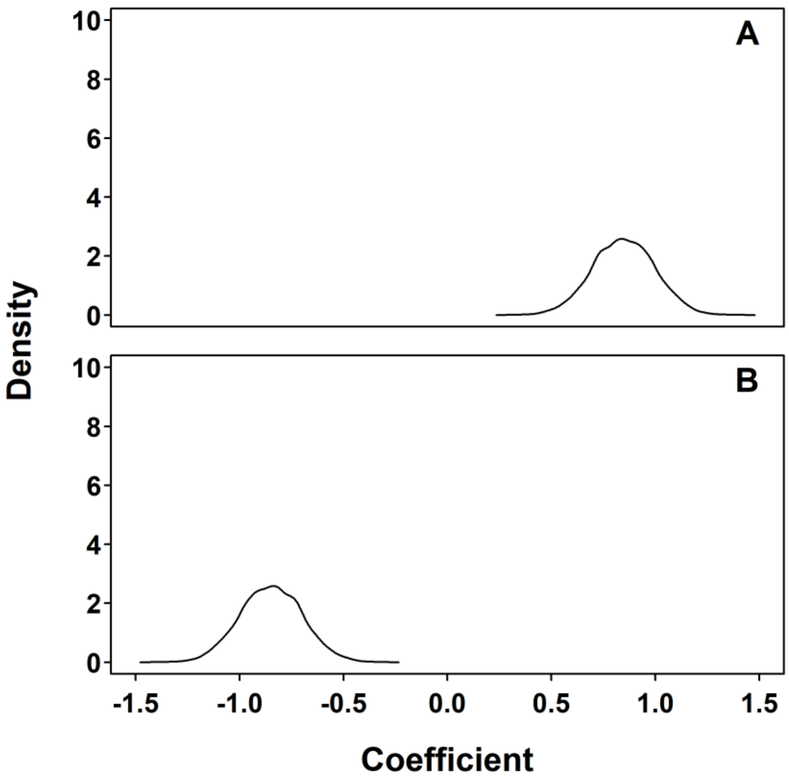

Supplementary Figure S33. Posterior densities of the ANOVA coefficients for the consumers' WTP for processed albacore tuna per sex (from A to B: "Female", and "Male"), extracted after 10^4 MCMC draws.

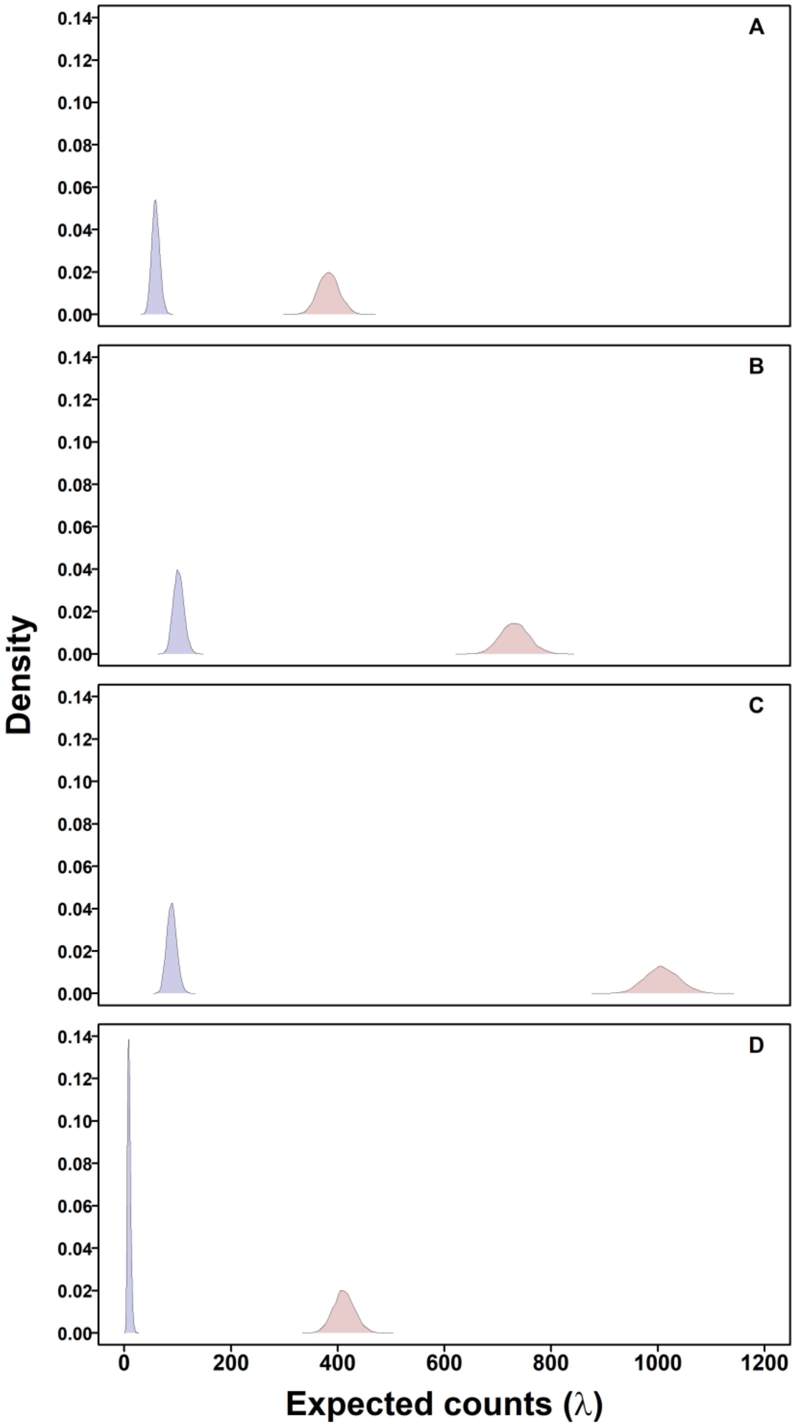

Supplementary Figure S34. Posterior densities of the expected counts (λ) for consumption of seafood (Red= "Yes" and Blue= "No") per age range (from A to D: 18–25, 26–40, 41–65, and 66+), extracted after 10^4 MCMC draws.

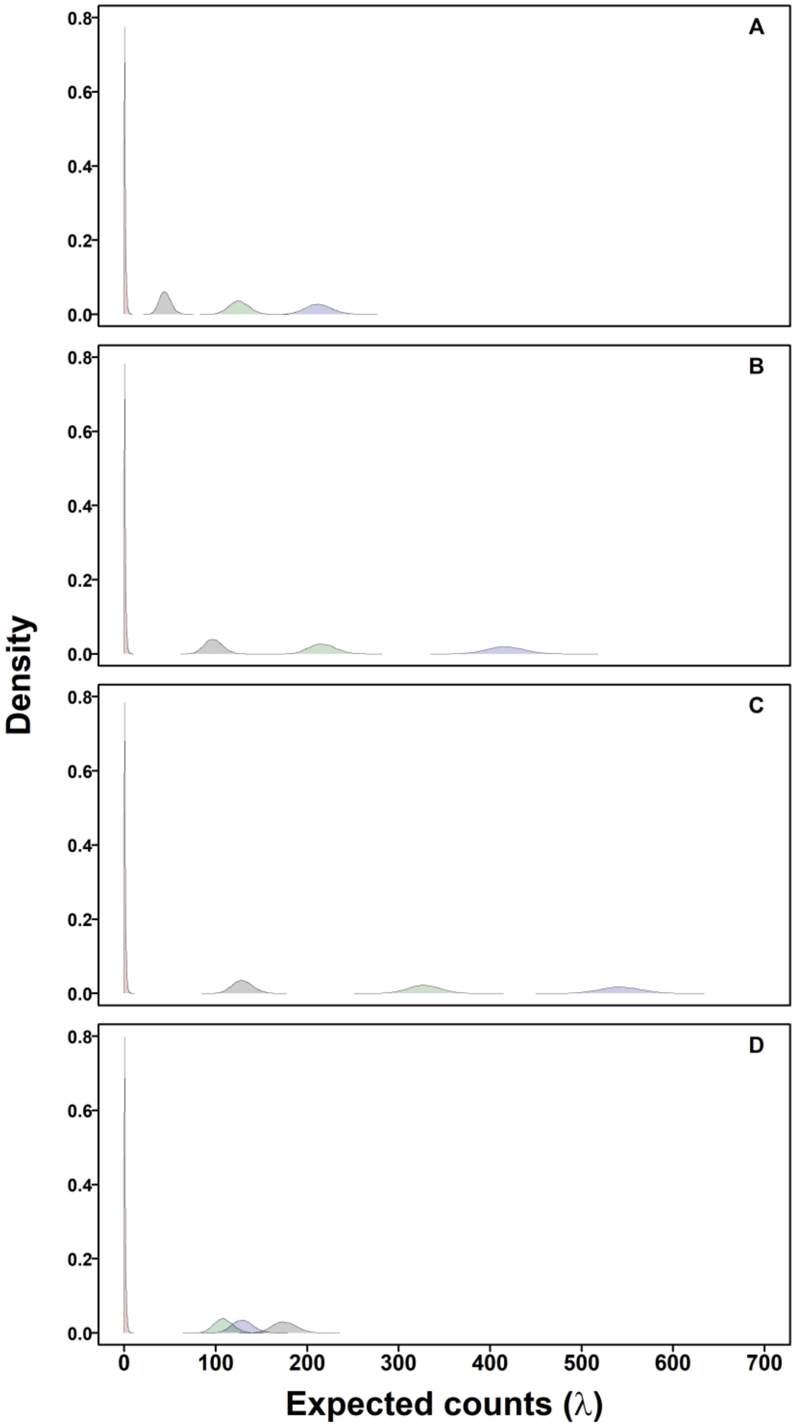

Supplementary Figure S35. Posterior densities of the expected counts (λ) for monthly seafood consumption rate (Red= 0, Blue= 1–5, Green= 6–10, and Dark gray= 10+) per age range (from A to D: 18–25, 26–40, 41–65, and 66+), extracted after 10^4 MCMC draws.

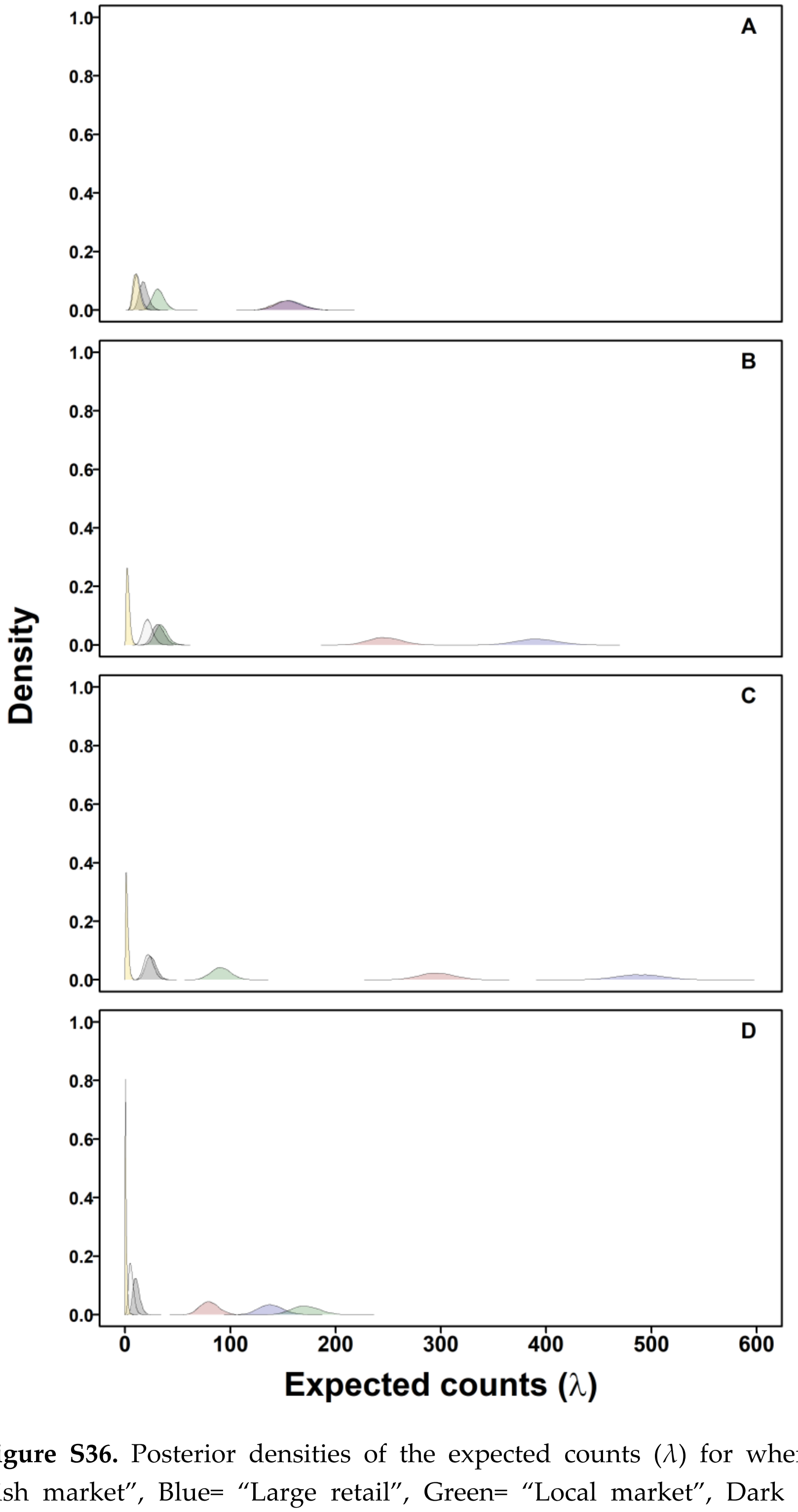

Supplementary Figure S36. Posterior densities of the expected counts (λ) for where consumers buy seafood (Red= "Fish market", Blue= "Large retail", Green= "Local market", Dark gray= "Not buy", Yellow= "Online", and Light gray = "Other") per age range (from A to D: 18–25, 26–40, 41–65, and 66+), extracted after 10^4 MCMC draws.

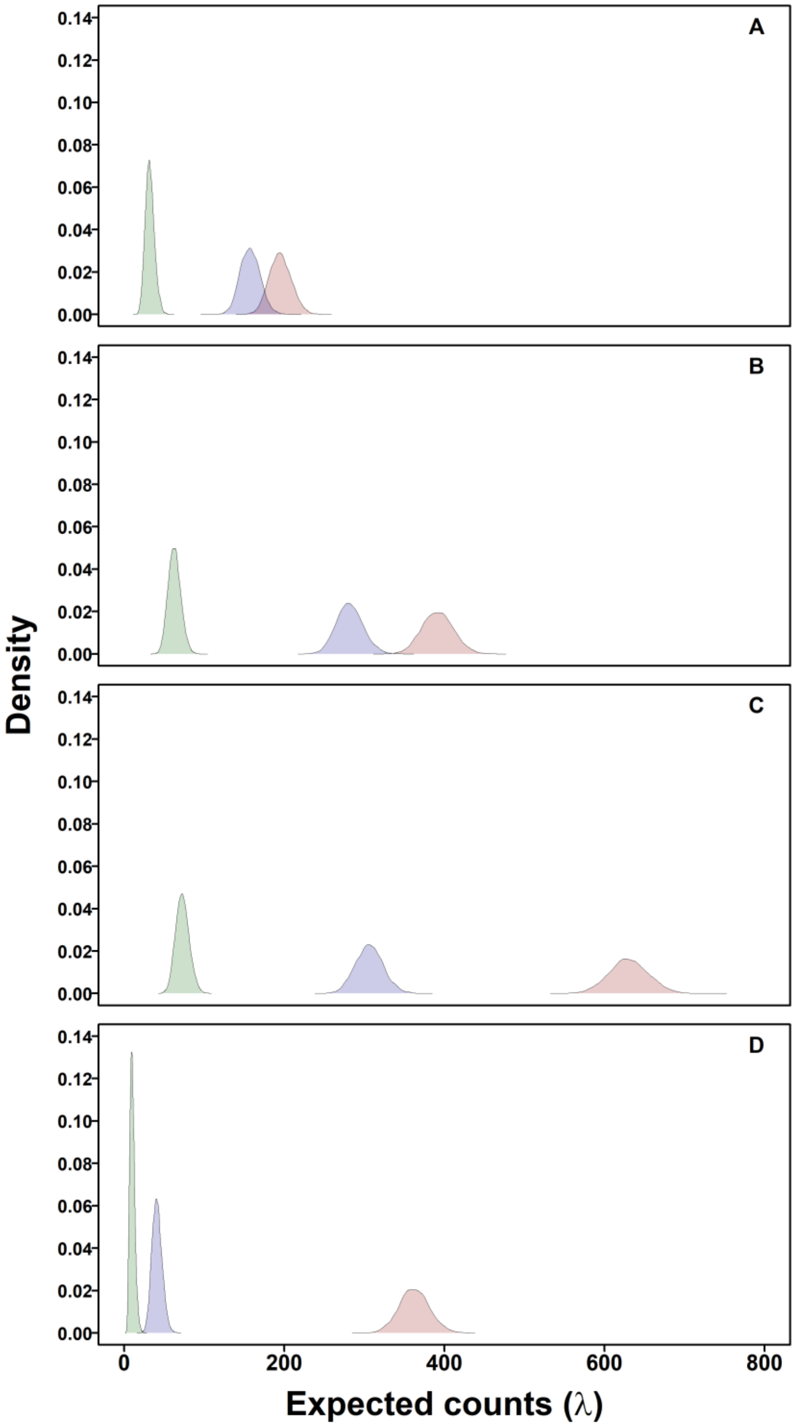

Supplementary Figure S37. Posterior densities of the expected counts (λ) for the degree of seafood processing consumers prefer (Red= "Fresh", Blue= "Frozen", and Green= "Processed") per age range (from A to D: 18–25, 26–40, 41–65, and 66+), extracted after 10^4 MCMC draws.

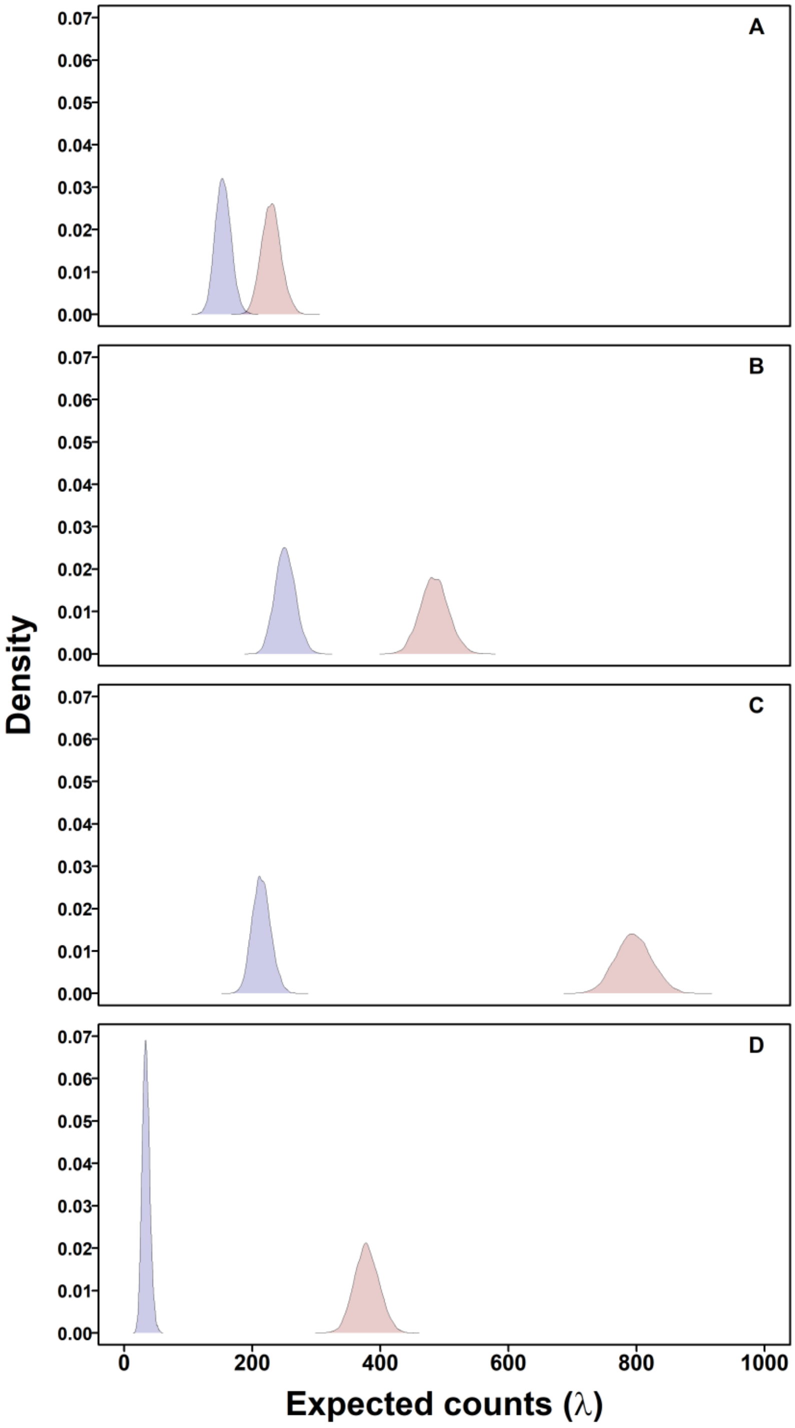

Supplementary Figure S38. Posterior densities of the expected counts (λ) for being informed on seafood origin (Red= “Yes”, and Blue= “No”) per age range (from A to D: 18–25, 26–40, 41–65, and 66+), extracted after 10^4 MCMC draws.

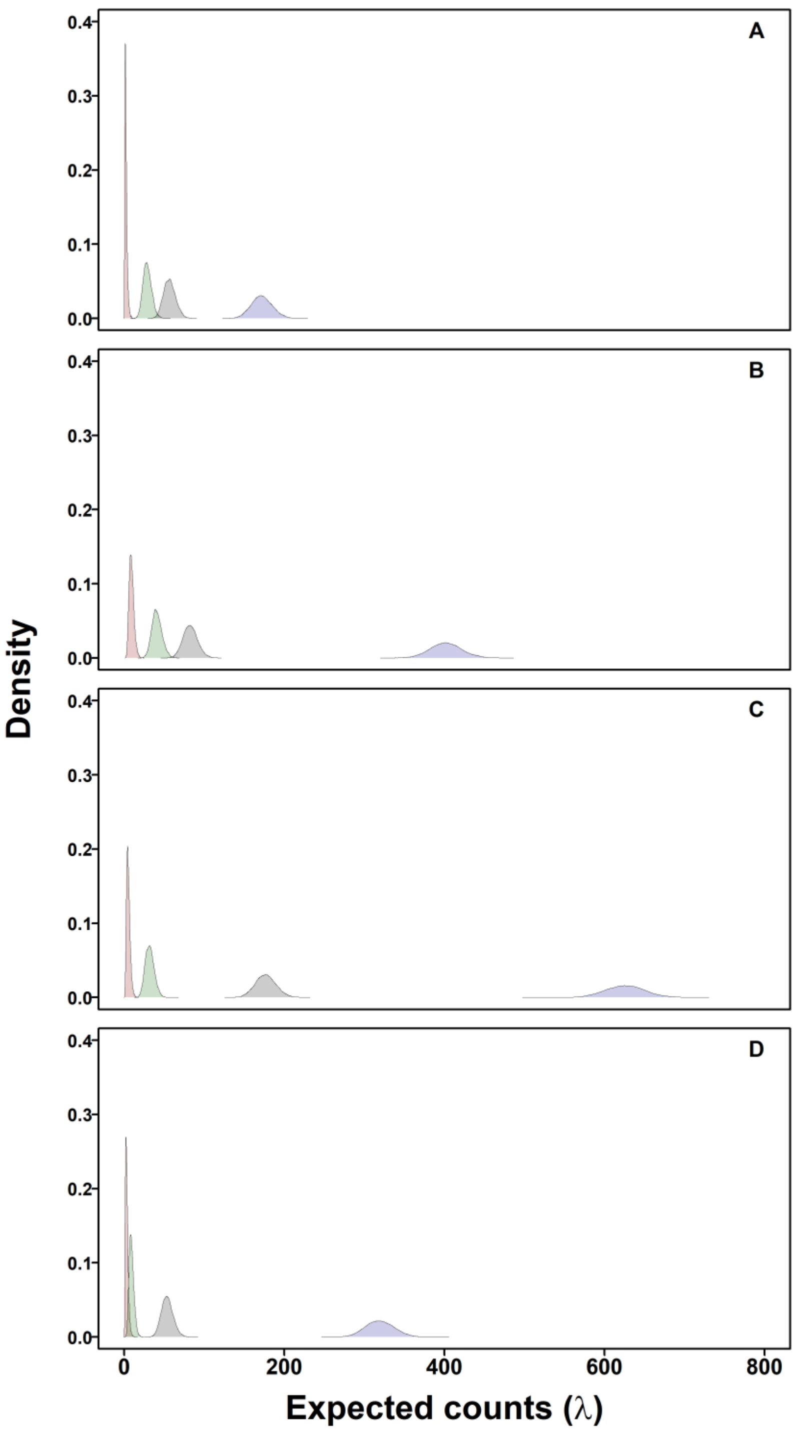

Supplementary Figure S39. Posterior densities of the expected counts (λ) for sources of information on seafood origin (Red= "Ads", Blue= "Label", Green= "Other", and Dark gray= "Retailer") per age range (from A to D: 18–25, 26–40, 41–65, and 66+), extracted after 10^4 MCMC draws.

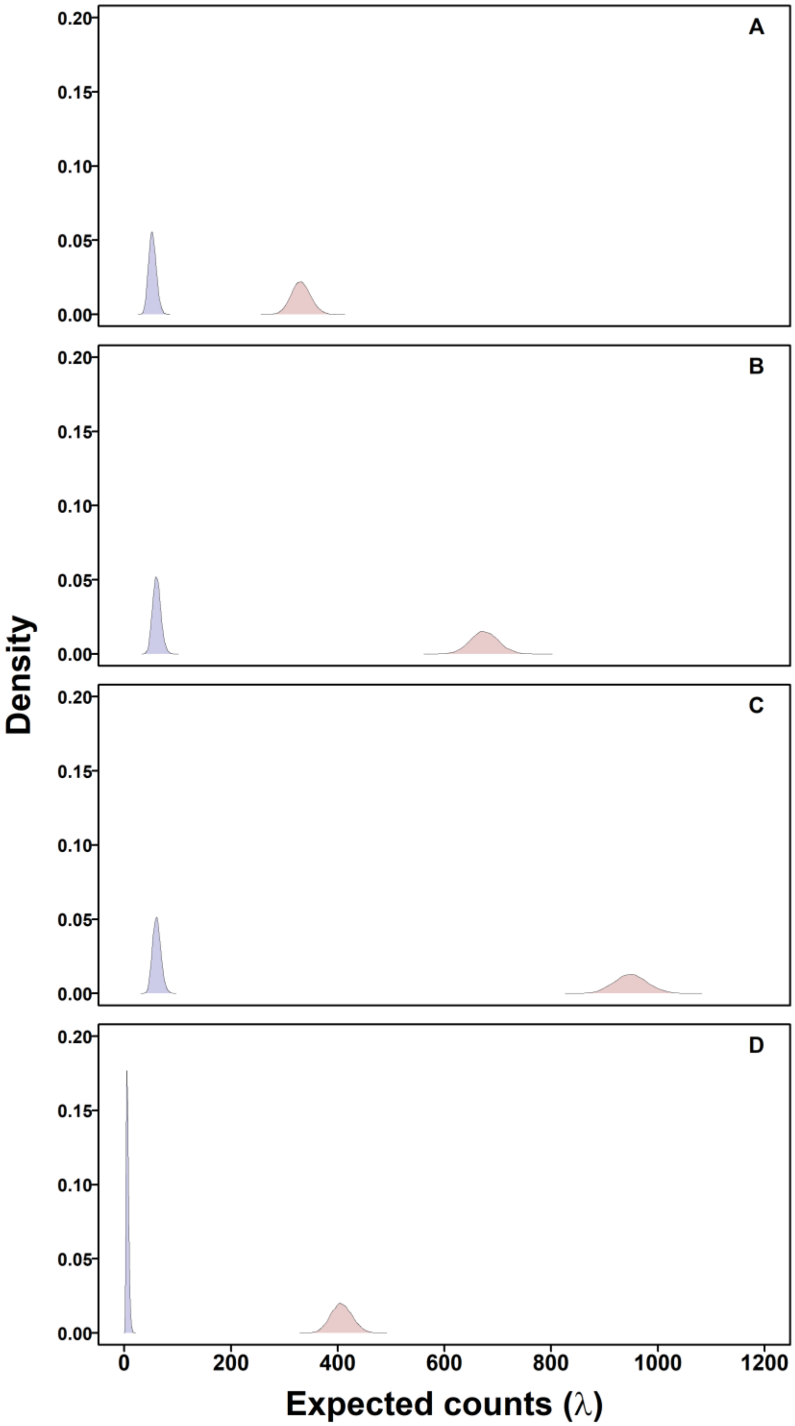

Supplementary Figure S40. Posterior densities of the expected counts (λ) for interest on seafood traceability (Red= "Yes", and Blue= "No") per age range (from A to D: 18–25, 26–40, 41–65, and 66+), extracted after 10^4 MCMC draws.

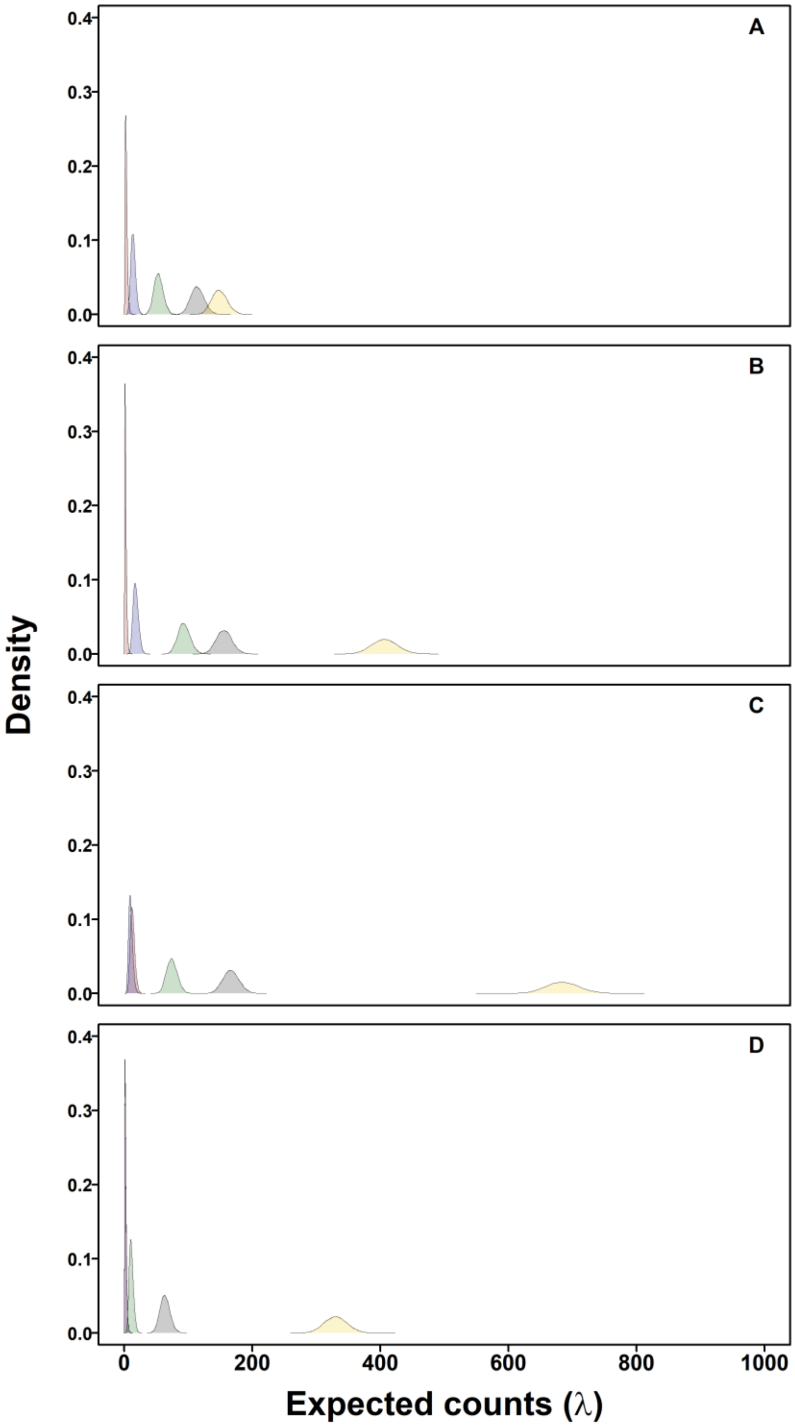

Supplementary Figure S41. Posterior densities of the expected counts (λ) for level of consumers' interest in seafood traceability (Red= 1, Blue= 2, Green= 3, Dark gray= 4, and Yellow= 5) per age range (from A to D: 18–25, 26–40, 41–65, and 66+), extracted after 10^4 MCMC draws.

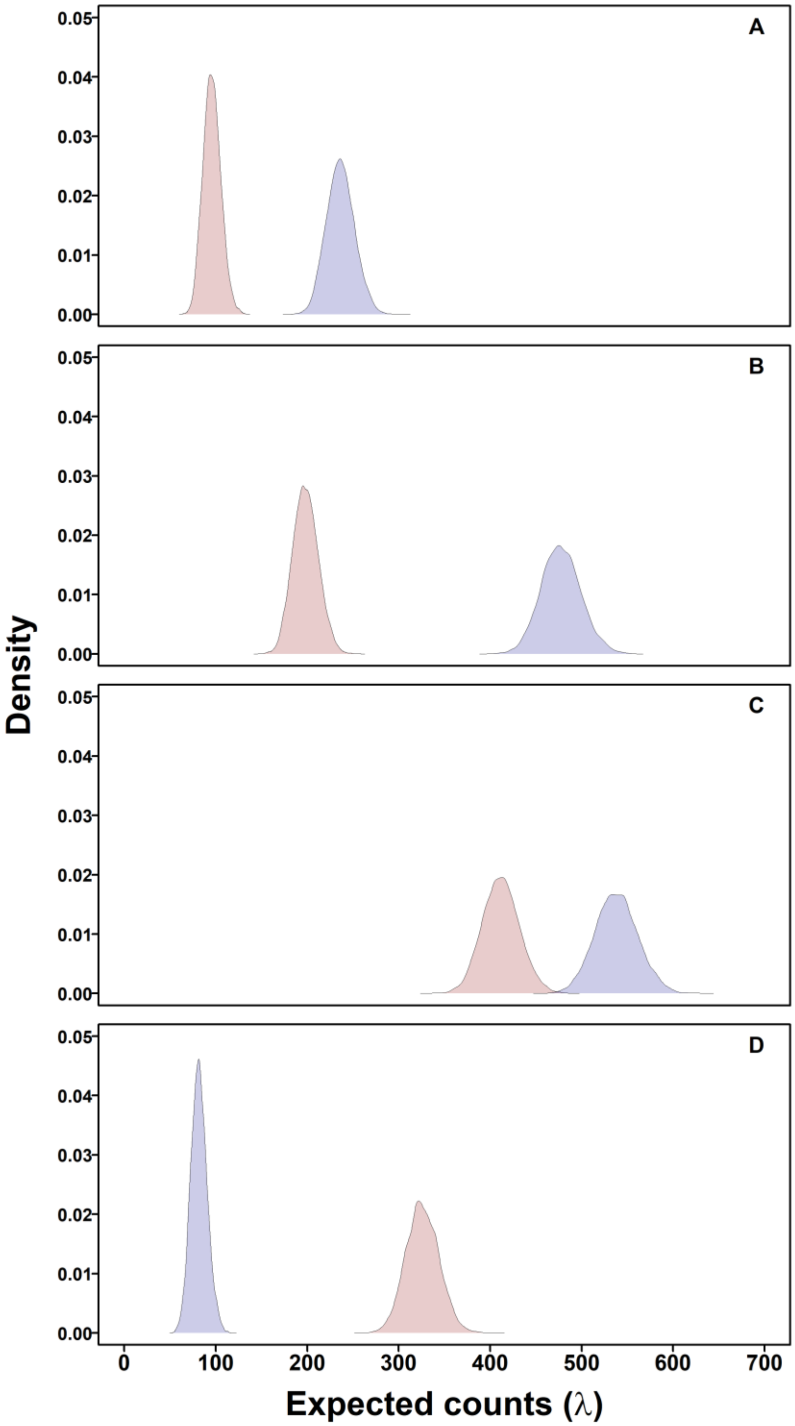

Supplementary Figure S42. Posterior densities of the expected counts (λ) for the consumption of Italian-farmed sea bass (Red= "Yes", and Blue= "No") per age range (from A to D: 18–25, 26–40, 41–65, and 66+), extracted after 10^4 MCMC draws.

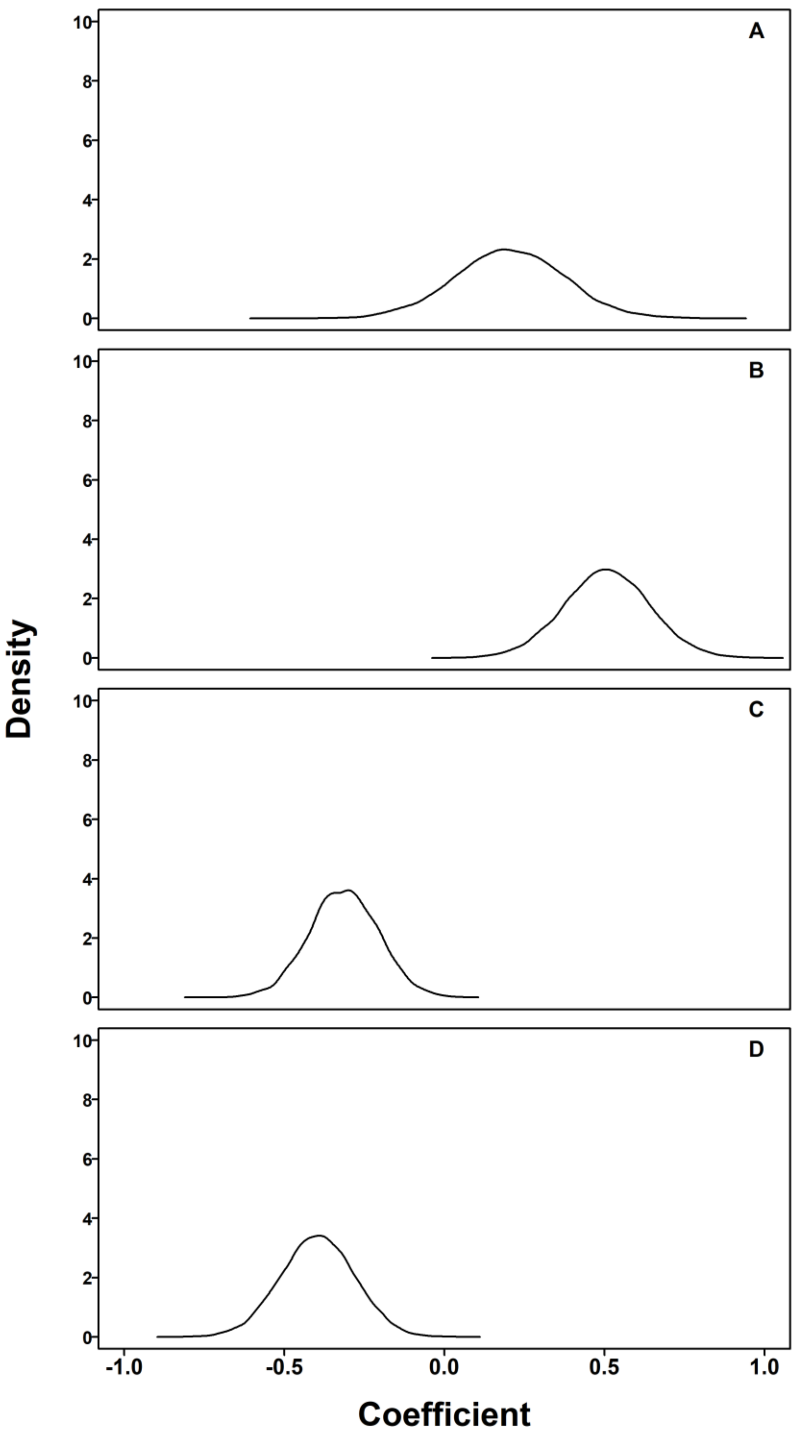

Supplementary Figure S43. Posterior densities of the ANOVA coefficients for the consumers' WTP for Italian-farmed sea bass per age range (from A to D: 18–25, 26–40, 41–65, and 66+), extracted after 10^4 MCMC draws.

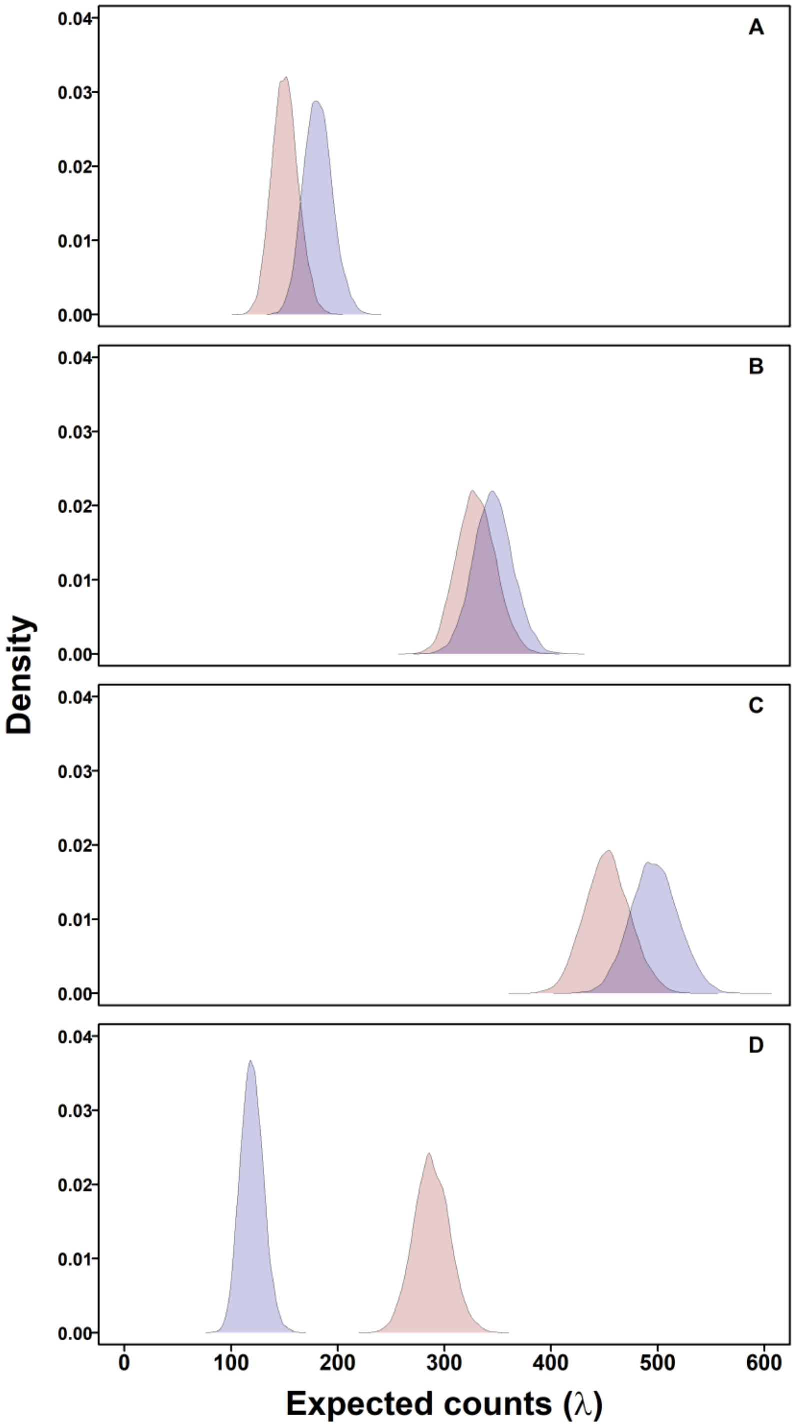

Supplementary Figure S44. Posterior densities of the expected counts (λ) for the consumption of striped venus clams (Red= "Yes", and Blue= "No") per age range (from A to D: 18–25, 26–40, 41–65, and 66+), extracted after 10^4 MCMC draws.

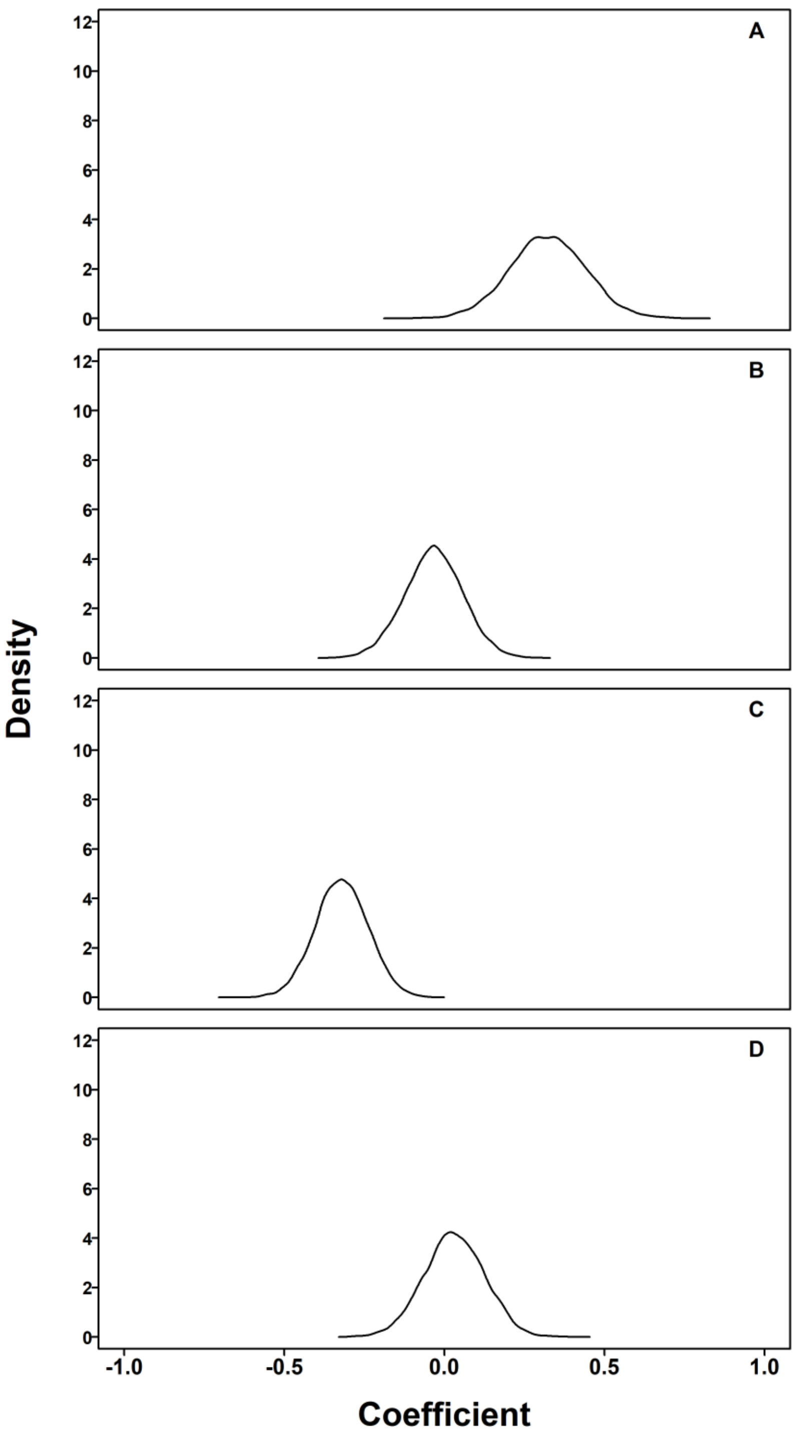

Supplementary Figure S45. Posterior densities of the ANOVA coefficients for the consumers' WTP for striped venus clams per age range (from A to D: 18–25, 26–40, 41–65, and 66+), extracted after 10^4 MCMC draws.

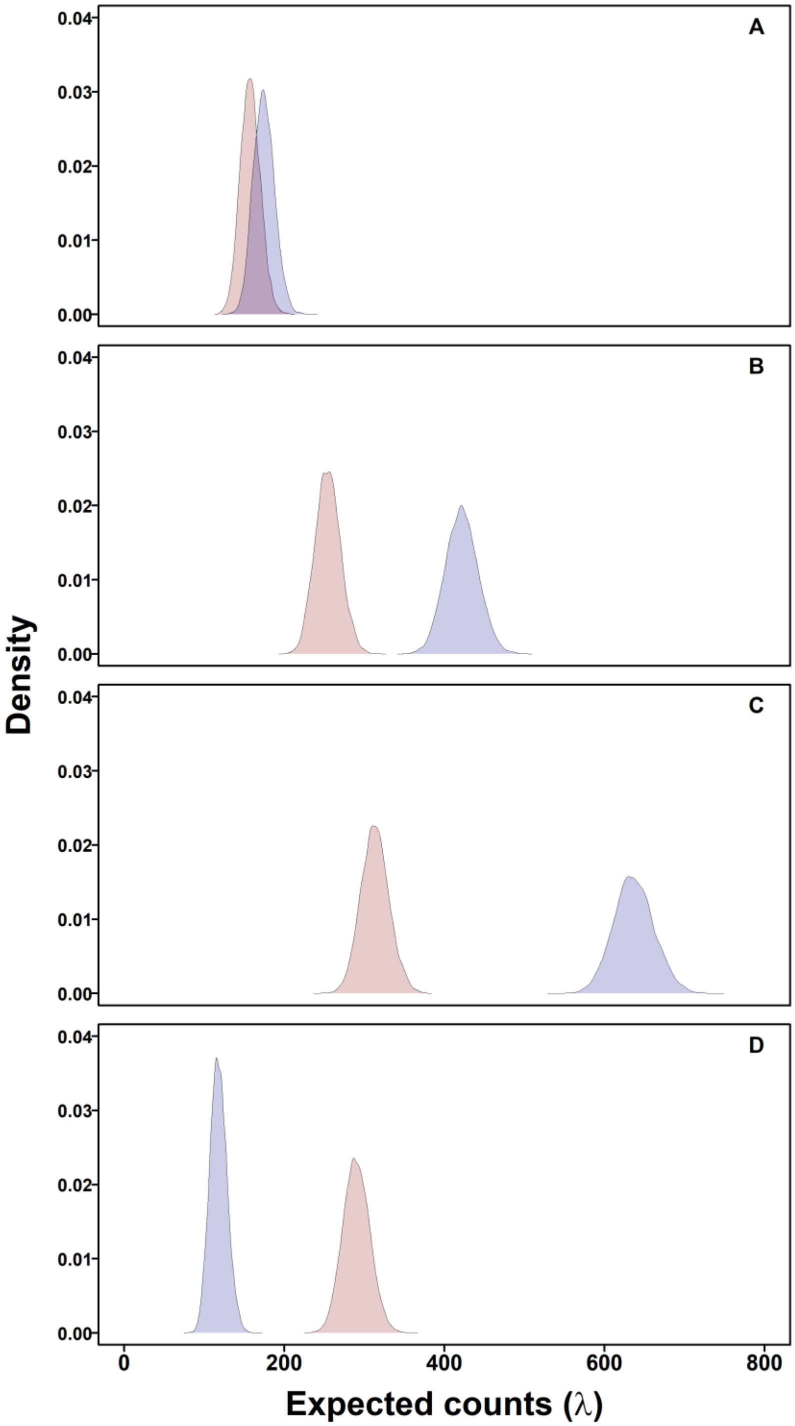

Supplementary Figure S46. Posterior densities of the expected counts (λ) for the consumption of giant red shrimp (Red= "Yes", and Blue= "No") per age range (from A to D: 18–25, 26–40, 41–65, and 66+), extracted after 10^4 MCMC draws.

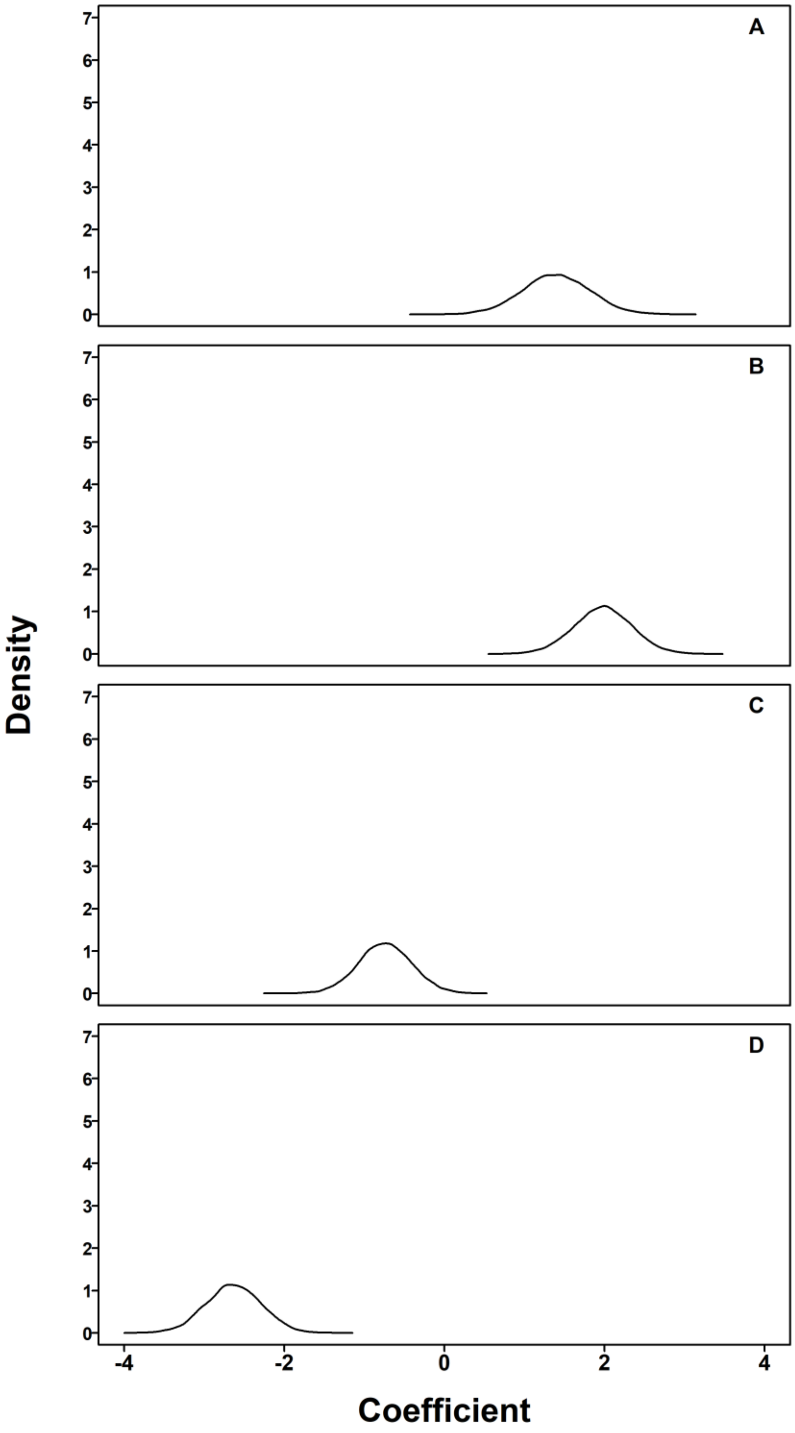

Supplementary Figure S47. Posterior densities of the ANOVA coefficients for the consumers' WTP for giant red shrimp per age range (from A to D: 18–25, 26–40, 41–65, and 66+), extracted after 10^4 MCMC draws.

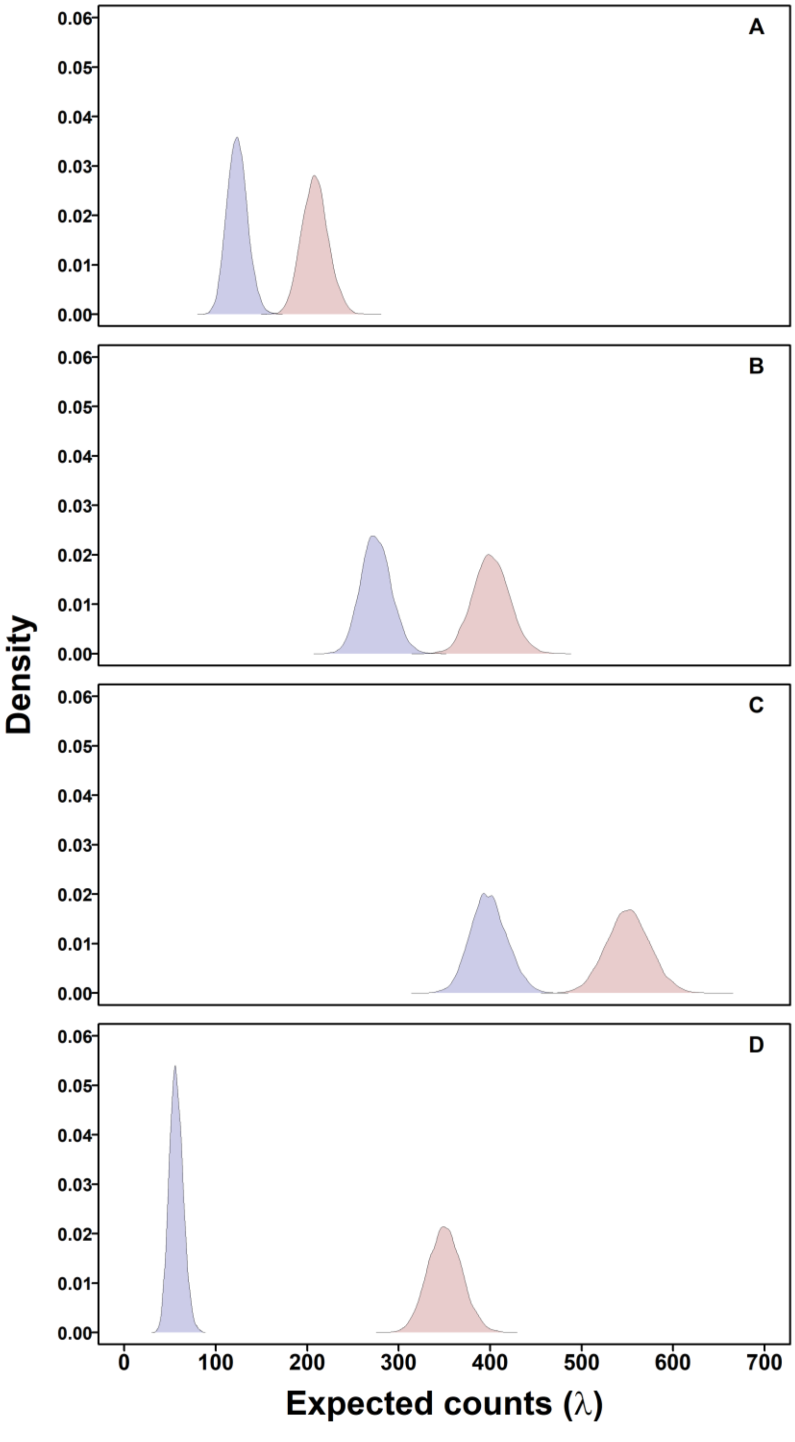

Supplementary Figure S48. Posterior densities of the expected counts (λ) for the consumption of processed albacore tuna (Red= "Yes", and Blue= "No") per age range (from A to D: 18–25, 26–40, 41–65, and 66+), extracted after 10^4 MCMC draws.

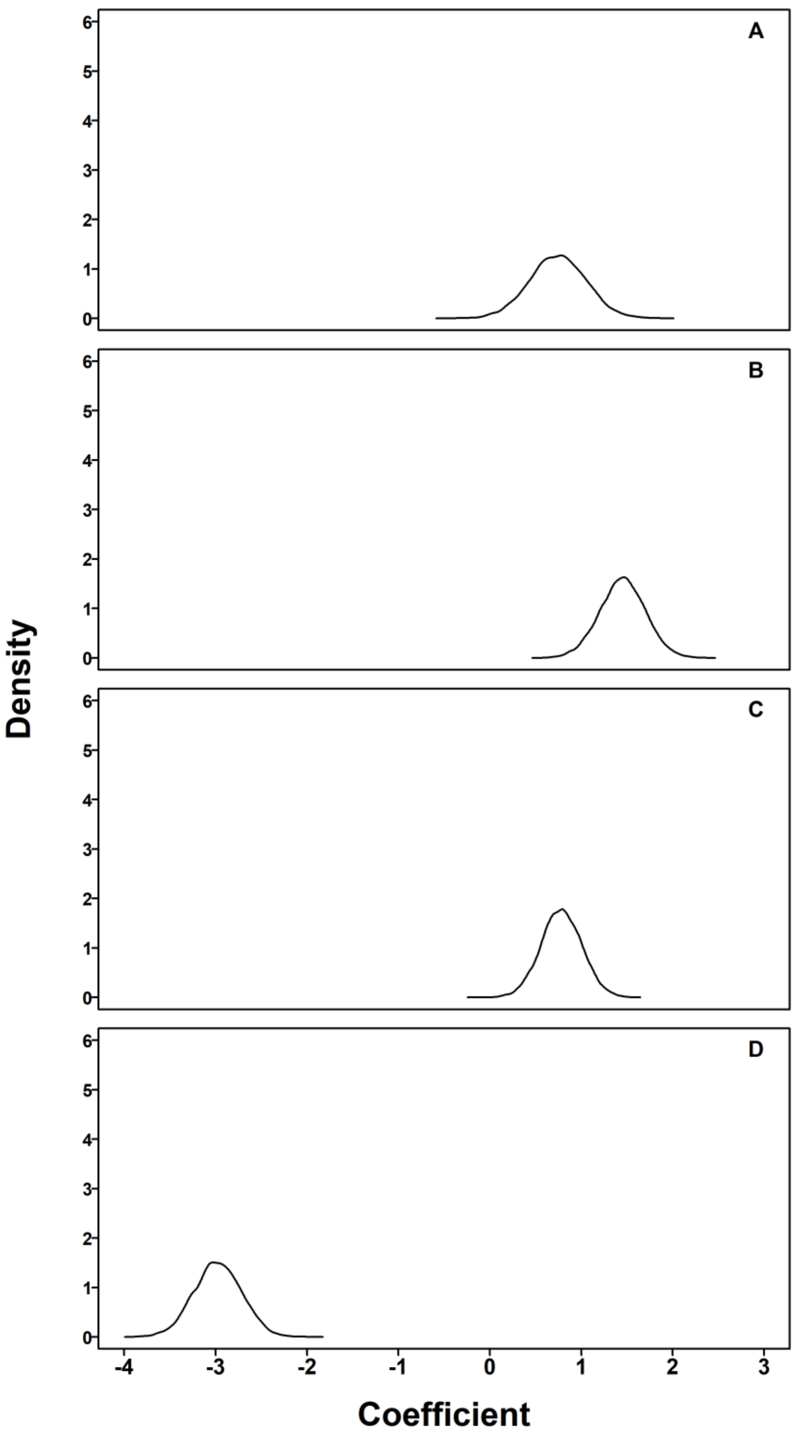

Supplementary Figure S49. Posterior densities of the ANOVA coefficients for the consumers' WTP for processed albacore tuna per age range (from A to D: 18–25, 26–40, 41–65, and 66+), extracted after 10^4 MCMC draws.

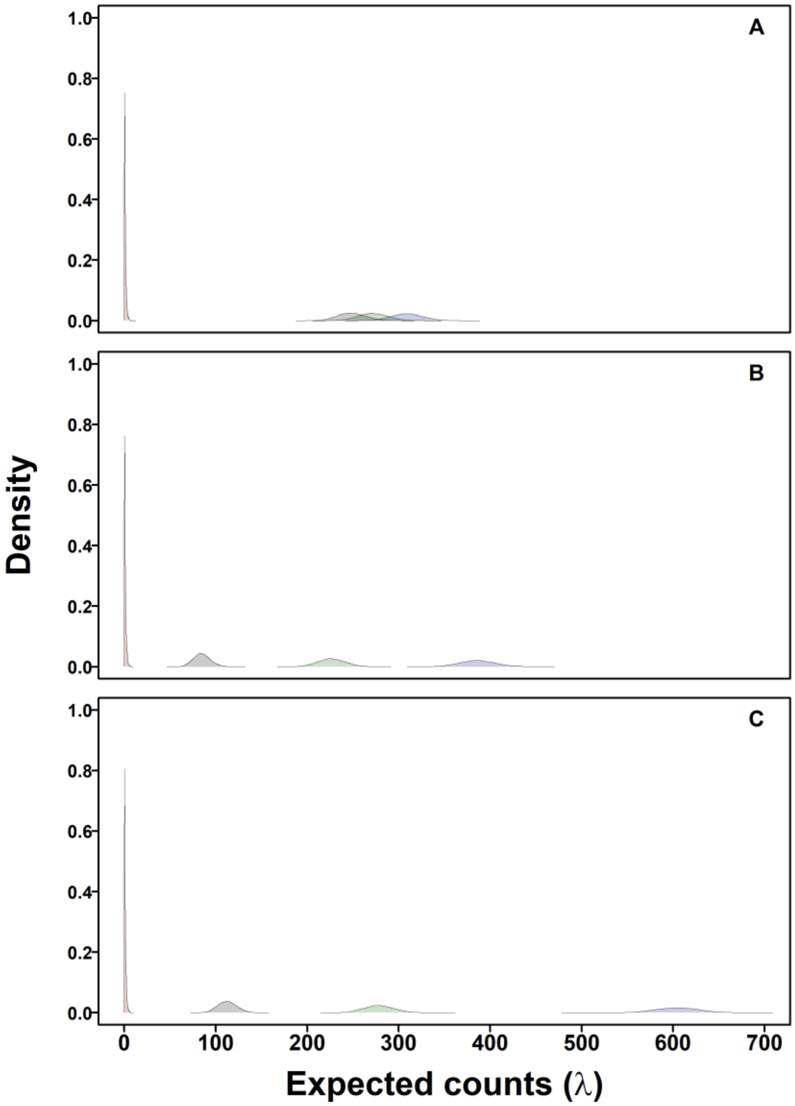

Supplementary Figure S50. Posterior densities of the expected counts (λ) for monthly seafood consumption rate (Red= 0, Blue= 1–5, Green= 6–10, and Dark gray= 10+) per province (from A to C: “South”, “Central”, and “North”), extracted after 10^4 MCMC draws.

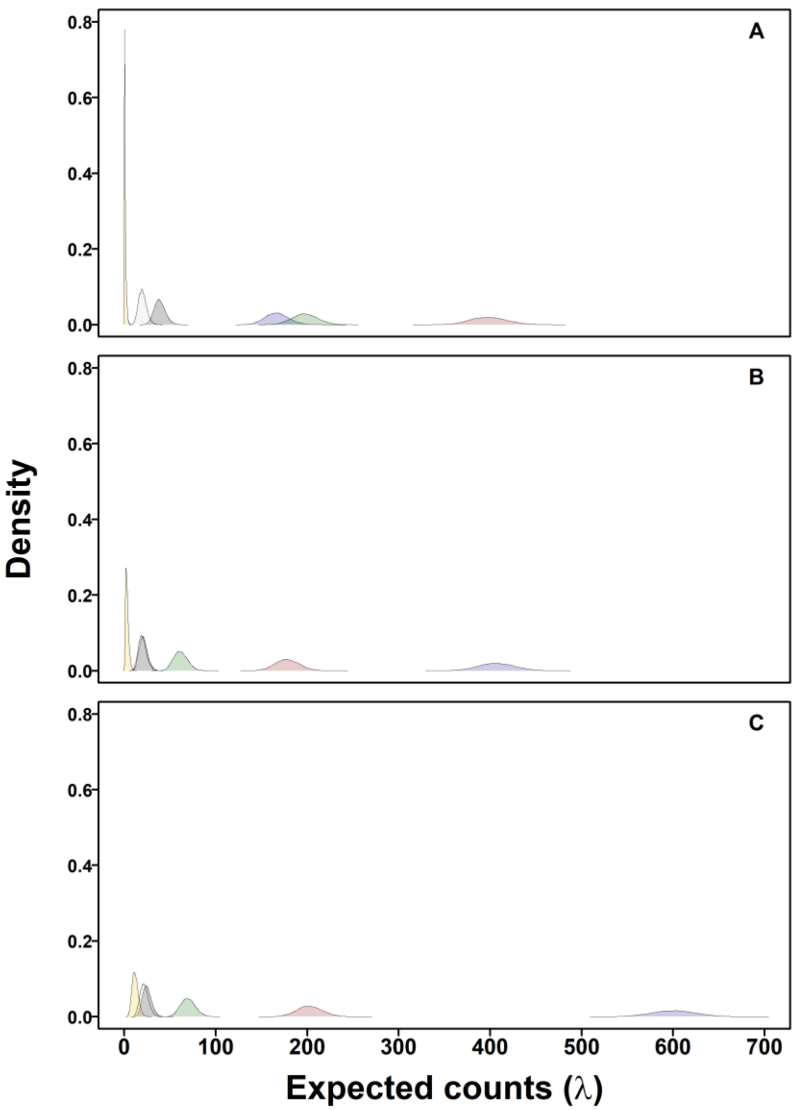

Supplementary Figure S51. Posterior densities of the expected counts (λ) for where consumers buy seafood (Red= "Fish market", Blue= "Large retail", Green= "Local market", Dark gray= "Not buy", Yellow= "Online", and Light gray = "Other") per province (from A to C: "South", "Central", and "North"), extracted after 10^4 MCMC draws.

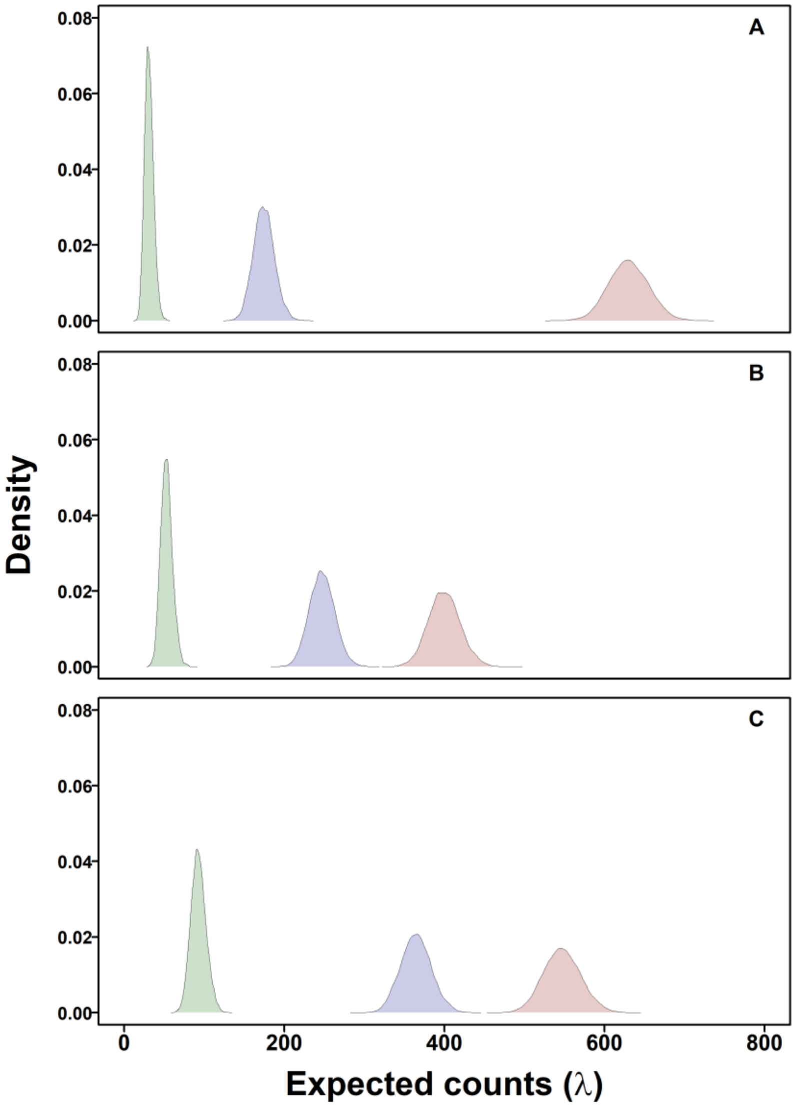

Supplementary Figure S52. Posterior densities of the expected counts (λ) for the degree of seafood processing consumers prefer (Red= "Fresh", Blue= "Frozen", and Green= "Processed") per province (from A to C: "South", "Central", and "North"), extracted after 10^4 MCMC draws.

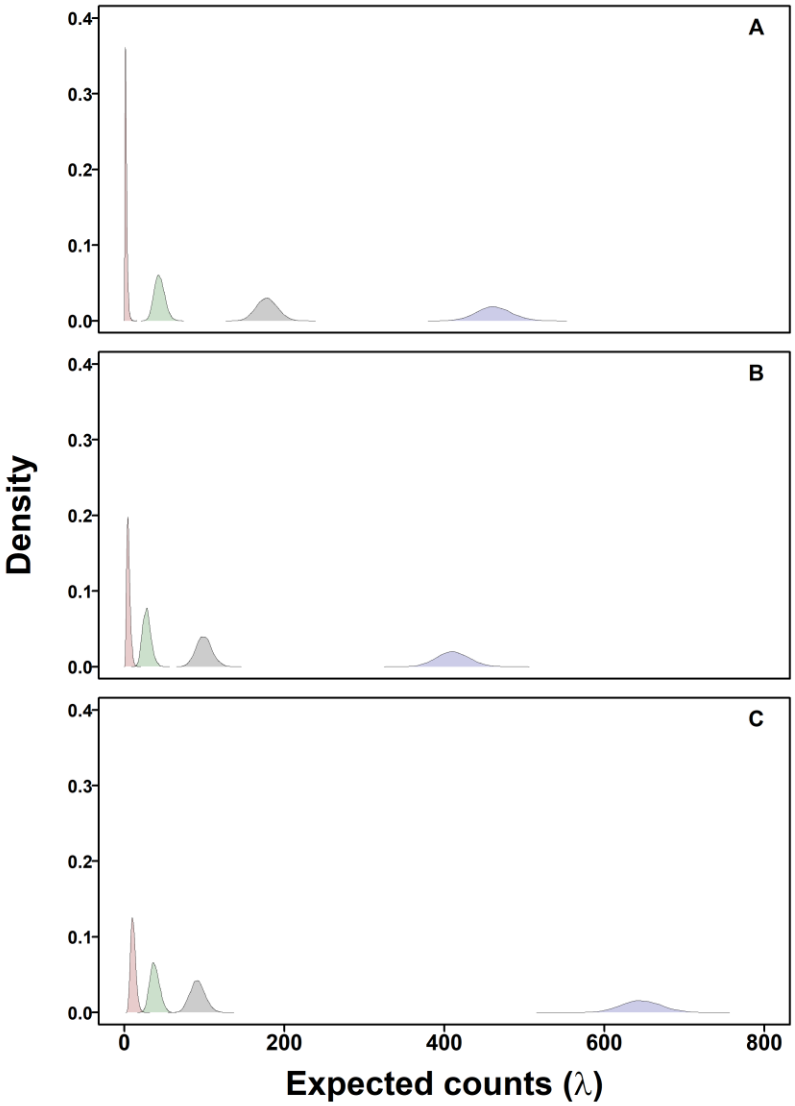

Supplementary Figure S53. Posterior densities of the expected counts (λ) for sources of information on seafood origin (Red= "Ads", Blue= "Label", Green= "Other", and Dark gray= "Retailer") per province (from A to C: "South", "Central", and "North"), extracted after 10^4 MCMC draws.

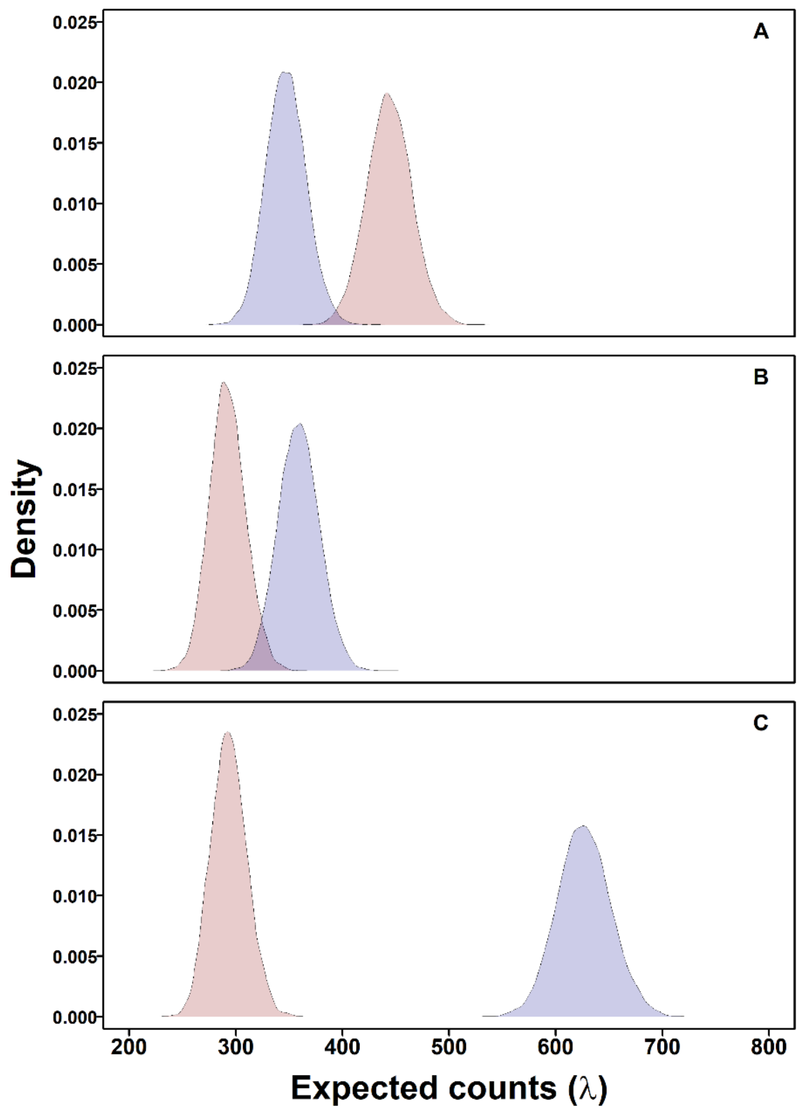

Supplementary Figure S54. Posterior densities of the expected counts (λ) for the consumption of Italian-farmed sea bass (Red= "Yes", and Blue= "No") per province (from A to C: "South", "Central", and "North"), extracted after 10^4 MCMC draws.

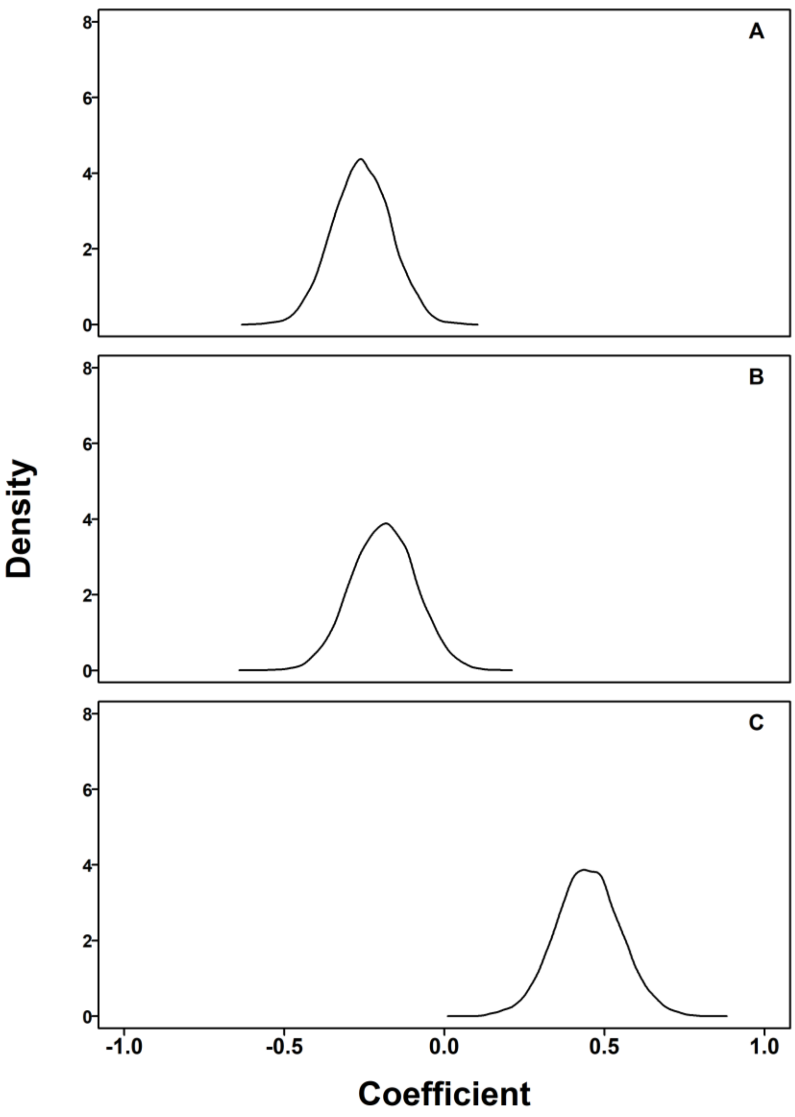

Supplementary Figure S55. Posterior densities of the ANOVA coefficients for the consumers' WTP for Italian-farmed sea bass per province (from A to C: "South", "Central", and "North"), extracted after 10^4 MCMC draws.

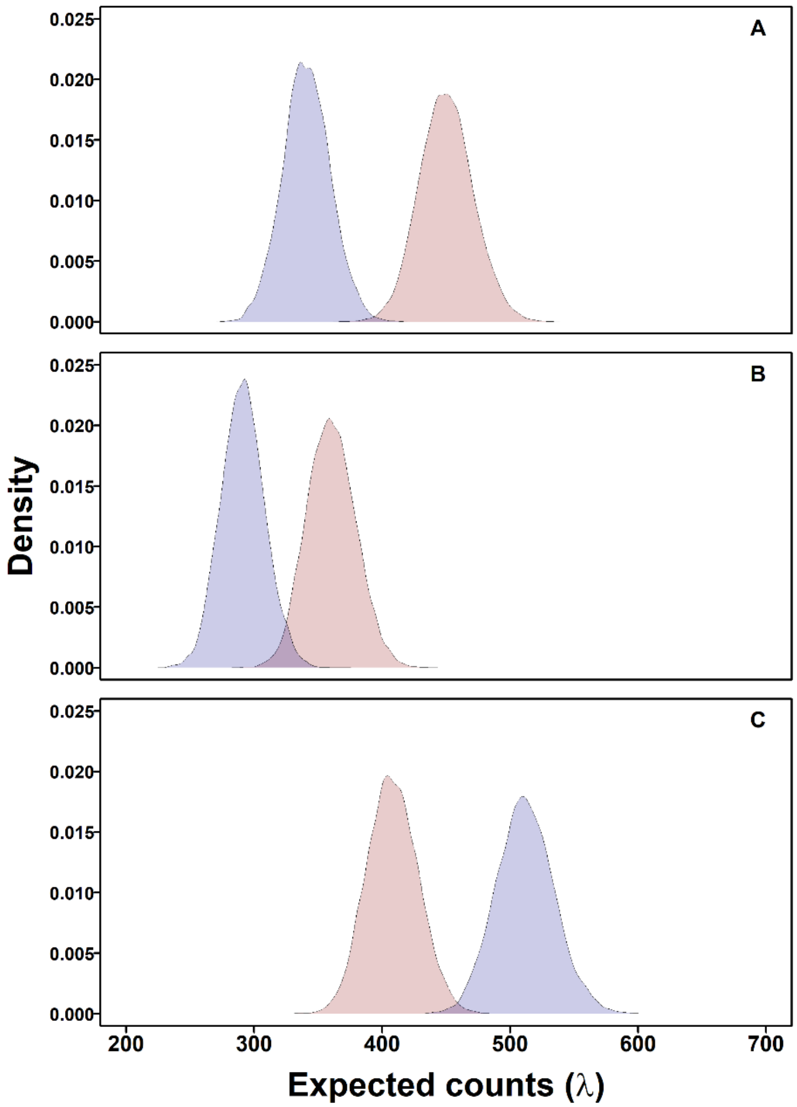

Supplementary Figure S56. Posterior densities of the expected counts (λ) for the consumption of striped venus clams (Red= "Yes", and Blue= "No") per province (from A to C: "South", "Central", and "North"), extracted after 10^4 MCMC draws.

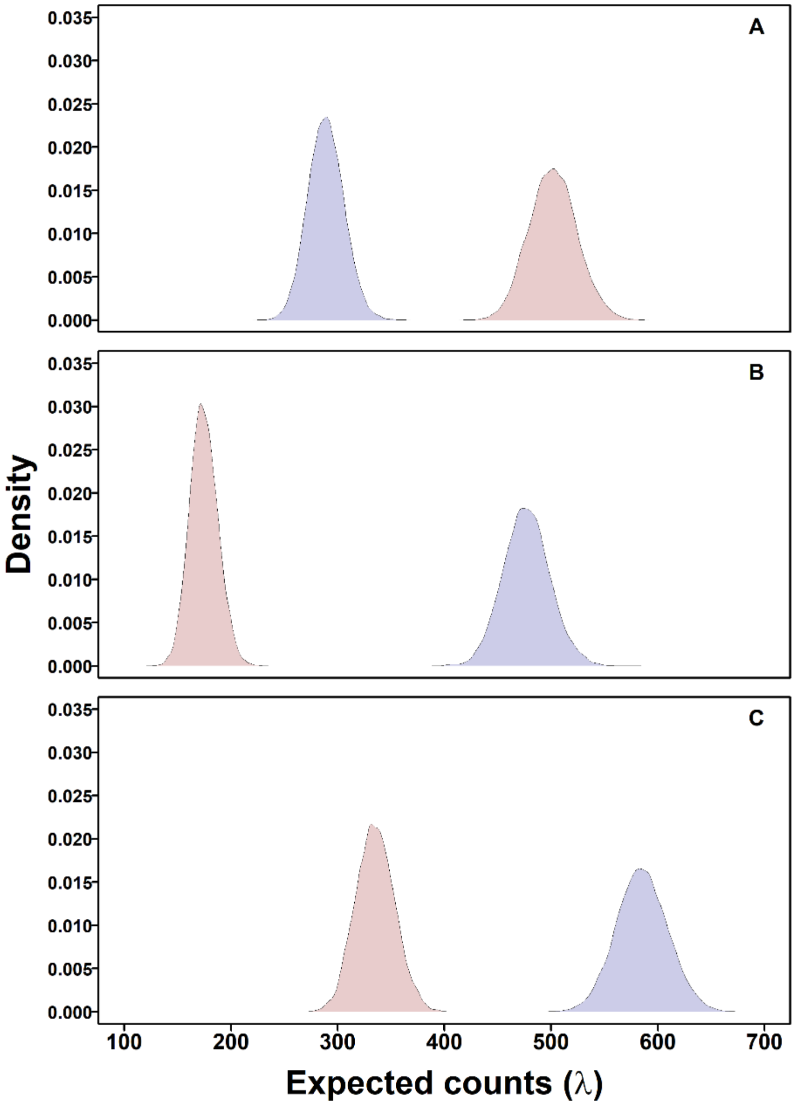

Supplementary Figure S57. Posterior densities of the expected counts (λ) for the consumption of giant red shrimp (Red= "Yes", and Blue= "No") per province (from A to C: "South", "Central", and "North"), extracted after 10^4 MCMC draws.

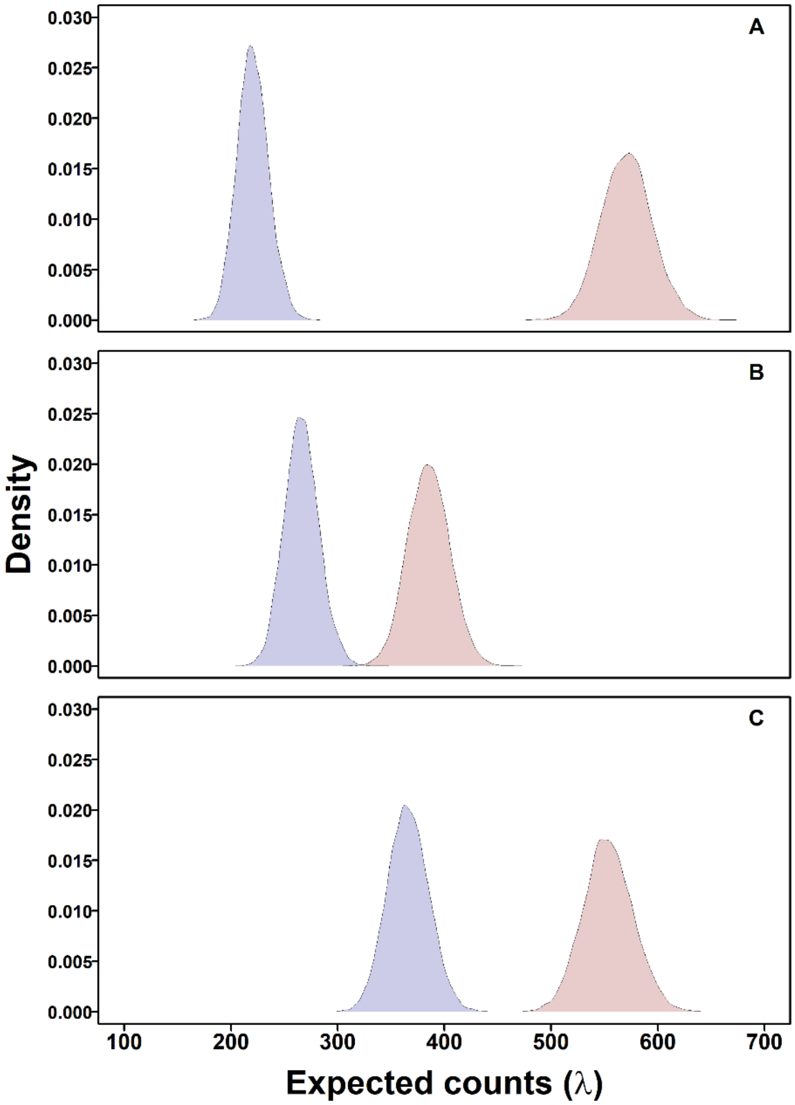

Supplementary Figure S58. Posterior densities of the expected counts (λ) for the consumption of processed albacore tuna (Red= "Yes", and Blue= "No") per province (from A to C: "South", "Central", and "North"), extracted after 10^4 MCMC draws.

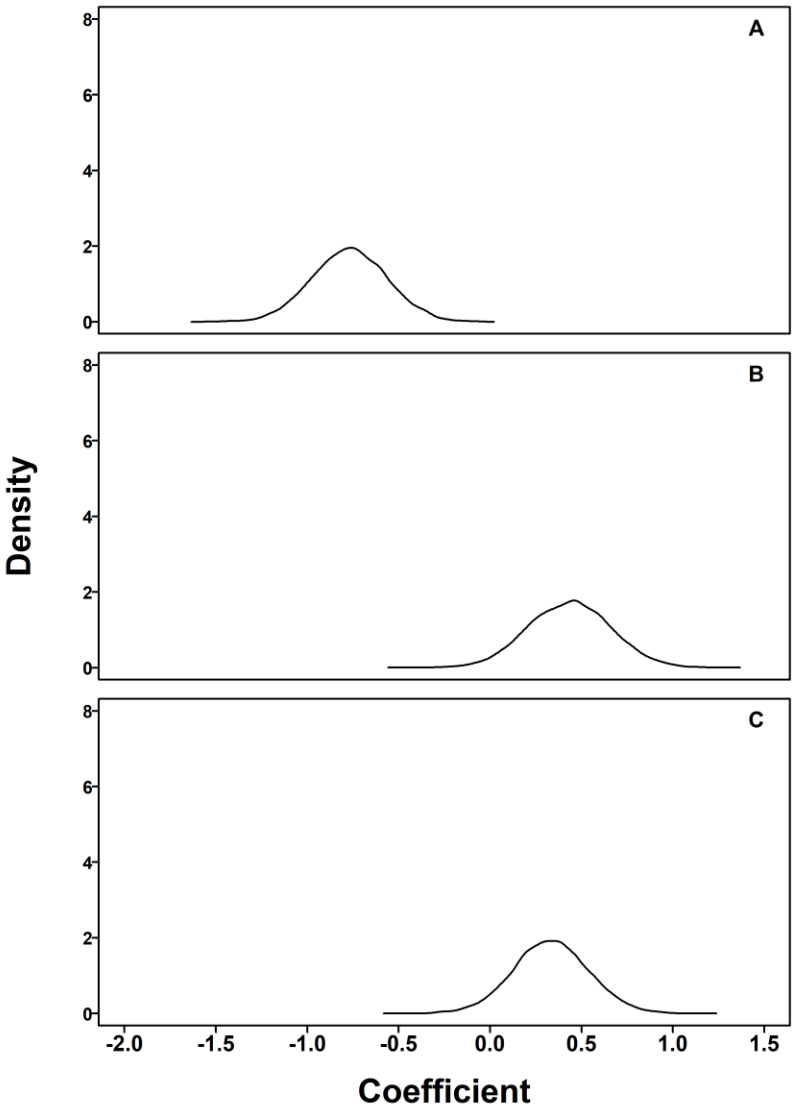

Supplementary Figure S59. Posterior densities of the ANOVA coefficients for the consumers' WTP for processed albacore tuna per province (from A to C: "South", "Central", and "North"), extracted after 10^4 MCMC draws.

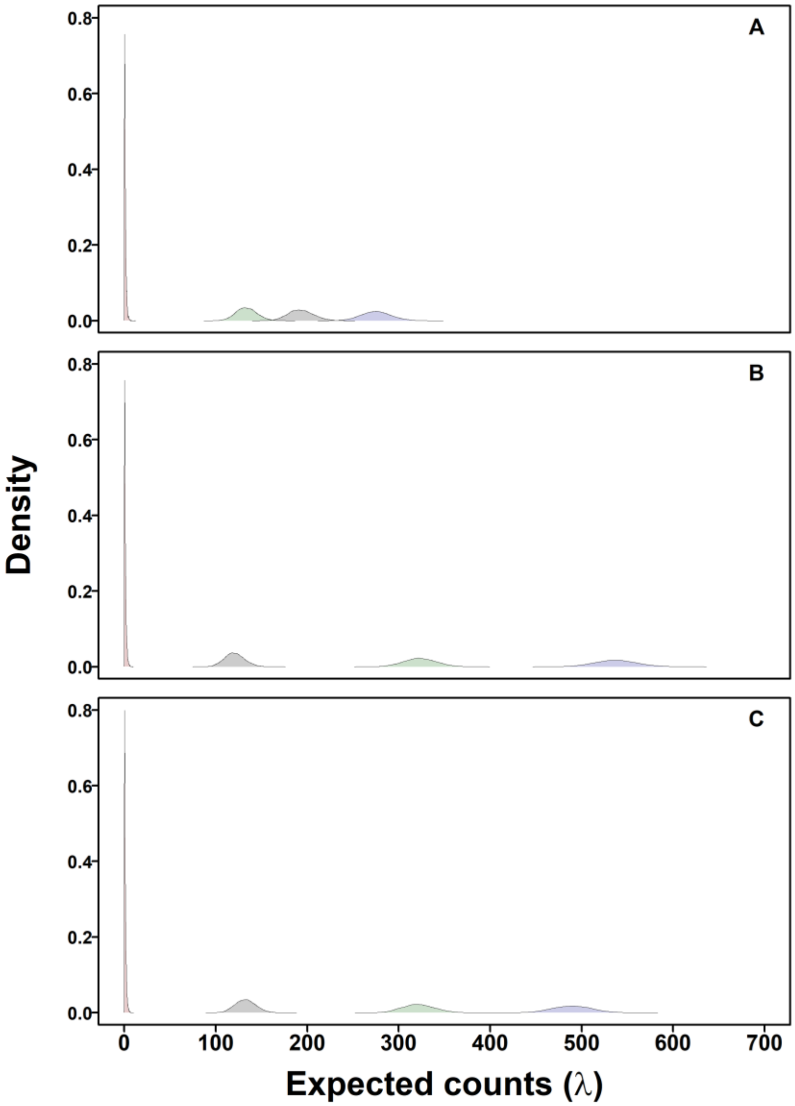

Supplementary Figure S60. Posterior densities of the expected counts (λ) for monthly seafood consumption rate (Red= 0, Blue= 1–5, Green= 6–10, and Dark gray= 10+) per educational level (from A to C: “Middle school degree”, “High school degree”, and “BSc degree or higher”), extracted after 10^4 MCMC draws.

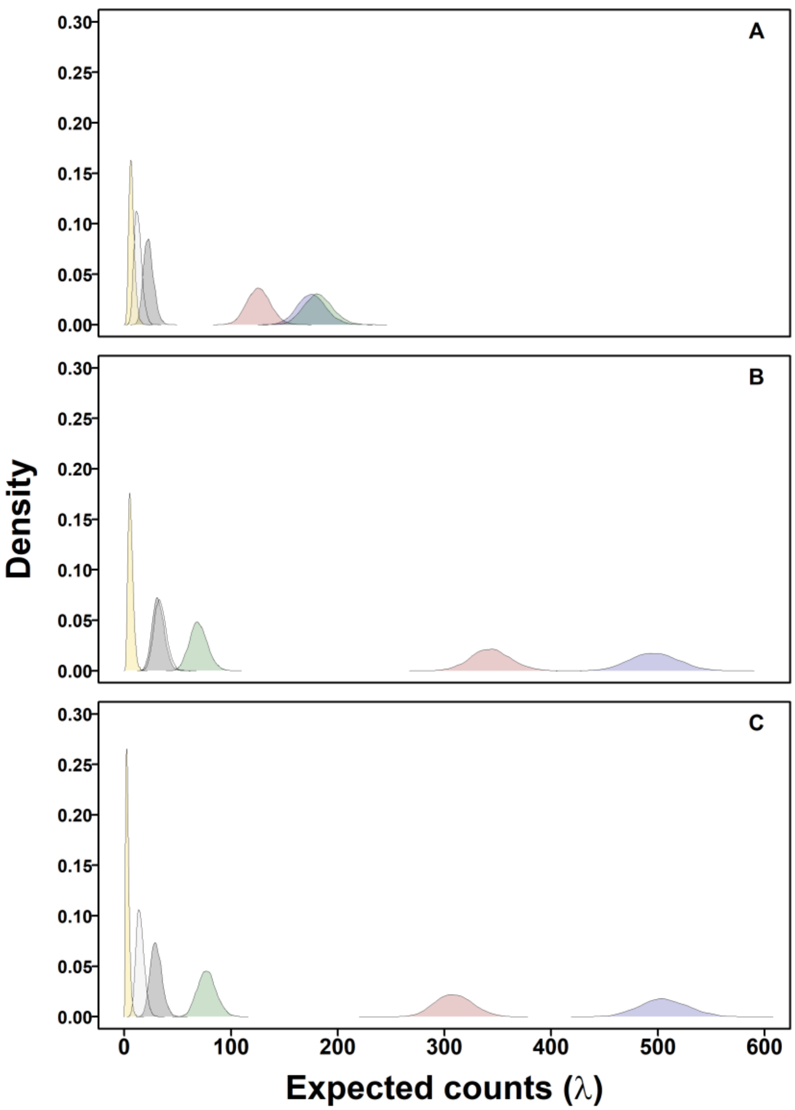

Supplementary Figure S61. Posterior densities of the expected counts (λ) for where consumers buy seafood (Red= "Fish market", Blue= "Large retail", Green= "Local market", Dark gray= "Not buy", Yellow= "Online", and Light gray = "Other") per educational level (from A to C: "Middle school degree", "High school degree", and "BSc degree or higher"), extracted after 10^4 MCMC draws.

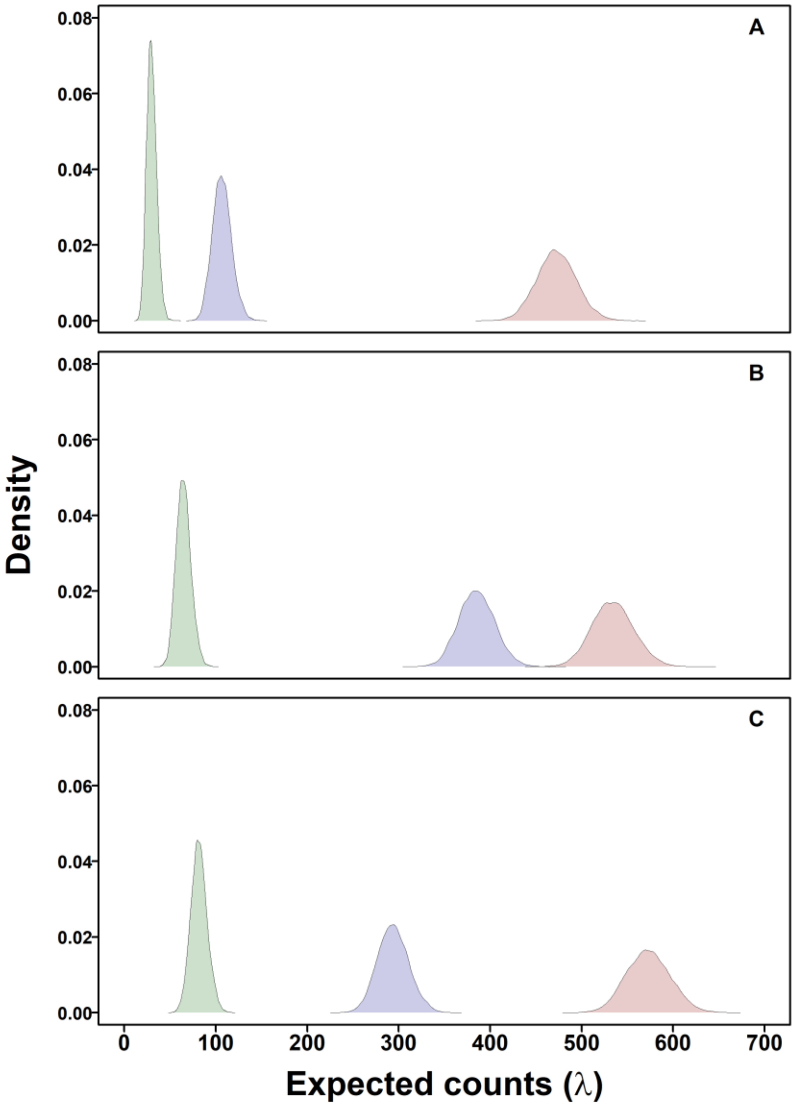

Supplementary Figure S62. Posterior densities of the expected counts (λ) for the degree of seafood processing consumers prefer (Red= "Fresh", Blue= "Frozen", and Green= "Processed") per educational level (from A to C: "Middle school degree", "High school degree", and "BSc degree or higher"), extracted after 10^4 MCMC draws.

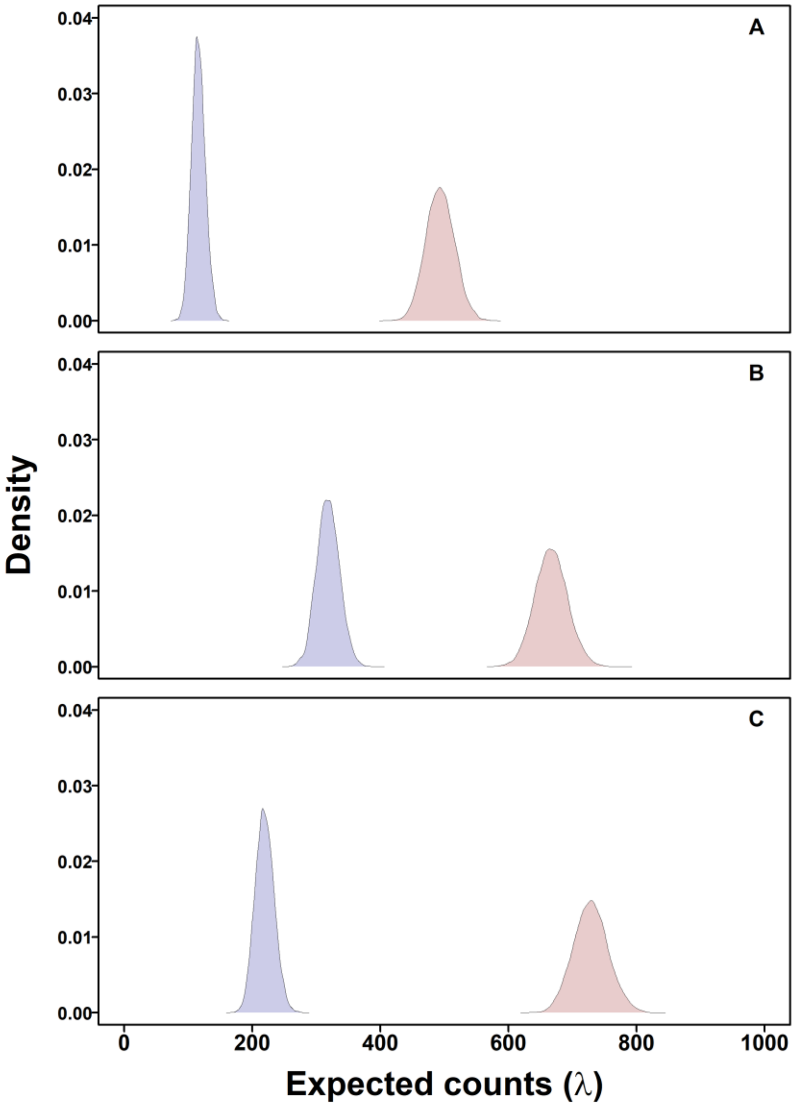

Supplementary Figure S63. Posterior densities of the expected counts (λ) for being informed on seafood origin (Red= "Yes" and Blue= "No") per educational level (from A to C: "Middle school degree", "High school degree", and "BSc degree or higher"), extracted after 10^4 MCMC draws.

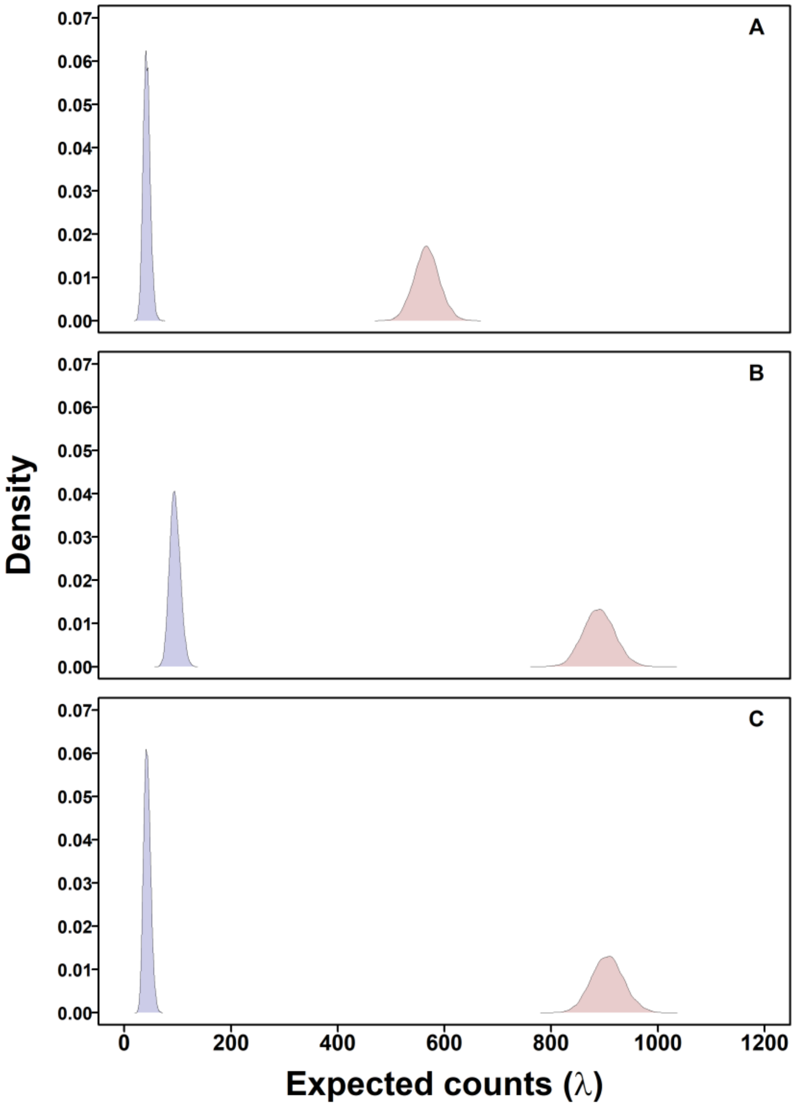

Supplementary Figure S64. Posterior densities of the expected counts (λ) for interest on seafood traceability (Red= “Yes” and Blue= “No”) per educational level (from A to C: “Middle school degree”, “High school degree”, and “BSc degree or higher”), extracted after 10^4 MCMC draws.

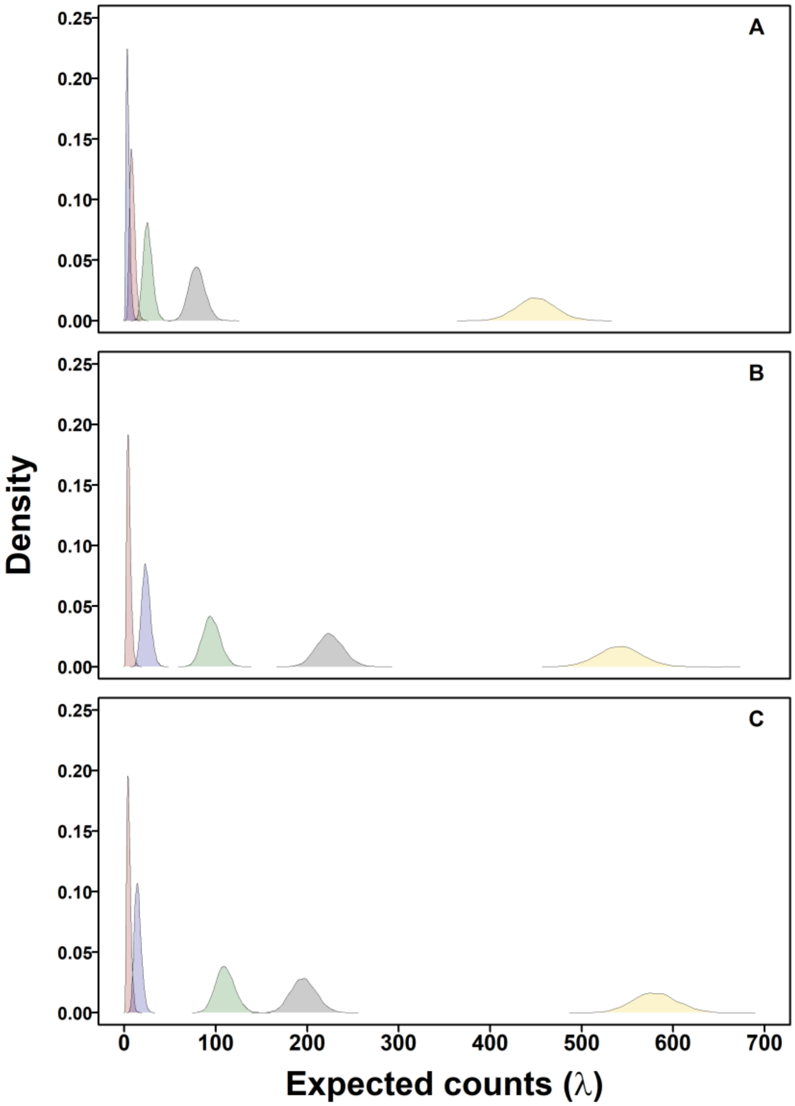

Supplementary Figure S65. Posterior densities of the expected counts (λ) for level of consumers' interest in seafood traceability (Red= 1, Blue= 2, Green= 3, Dark gray= 4, and Yellow= 5) per educational level (from A to C: "Middle school degree", "High school degree", and "BSc degree or higher"), extracted after 10^4 MCMC draws.

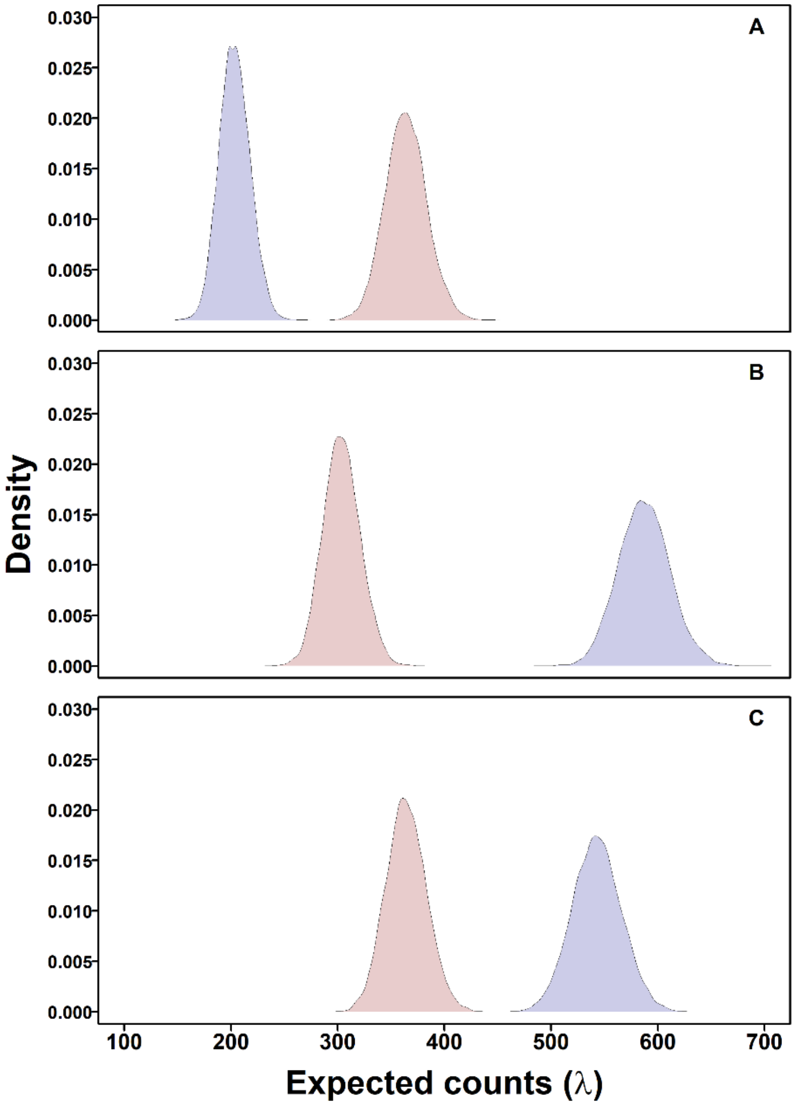

Supplementary Figure S66. Posterior densities of the expected counts (λ) for the consumption of Italian-farmed sea bass (Red= "Yes" and Blue= "No") per educational level (from A to C: "Middle school degree", "High school degree", and "BSc degree or higher"), extracted after 10^4 MCMC draws.

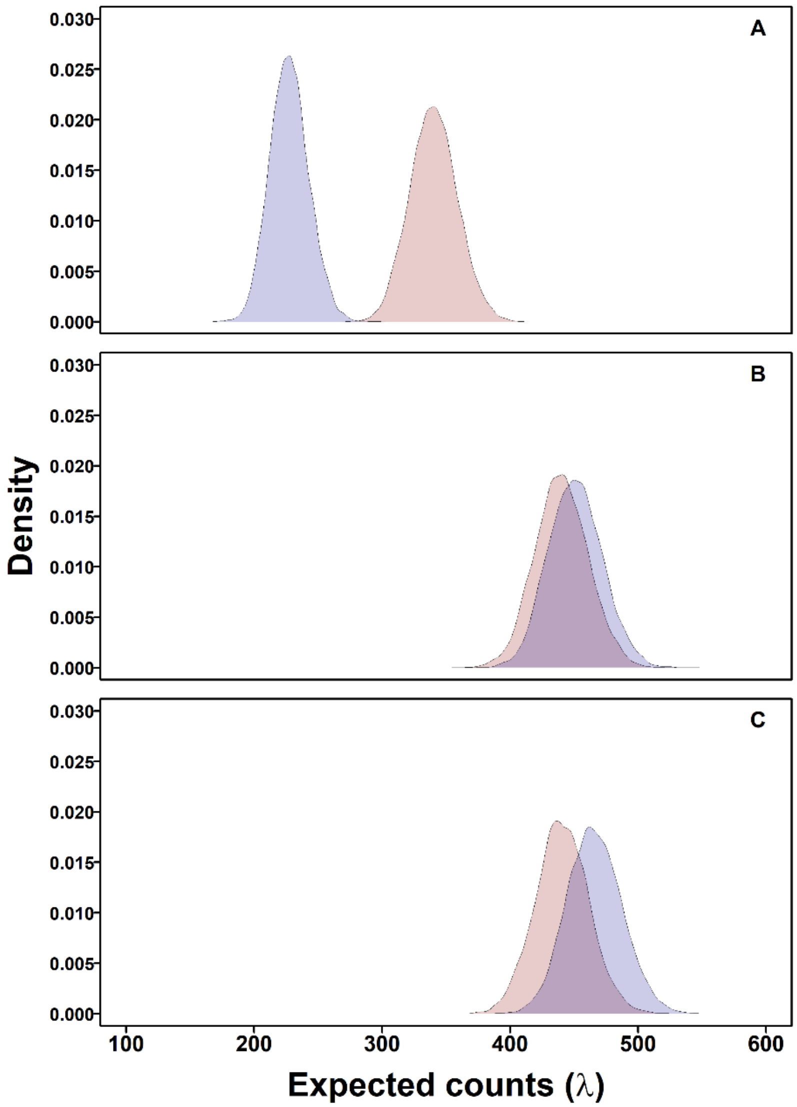

Supplementary Figure S67. Posterior densities of the expected counts (λ) for the consumption of striped venus clams (Red= "Yes" and Blue= "No") per educational level (from A to C: "Middle school degree", "High school degree", and "BSc degree or higher"), extracted after 10^4 MCMC draws.

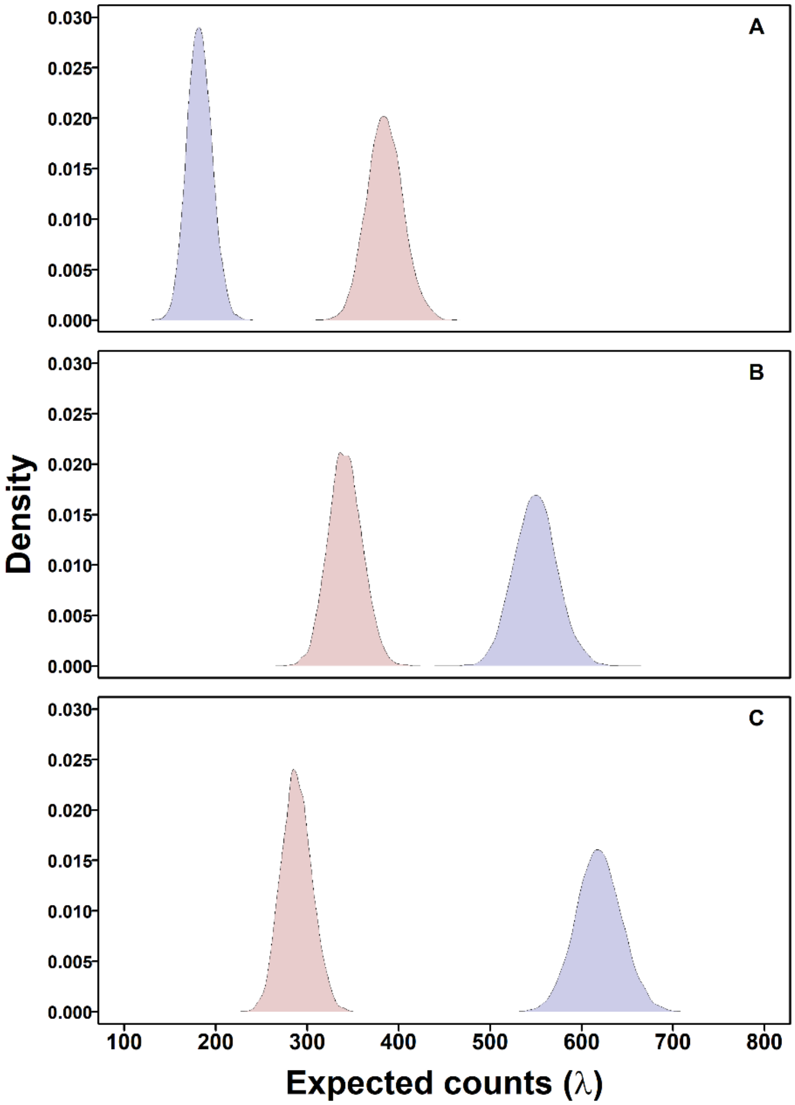

Supplementary Figure S68. Posterior densities of the expected counts (λ) for the consumption of red giant shrimp (Red= "Yes" and Blue= "No") per educational level (from A to C: "Middle school degree", "High school degree", and "BSc degree or higher"), extracted after 10^4 MCMC draws.

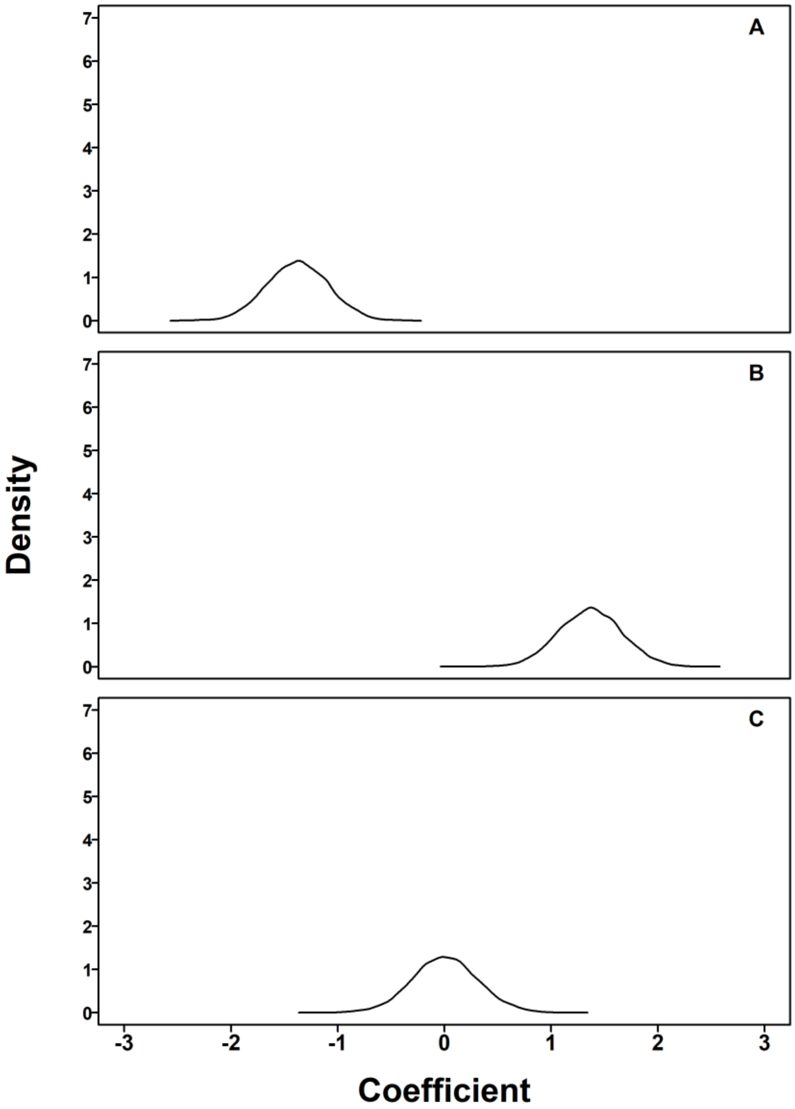

Supplementary Figure S69. Posterior densities of the ANOVA coefficients for the consumers' WTP for red giant shrimp per educational level (from A to C: "Middle school degree", "High school degree", and "BSc degree or higher"), extracted after 10^4 MCMC draws.

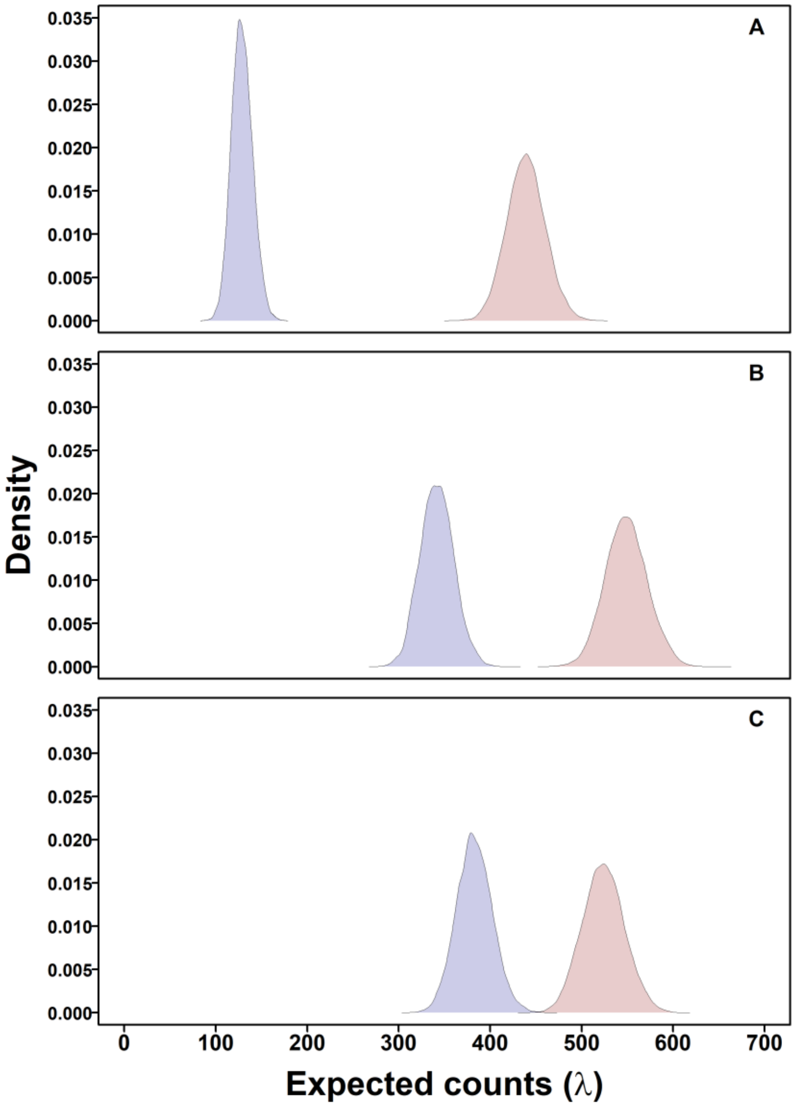

Supplementary Figure S70. Posterior densities of the expected counts (λ) for the consumption of processed albacore tuna (Red= "Yes" and Blue= "No") per educational level (from A to C: "Middle school degree", "High school degree", and "BSc degree or higher"), extracted after 10^4 MCMC draws.

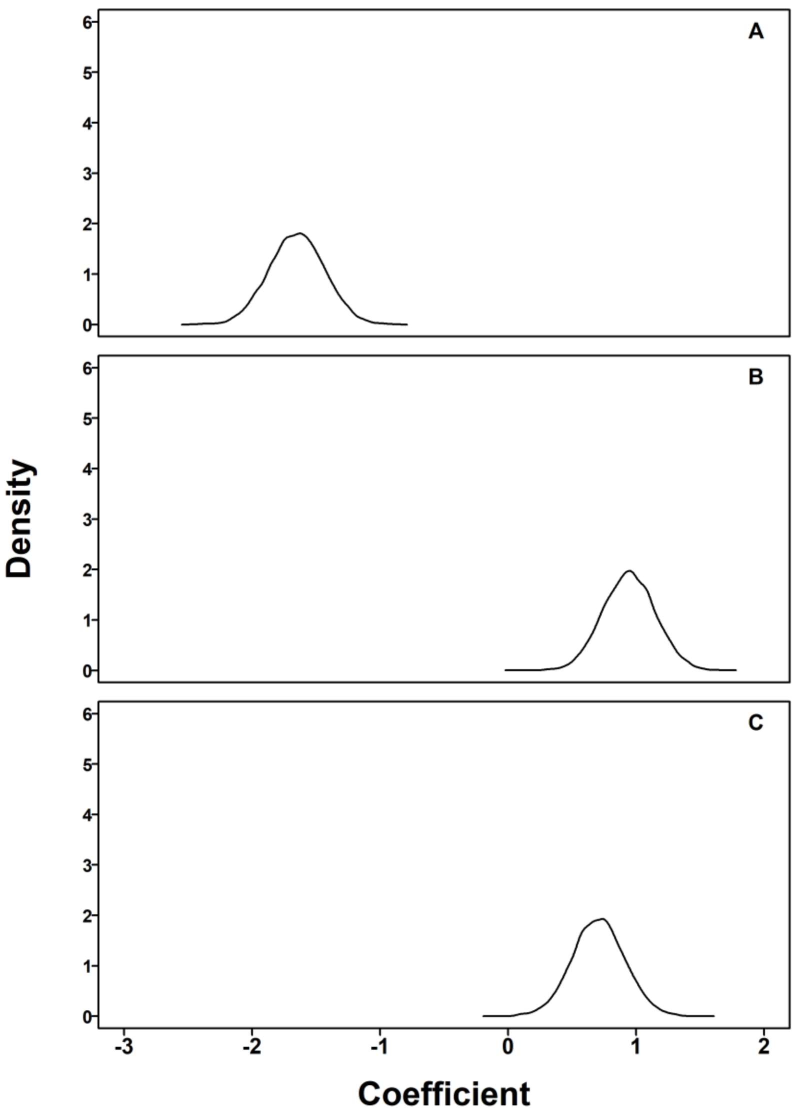

Supplementary Figure S71. Posterior densities of the ANOVA coefficients for the consumers' WTP for processed albacore tuna per educational level (from A to C: "Middle school degree", "High school degree", and "BSc degree or higher"), extracted after 10^4 MCMC draws.

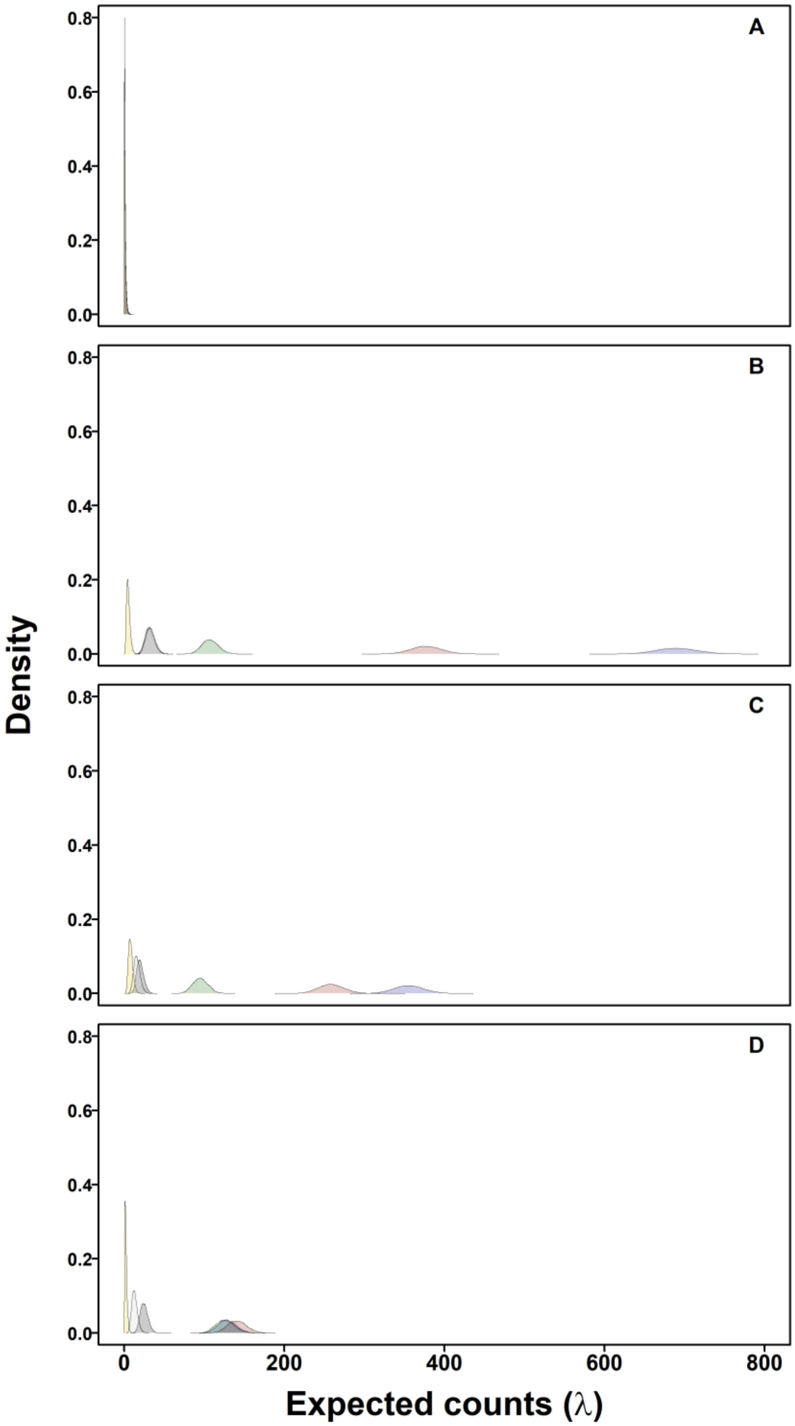

Supplementary Figure S72. Posterior densities of the expected counts (λ) for where consumers buy seafood (Red= "Fish market", Blue= "Large retail", Green= "Local market", Dark gray= "Not buy", Yellow= "Online", and Light gray = "Other") per monthly seafood consumption rate (from A to D: 0, 1–5, 6–10, and 10+), extracted after 10^4 MCMC draws.

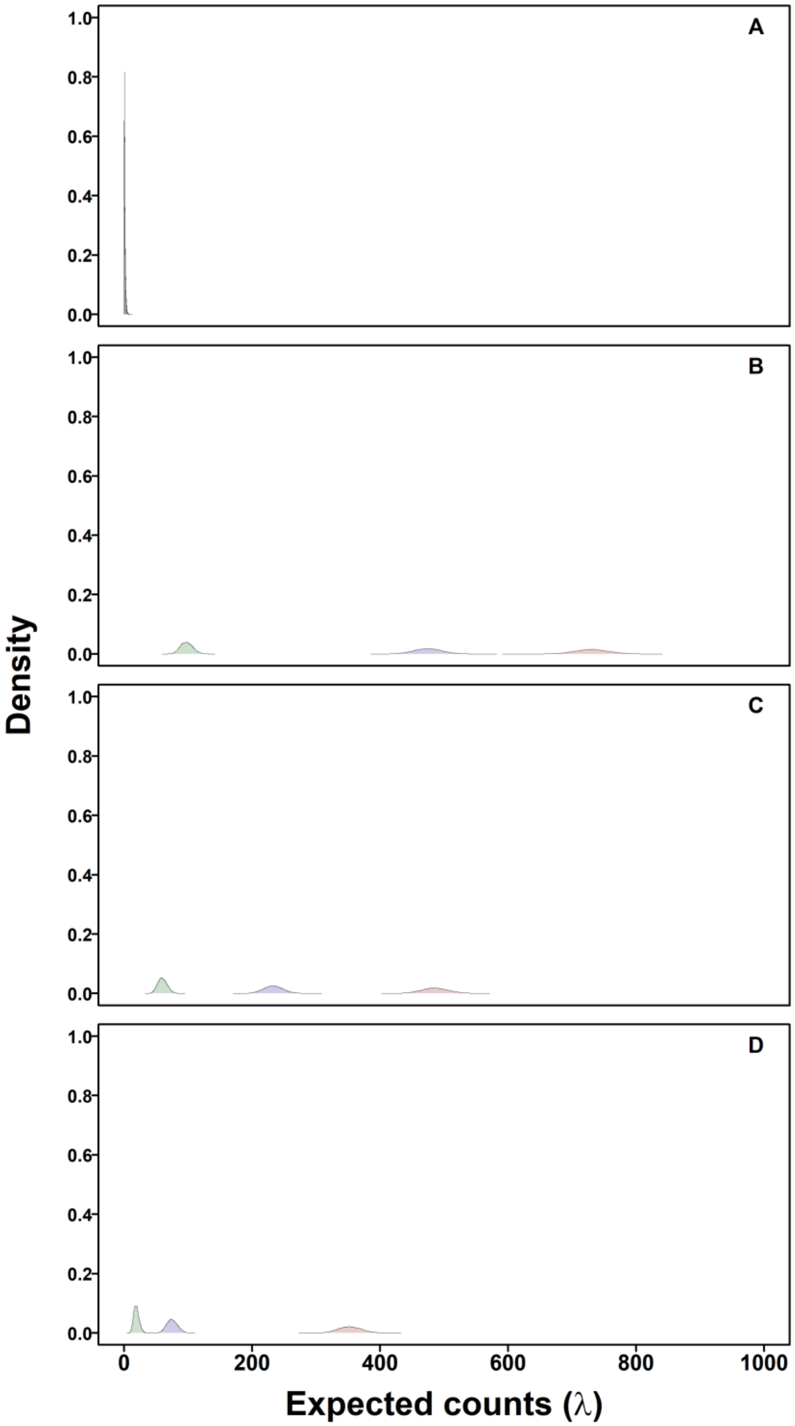

Supplementary Figure S73. Posterior densities of the expected counts (λ) for the degree of seafood processing consumers prefer (Red= "Fresh", Blue= "Frozen", and Green= "Processed") per monthly seafood consumption rate (from A to D: 0, 1–5, 6–10, and 10+), extracted after 10^4 MCMC draws.

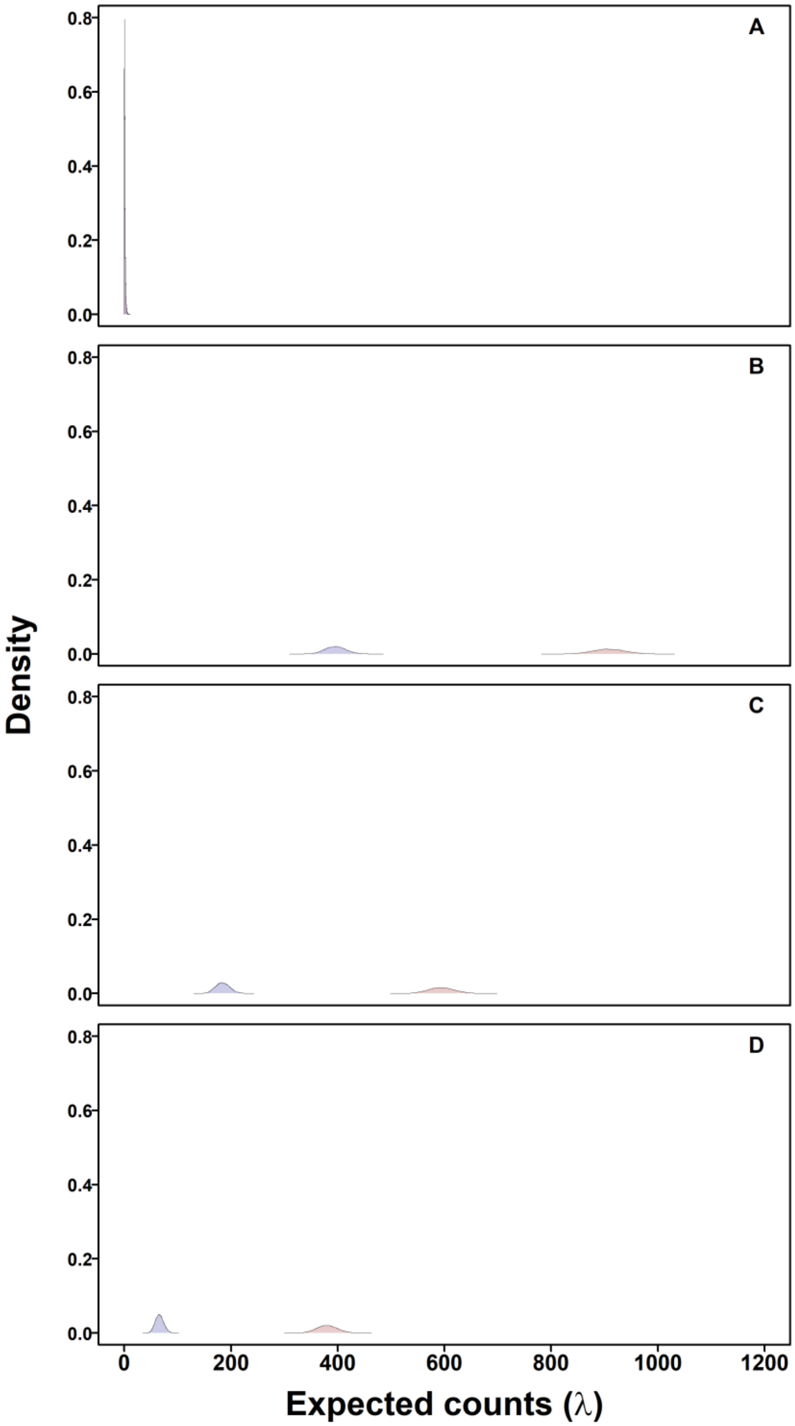

Supplementary Figure S74. Posterior densities of the expected counts (λ) for being informed on seafood origin (Red= "Yes" and Blue= "No") per monthly seafood consumption rate (from A to D: 0, 1–5, 6–10, and 10+), extracted after 10^4 MCMC draws.

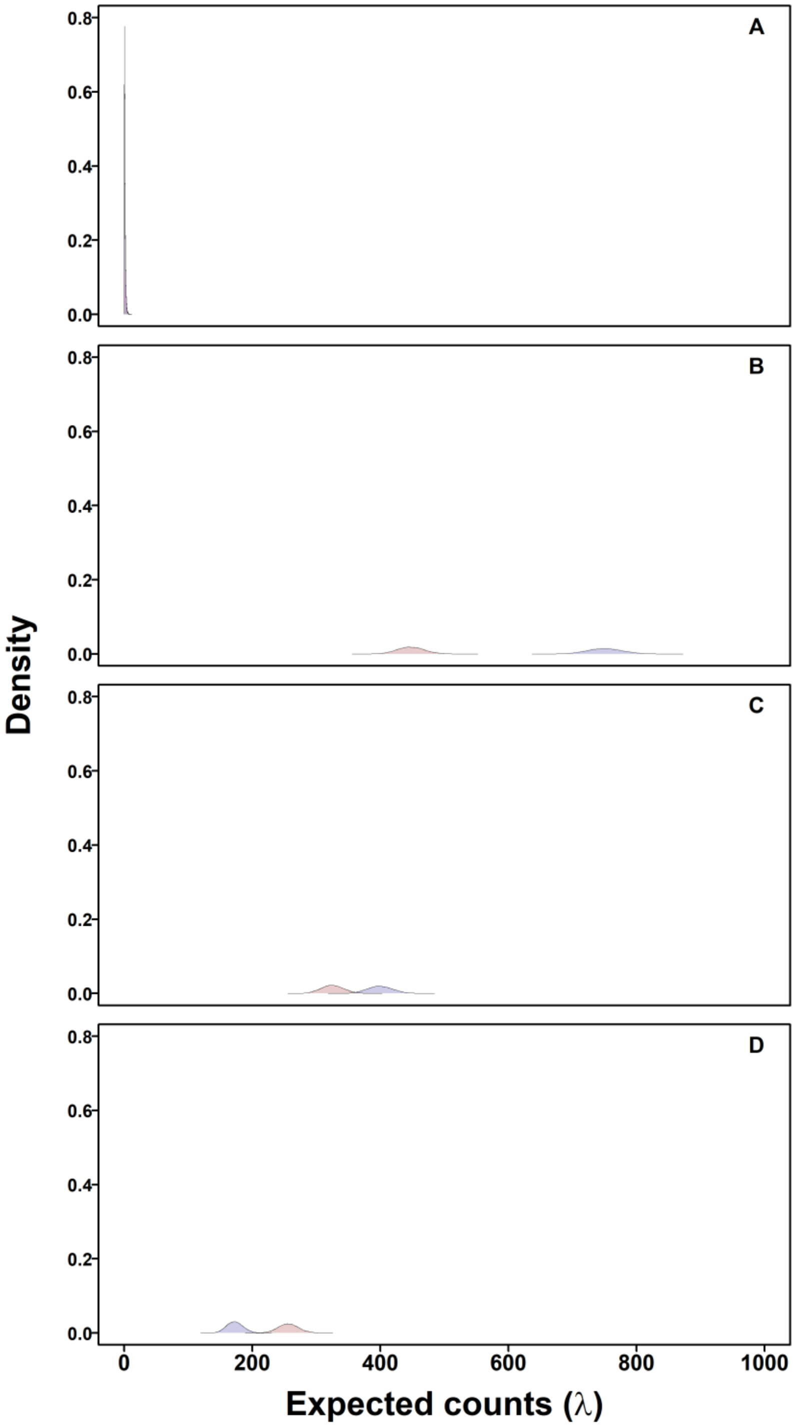

Supplementary Figure S75. Posterior densities of the expected counts (λ) for the consumption of Italian-farmed sea bass (Red= "Yes" and Blue= "No") per monthly seafood consumption rate (from A to D: 0, 1–5, 6–10, and 10+), extracted after 10^4 MCMC draws.

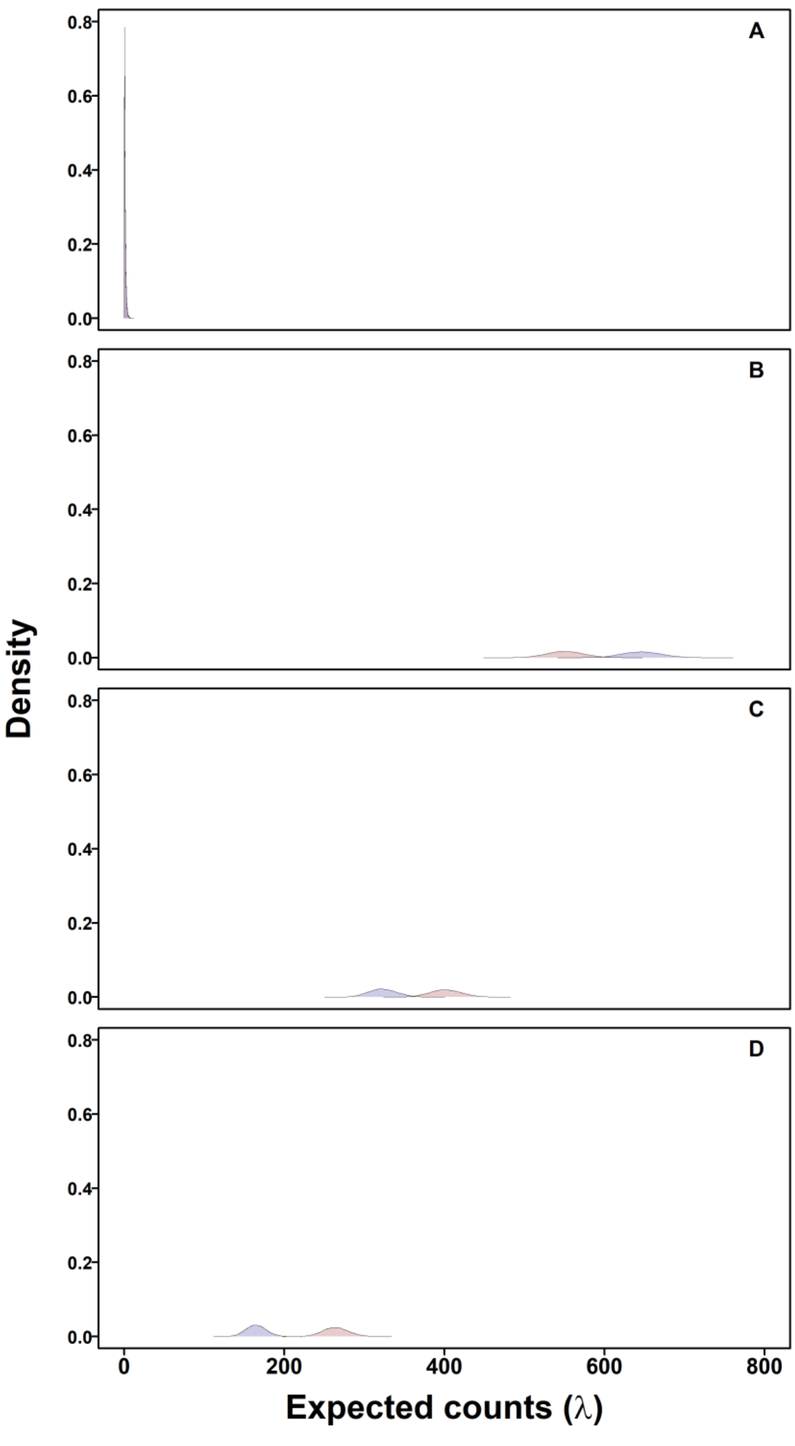

Supplementary Figure S76. Posterior densities of the expected counts (λ) for the consumption of striped venus clams (Red= “Yes” and Blue= “No”) per monthly seafood consumption rate (from A to D: 0, 1–5, 6–10, and 10+), extracted after 10^4 MCMC draws.

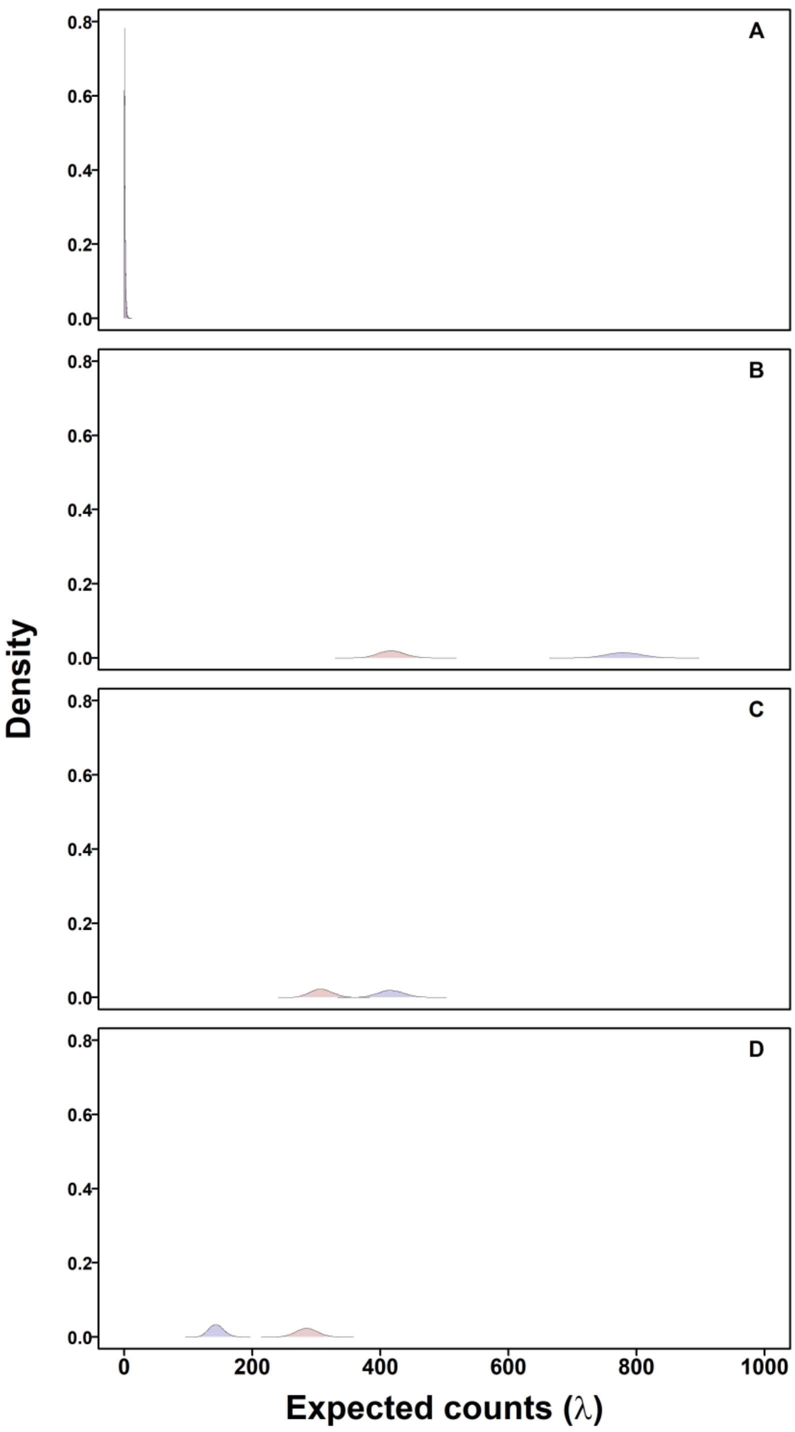

Supplementary Figure S77. Posterior densities of the expected counts (λ) for the consumption of giant red shrimp (Red= "Yes" and Blue= "No") per monthly seafood consumption rate (from A to D: 0, 1–5, 6–10, and 10+), extracted after 10^4 MCMC draws.

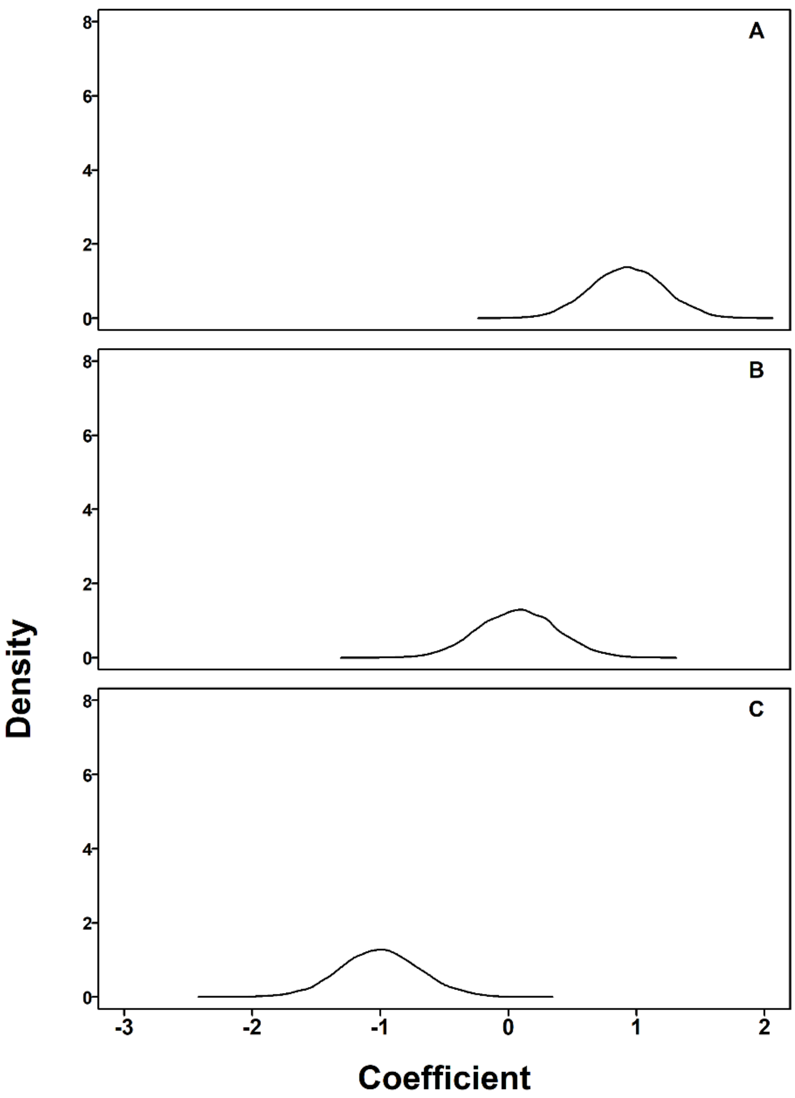

Supplementary Figure S78. Posterior densities of the ANOVA coefficients for the consumers' WTP for giant red shrimp per monthly seafood consumption rate (from A to C: 1–5, 6–10, and 10+), extracted after 10^4 MCMC draws. Note the absence of data for 0 monthly consumption.

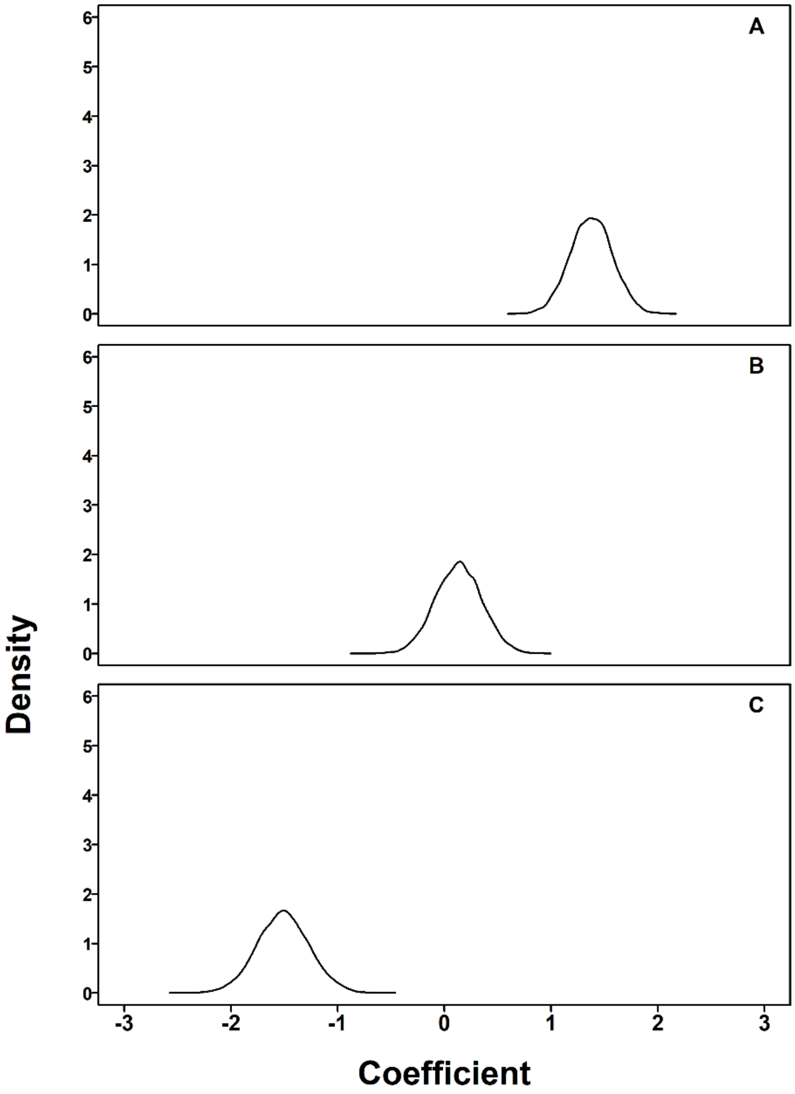

Supplementary Figure S79. Posterior densities of the ANOVA coefficients for the consumers' WTP for processed albacore tuna per monthly seafood consumption rate (from A to C: 1–5, 6–10, and 10+), extracted after 10^4 MCMC draws. Note the absence of data for 0 monthly consumption.

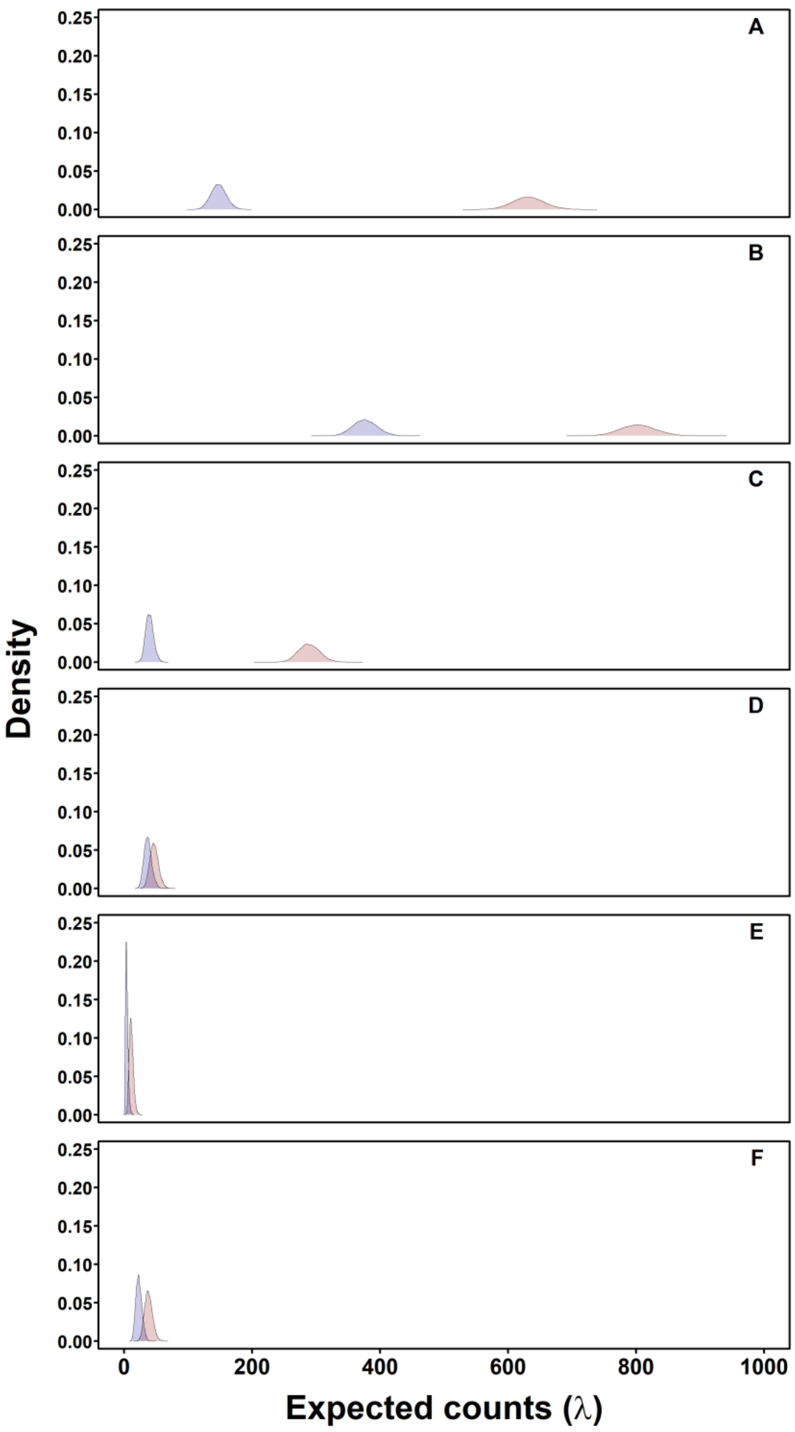

Supplementary Figure S80. Posterior densities of the expected counts (λ) for being informed on seafood origin (Red= "Yes" and Blue= "No") per where consumers buy seafood (from A to F: "Fish market", "Large retail", "Local market", "Not buy", "Online", and "Other"), extracted after 10^4 MCMC draws.

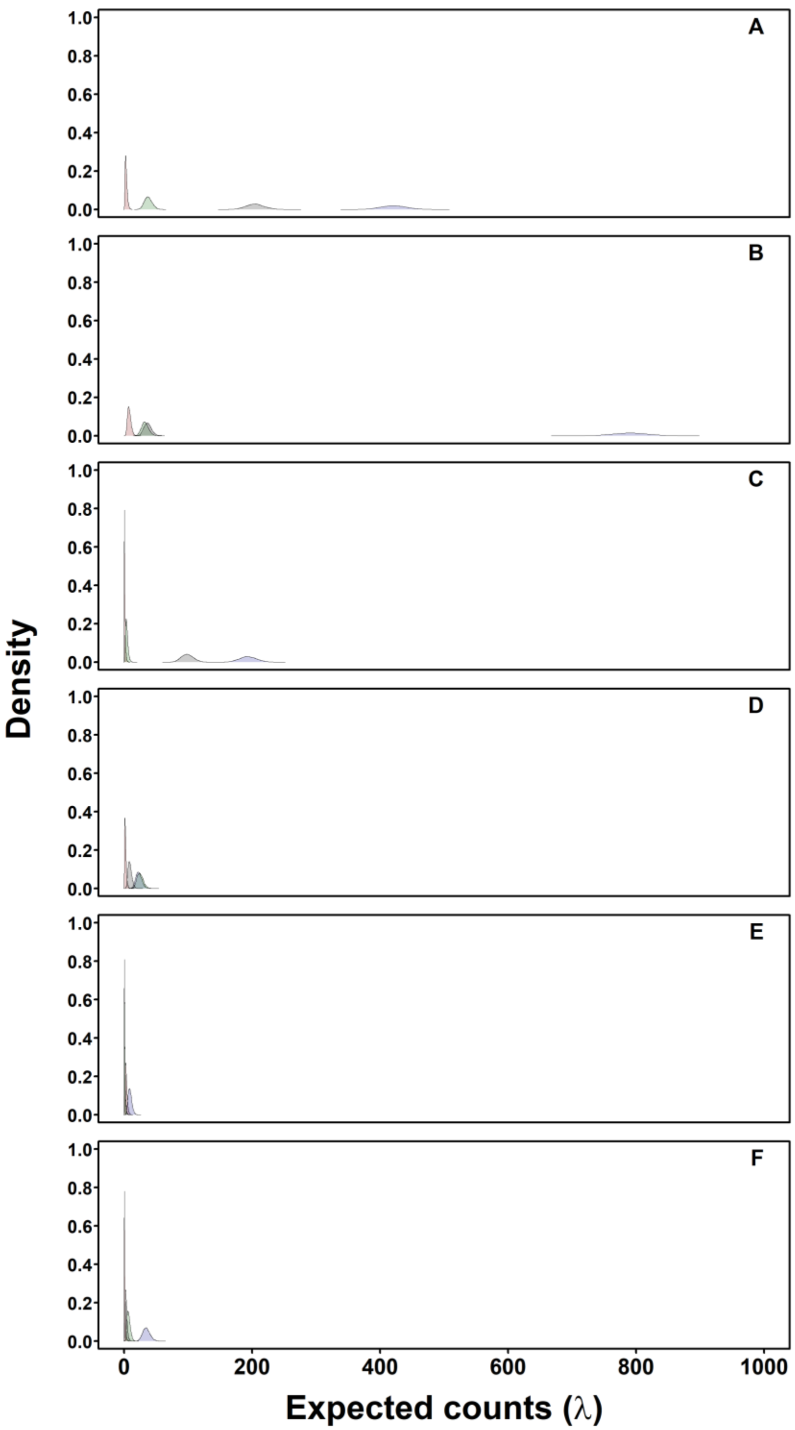

Supplementary Figure S81. Posterior densities of the expected counts (λ) for sources of information on seafood origin (Red= “Ads”, Blue= “Label”, Green= “Other”, and Dark gray= “Retailer”) per where consumers buy seafood (from A to F: “Fish market”, “Large retail”, “Local market”, “Not buy”, “Online”, and “Other”), extracted after 10^4 MCMC draws.

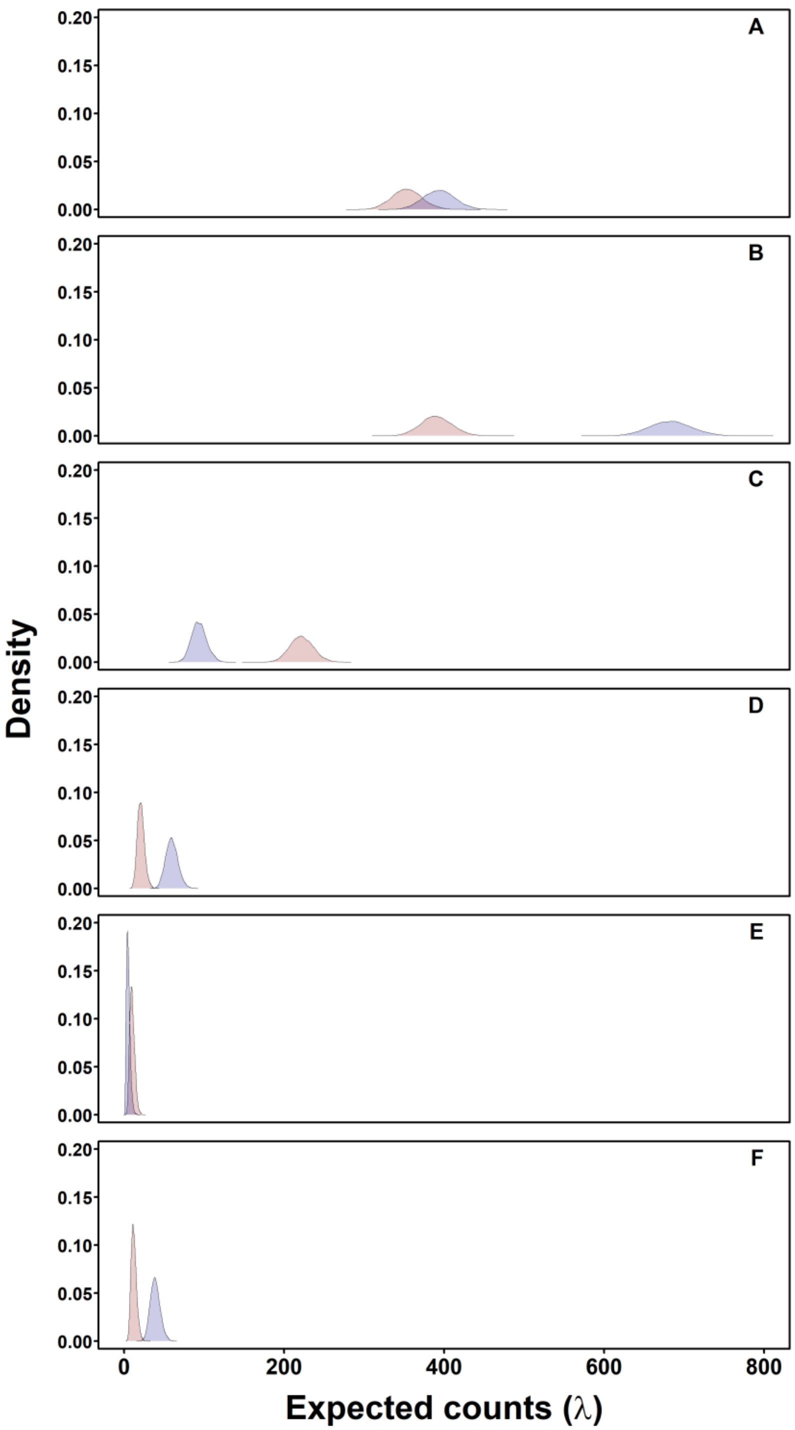

Supplementary Figure S82. Posterior densities of the expected counts (λ) for the consumption of Italian-farmed sea bass (Red= "Yes" and Blue= "No") per where consumers buy seafood (from A to F: "Fish market", "Large retail", "Local market", "Not buy", "Online", and "Other"), extracted after 10^4 MCMC draws.

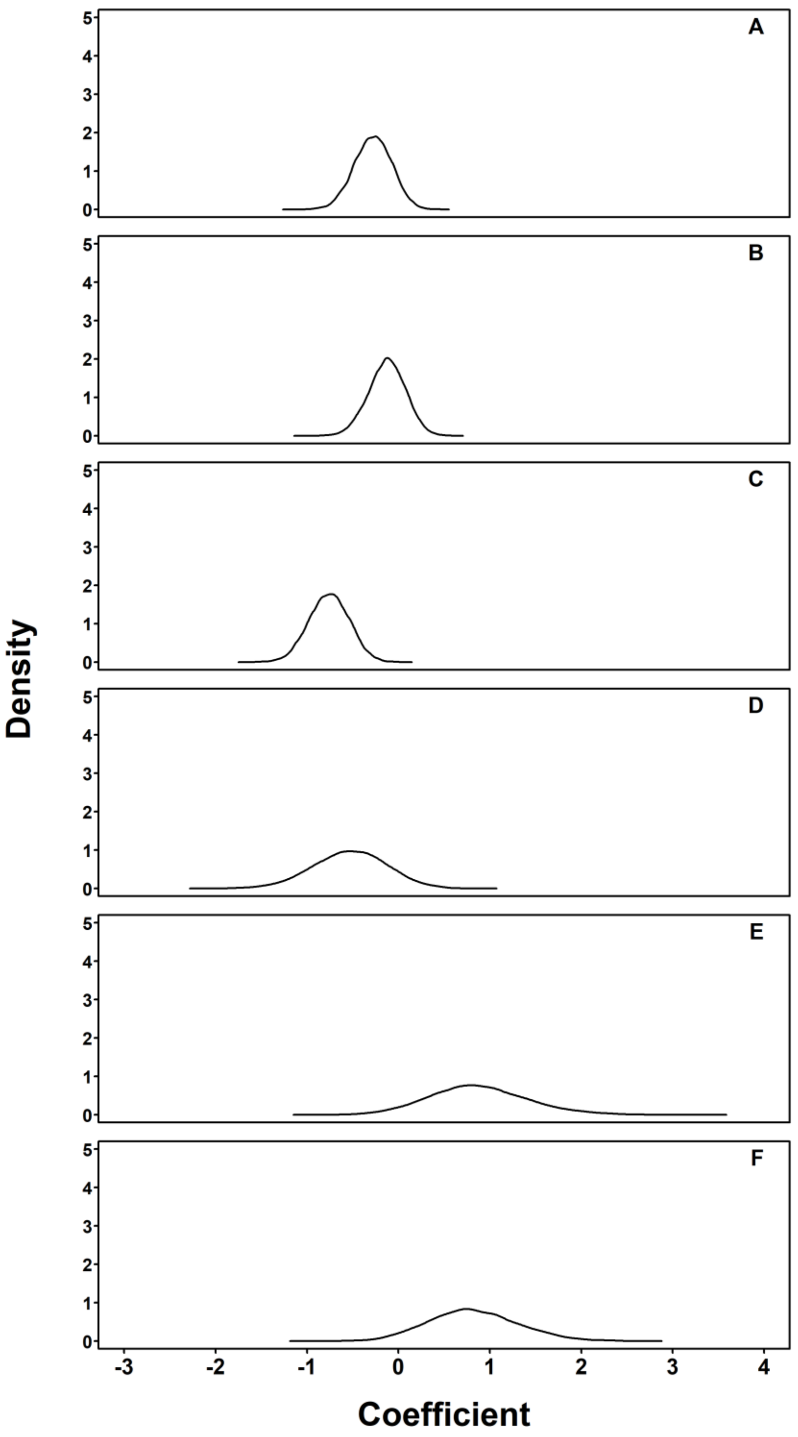

Supplementary Figure S83. Posterior densities of the ANOVA coefficients for the consumers' WTP for Italian-farmed sea bass per where consumers buy seafood (from A to F: "Fish market", "Large retail", "Local market", "Not buy", "Online", and "Other"), extracted after 10^4 MCMC draws.

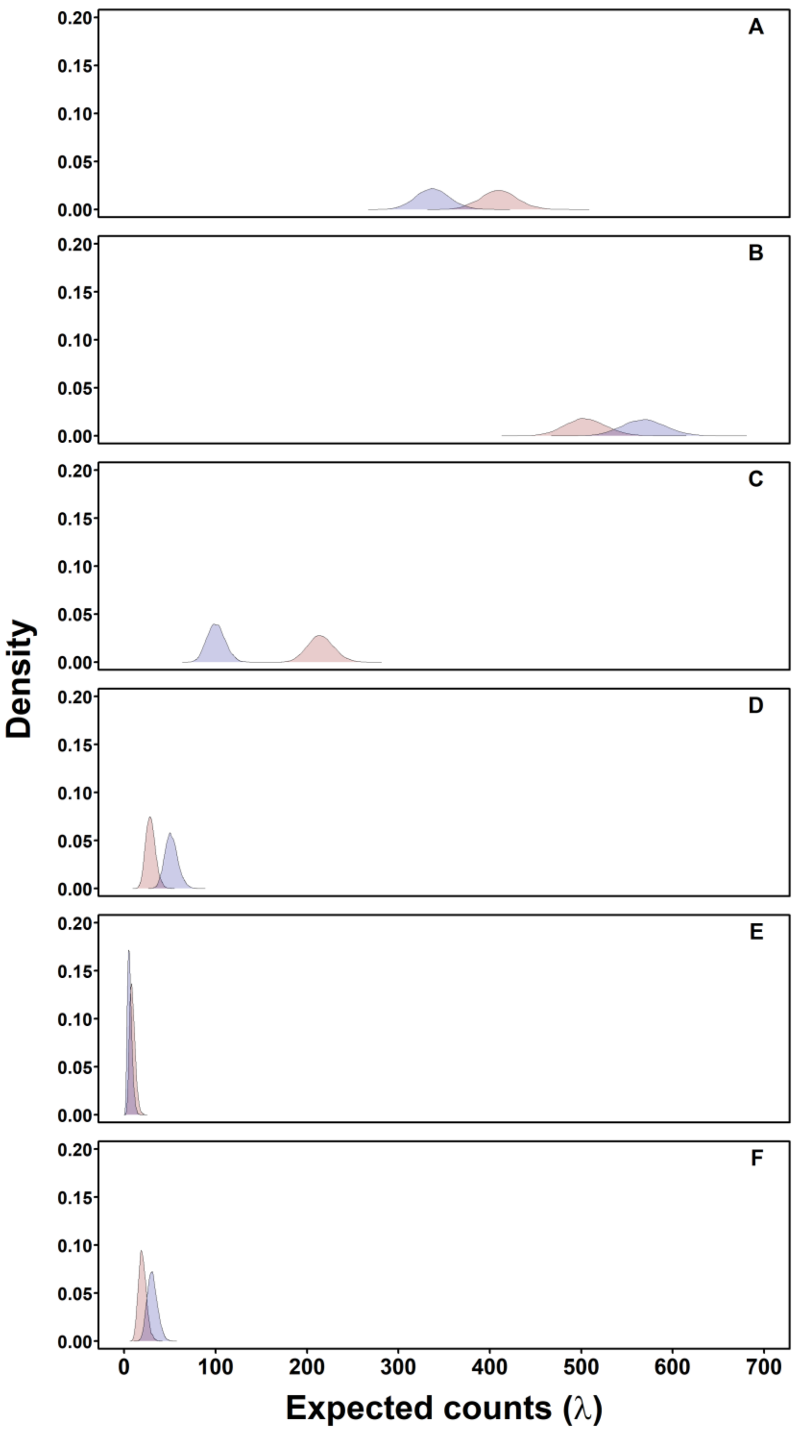

Supplementary Figure S84. Posterior densities of the expected counts (λ) for the consumption of striped venus clams (Red= "Yes" and Blue= "No") per where consumers buy seafood (from A to F: "Fish market", "Large retail", "Local market", "Not buy", "Online", and "Other"), extracted after 10^4 MCMC draws.

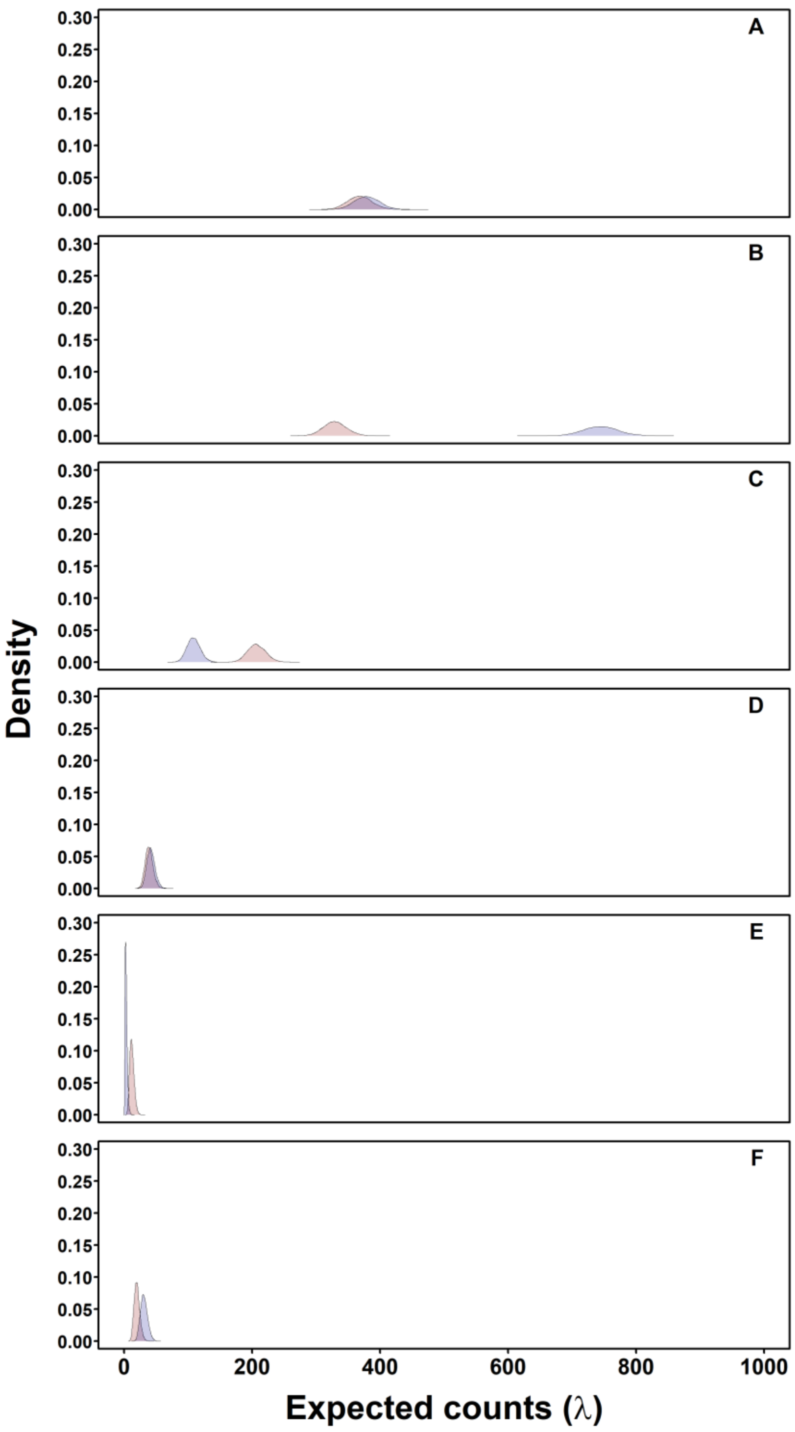

Supplementary Figure S85. Posterior densities of the expected counts (λ) for the consumption of giant red shrimp (Red= "Yes" and Blue= "No") per where consumers buy seafood (from A to F: "Fish market", "Large retail", "Local market", "Not buy", "Online", and "Other"), extracted after 10^4 MCMC draws.

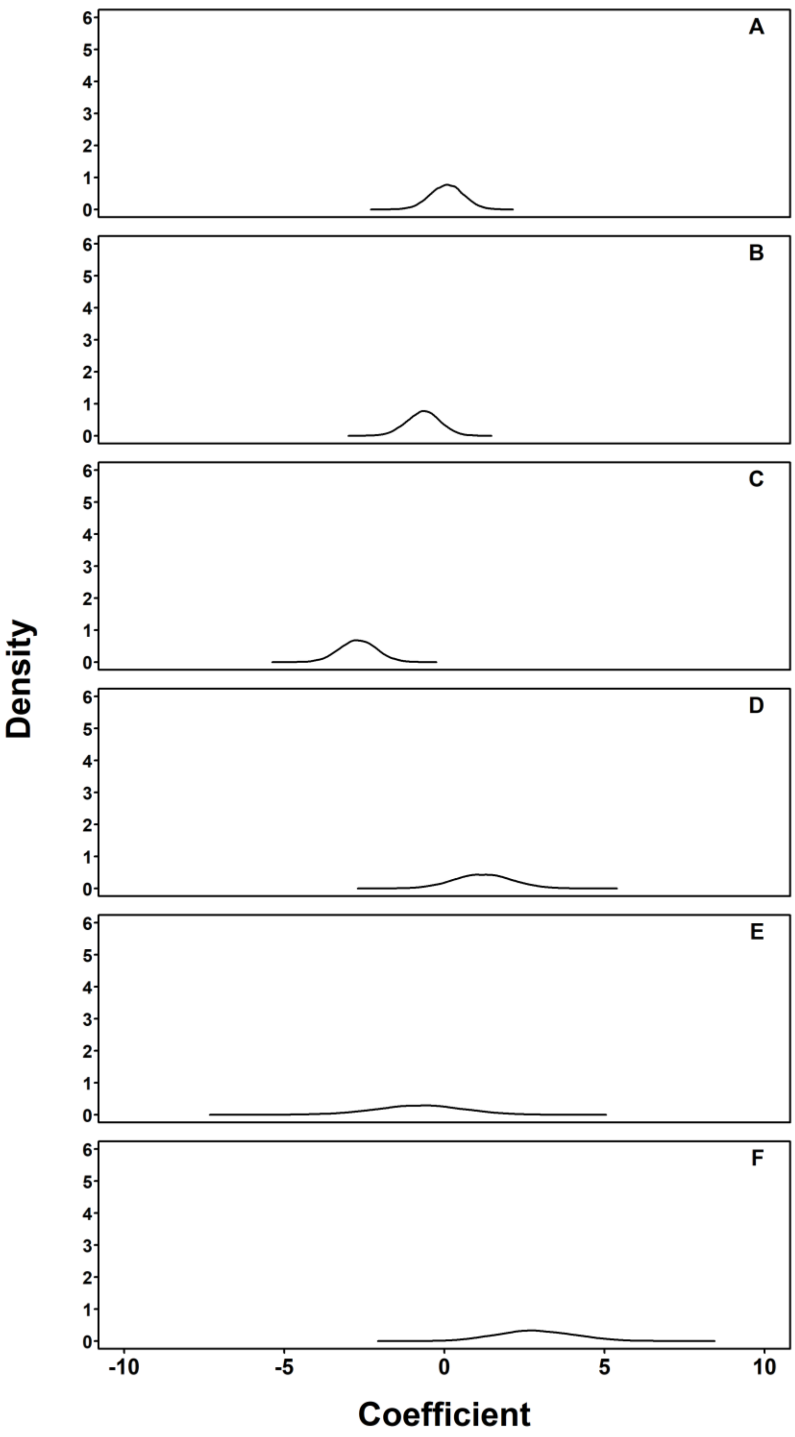

Supplementary Figure S86. Posterior densities of the ANOVA coefficients for the consumers' WTP for giant red shrimp per where consumers buy seafood (from A to F: "Fish market", "Large retail", "Local market", "Not buy", "Online", and "Other"), extracted after 10^4 MCMC draws.

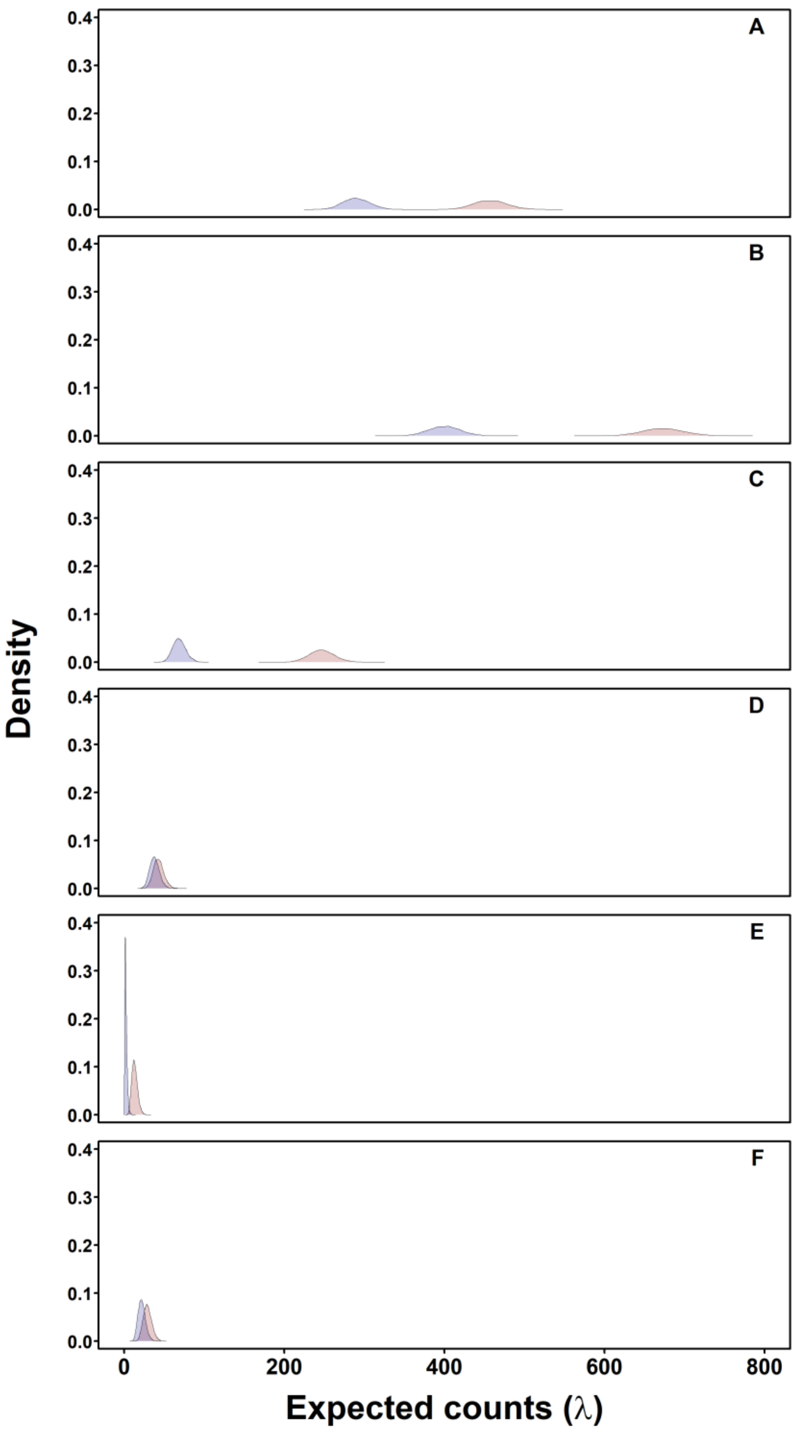

Supplementary Figure S87. Posterior densities of the expected counts (λ) for the consumption of processed albacore tuna (Red= "Yes" and Blue= "No") per where consumers buy seafood (from A to F: "Fish market", "Large retail", "Local market", "Not buy", "Online", and "Other"), extracted after 10^4 MCMC draws.

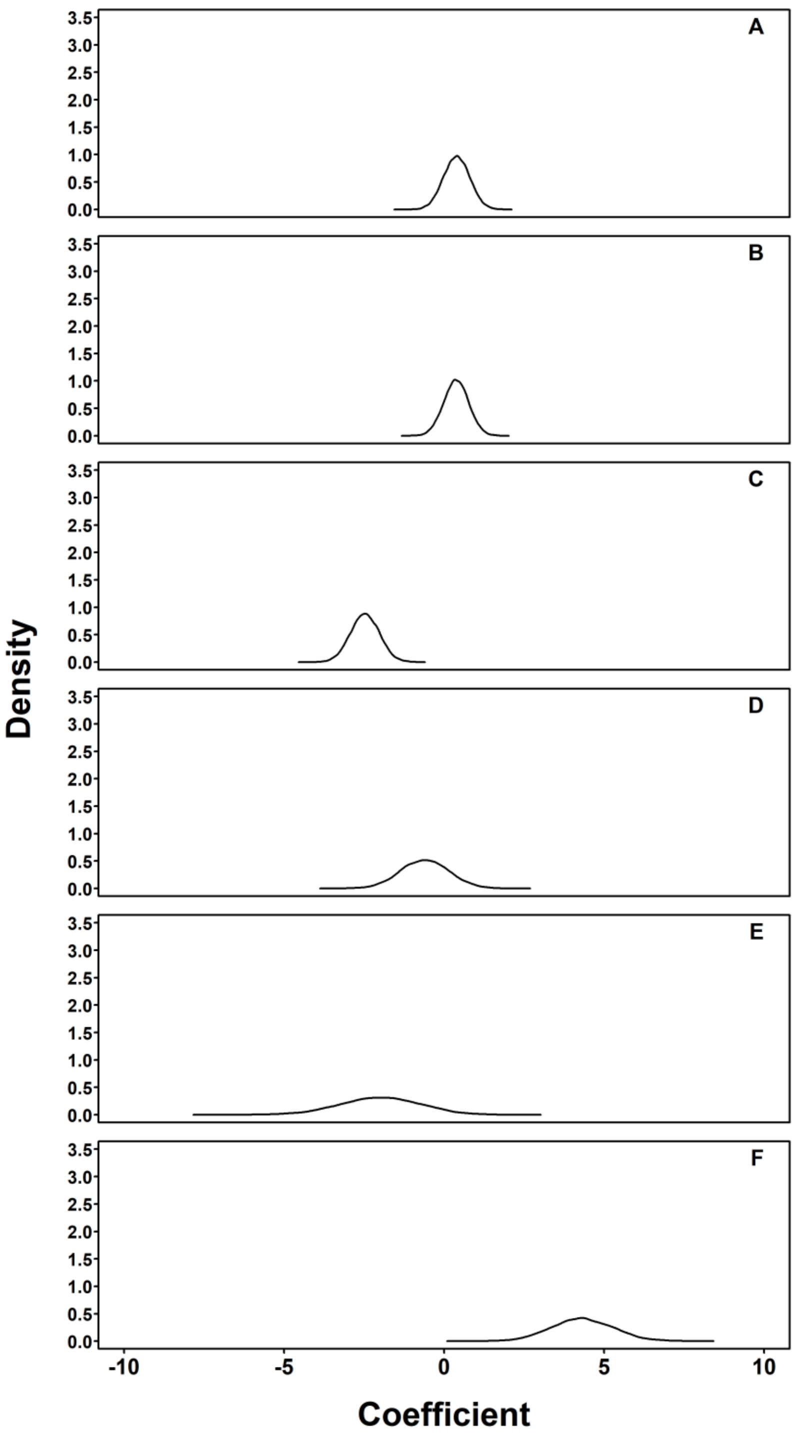

Supplementary Figure S88. Posterior densities of the ANOVA coefficients for the consumers' WTP for processed albacore tuna per where consumers buy seafood (from A to F: "Fish market", "Large retail", "Local market", "Not buy", "Online", and "Other"), extracted after 10^4 MCMC draws.

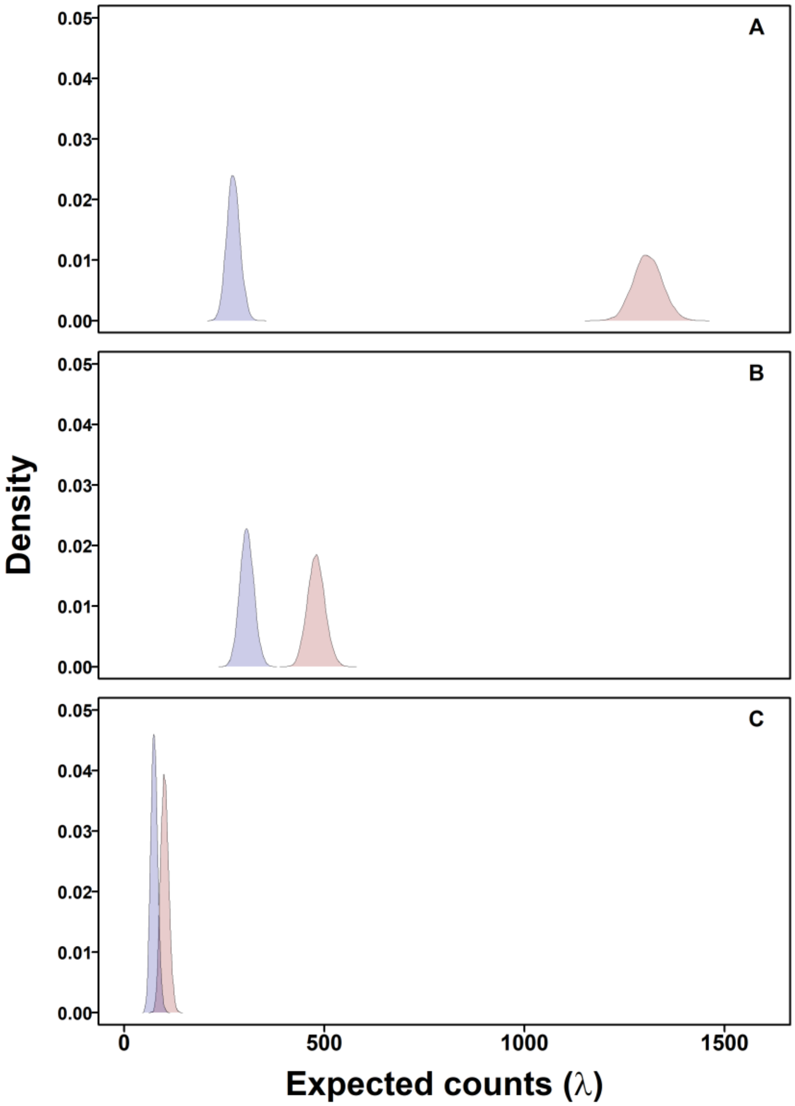

Supplementary Figure S89. Posterior densities of the expected counts (λ) for being informed on seafood origin (Red= "Yes", and Blue= "No") per degree of seafood processing consumers prefer (from A to C: "Fresh" "Frozen", and "Processed"), extracted after 10^4 MCMC draws.

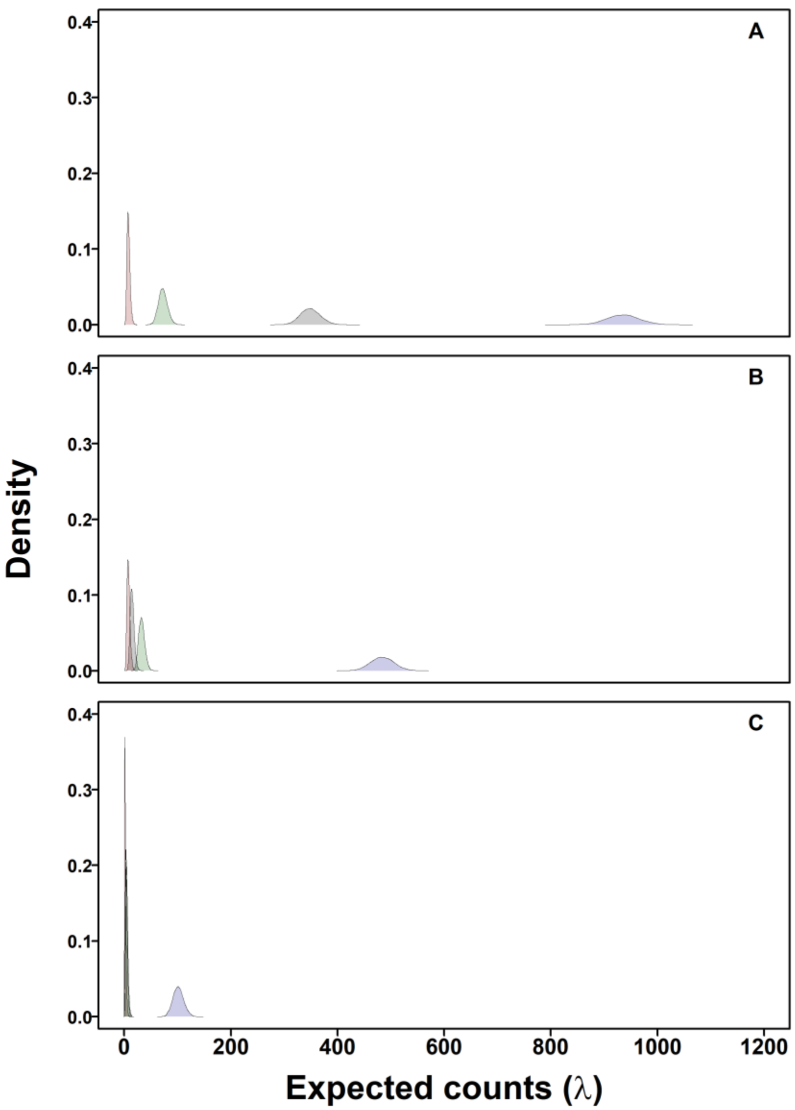

Supplementary Figure S90. Posterior densities of the expected counts (λ) for sources of information on seafood origin (Red= “Ads”, Blue= “Label”, Green= “Other”, and Dark gray= “Retailer”) per degree of seafood processing consumers prefer (from A to C: “Fresh” “Frozen”, and “Processed”), extracted after 10^4 MCMC draws.

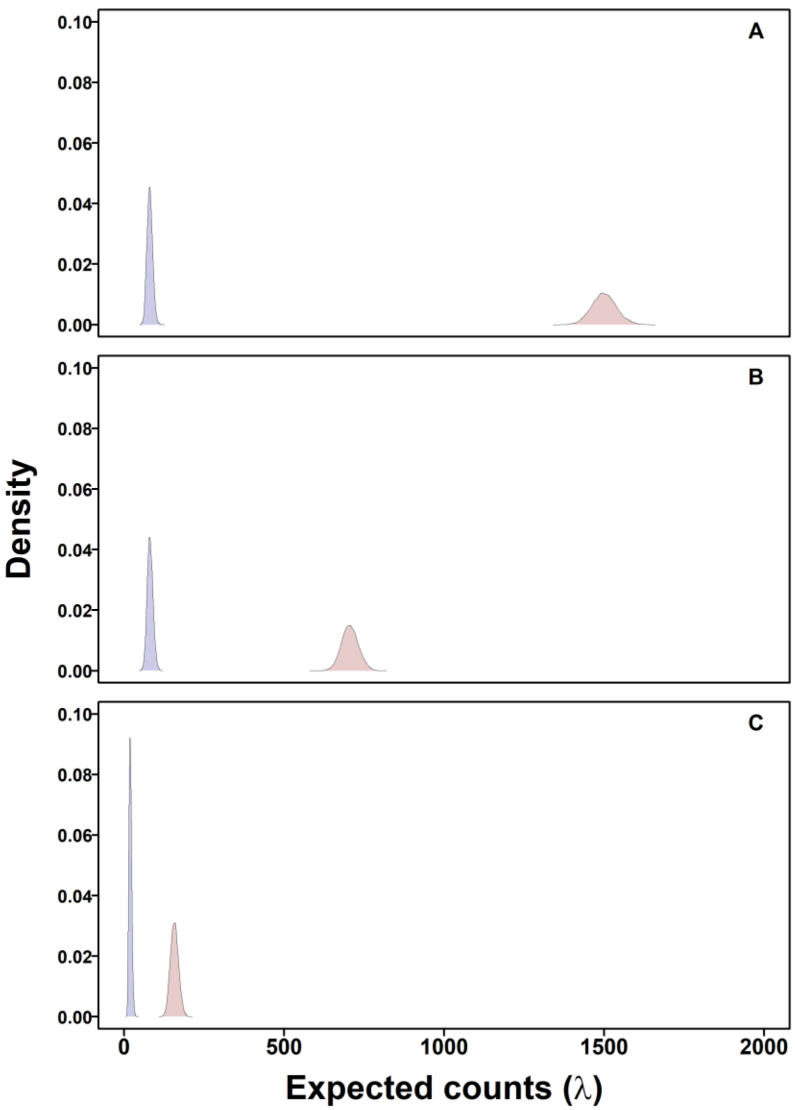

Supplementary Figure S91. Posterior densities of the expected counts (λ) for interest on seafood traceability (Red= "Yes", and Blue= "No") per degree of seafood processing consumers prefer (from A to C: "Fresh" "Frozen", and "Processed"), extracted after 10^4 MCMC draws.

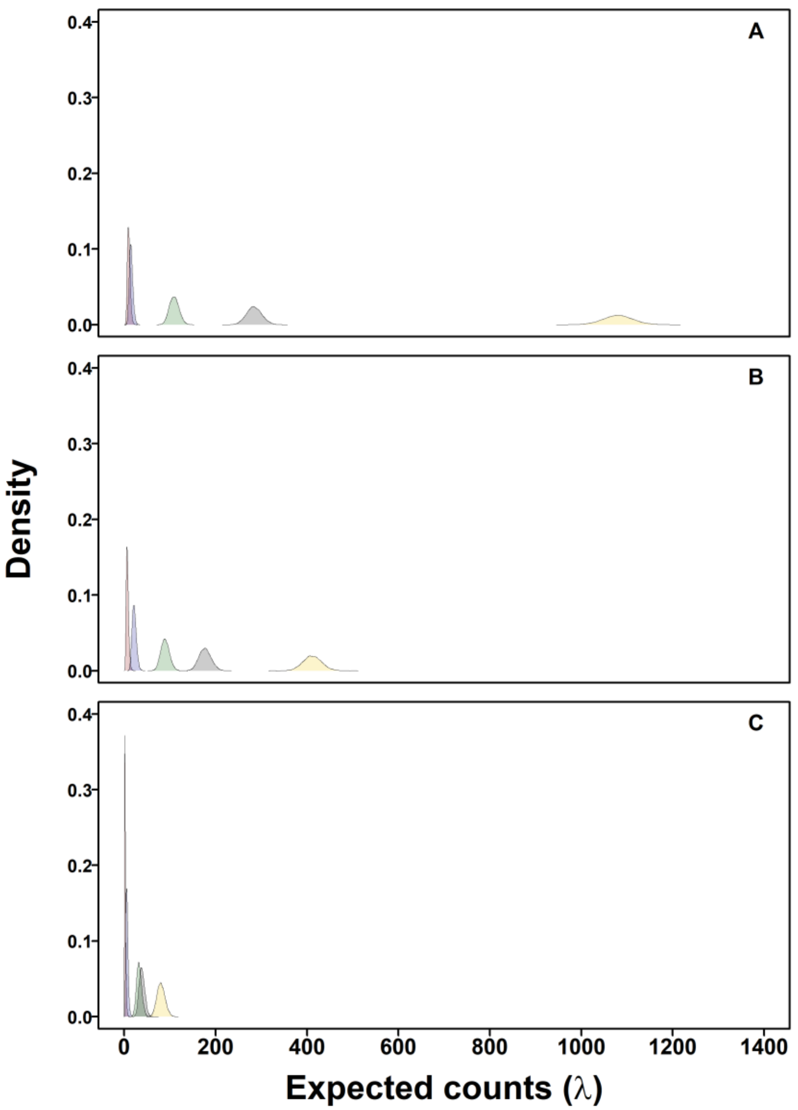

Supplementary Figure S92. Posterior densities of the expected counts (λ) for level of consumers' interest in seafood traceability (Red= 1, Blue= 2, Green= 3, Dark gray= 4, and Yellow= 5) per degree of seafood processing consumers prefer (from A to C: "Fresh" "Frozen", and "Processed"), extracted after 10^4 MCMC draws.

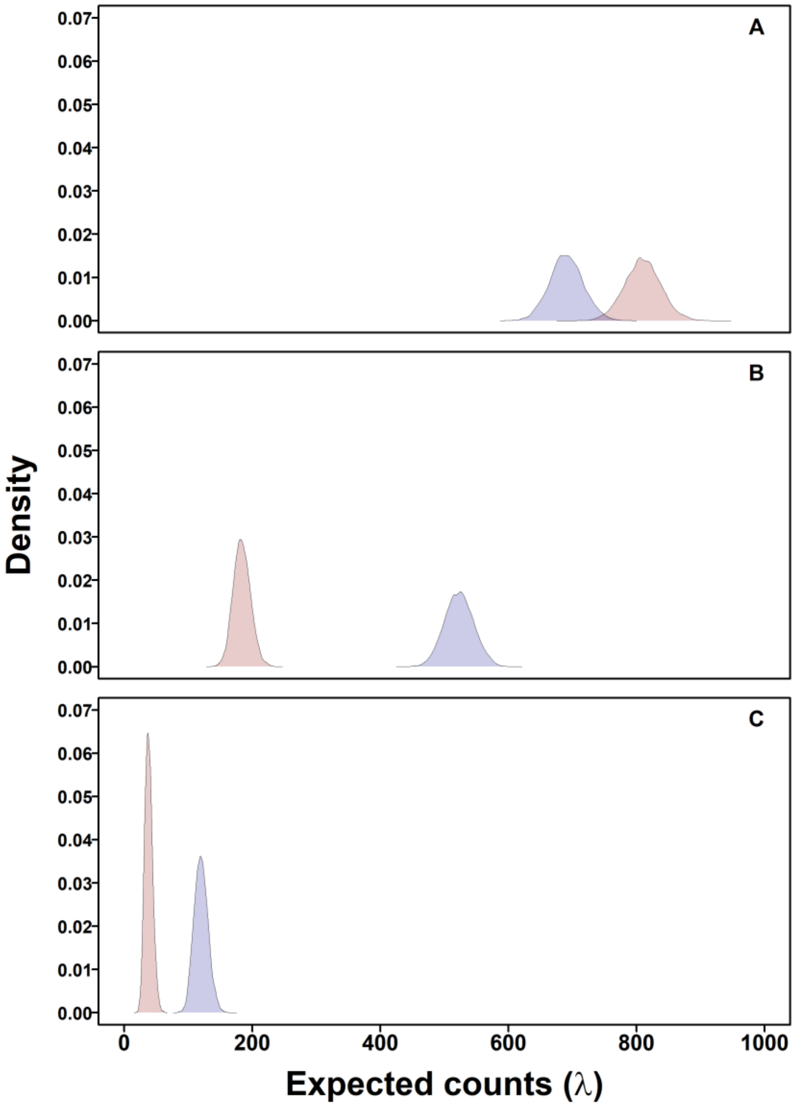

Supplementary Figure S93. Posterior densities of the expected counts (λ) for the consumption of Italian-farmed sea bass (Red= "Yes", and Blue= "No") per degree of seafood processing consumers prefer (from A to C: "Fresh" "Frozen", and "Processed"), extracted after 10^4 MCMC draws.

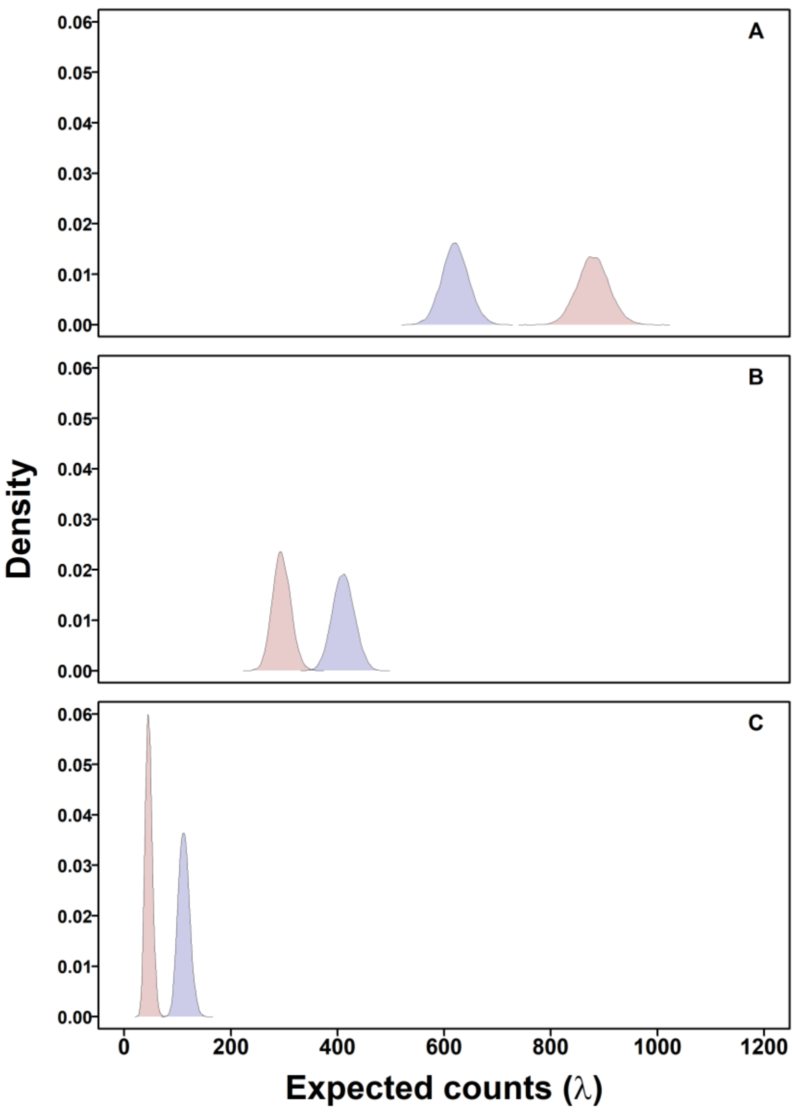

Supplementary Figure S94. Posterior densities of the expected counts (λ) for the consumption of striped venus clams (Red= "Yes", and Blue= "No") per degree of seafood processing consumers prefer (from A to C: "Fresh" "Frozen", and "Processed"), extracted after 10^4 MCMC draws.

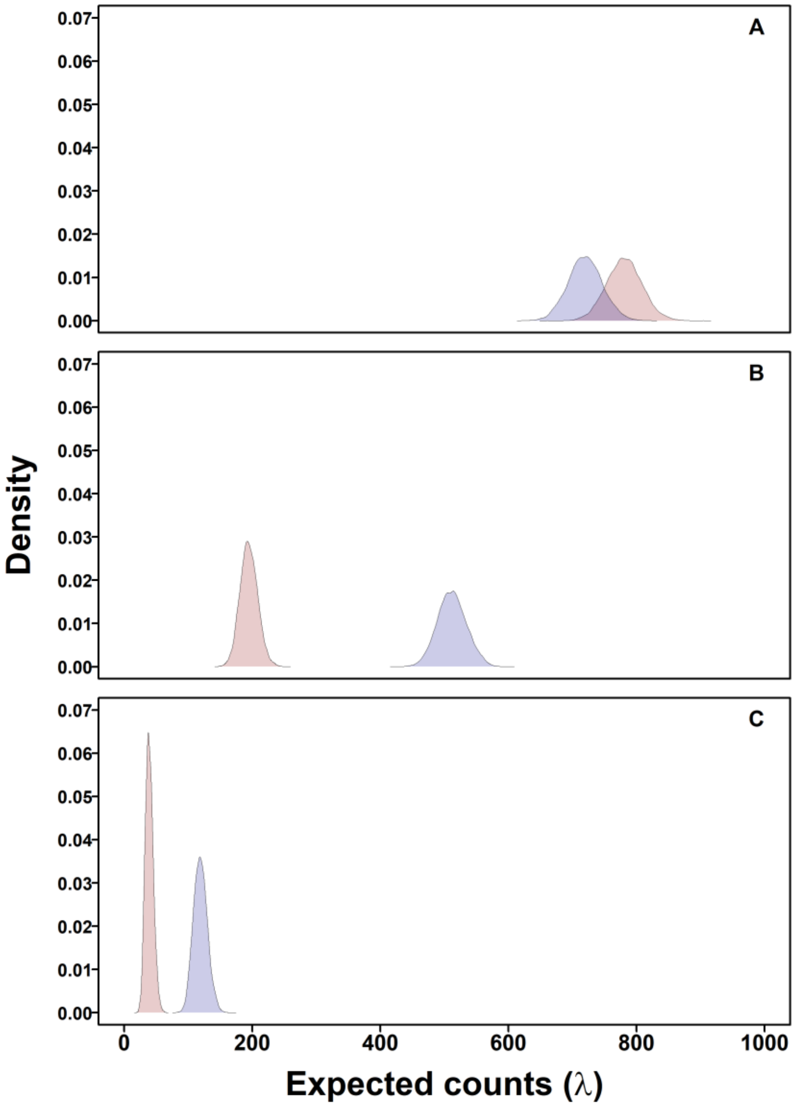

Supplementary Figure S95. Posterior densities of the expected counts (λ) for the consumption of giant red shrimp (Red= "Yes", and Blue= "No") per degree of seafood processing consumers prefer (from A to C: "Fresh" "Frozen", and "Processed"), extracted after 10^4 MCMC draws.

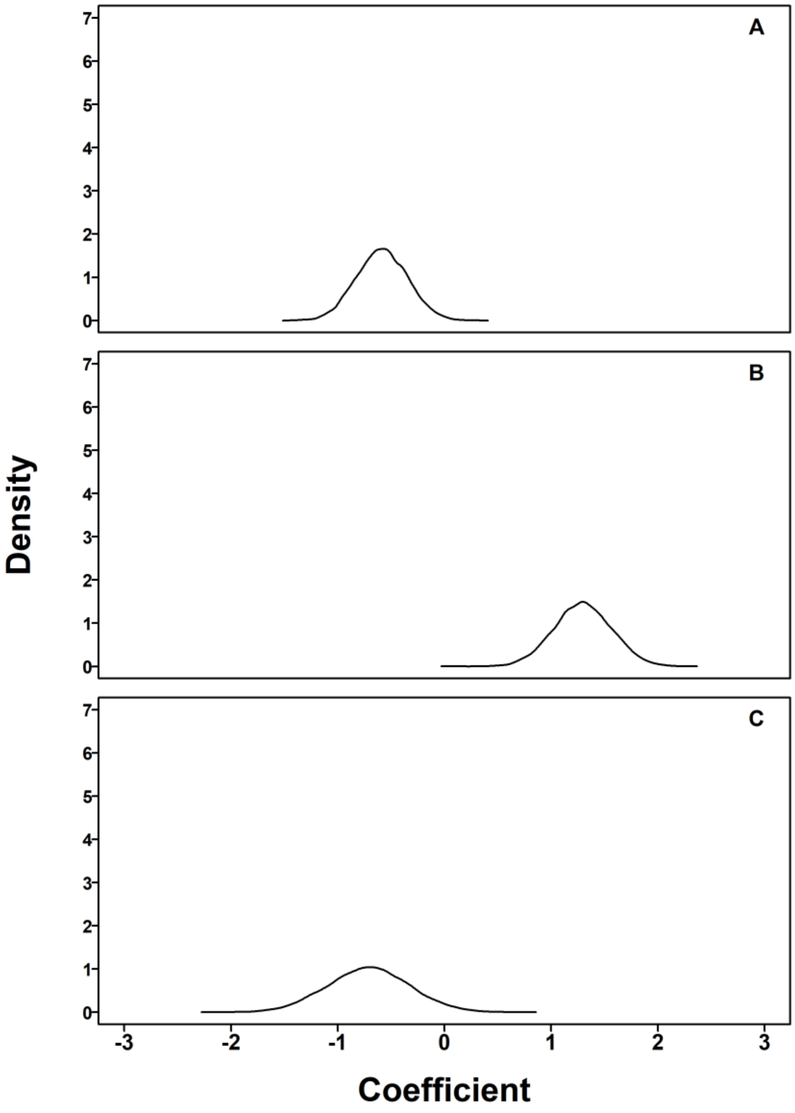

Supplementary Figure S96. Posterior densities of the ANOVA coefficients for the consumers' WTP for processed albacore tuna per degree of seafood processing consumers prefer (from A to C: "Fresh" "Frozen", and "Processed"), extracted after 10^4 MCMC draws.

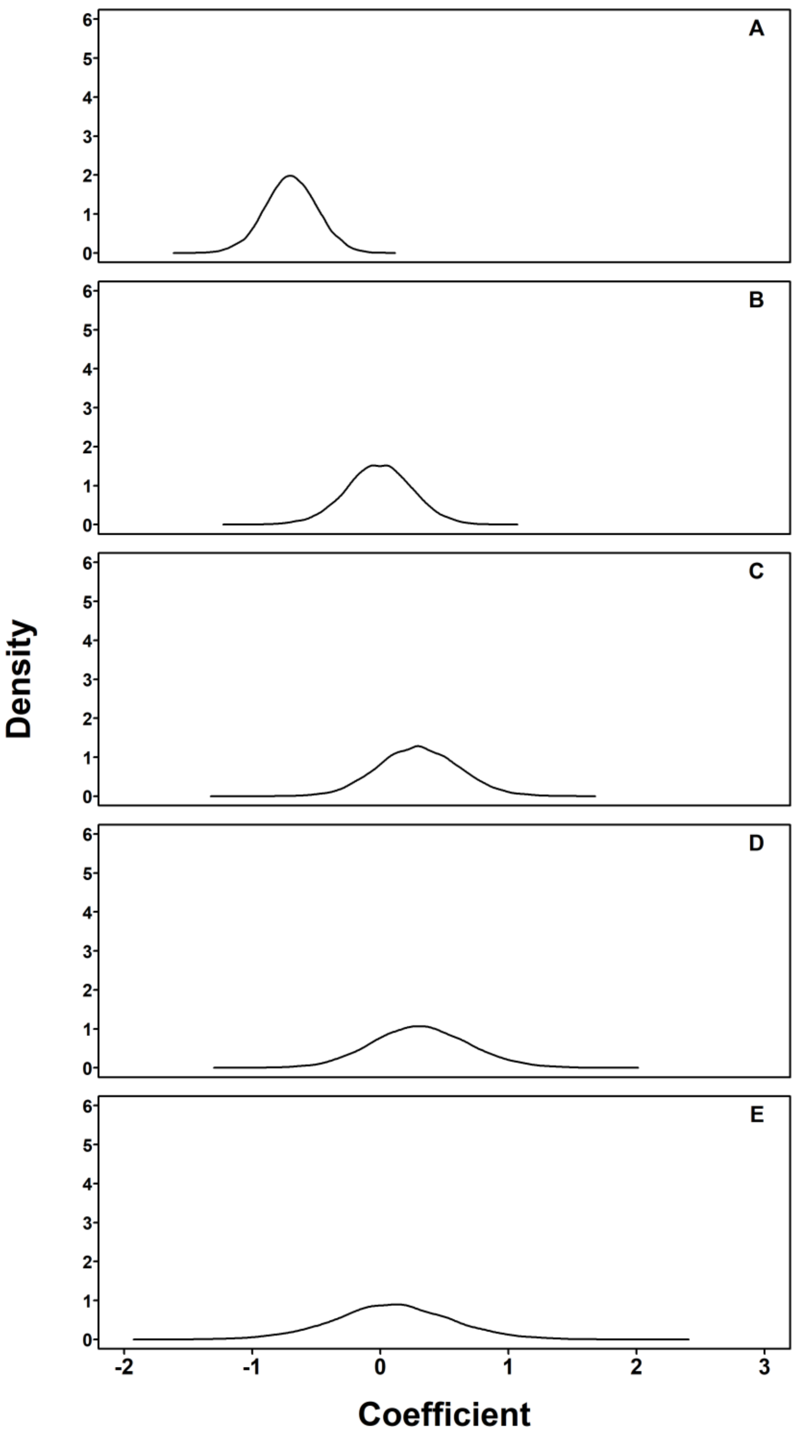

Supplementary Figure S97. Posterior densities of the ANOVA coefficients for the consumers' WTP for striped venus clams per frequency of online seafood purchasing (from A to E: "Never", "Rarely", "Sometimes", "Often", and "Always"), extracted after 10^4 MCMC draws.

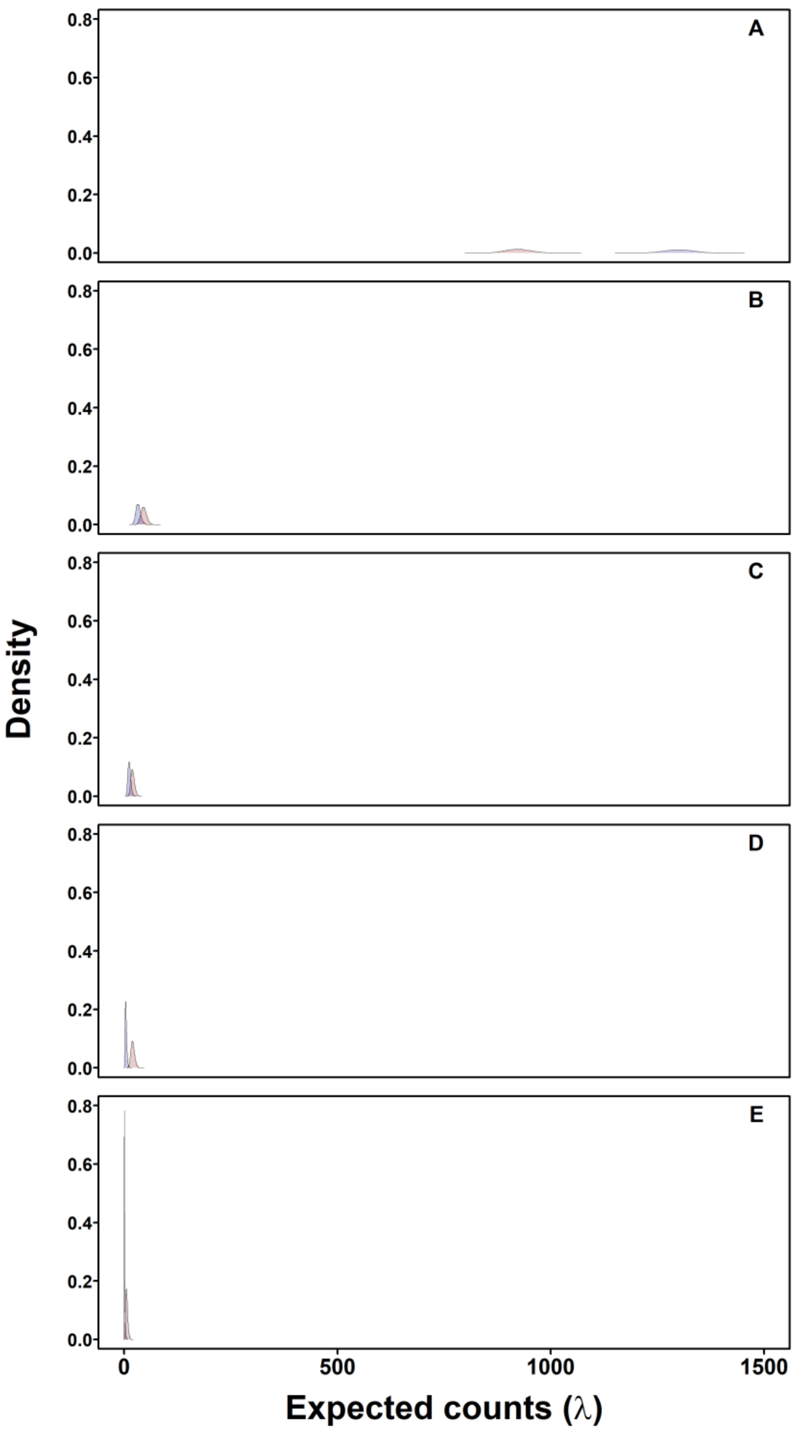

Supplementary Figure S98. Posterior densities of the expected counts (λ) for the consumption of giant red shrimp (Red= "Yes", and Blue= "No") per frequency of online seafood purchasing (from A to E: "Never", "Rarely", "Sometimes", "Often", and "Always"), extracted after 10^4 MCMC draws.

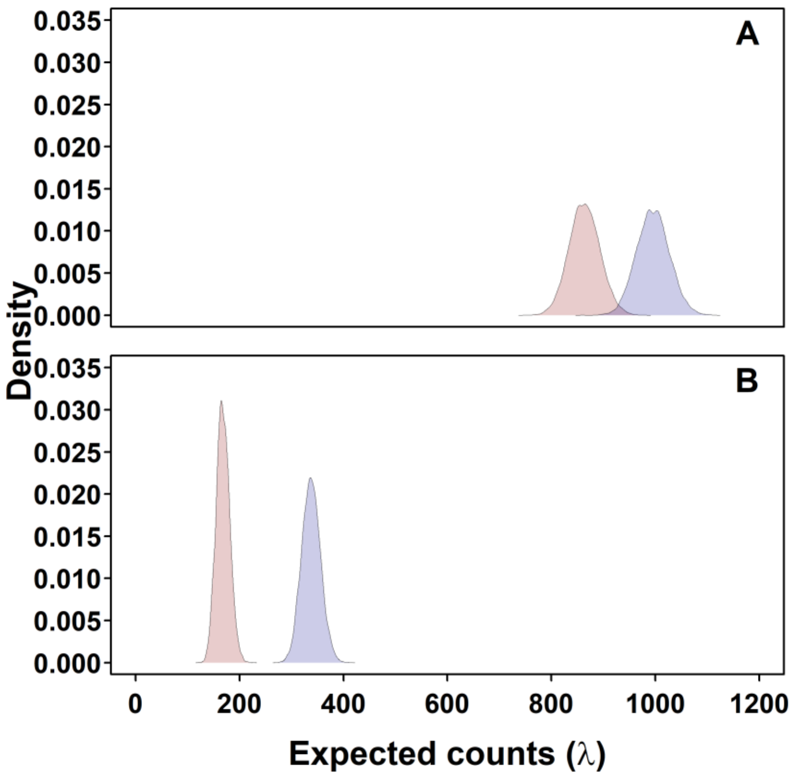

Supplementary Figure 99. Posterior densities of the expected counts (λ) for the consumption of Italian-farmed sea bass (Red= "Yes", and Blue= "No") per being informed on seafood origin (from A to B: "Yes", and "No"), extracted after 10^4 MCMC draws.

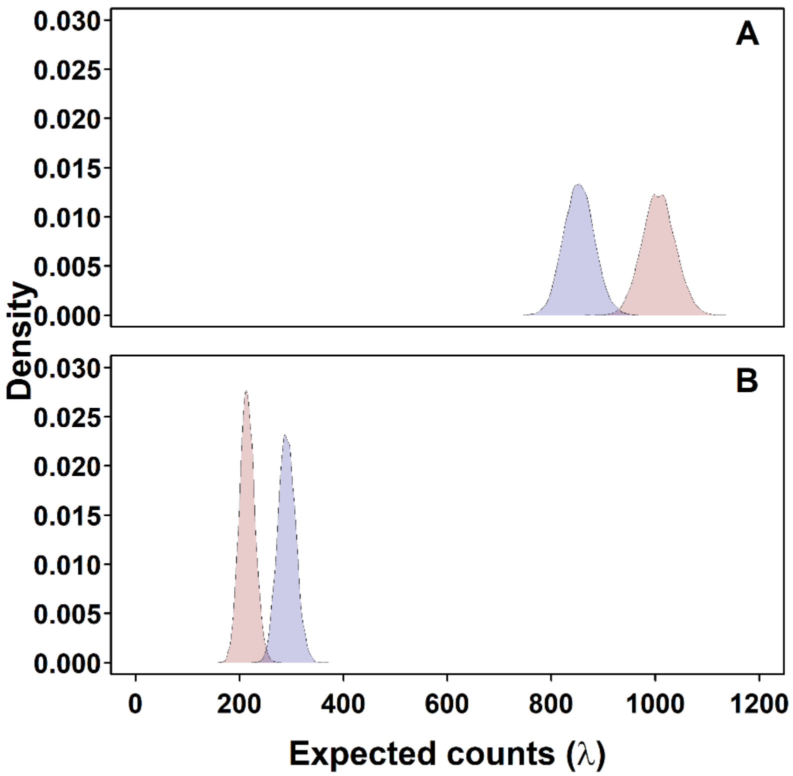

Supplementary Figure 100. Posterior densities of the expected counts (λ) for the consumption of striped venus clams (Red= "Yes", and Blue= "No") per being informed on seafood origin (from A to B: "Yes", and "No"), extracted after 10^4 MCMC draws.

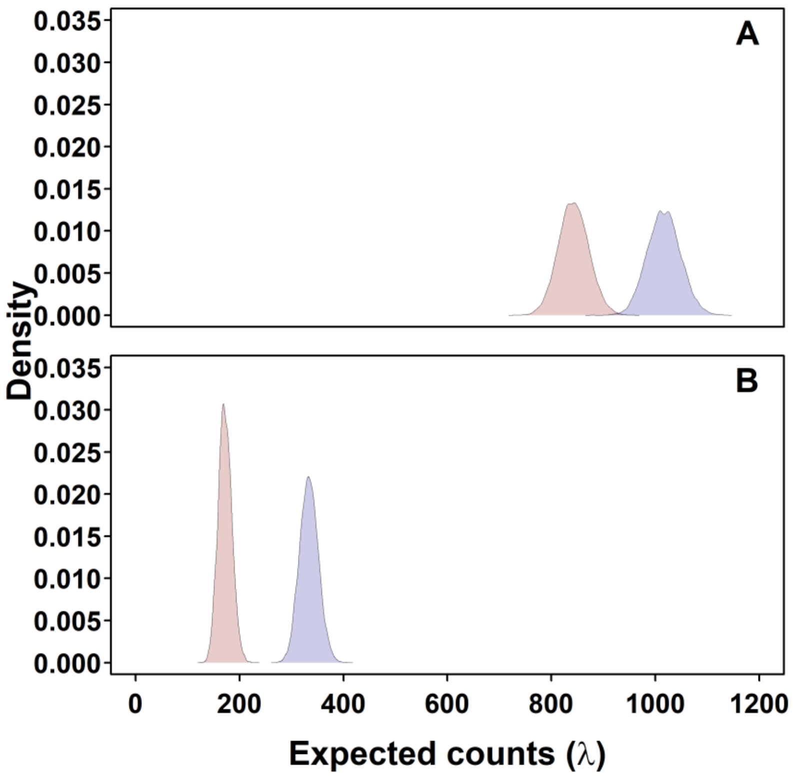

Supplementary Figure 101. Posterior densities of the expected counts (λ) for the consumption of giant red shrimp (Red= "Yes", and Blue= "No") per being informed on seafood origin (from A to B: "Yes", and "No"), extracted after 10^4 MCMC draws.

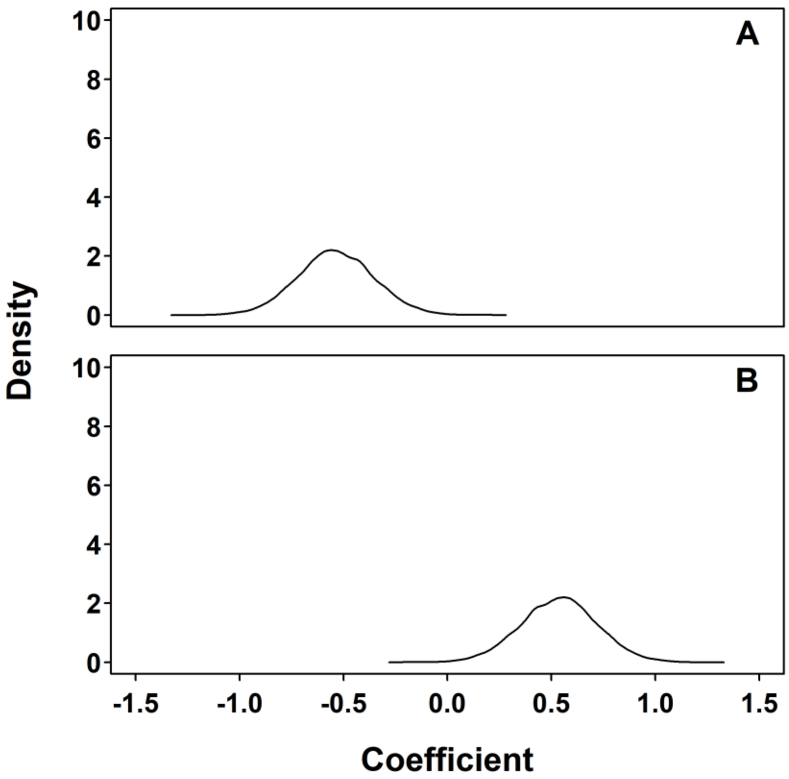

Supplementary Figure 102. Posterior densities of the ANOVA coefficients for the consumers' WTP for processed albacore tuna per being informed on seafood origin (from A to B: "Yes", and "No"), extracted after 10^4 MCMC draws.

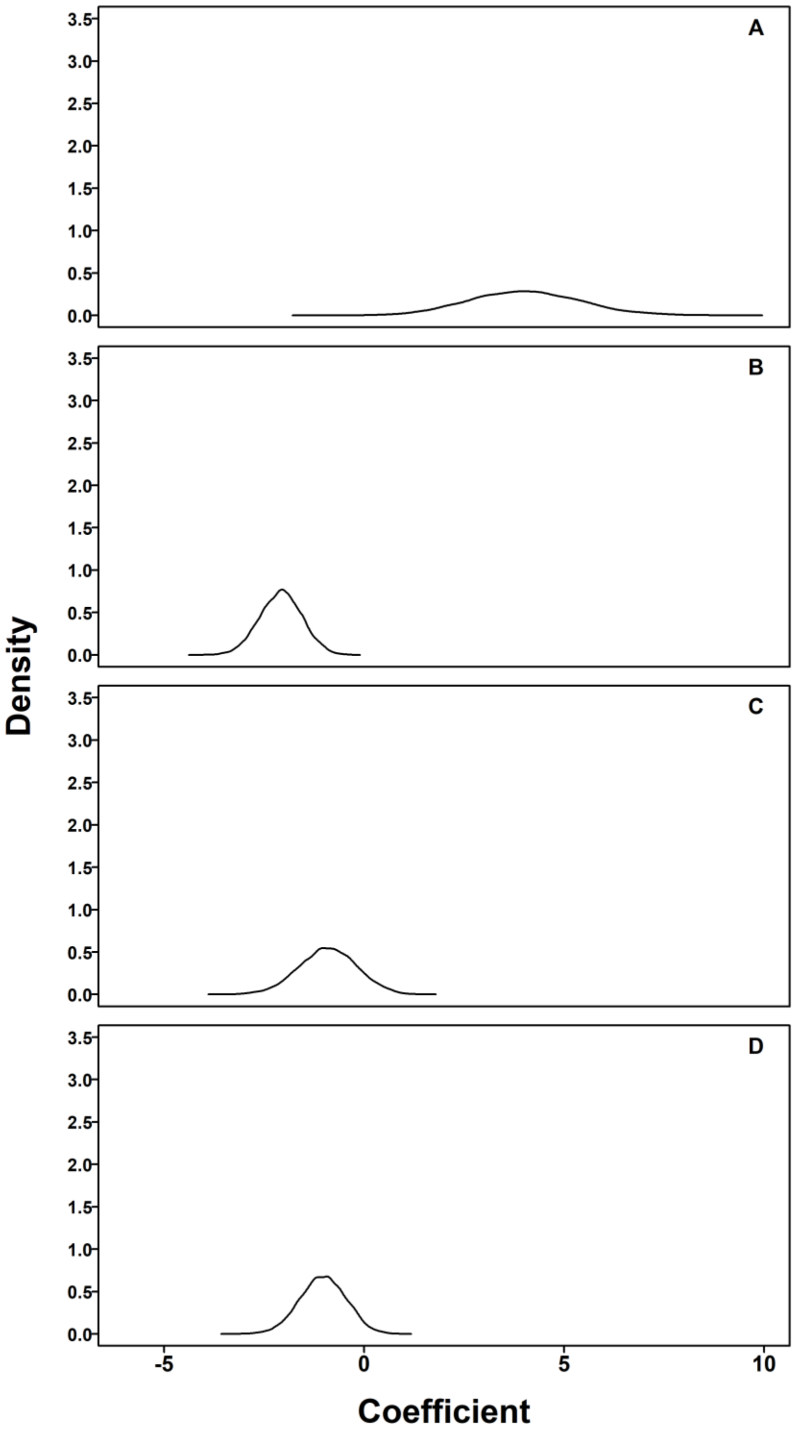

Supplementary Figure 103. Posterior densities of the ANOVA coefficients for the consumers' WTP for processed albacore tuna per source of information on seafood origin (from A to D: "Ads", "Label", "Other", and "Retailer"), extracted after 10^4 MCMC draws.

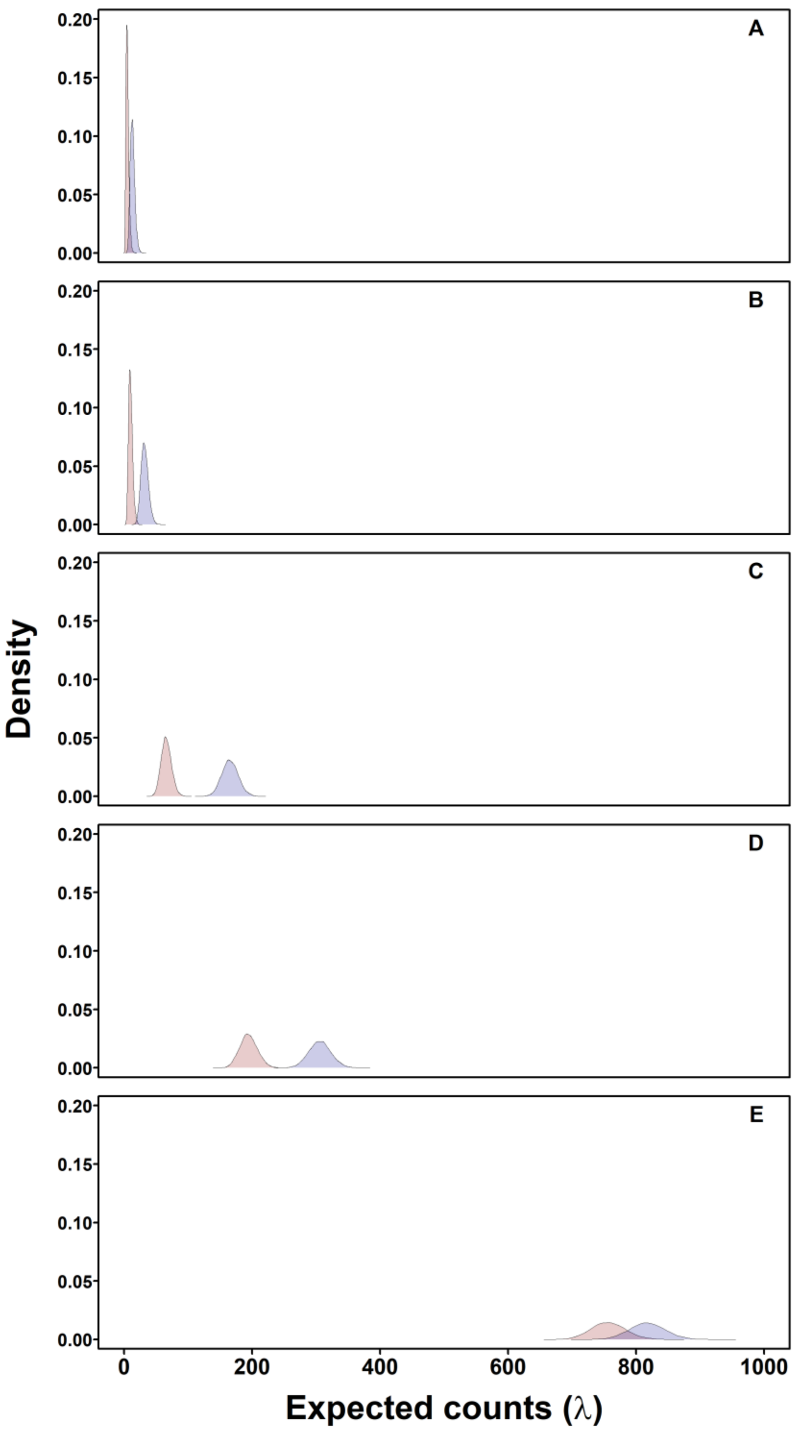

Supplementary Figure 104. Posterior densities of the expected counts (λ) for the consumption of Italian-farmed sea bass (Red= "Yes", and Blue= "No") per level of consumers' interest in seafood traceability (from A to E: 1, 2, 3, 4, and 5), extracted after 10^4 MCMC draws.

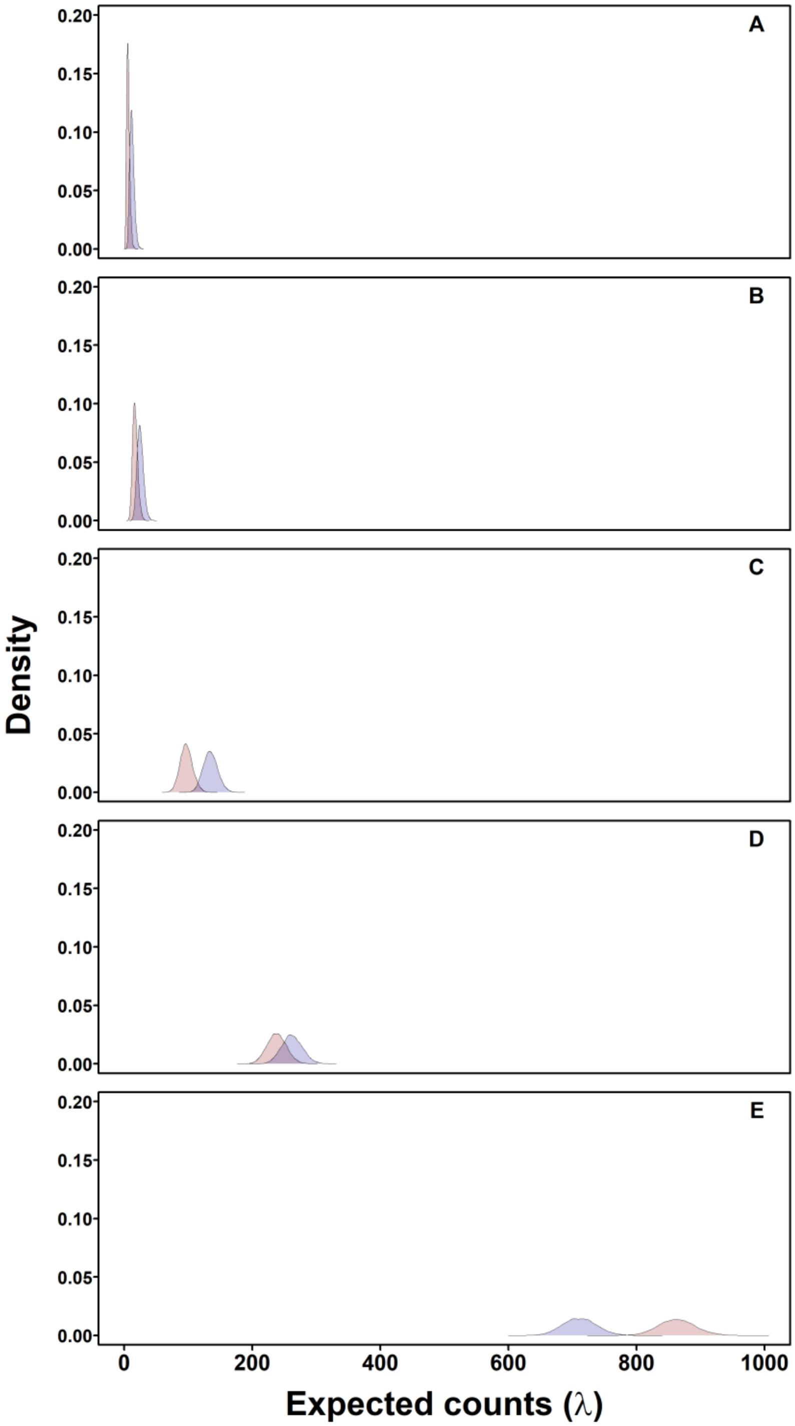

Supplementary Figure 105. Posterior densities of the expected counts (λ) for the consumption of striped venus clams (Red= "Yes", and Blue= "No") per level of consumers' interest in seafood traceability (from A to E: 1, 2, 3, 4, and 5), extracted after 10^4 MCMC draws.

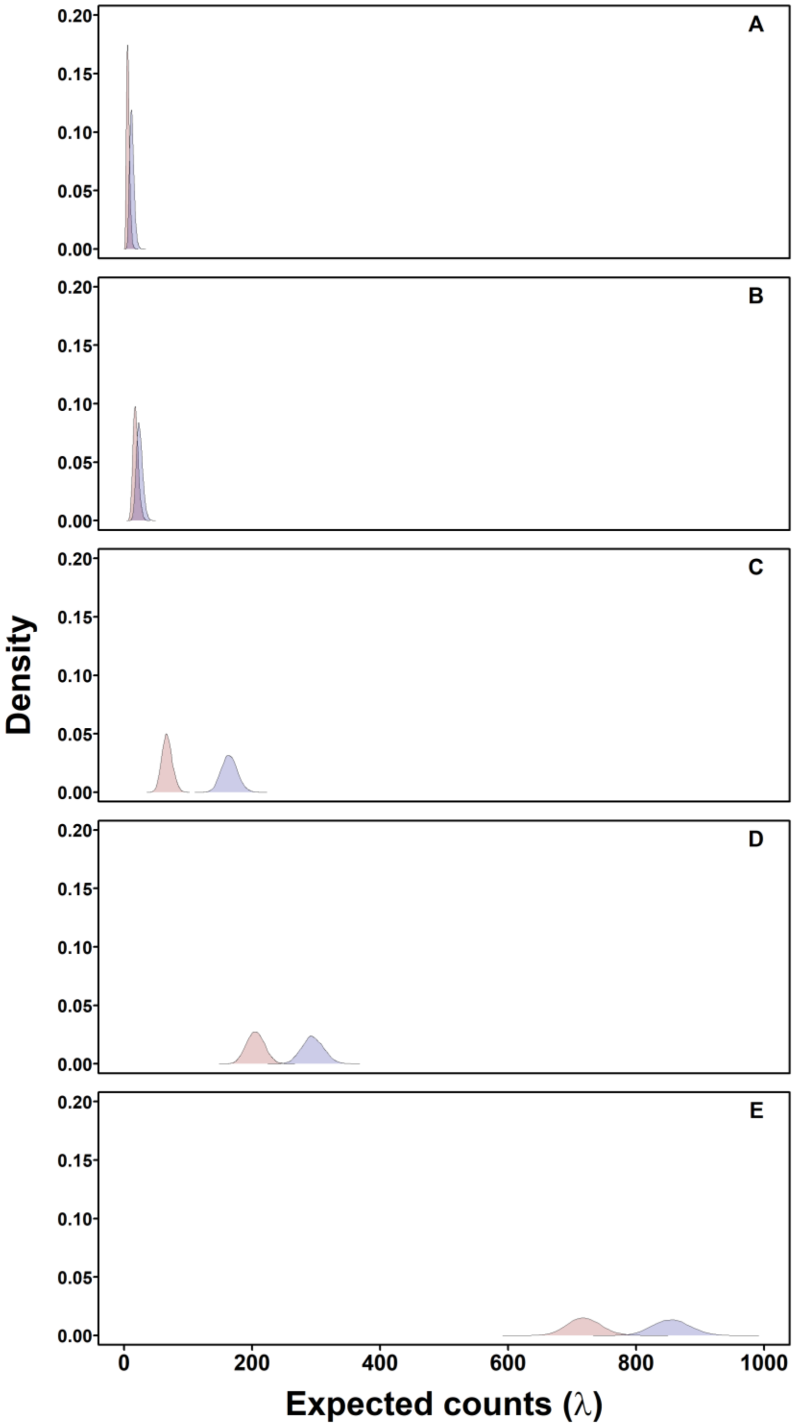

Supplementary Figure 106. Posterior densities of the expected counts (λ) for the consumption of giant red shrimp (Red= "Yes", and Blue= "No") per level of consumers' interest in seafood traceability (from A to E: 1, 2, 3, 4, and 5), extracted after 10^4 MCMC draws.

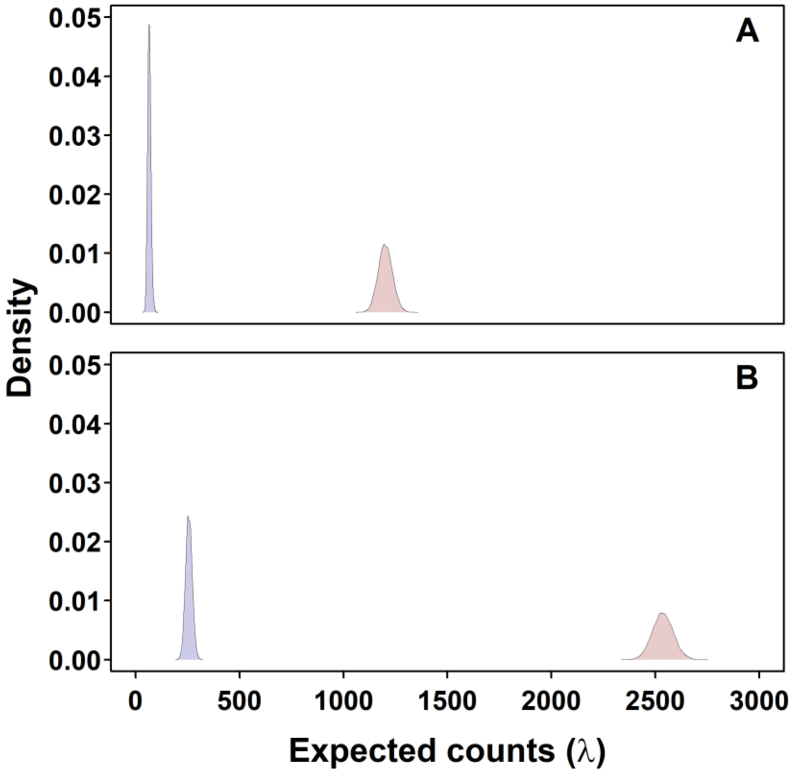

Supplementary Figure 107. Posterior densities of the expected counts (λ) for the consumption of seafood (Red= "Yes", and Blue= "No") per year (from A to B: 2022, and 2024), extracted after 10^4 MCMC draws.

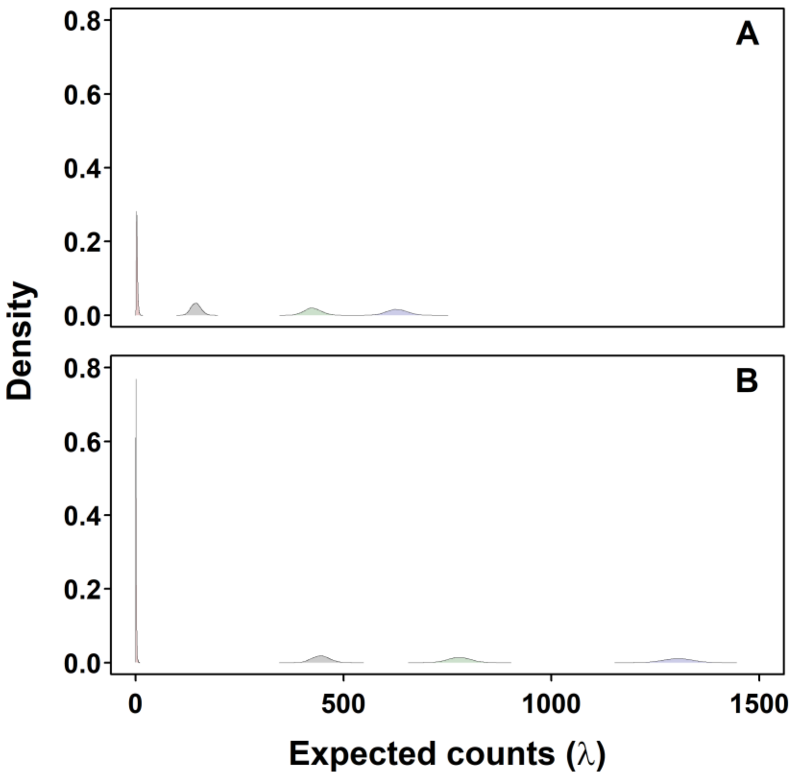

Supplementary Figure 108. Posterior densities of the expected counts (λ) for monthly seafood consumption rate (Red= 0, Blue= 1–5, Green= 6–10, and Dark gray= 10+) per year (from A to B: 2022, and 2024), extracted after 10^4 MCMC draws.

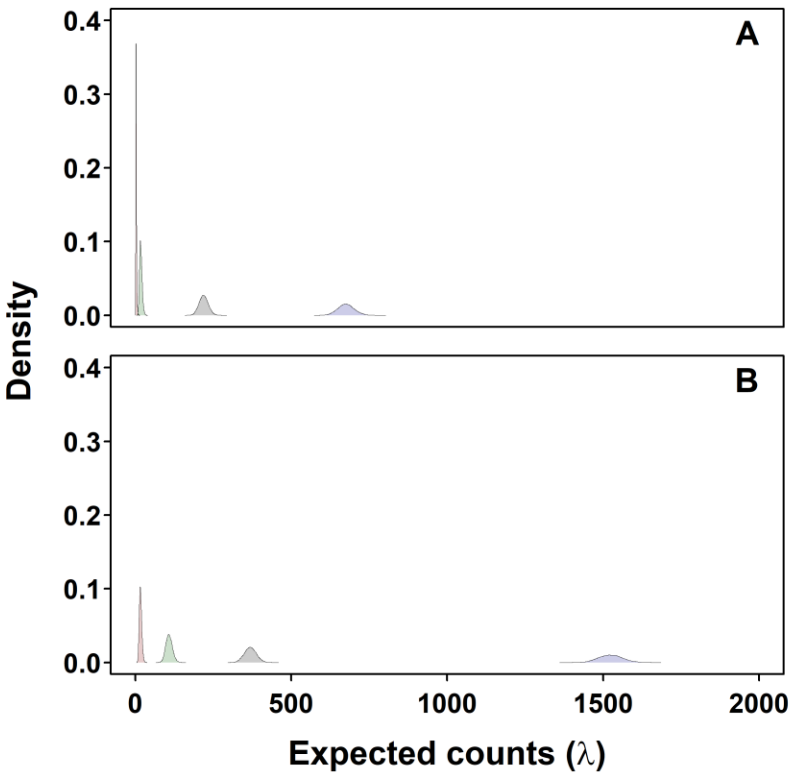

Supplementary Figure 109. Posterior densities of the expected counts (λ) for sources of information on seafood origin (Red= “Ads”, Blue= “Label”, Green= “Other”, and Dark gray= “Retailer”) per year (from A to B: 2022, and 2024), extracted after 10^4 MCMC draws.

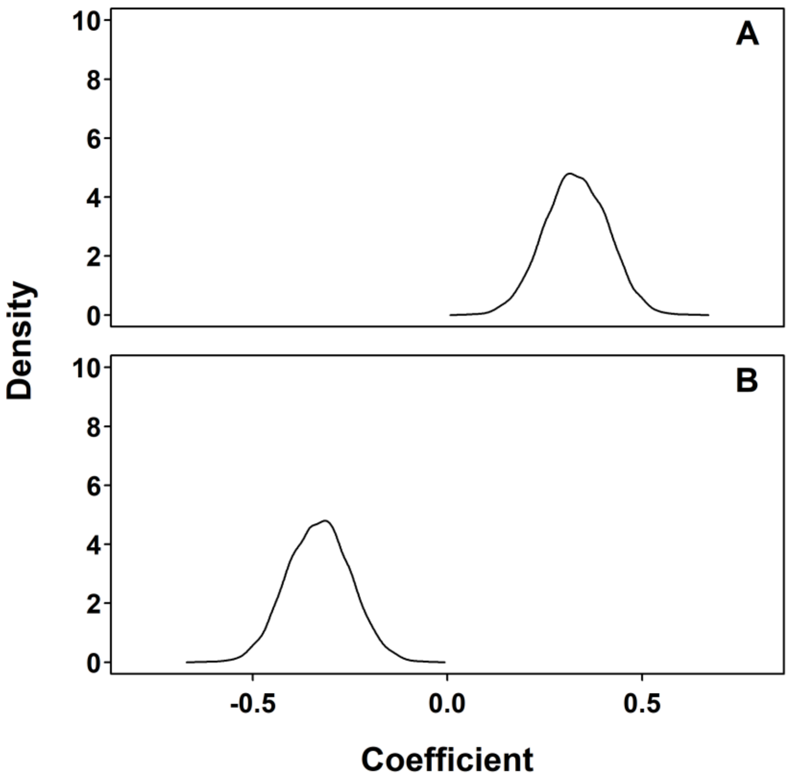

Supplementary Figure 110. Posterior densities of the ANOVA coefficients for the consumers' WTP for Italian-farmed sea bass per year (from A to B: 2022, and 2024), extracted after 10^4 MCMC draws.

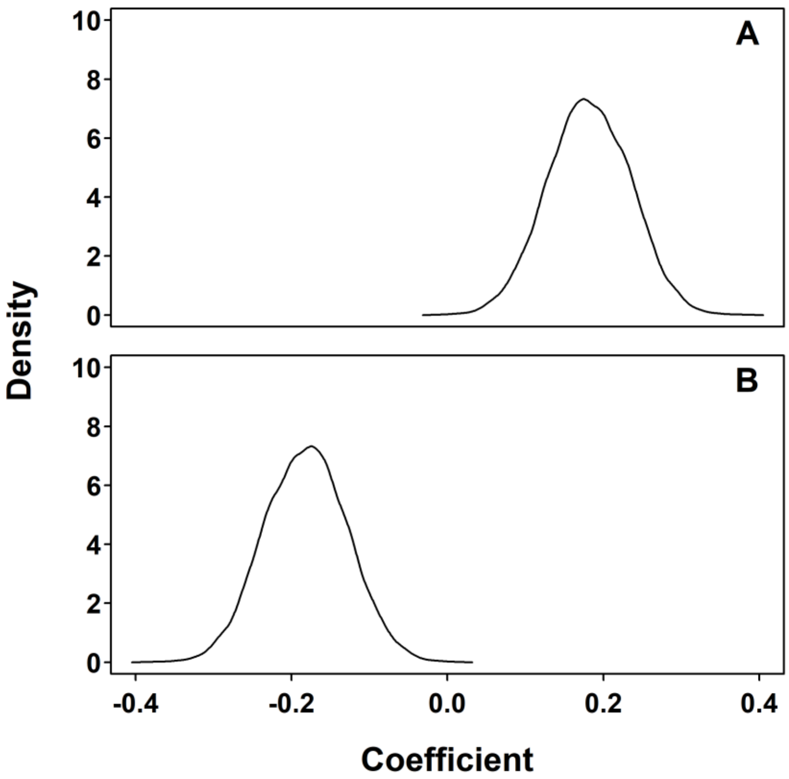

Supplementary Figure 111. Posterior densities of the ANOVA coefficients for the consumers' WTP for striped venus clams per year (from A to B: 2022, and 2024), extracted after 10^4 MCMC draws.

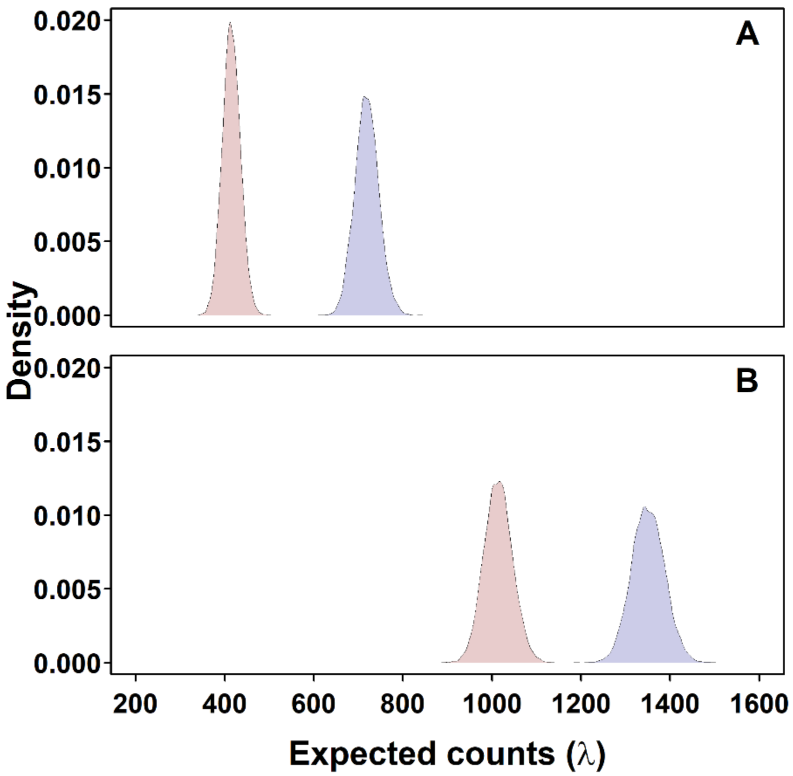

Supplementary Figure 112. Posterior densities of the expected counts (λ) for the consumption of giant red shrimp (Red= “Yes”, and Blue= “No”) per year (from A to B: 2022, and 2024), extracted after 10^4 MCMC draws.

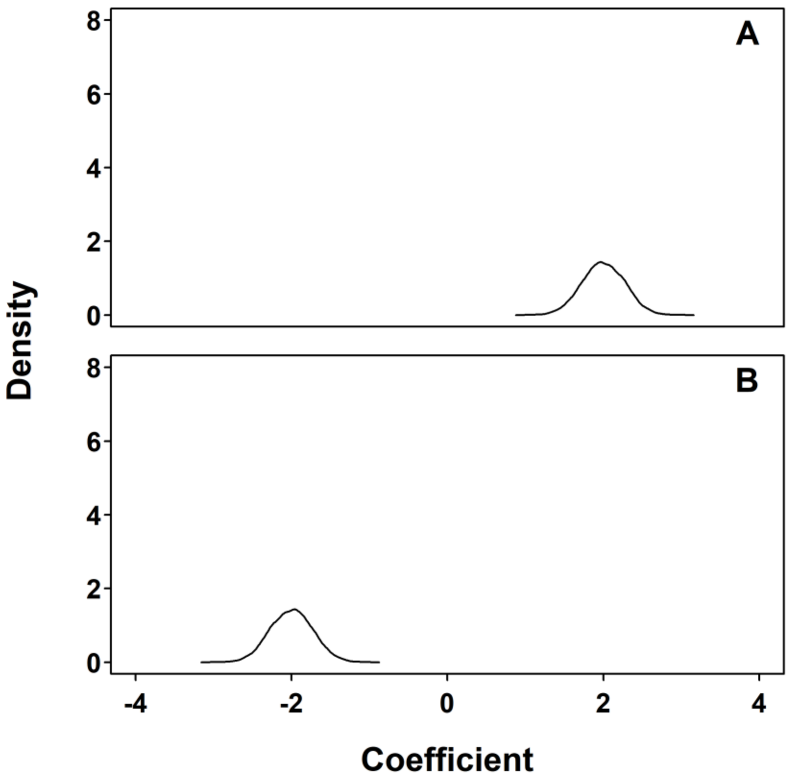

Supplementary Figure 113. Posterior densities of the ANOVA coefficients for the consumers' WTP for giant red shrimp per year (from A to B: 2022, and 2024), extracted after 10^4 MCMC draws.

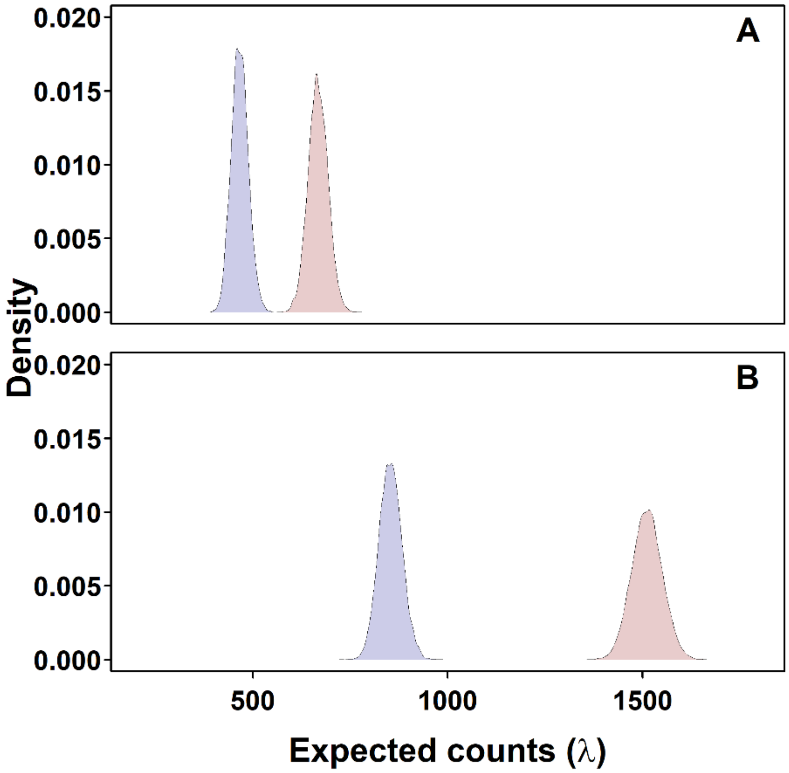

Supplementary Figure 114. Posterior densities of the expected counts (λ) for the consumption of processed albacore tuna (Red= "Yes", and Blue= "No") per year (from A to B: 2022, and 2024), extracted after 10^4 MCMC draws.

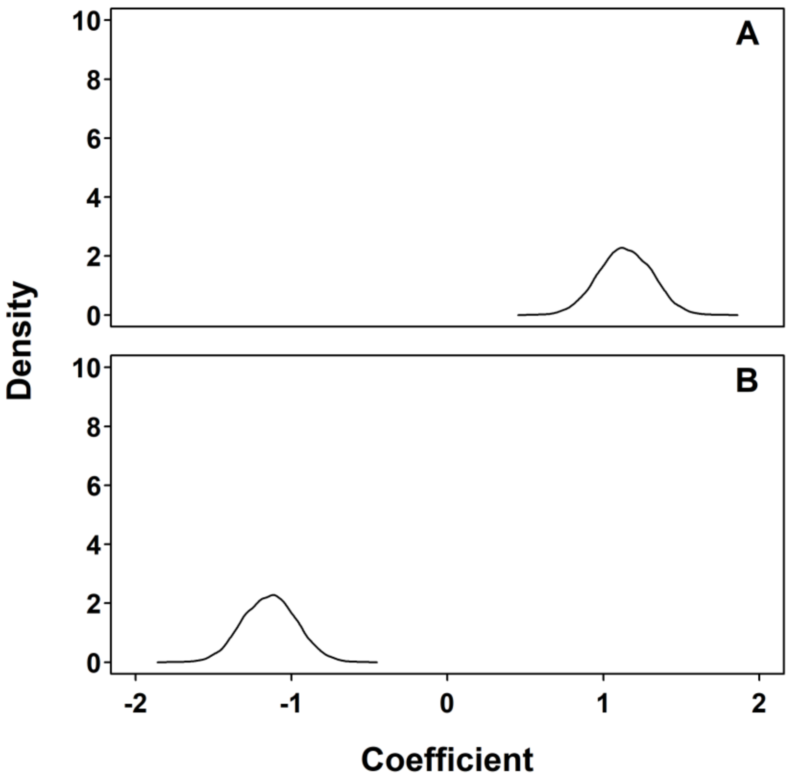

Supplementary Figure 115. Posterior densities of the ANOVA coefficients for the consumers' WTP for processed albacore tuna per year (from A to B: 2022, and 2024), extracted after 10^4 MCMC draws.

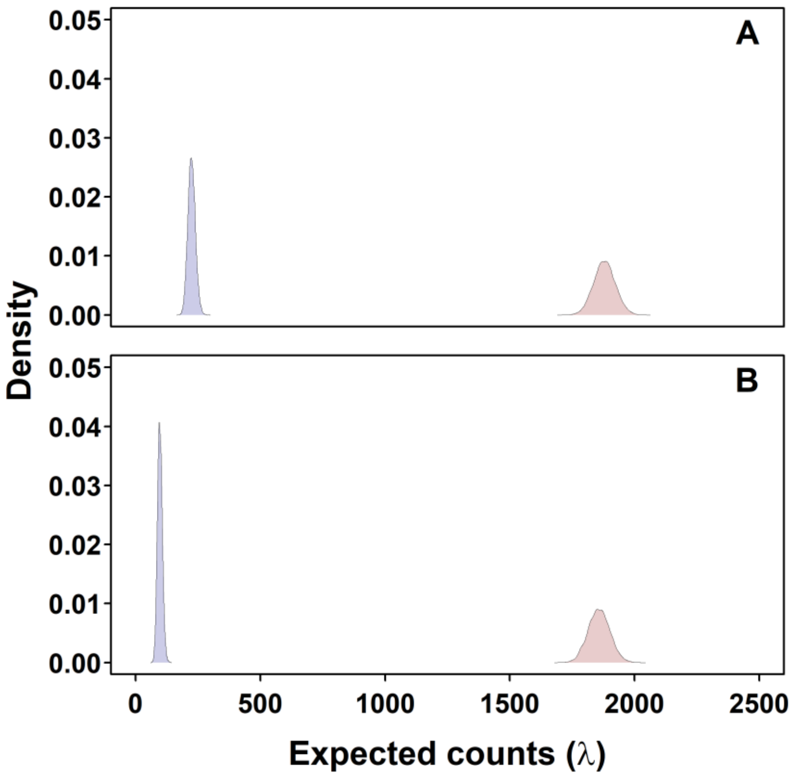

Supplementary Figure 116. Posterior densities of the expected counts (λ) for the consumption of seafood (Red= "Yes", and Blue= "No") per sex (from A to B: "Female", and "Male"), extracted after 10^4 MCMC draws.

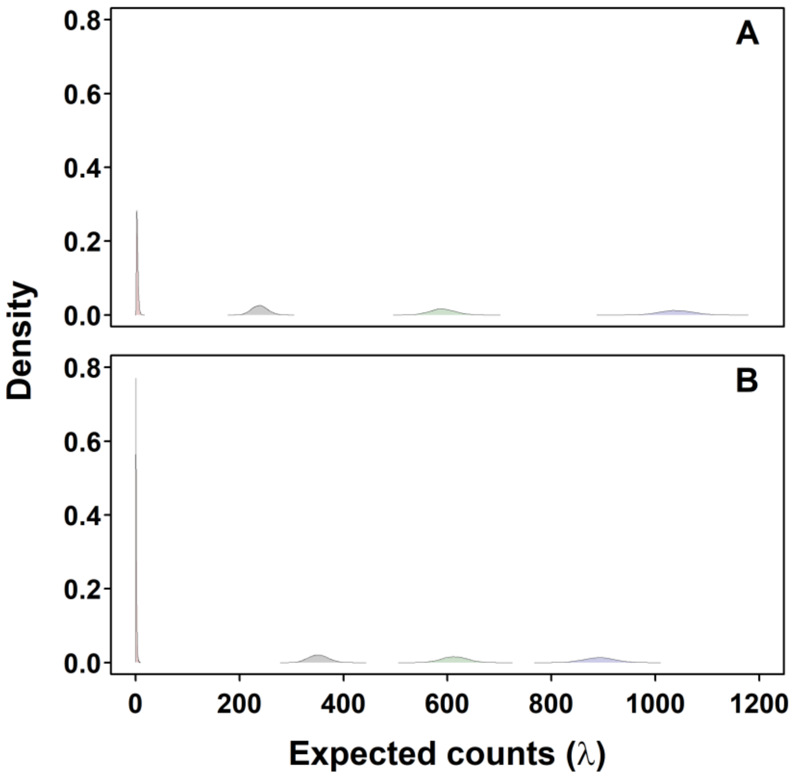

Supplementary Figure 117. Posterior densities of the expected counts (λ) for monthly seafood consumption rate (Red= 0, Blue= 1–5, Green= 6–10, and Dark gray= 10+) per sex (from A to B: "Female", and "Male"), extracted after 10^4 MCMC draws.

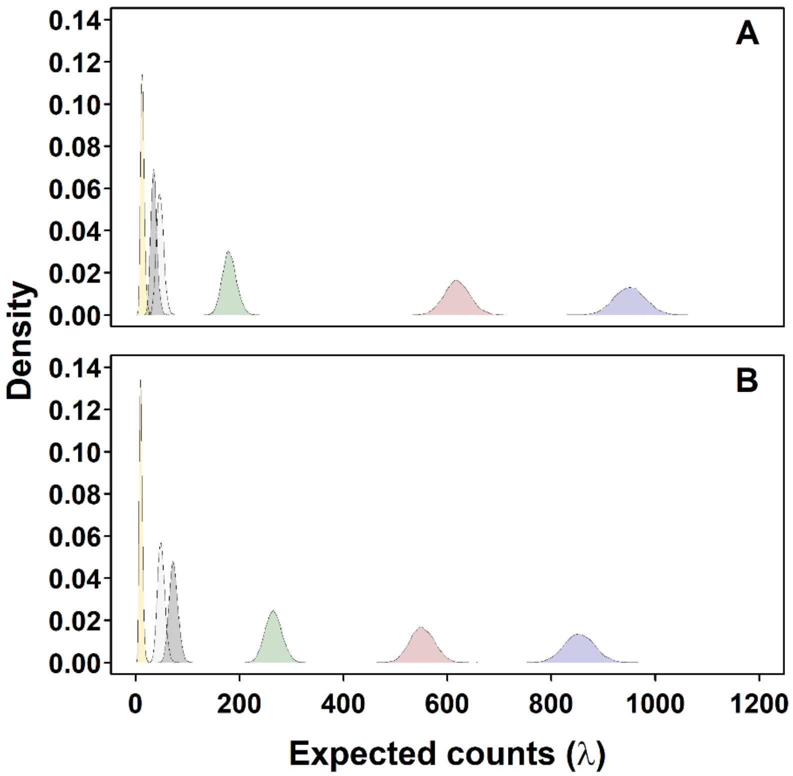

Supplementary Figure 118. Posterior densities of the expected counts (λ) for where consumers buy seafood (Red= "Fish market", Blue= "Large retail", Green= "Local market", Dark gray= "Not buy", Yellow= "Online", and Light gray = "Other") per sex (from A to B: "Female", and "Male"), extracted after 10^4 MCMC draws.

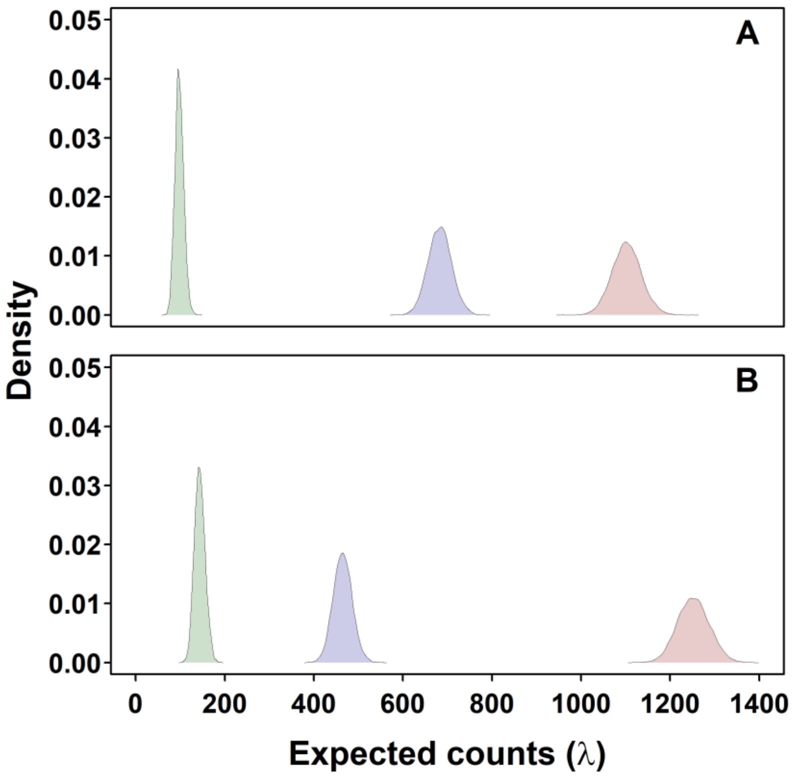

Supplementary Figure 119. Posterior densities of the expected counts (λ) for the degree of seafood processing consumers prefer (Red= "Fresh", Blue= "Frozen", and Green= "Processed") per sex (from A to B: "Female", and "Male"), extracted after 10^4 MCMC draws.

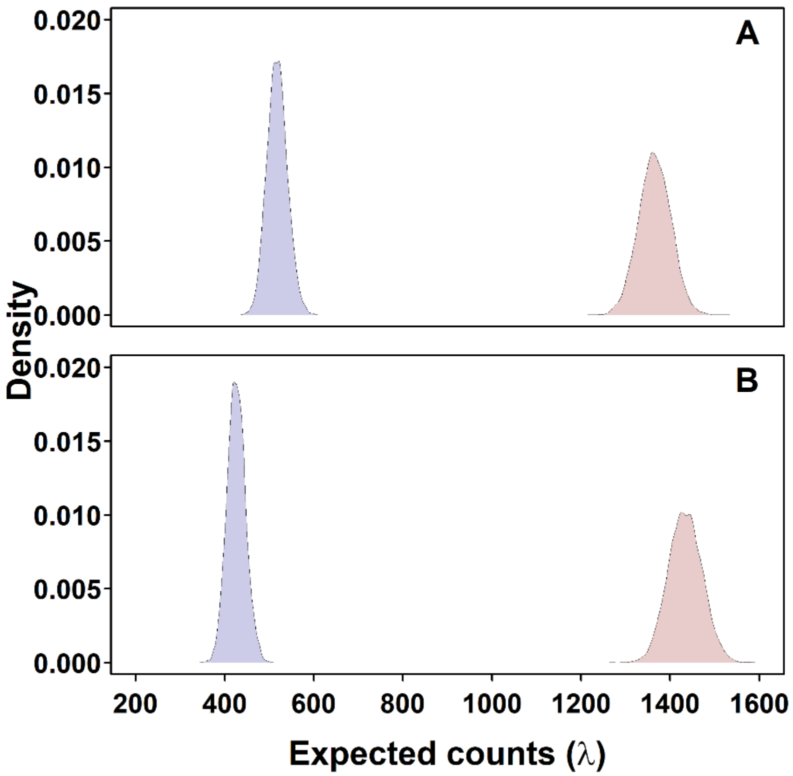

Supplementary Figure 120. Posterior densities of the expected counts (λ) for being informed on seafood origin (Red= "Yes" and Blue= "No") per sex (from A to B: "Female", and "Male"), extracted after 10^4 MCMC draws.

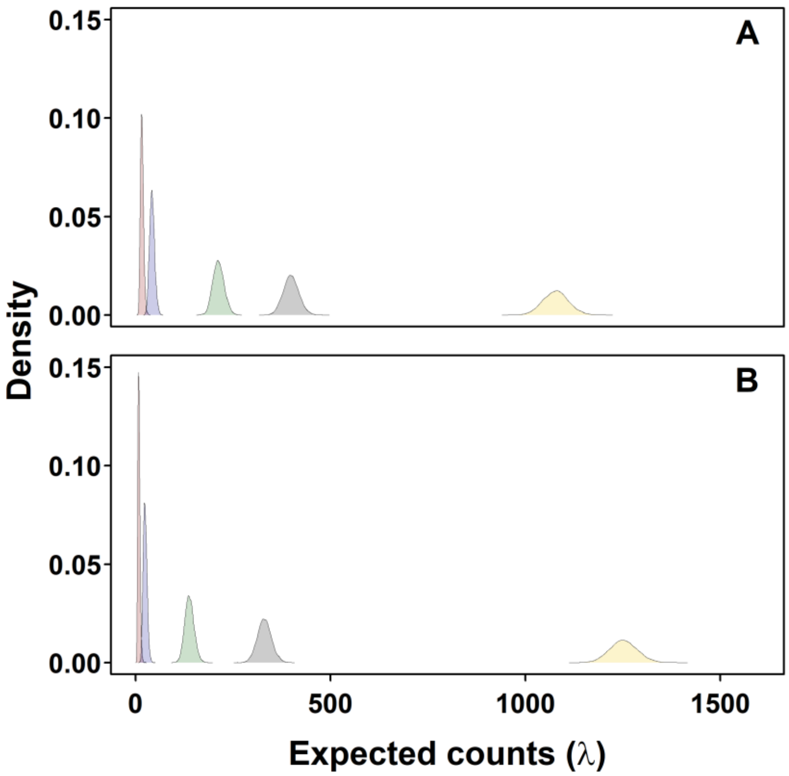

Supplementary Figure 121. Posterior densities of the expected counts (λ) for level of consumers' interest in seafood traceability (Red= 1, Blue= 2, Green= 3, Dark gray= 4, and Yellow= 5) per sex (from A to B: "Female", and "Male"), extracted after 10^4 MCMC draws.

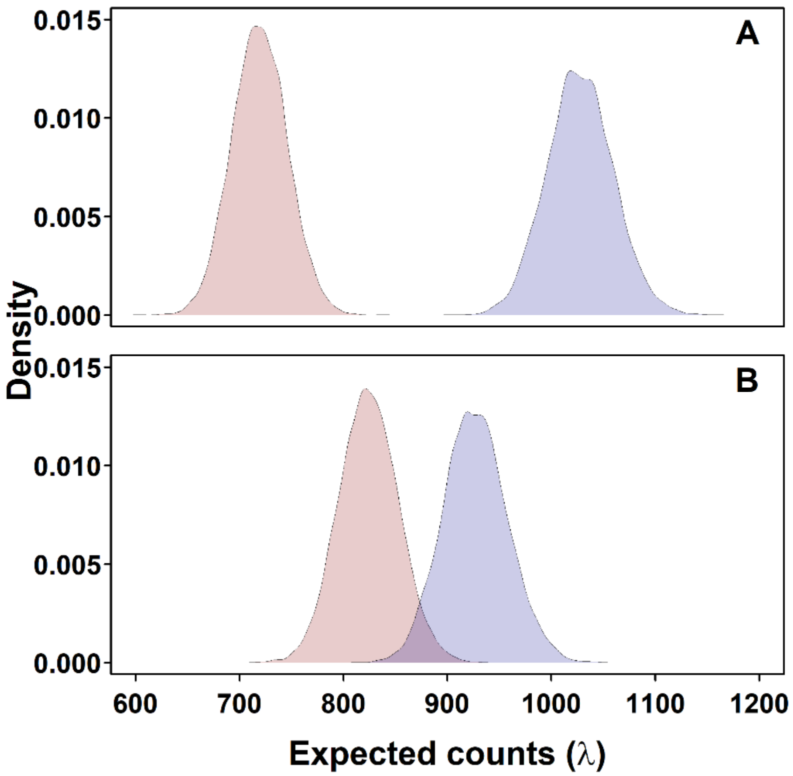

Supplementary Figure 122. Posterior densities of the expected counts (λ) for the consumption of Italian-farmed sea bass (Red= “Yes”, and Blue= “No”) per sex (from A to B: “Female”, and “Male”), extracted after 10^4 MCMC draws.

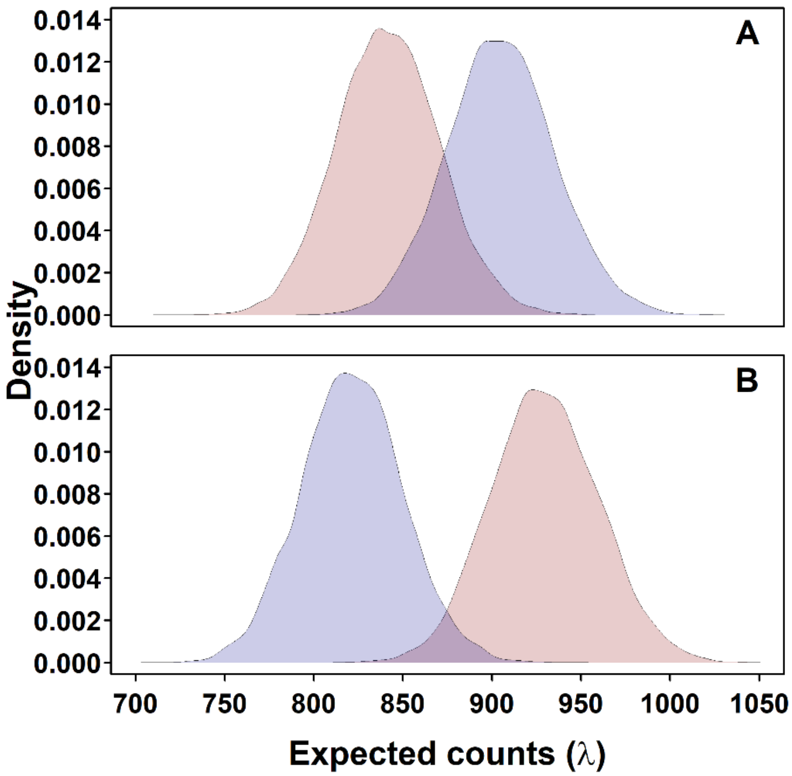

Supplementary Figure 123. Posterior densities of the expected counts (λ) for the consumption of striped venus clams (Red= “Yes”, and Blue= “No”) per sex (from A to B: “Female”, and “Male”), extracted after 10^4 MCMC draws.

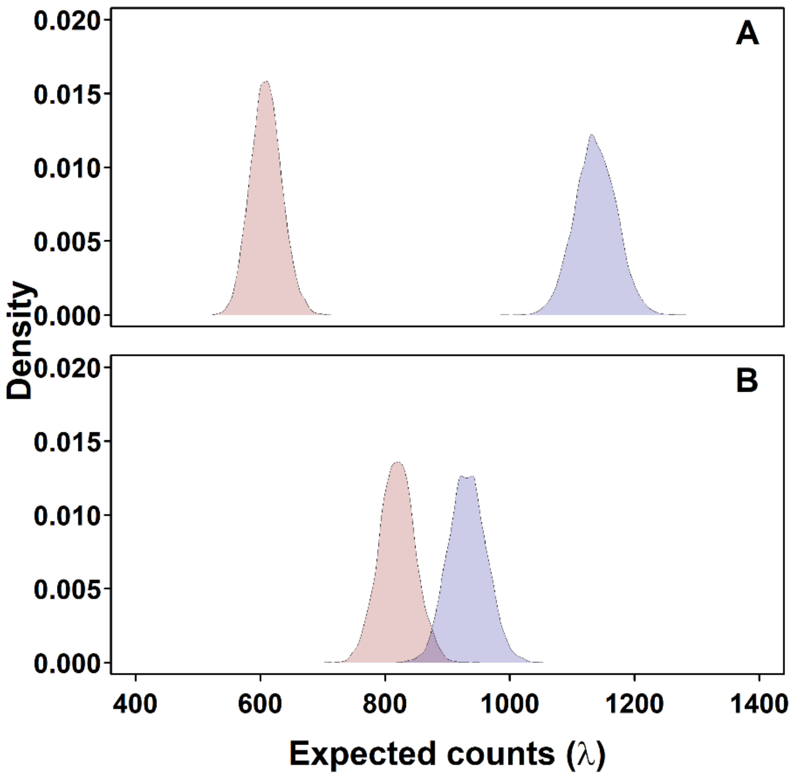

Supplementary Figure 124. Posterior densities of the expected counts (λ) for the consumption of giant red shrimp (Red= "Yes", and Blue= "No") per sex (from A to B: "Female", and "Male"), extracted after 10^4 MCMC draws.

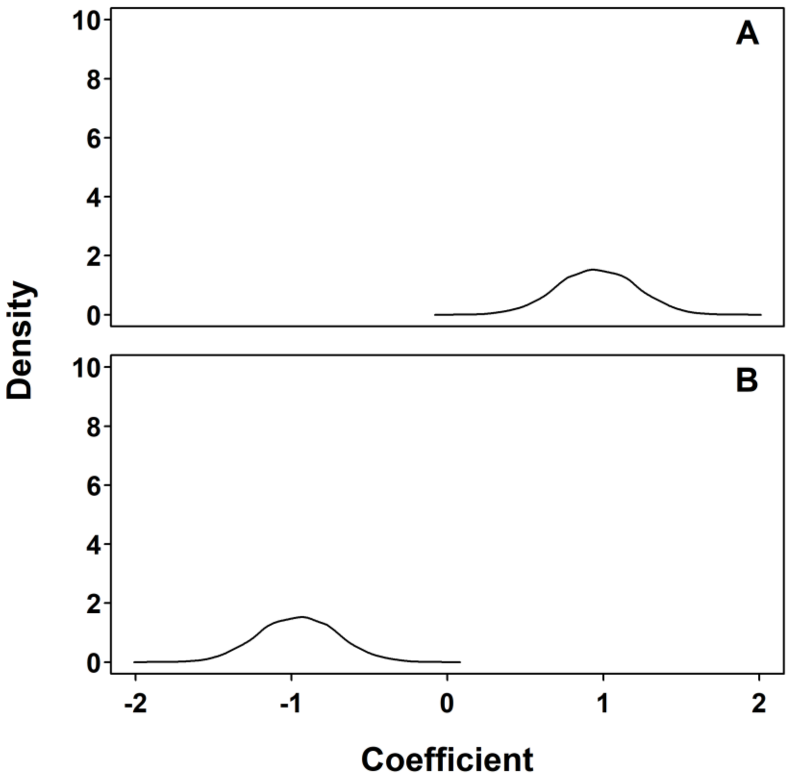

Supplementary Figure 125. Posterior densities of the ANOVA coefficients for the consumers' WTP for giant red shrimp per sex (from A to B: "Female", and "Male"), extracted after 10^4 MCMC draws.

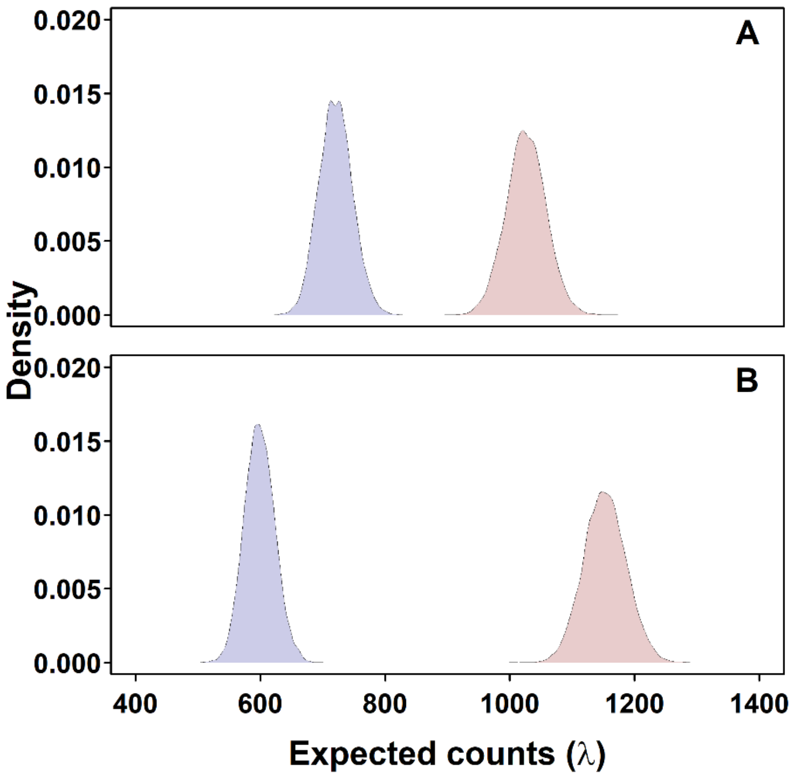

Supplementary Figure 126. Posterior densities of the expected counts (λ) for the consumption of processed albacore tuna (Red= “Yes”, and Blue= “No”) per sex (from A to B: “Female”, and “Male”), extracted after 10^4 MCMC draws.

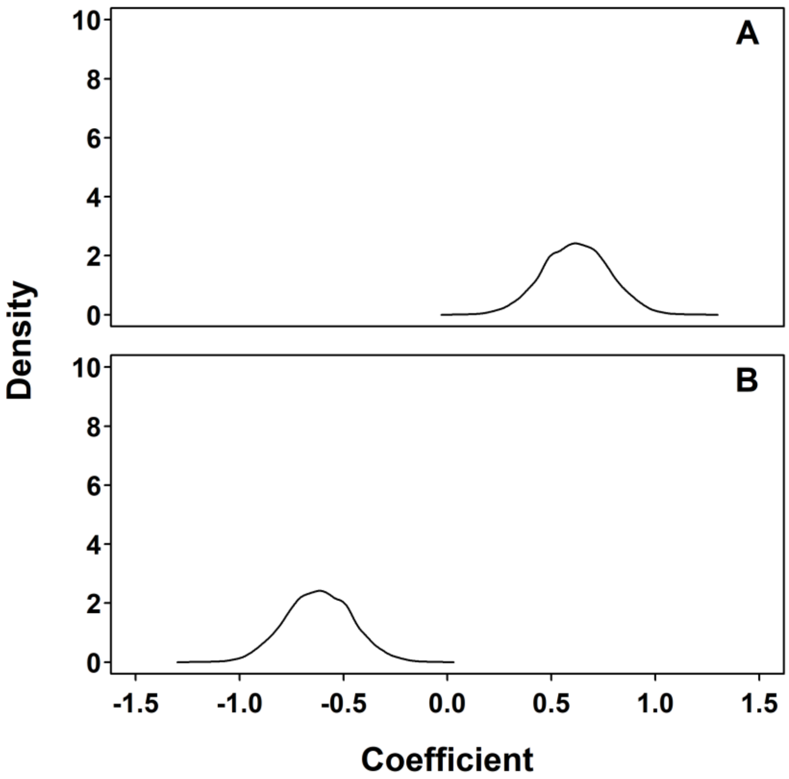

Supplementary Figure 127. Posterior densities of the ANOVA coefficients for the consumers' WTP for processed albacore tuna per sex (from A to B: "Female", and "Male"), extracted after 10^4 MCMC draws.

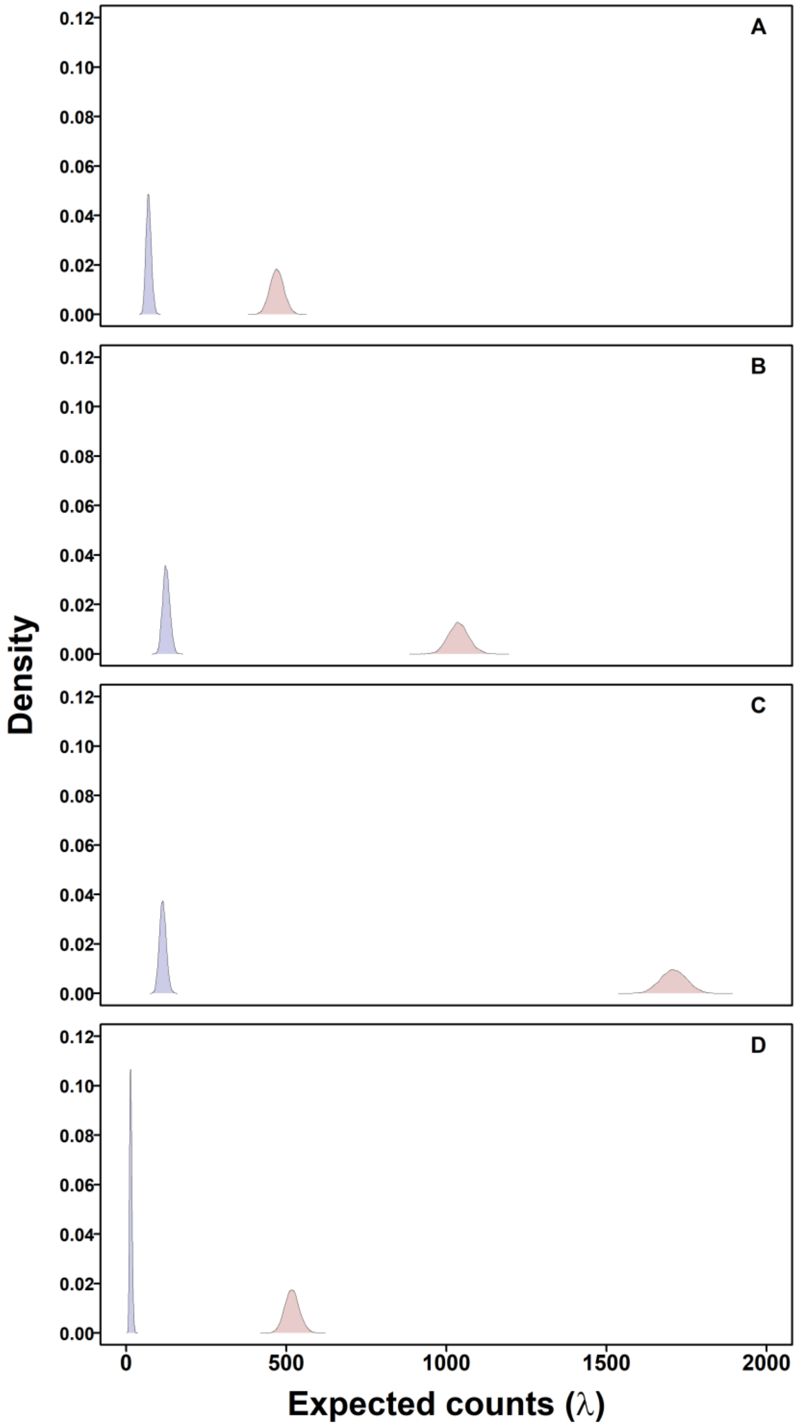

Supplementary Figure 128. Posterior densities of the expected counts (λ) for the consumption of seafood (Red= "Yes", and Blue= "No") per age range (from A to D: 18–25, 26–40, 41–65, and 66+), extracted after 10^4 MCMC draws.

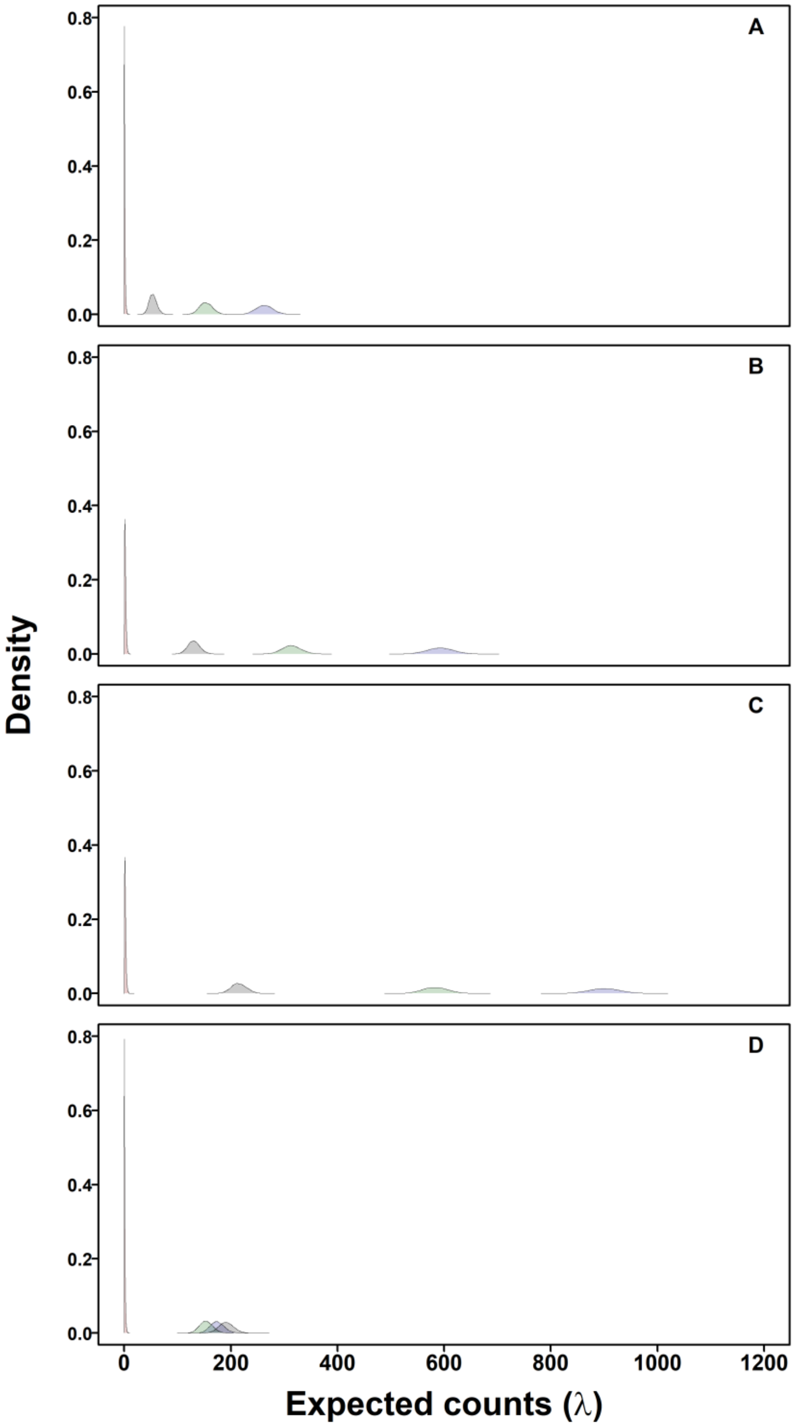

Supplementary Figure 129. Posterior densities of the expected counts (λ) for monthly seafood consumption rate (Red= 0, Blue= 1–5, Green= 6–10, and Dark gray= 10+) per age range (from A to D: 18–25, 26–40, 41–65, and 66+), extracted after 10^4 MCMC draws.

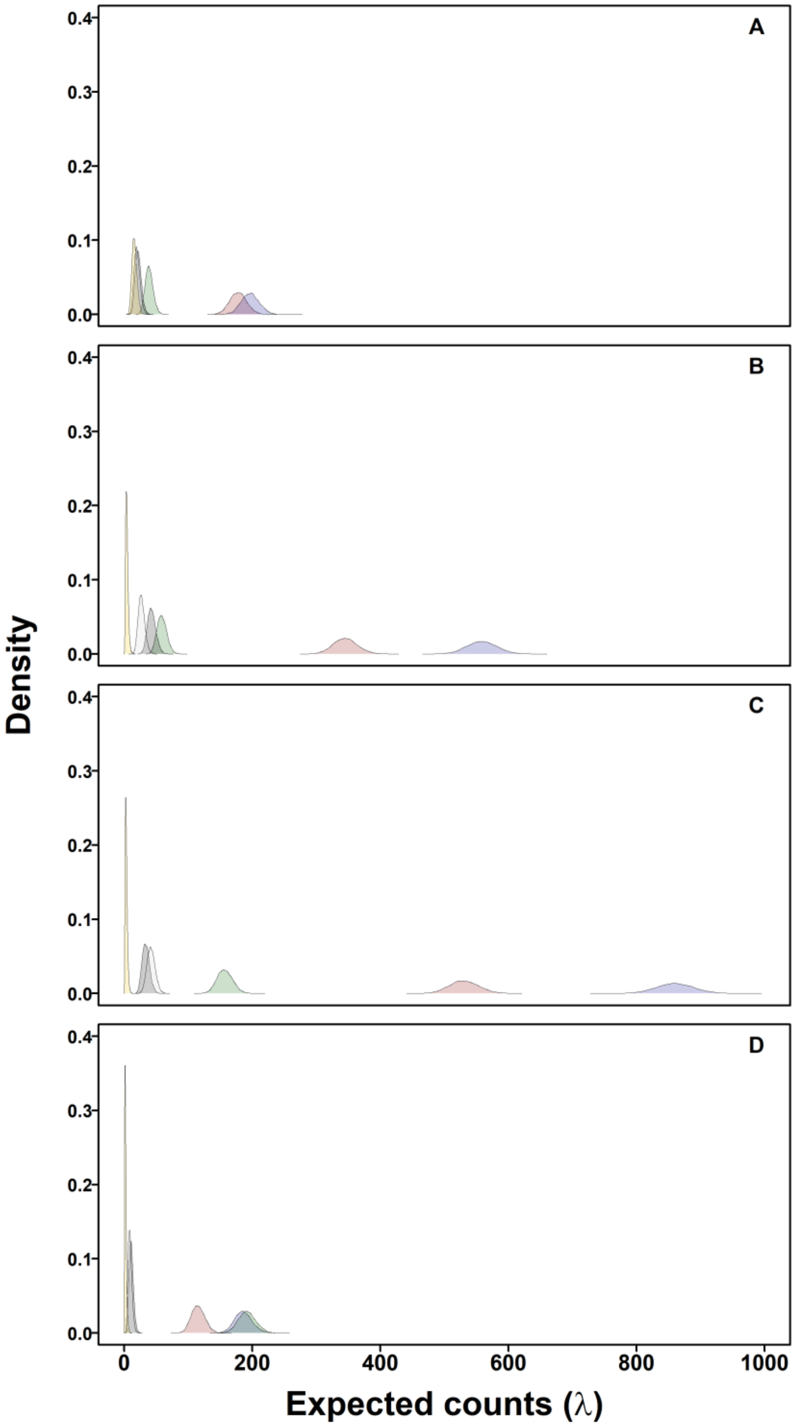

Supplementary Figure 130. Posterior densities of the expected counts (λ) for where consumers buy seafood (Red= "Fish market", Blue= "Large retail", Green= "Local market", Dark gray= "Not buy", Yellow= "Online", and Light gray = "Other") per age range (from A to D: 18–25, 26–40, 41–65, and 66+), extracted after 10^4 MCMC draws.

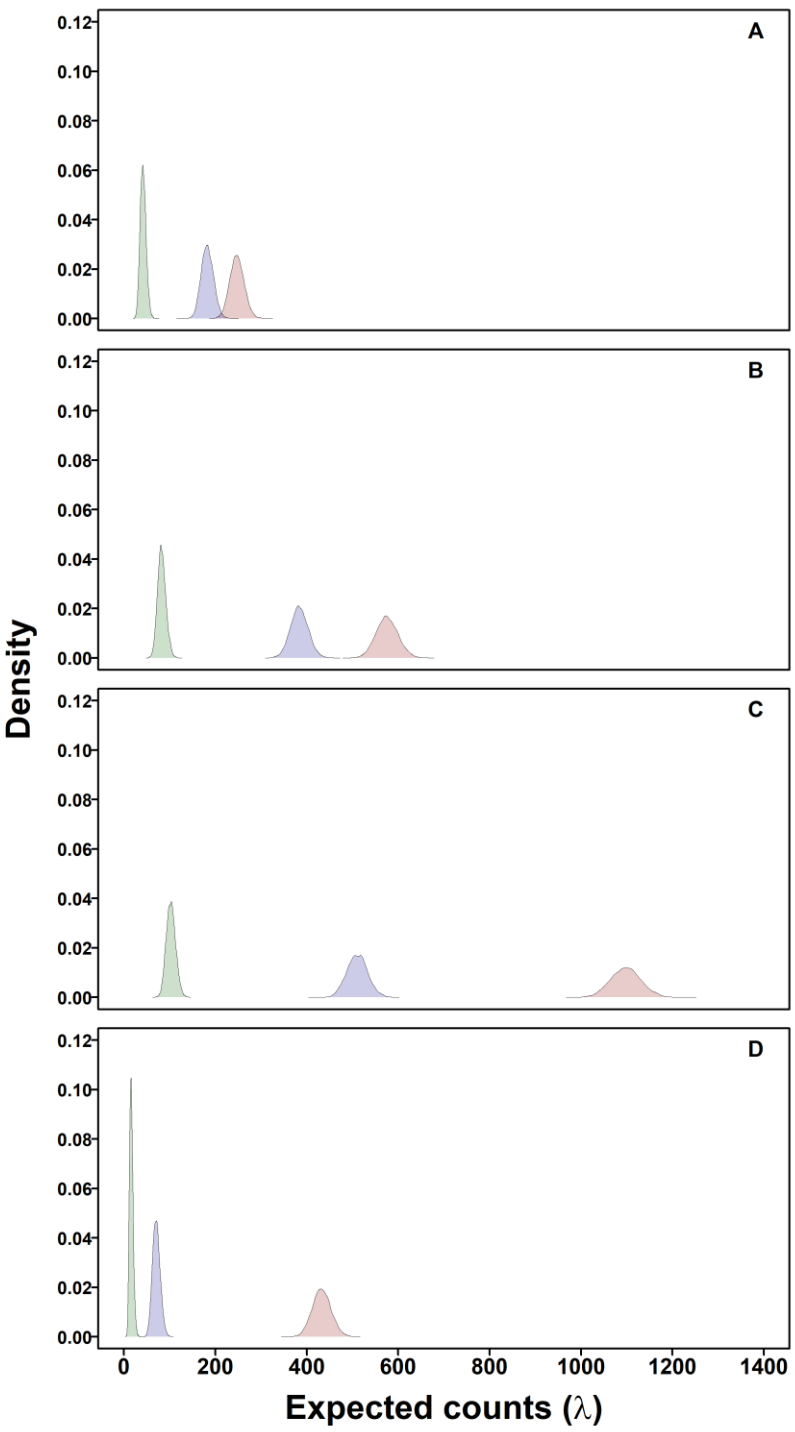

Supplementary Figure 131. Posterior densities of the expected counts (λ) for the degree of seafood processing consumers prefer (Red= "Fresh", Blue= "Frozen", and Green= "Processed") per age range (from A to D: 18–25, 26–40, 41–65, and 66+), extracted after 10^4 MCMC draws.

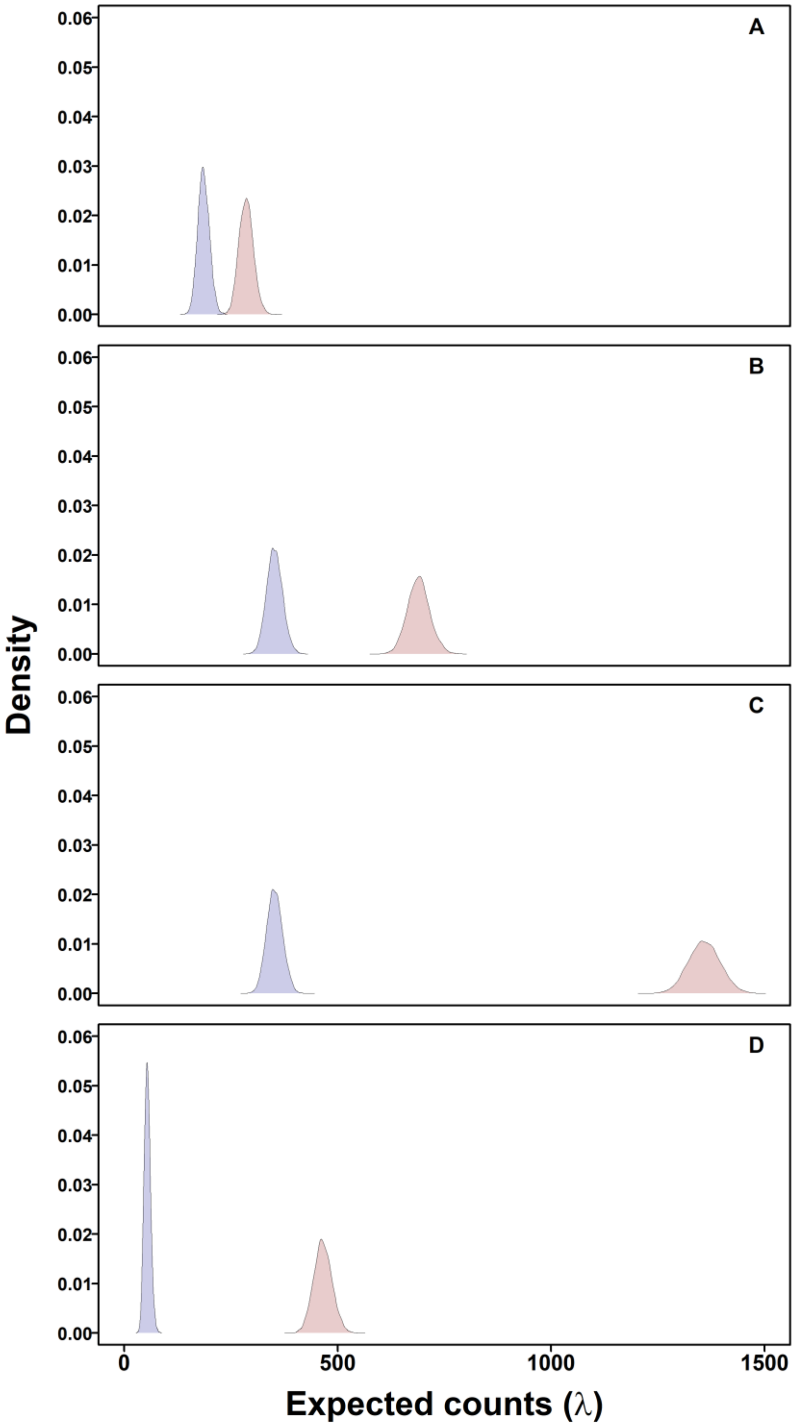

Supplementary Figure 132. Posterior densities of the expected counts (λ) for being informed on seafood origin (Red= "Yes" and Blue= "No") per age range (from A to D: 18–25, 26–40, 41–65, and 66+), extracted after 10^4 MCMC draws.

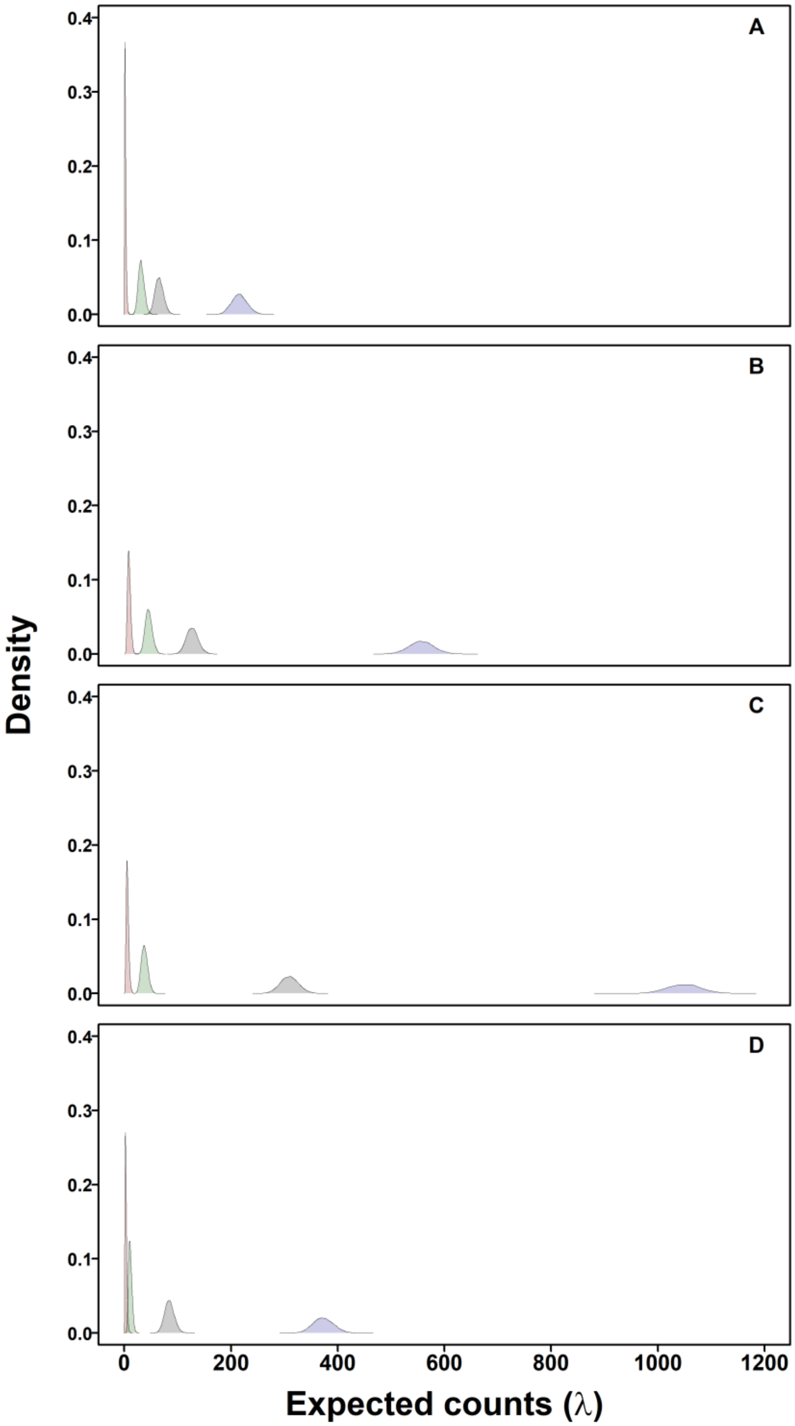

Supplementary Figure 133. Posterior densities of the expected counts (λ) for sources of information on seafood origin (Red= "Ads", Blue= "Label", Green= "Other", and Dark gray= "Retailer") per age range (from A to D: 18–25, 26–40, 41–65, and 66+), extracted after 10^4 MCMC draws.

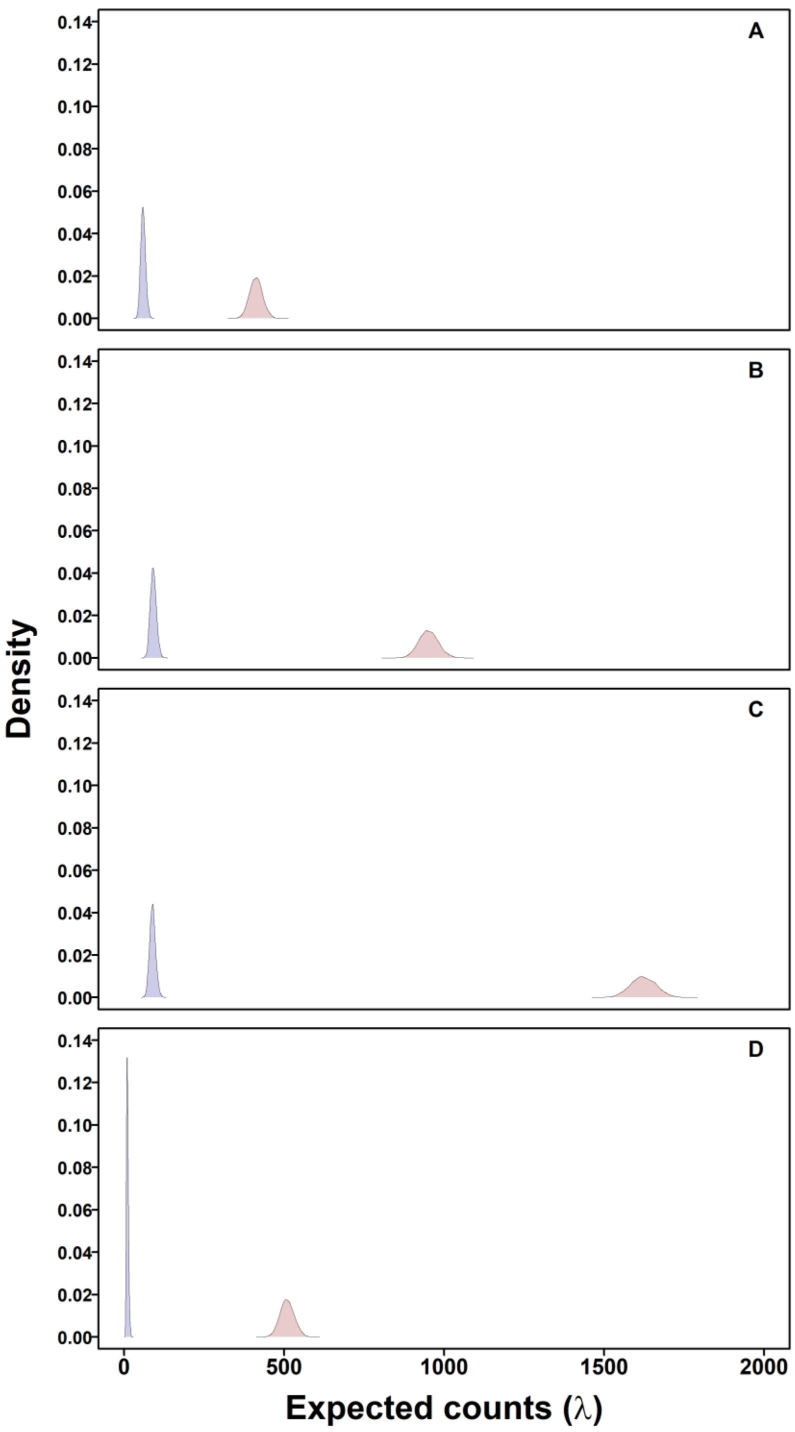

Supplementary Figure 134. Posterior densities of the expected counts (λ) for interest on seafood traceability (Red= "Yes" and Blue= "No") per age range (from A to D: 18–25, 26–40, 41–65, and 66+), extracted after 10^4 MCMC draws.

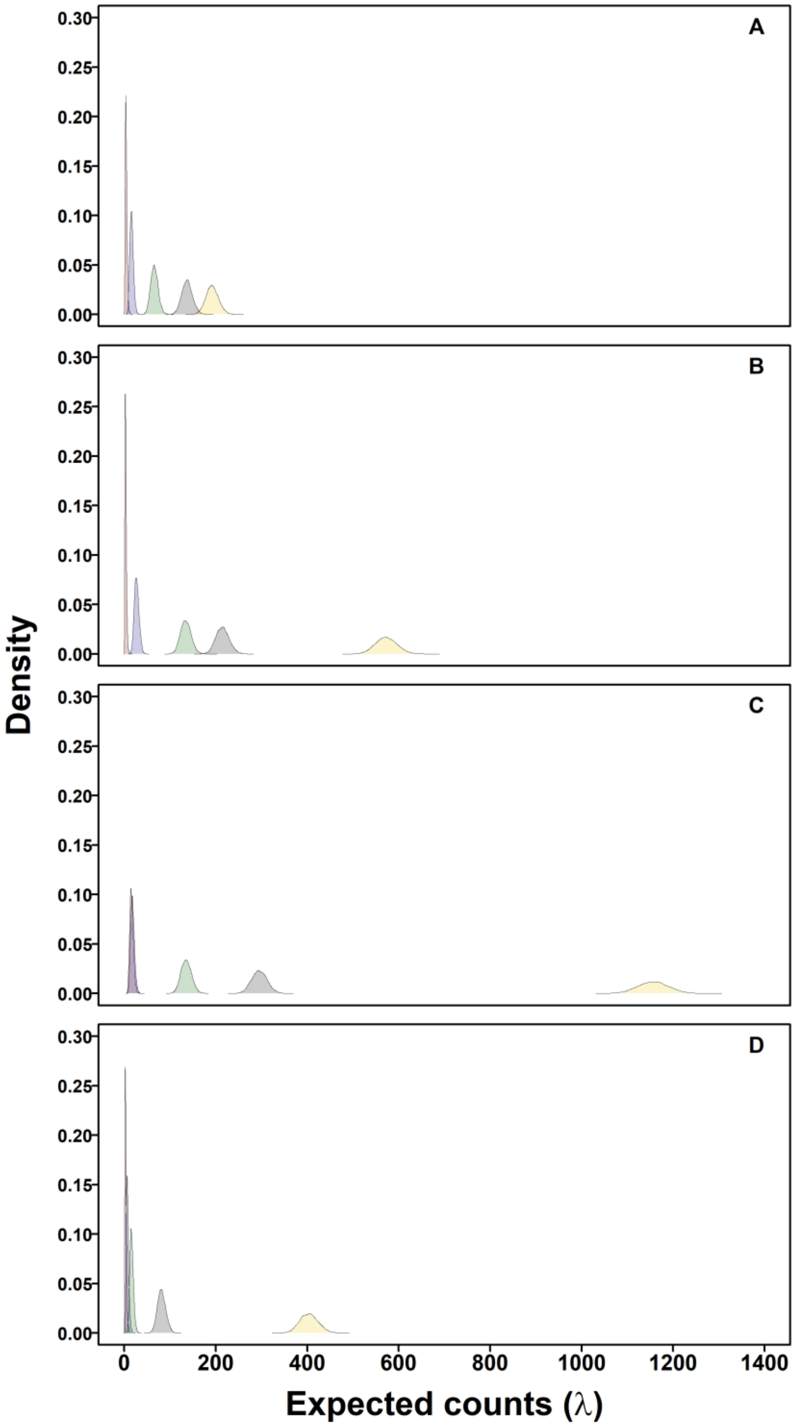

Supplementary Figure 135. Posterior densities of the expected counts (λ) for level of consumers' interest in seafood traceability (Red= 1, Blue= 2, Green= 3, Dark gray= 4, and Yellow= 5) per age range (from A to D: 18–25, 26–40, 41–65, and 66+), extracted after 10^4 MCMC draws.

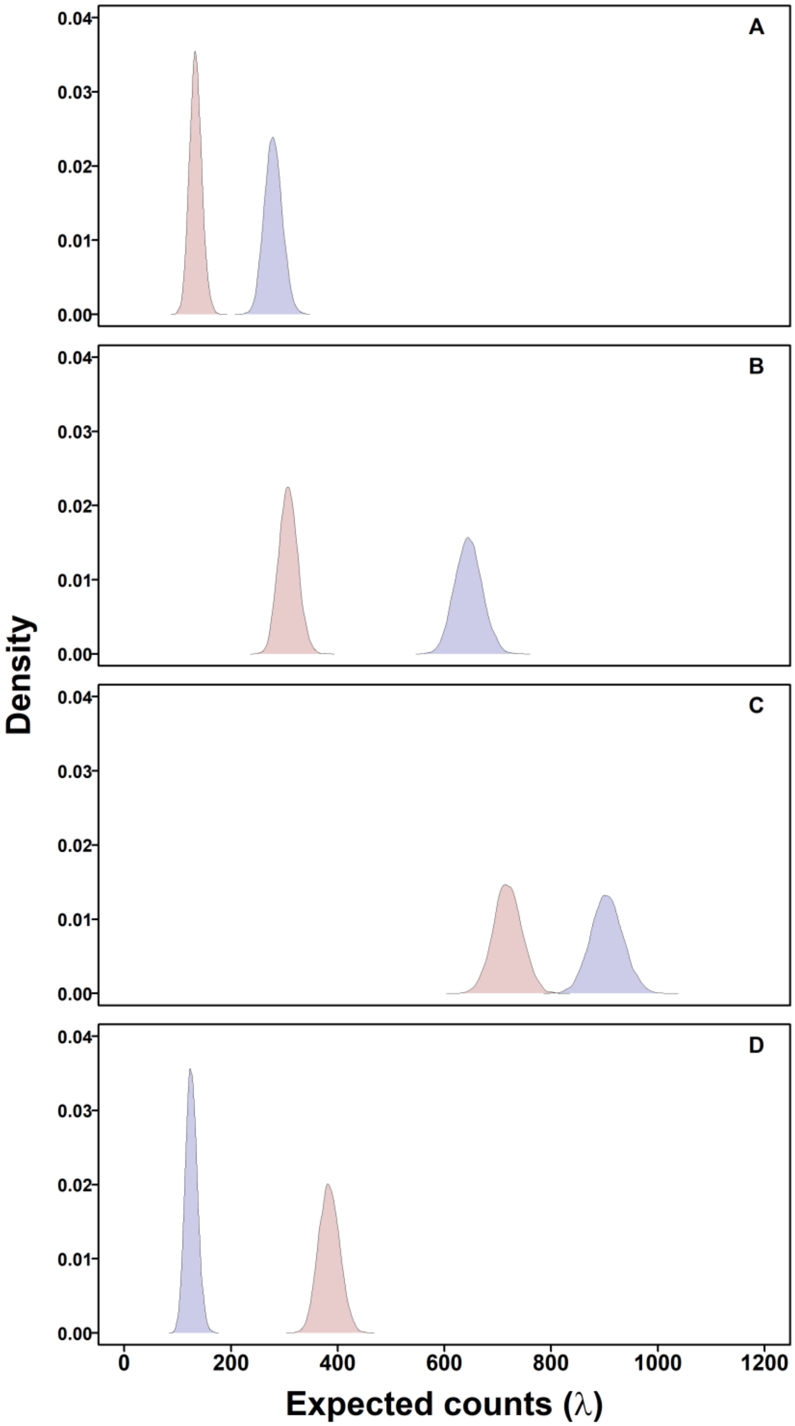

Supplementary Figure 136. Posterior densities of the expected counts (λ) for the consumption of Italian-farmed sea bass (Red= "Yes", and Blue= "No") per age range (from A to D: 18–25, 26–40, 41–65, and 66+), extracted after 10^4 MCMC draws.

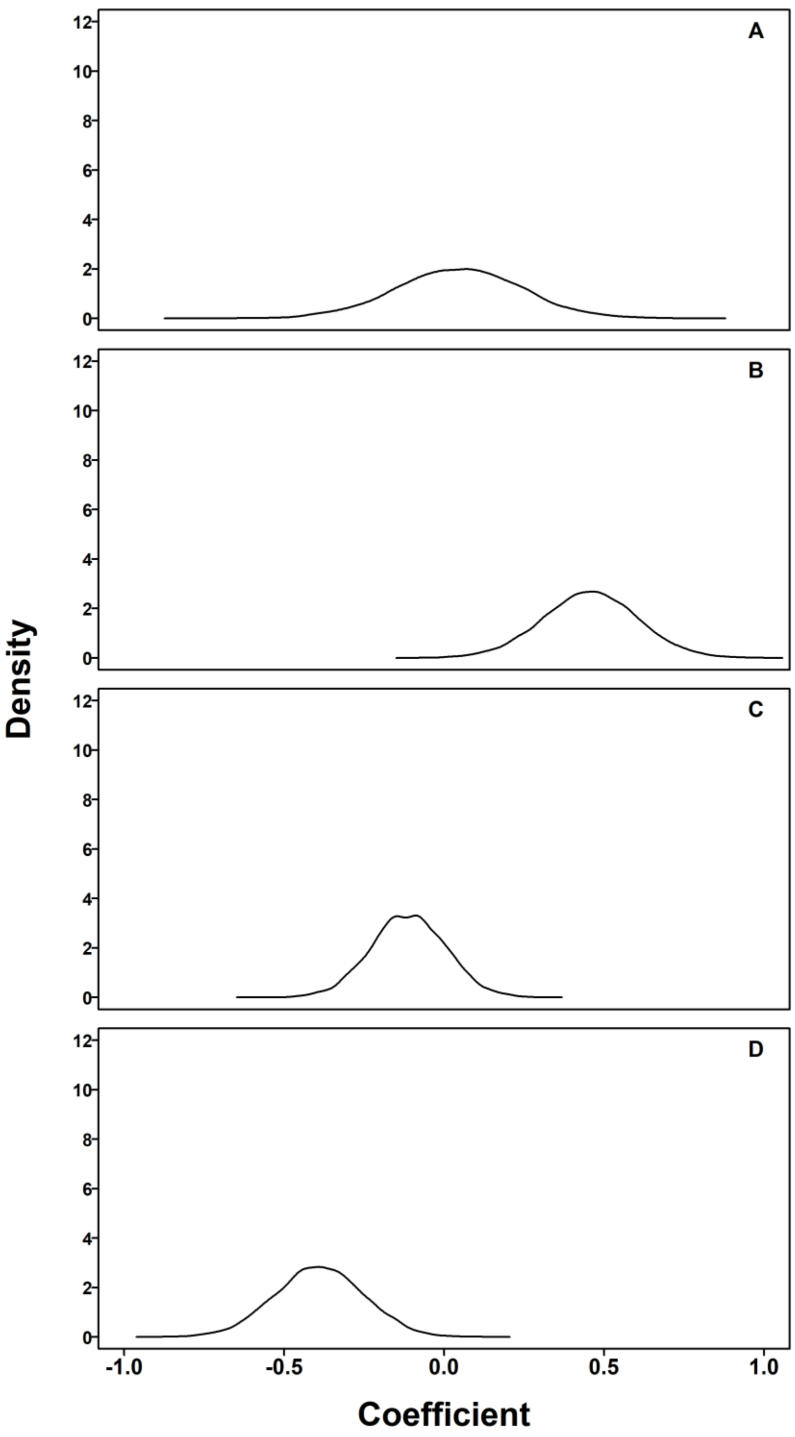

Supplementary Figure 137. Posterior densities of the ANOVA coefficients for the consumers' WTP for Italian-farmed sea bass per age range (from A to D: 18–25, 26–40, 41–65, and 66+), extracted after 10^4 MCMC draws.

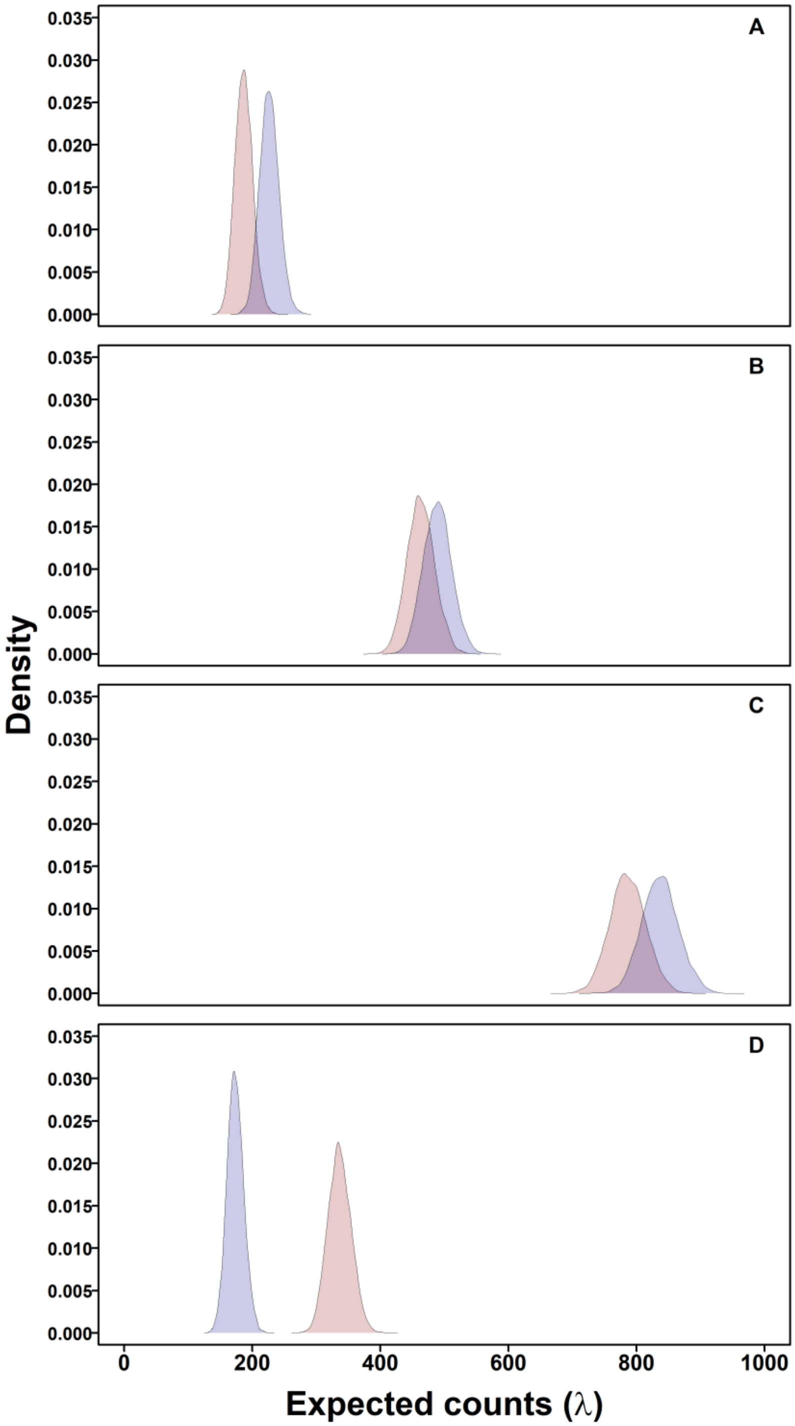

Supplementary Figure 138. Posterior densities of the expected counts (λ) for the consumption of striped venus clams (Red= “Yes”, and Blue= “No”) per age range (from A to D: 18–25, 26–40, 41–65, and 66+), extracted after 10^4 MCMC draws.

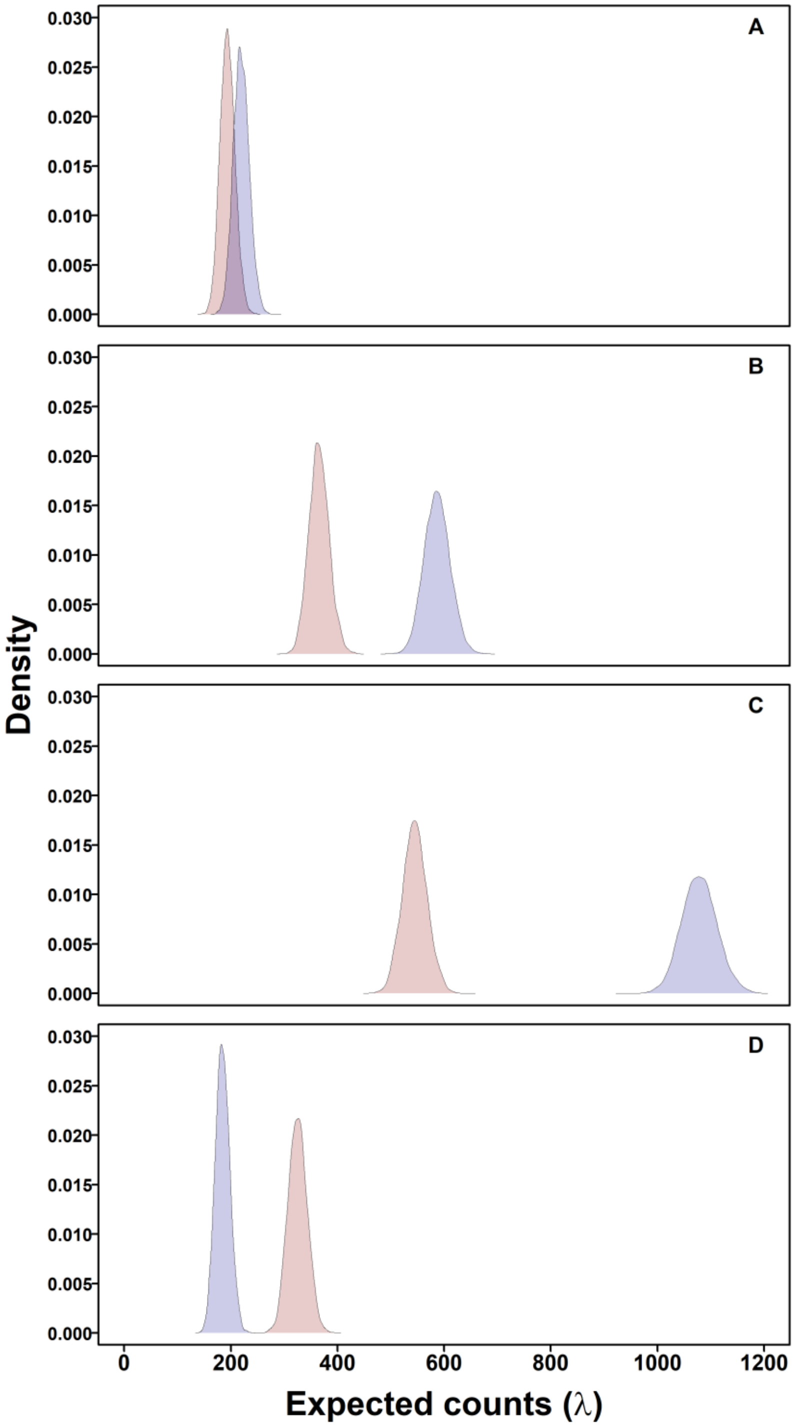

Supplementary Figure 139. Posterior densities of the expected counts (λ) for the consumption of giant red shrimp (Red= "Yes", and Blue= "No") per age range (from A to D: 18–25, 26–40, 41–65, and 66+), extracted after 10^4 MCMC draws.

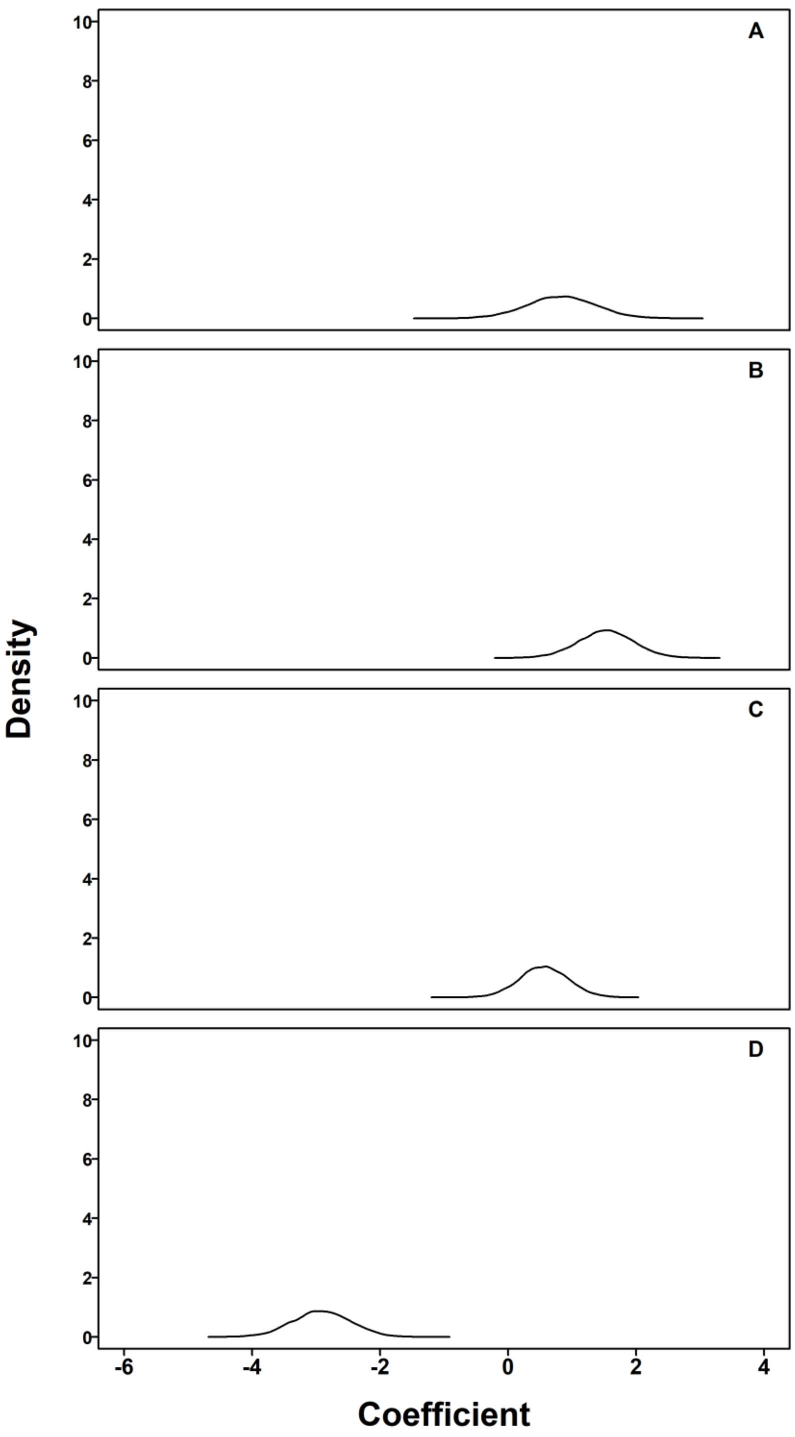

Supplementary Figure 140. Posterior densities of the ANOVA coefficients for the consumers' WTP for giant red shrimp per age range (from A to D: 18–25, 26–40, 41–65, and 66+), extracted after 10^4 MCMC draws.

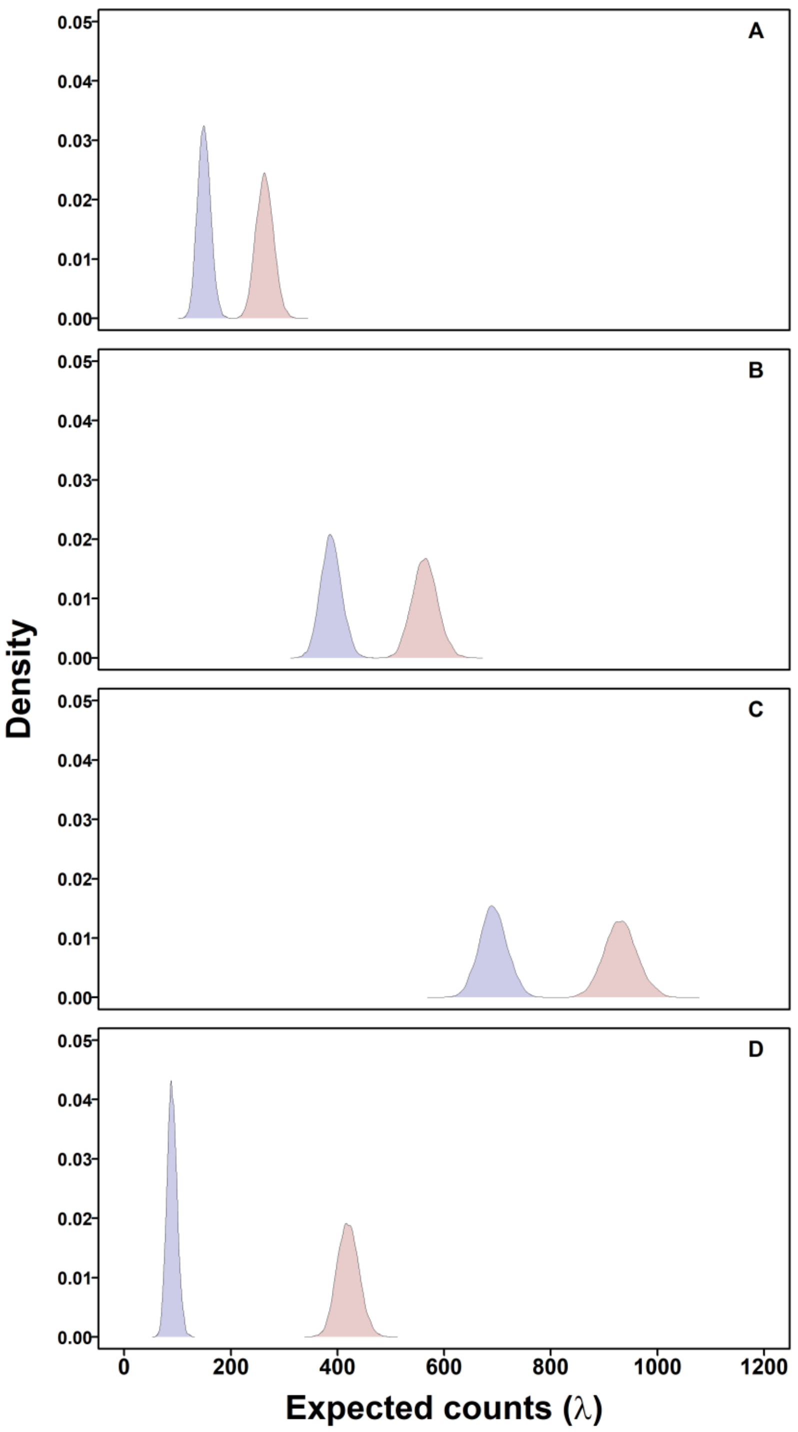

Supplementary Figure 141. Posterior densities of the expected counts (λ) for the consumption of processed albacore tuna (Red= "Yes", and Blue= "No") per age range (from A to D: 18–25, 26–40, 41–65, and 66+), extracted after 10^4 MCMC draws.

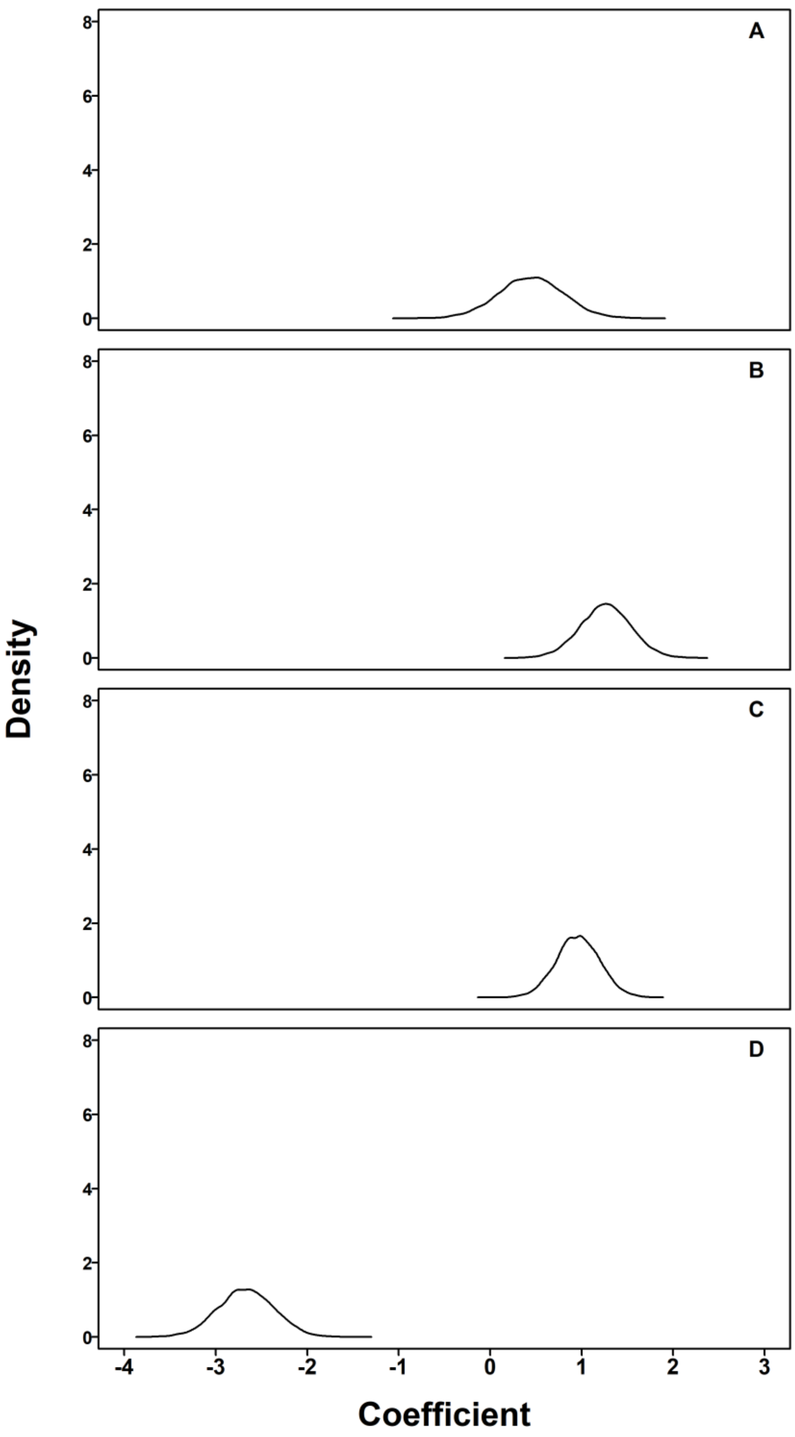

Supplementary Figure 142. Posterior densities of the ANOVA coefficients for the consumers' WTP for processed albacore tuna per age range (from A to D: 18–25, 26–40, 41–65, and 66+), extracted after 10^4 MCMC draws.

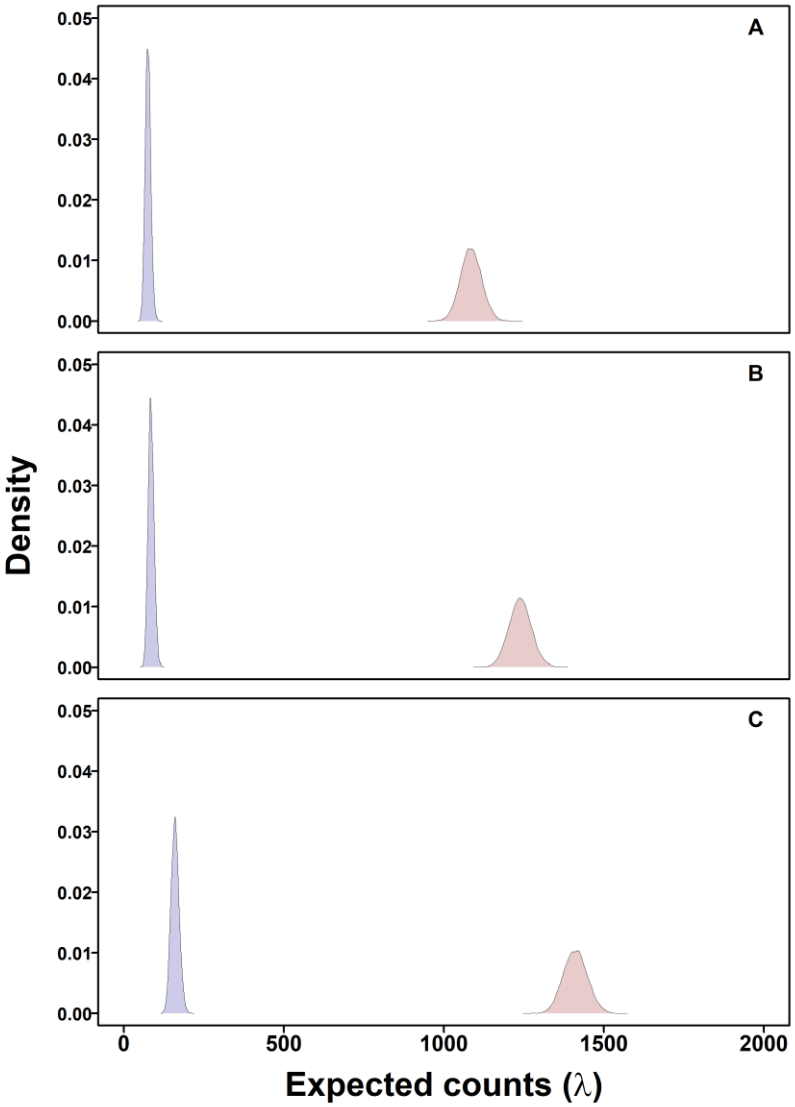

Supplementary Figure 143. Posterior densities of the expected counts (λ) for the consumption of seafood (Red= "Yes", and Blue= "No") per province (from A to C: "South", "Central", and "North"), extracted after 10^4 MCMC draws.

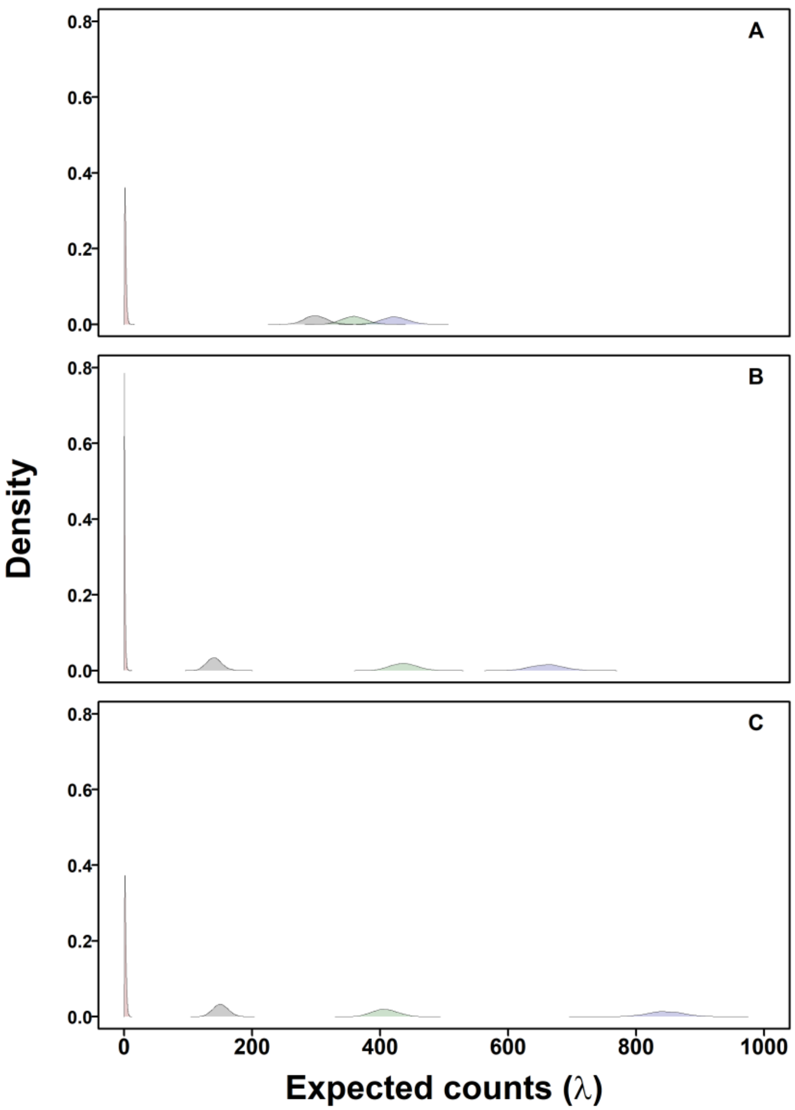

Supplementary Figure 144. Posterior densities of the expected counts (λ) for monthly seafood consumption rate (Red= 0, Blue= 1–5, Green= 6–10, and Dark gray= 10+) per province (from A to C: "South", "Central", and "North"), extracted after 10^4 MCMC draws.

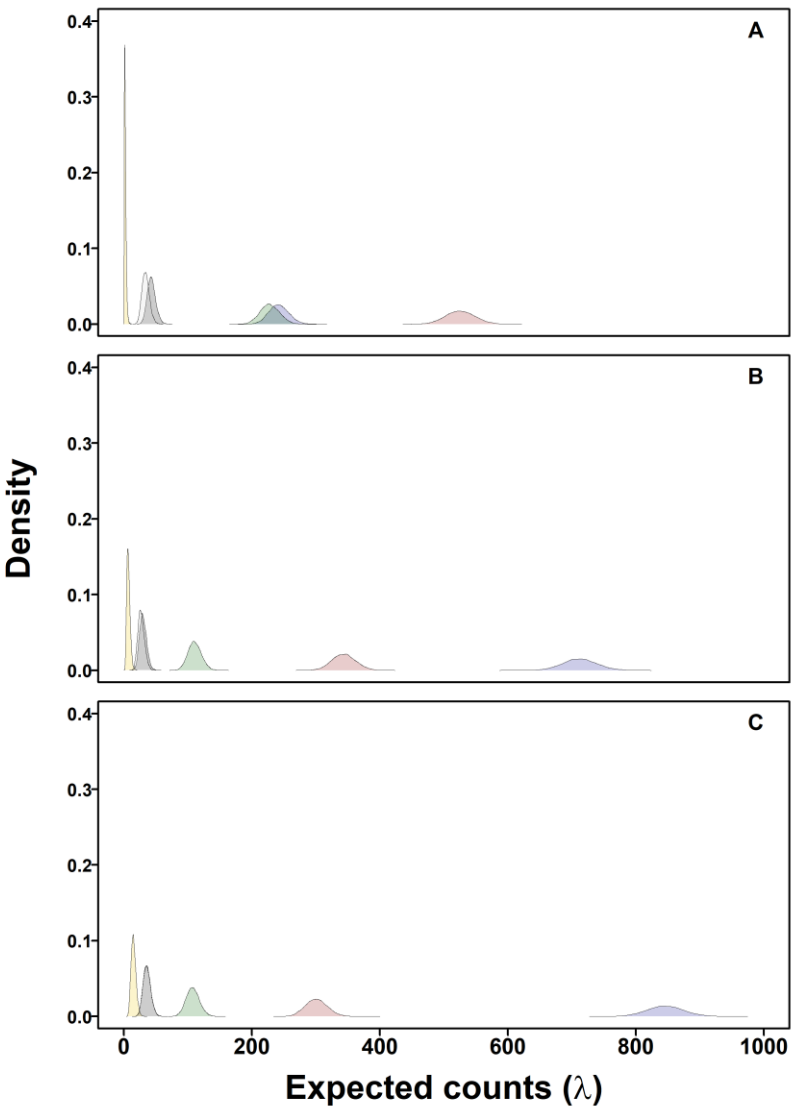

Supplementary Figure 145. Posterior densities of the expected counts (λ) for where consumers buy seafood (Red= "Fish market", Blue= "Large retail", Green= "Local market", Dark gray= "Not buy", Yellow= "Online", and Light gray = "Other") per province (from A to C: "South", "Central", and "North"), extracted after 10^4 MCMC draws.

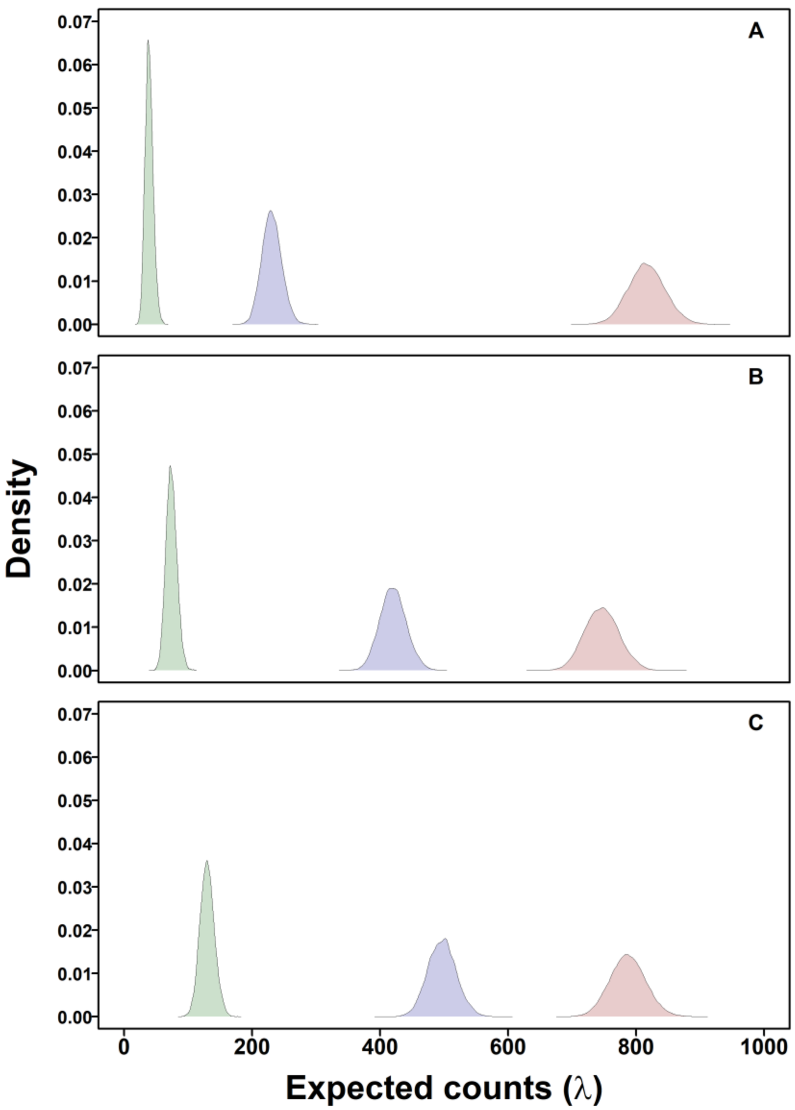

Supplementary Figure 146. Posterior densities of the expected counts (λ) for the degree of seafood processing consumers prefer (Red= "Fresh", Blue= "Frozen", and Green= "Processed") per province (from A to C: "South", "Central", and "North"), extracted after 10^4 MCMC draws.

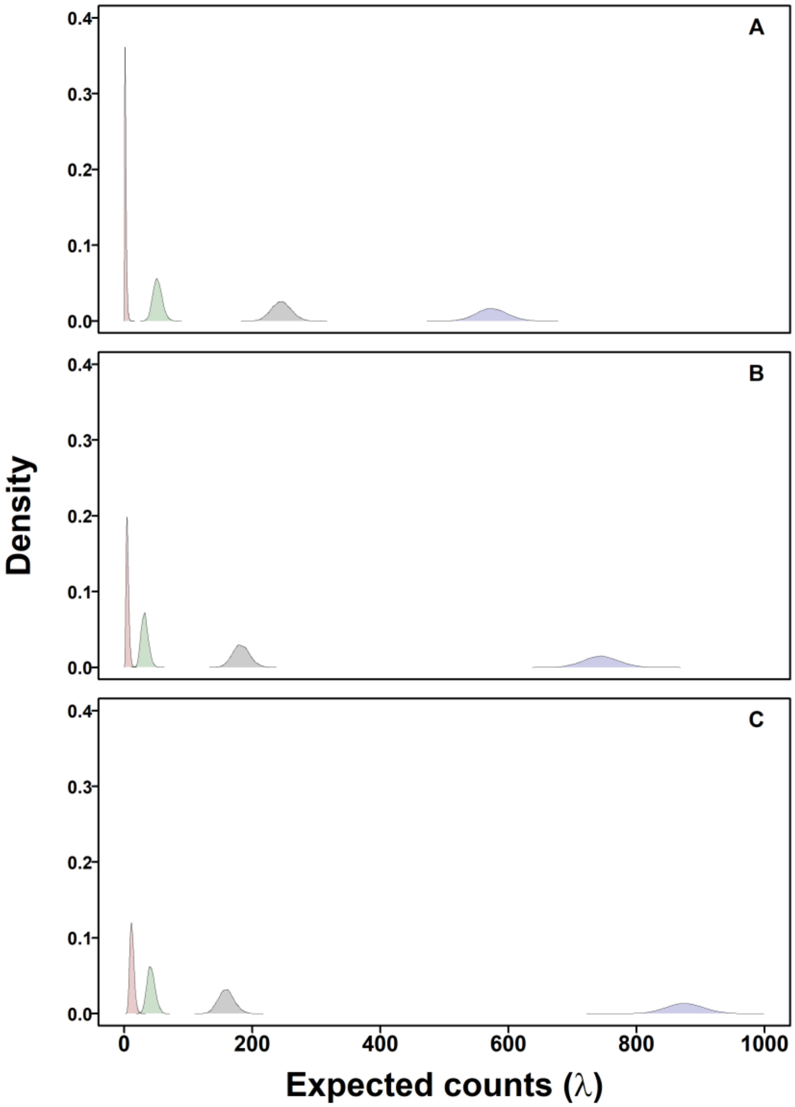

Supplementary Figure 147. Posterior densities of the expected counts (λ) for sources of information on seafood origin (Red= "Ads", Blue= "Label", Green= "Other", and Dark gray= "Retailer") per province (from A to C: "South", "Central", and "North"), extracted after 10^4 MCMC draws.

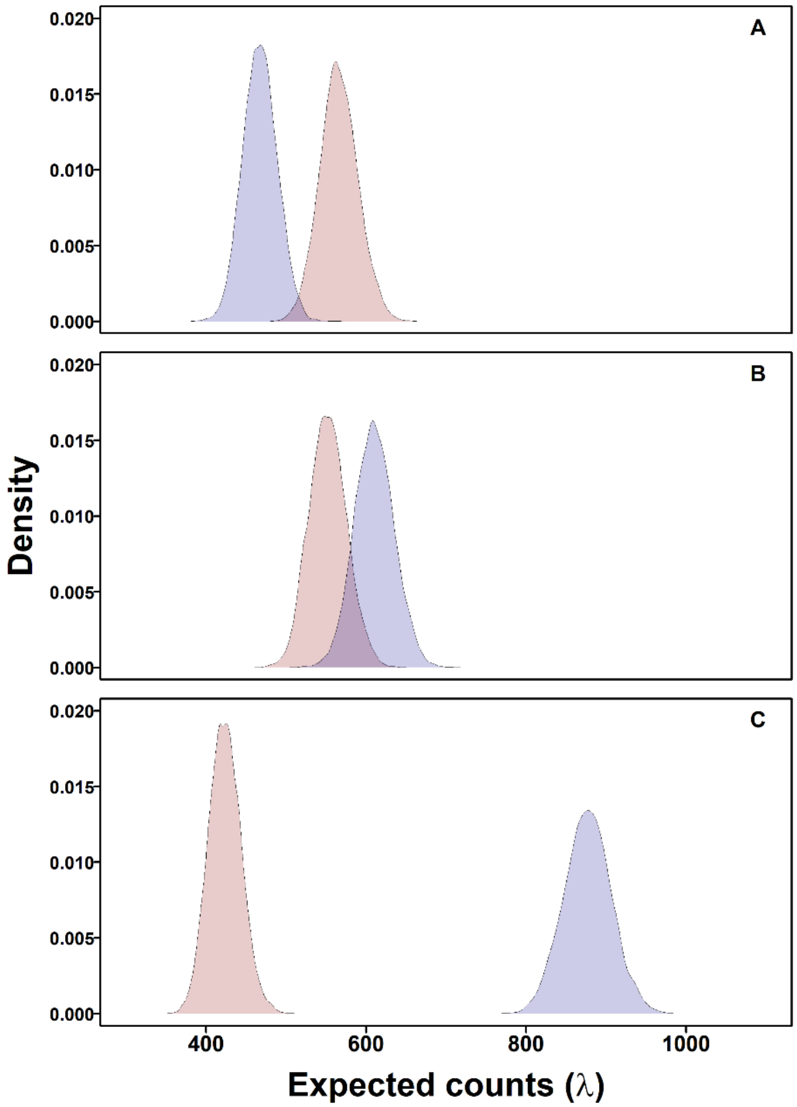

Supplementary Figure 148. Posterior densities of the expected counts (λ) for the consumption of Italian-farmed sea bass (Red= “Yes”, and Blue= “No”) per province (from A to C: “South”, “Central”, and “North”), extracted after 10^4 MCMC draws.

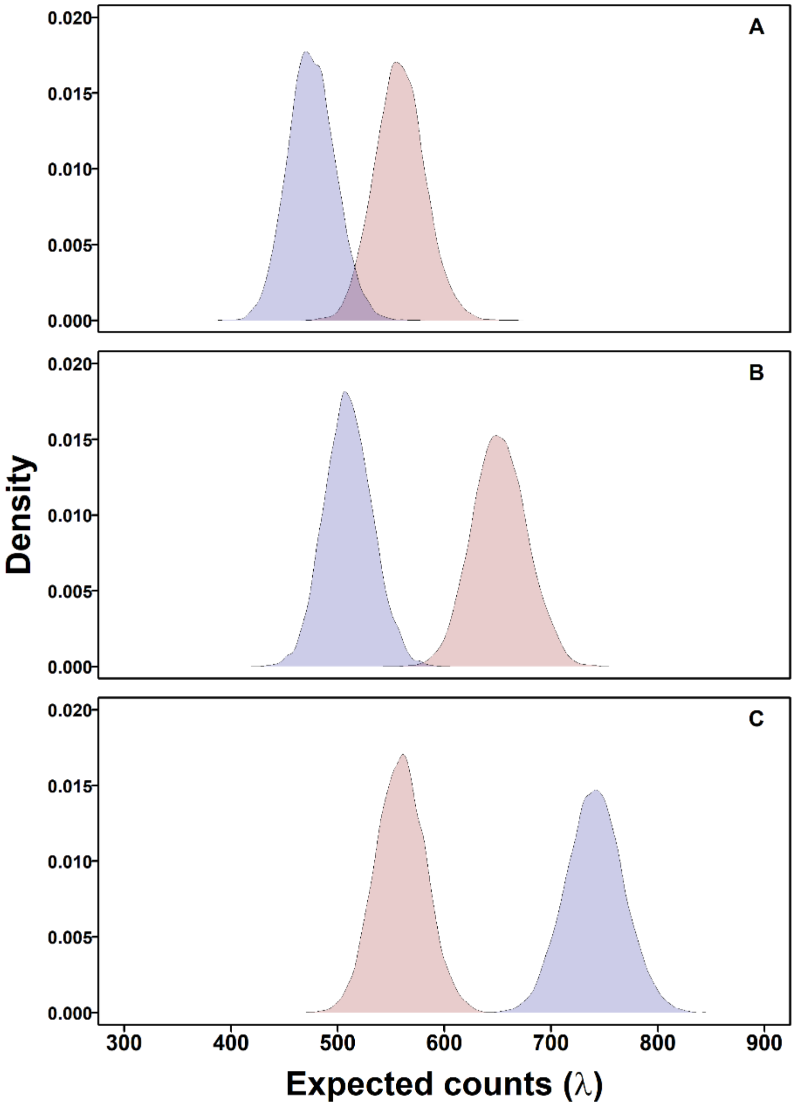

Supplementary Figure 149. Posterior densities of the expected counts (λ) for the consumption of striped venus clams (Red= "Yes", and Blue= "No") per province (from A to C: "South", "Central", and "North"), extracted after 10^4 MCMC draws.

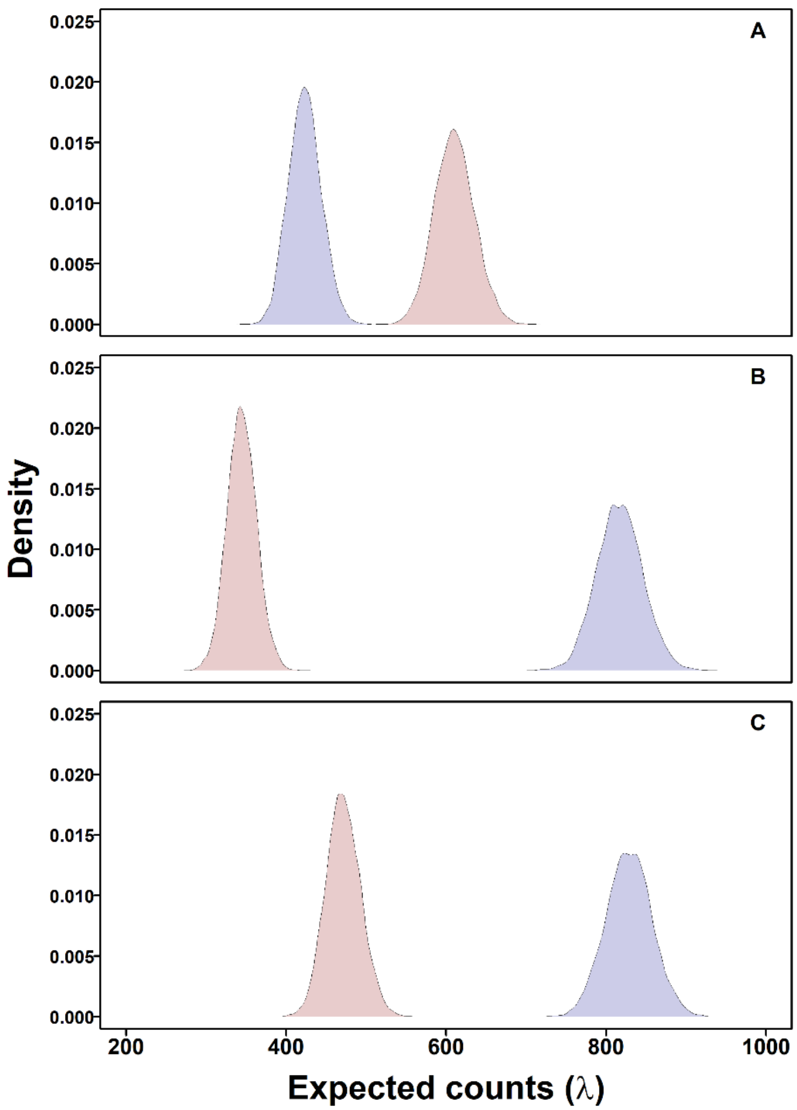

Supplementary Figure 150. Posterior densities of the expected counts (λ) for the consumption of giant red shrimp (Red= "Yes", and Blue= "No") per province (from A to C: "South", "Central", and "North"), extracted after 10^4 MCMC draws.

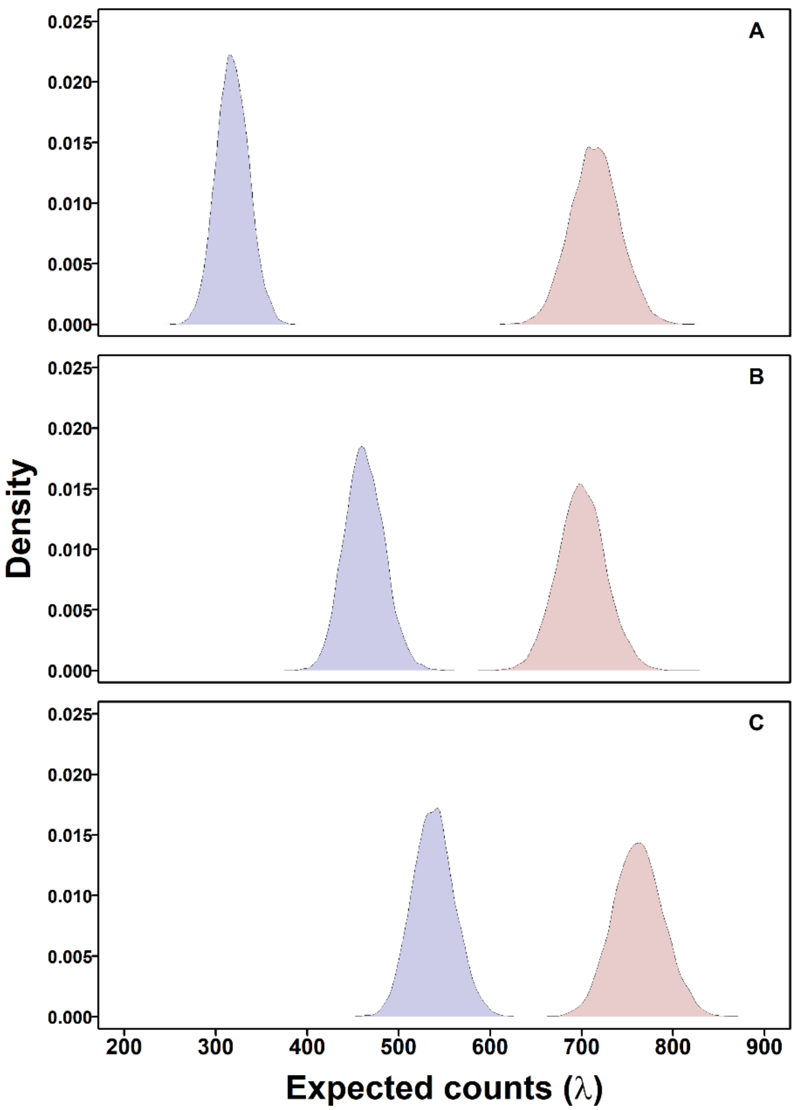

Supplementary Figure 151. Posterior densities of the expected counts (λ) for the consumption of processed albacore tuna (Red= "Yes", and Blue= "No") per province (from A to C: "South", "Central", and "North"), extracted after 10^4 MCMC draws.

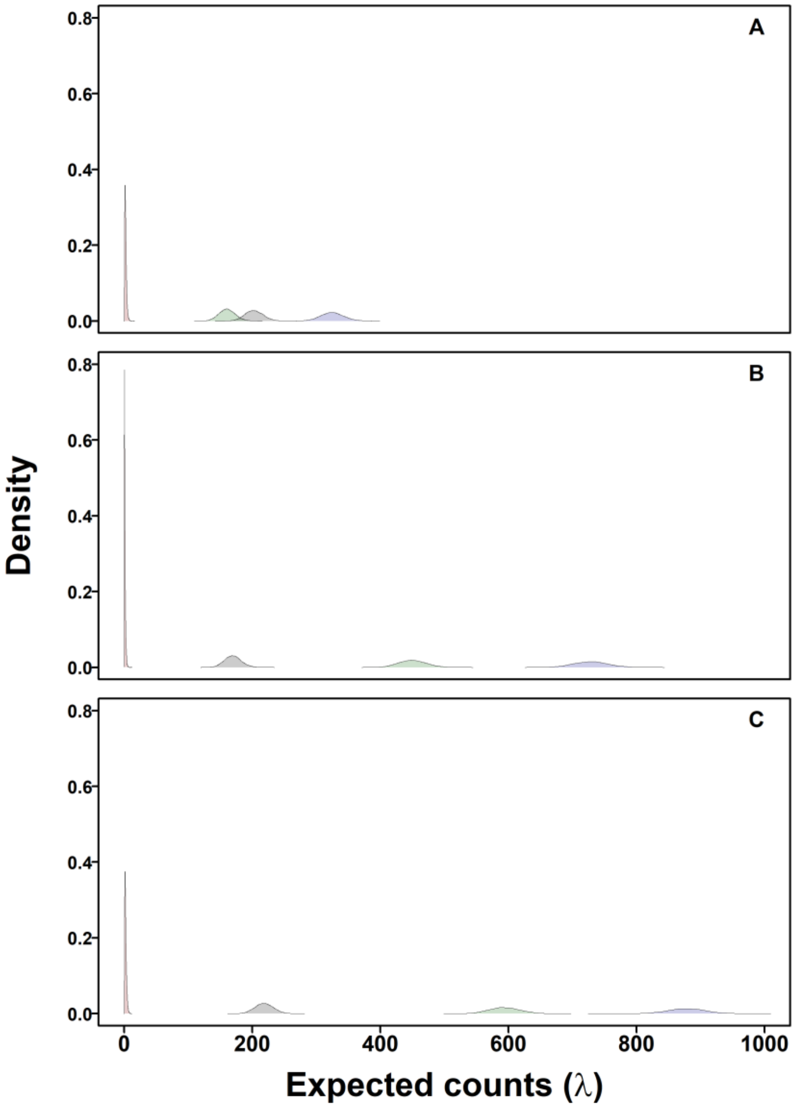

Supplementary Figure 152. Posterior densities of the expected counts (λ) for monthly seafood consumption rate (Red= 0, Blue= 1–5, Green= 6–10, and Dark gray= 10+) per educational level (from A to C: “Middle school degree”, “High school degree”, and “BSc degree or higher”), extracted after 10^4 MCMC draws.

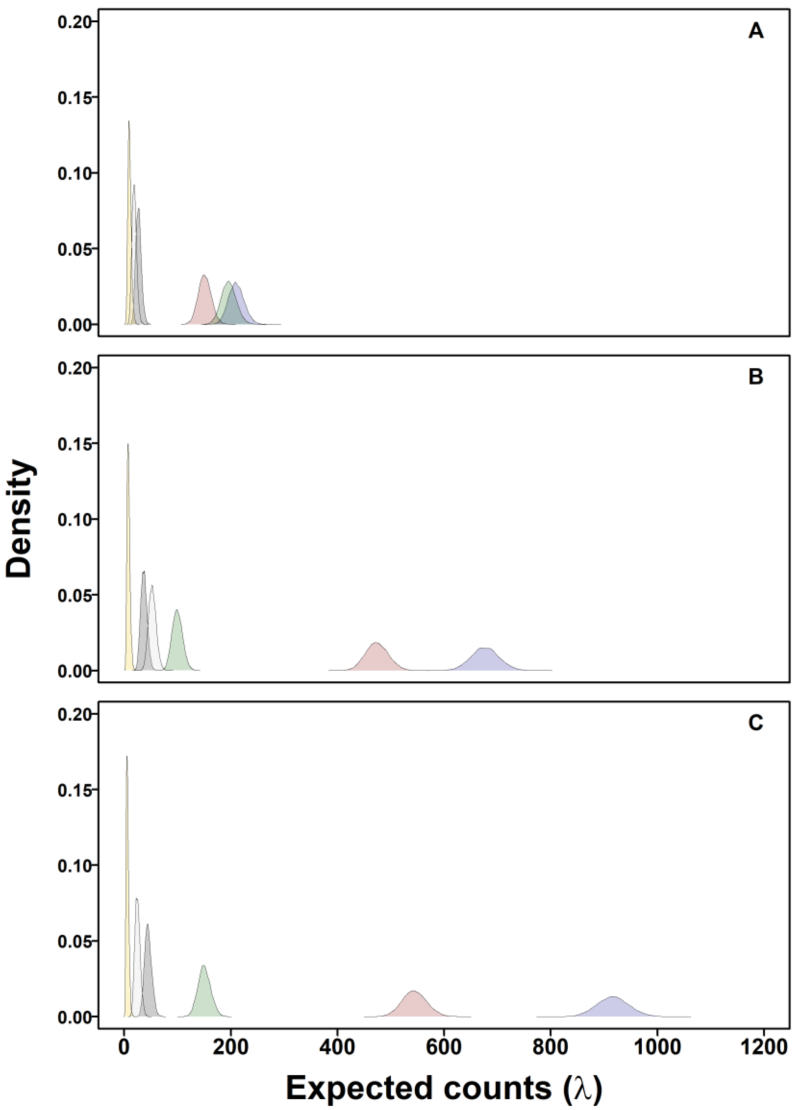

Supplementary Figure 153. Posterior densities of the expected counts (λ) for where consumers buy seafood (Red= "Fish market", Blue= "Large retail", Green= "Local market", Dark gray= "Not buy", Yellow= "Online", and Light gray = "Other") per educational level (from A to C: "Middle school degree", "High school degree", and "BSc degree or higher"), extracted after 10^4 MCMC draws.

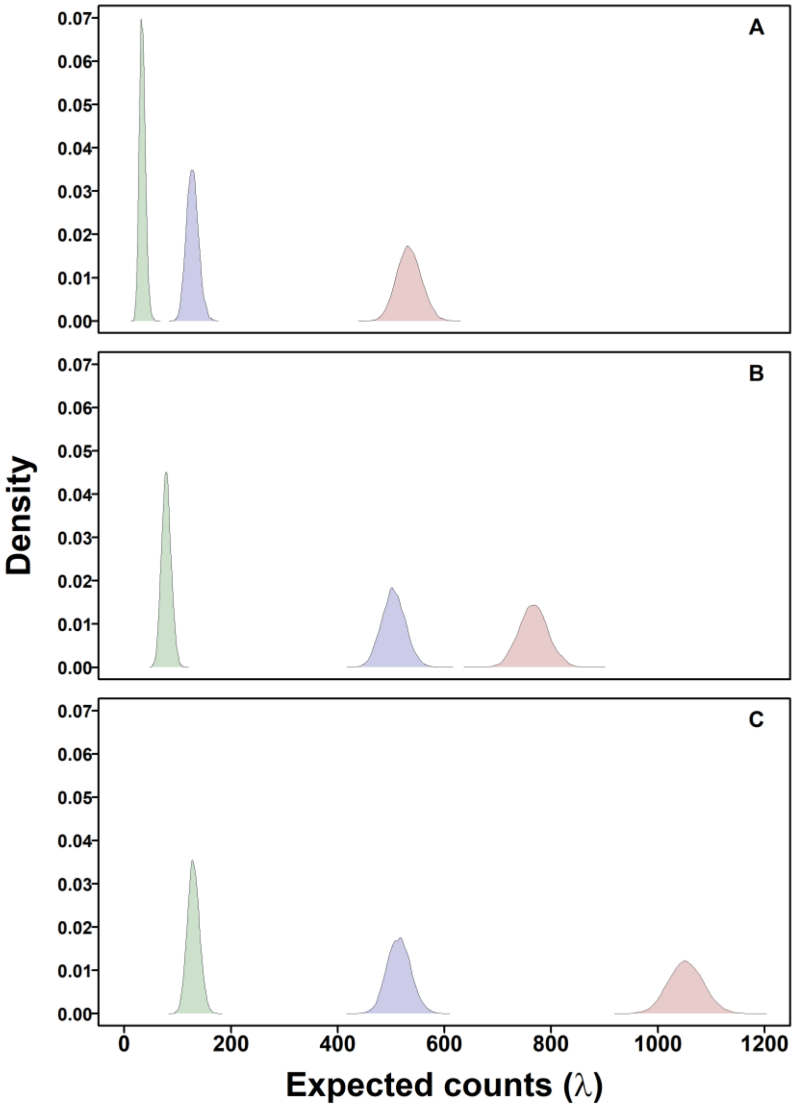

Supplementary Figure 154. Posterior densities of the expected counts (λ) for the degree of seafood processing consumers prefer (Red= "Fresh", Blue= "Frozen", and Green= "Processed") per educational level (from A to C: "Middle school degree", "High school degree", and "BSc degree or higher"), extracted after 10^4 MCMC draws.

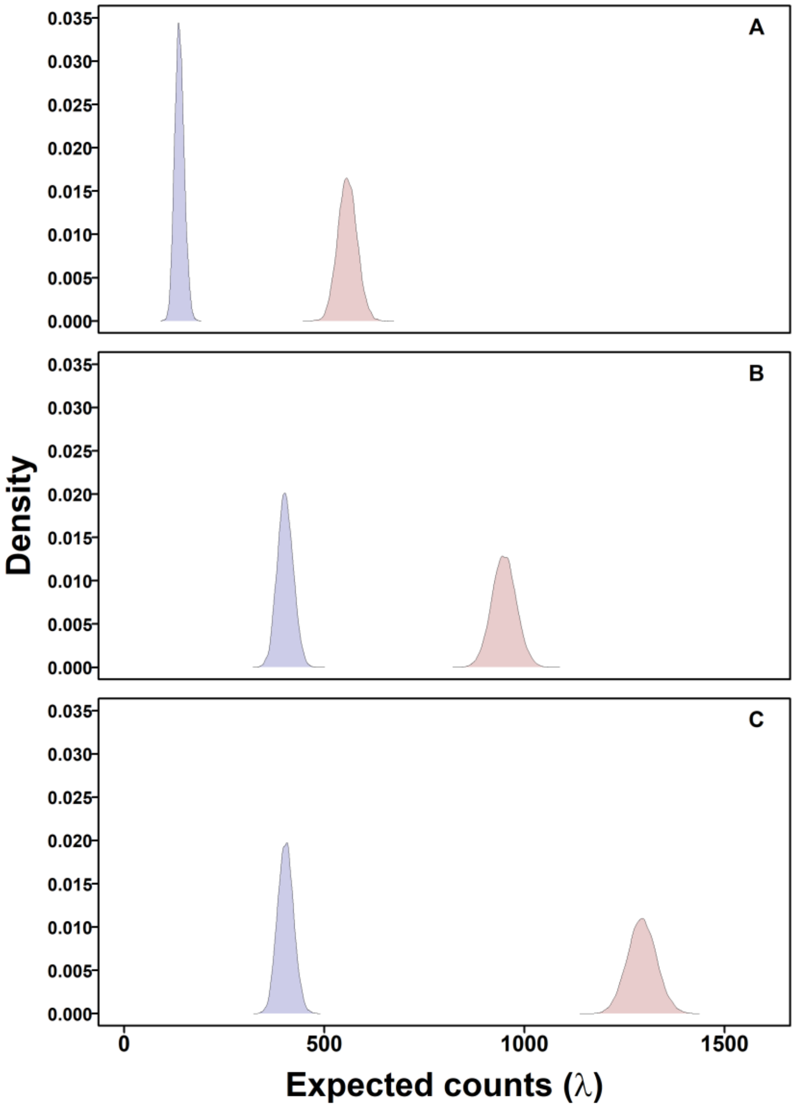

Supplementary Figure 155. Posterior densities of the expected counts (λ) for being informed on seafood origin (Red= "Yes" and Blue= "No") per educational level (from A to C: "Middle school degree", "High school degree", and "BSc degree or higher"), extracted after 10^4 MCMC draws.

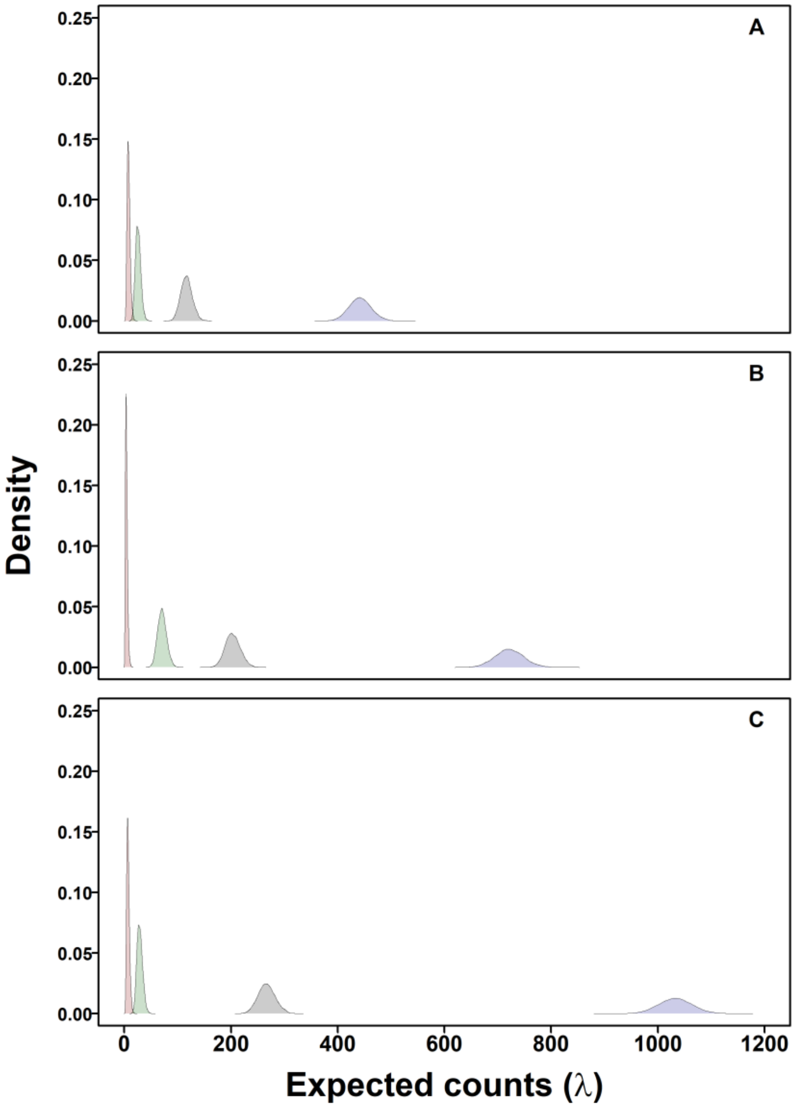

Supplementary Figure 156. Posterior densities of the expected counts (λ) for sources of information on seafood origin (Red= "Ads", Blue= "Label", Green= "Other", and Dark gray= "Retailer") per educational level (from A to C: "Middle school degree", "High school degree", and "BSc degree or higher"), extracted after 10^4 MCMC draws.

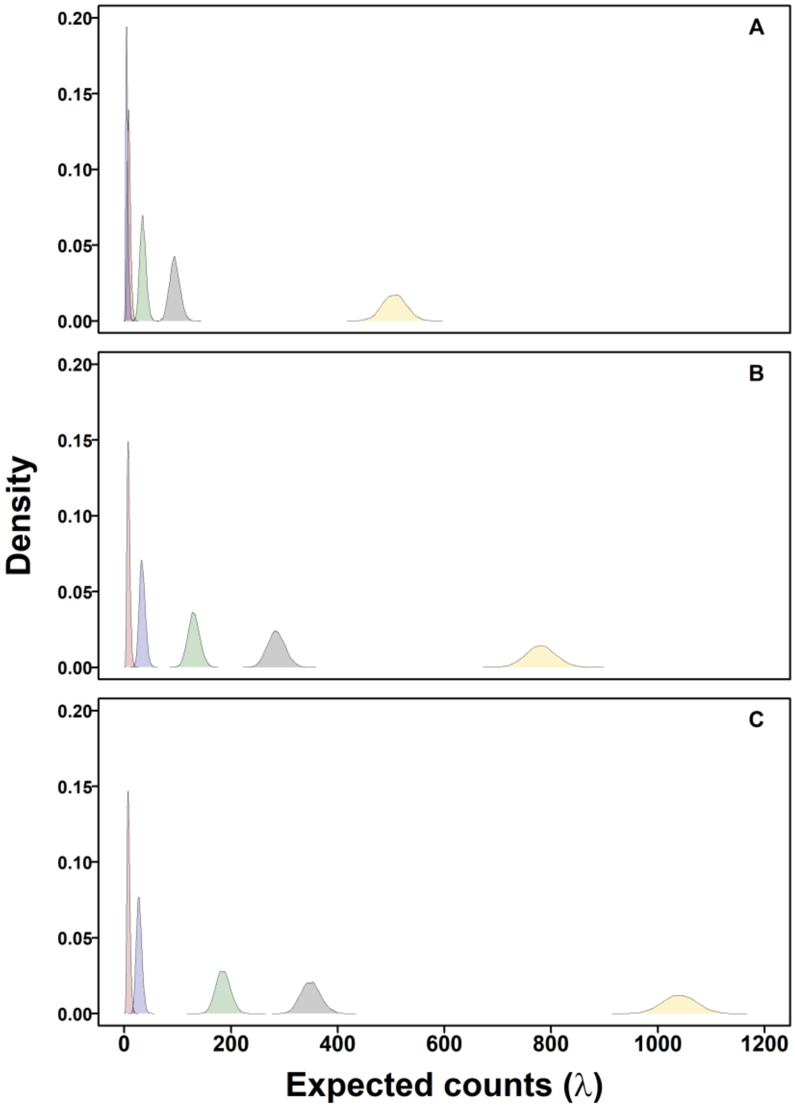

Supplementary Figure 157. Posterior densities of the expected counts (λ) for level of consumers' interest in seafood traceability (Red= 1, Blue= 2, Green= 3, Dark gray= 4, and Yellow= 5) per educational level (from A to C: "Middle school degree", "High school degree", and "BSc degree or higher"), extracted after 10^4 MCMC draws.

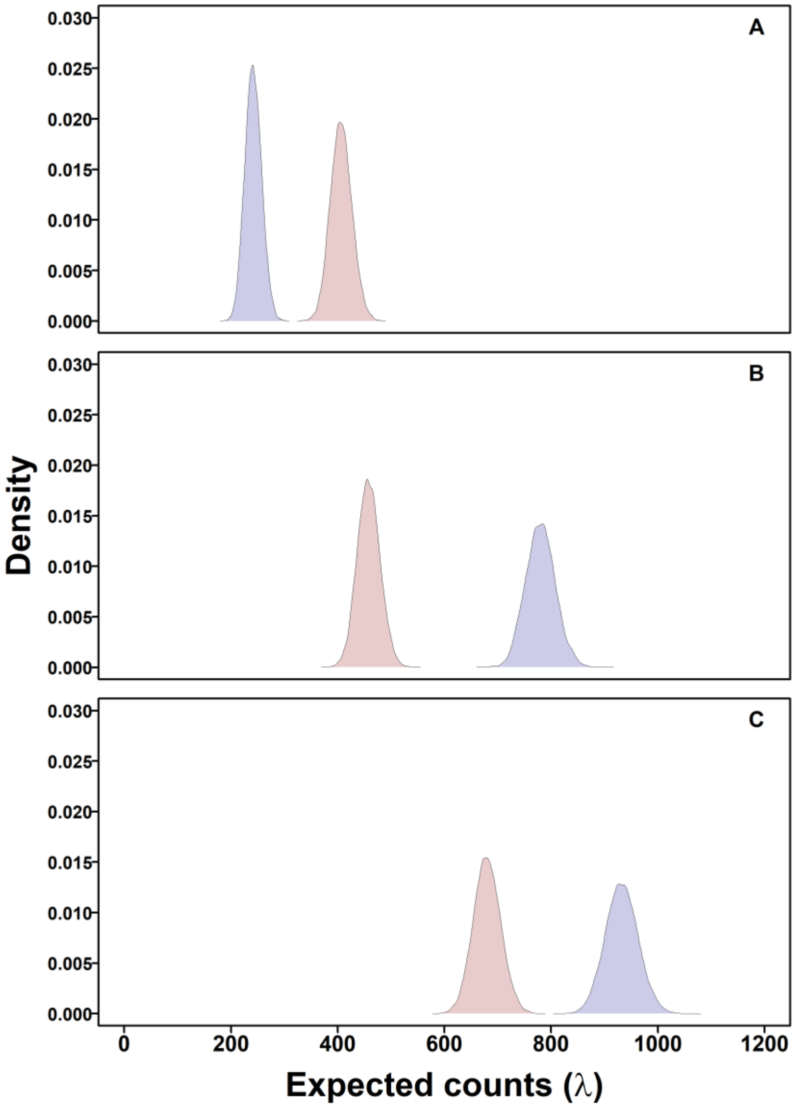

Supplementary Figure 158. Posterior densities of the expected counts (λ) for the consumption of Italian-farmed sea bass (Red= "Yes", and Blue= "No") per educational level (from A to C: "Middle school degree", "High school degree", and "BSc degree or higher"), extracted after 10^4 MCMC draws.

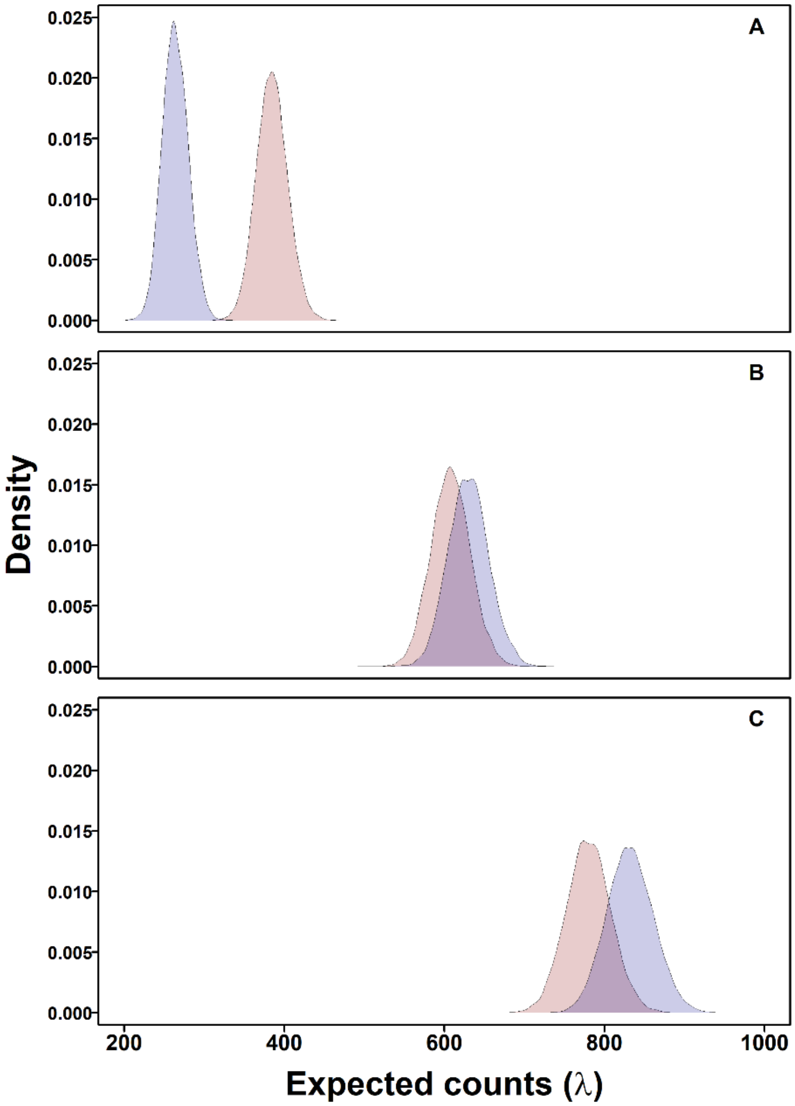

Supplementary Figure 159. Posterior densities of the expected counts (λ) for the consumption of striped venus clams (Red= “Yes”, and Blue= “No”) per educational level (from A to C: “Middle school degree”, “High school degree”, and “BSc degree or higher”), extracted after 10^4 MCMC draws.

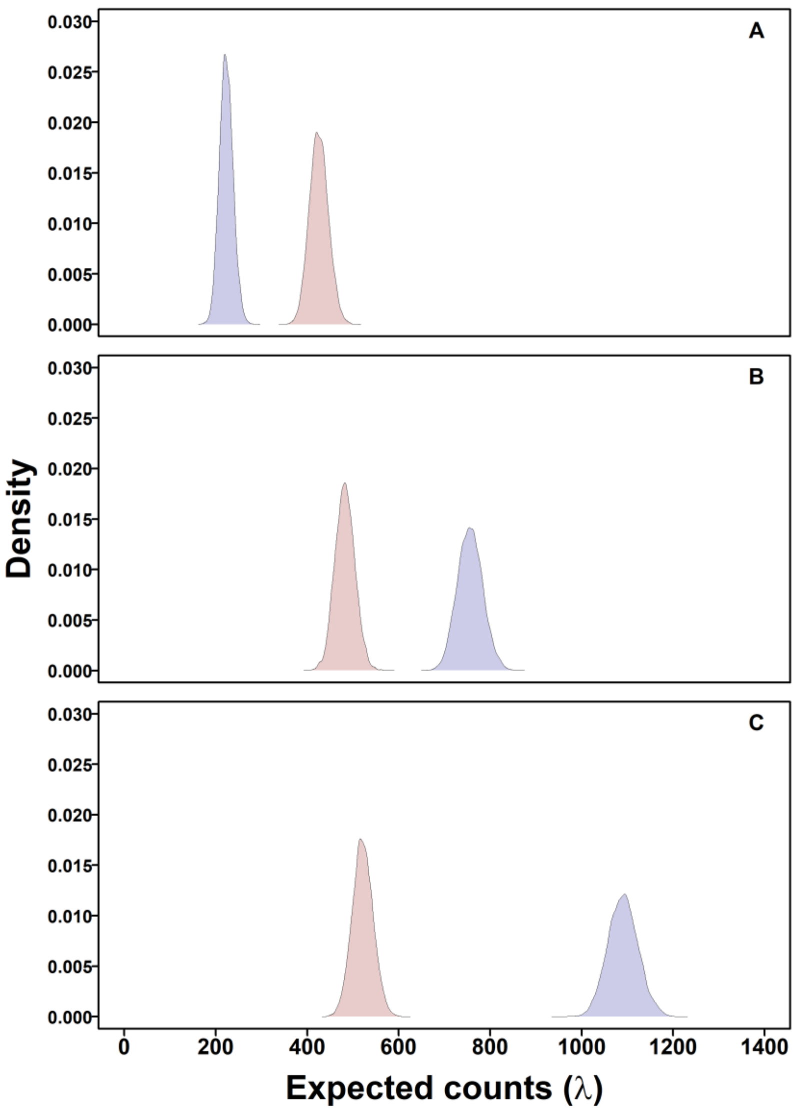

Supplementary Figure 160. Posterior densities of the expected counts (λ) for the consumption of giant red shrimp (Red= “Yes”, and Blue= “No”) per educational level (from A to C: “Middle school degree”, “High school degree”, and “BSc degree or higher”), extracted after 10^4 MCMC draws.

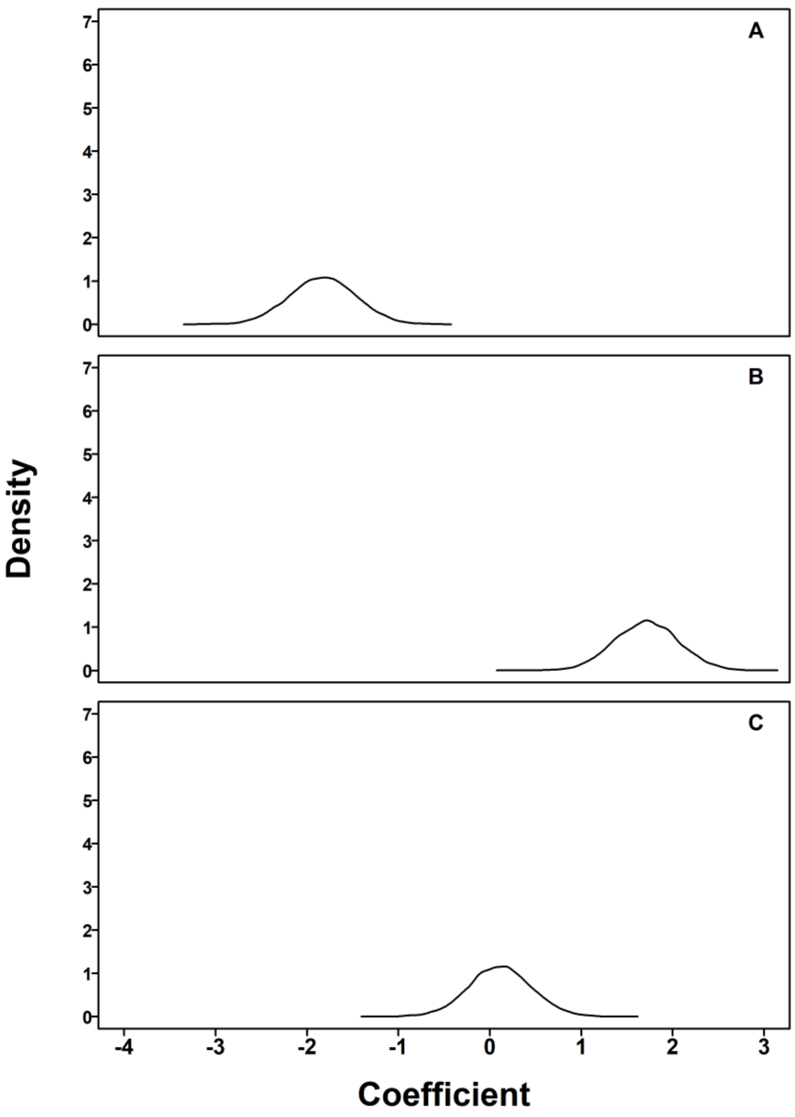

Supplementary Figure 161. Posterior densities of the ANOVA coefficients for the consumers' WTP for giant red shrimp per educational level (from A to C: "Middle school degree", "High school degree", and "BSc degree or higher"), extracted after 10^4 MCMC draws.

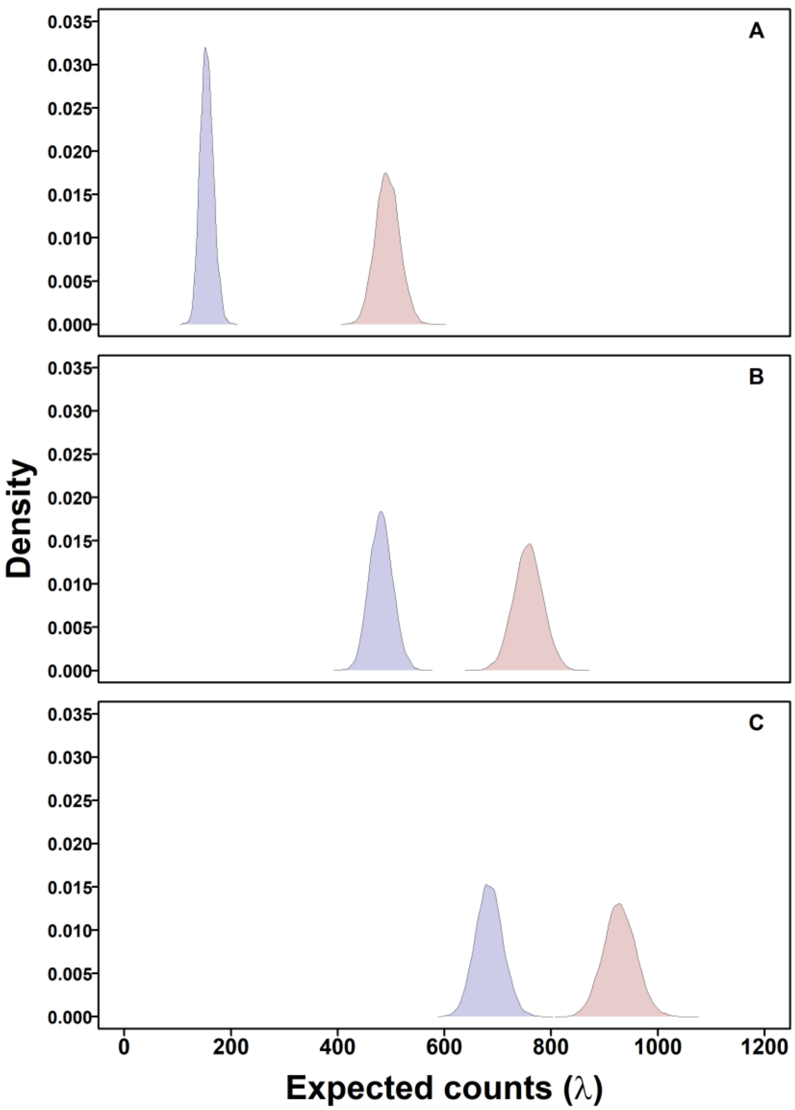

Supplementary Figure 162. Posterior densities of the expected counts (λ) for the consumption of processed albacore tuna (Red= "Yes", and Blue= "No") per educational level (from A to C: "Middle school degree", "High school degree", and "BSc degree or higher"), extracted after 10^4 MCMC draws.

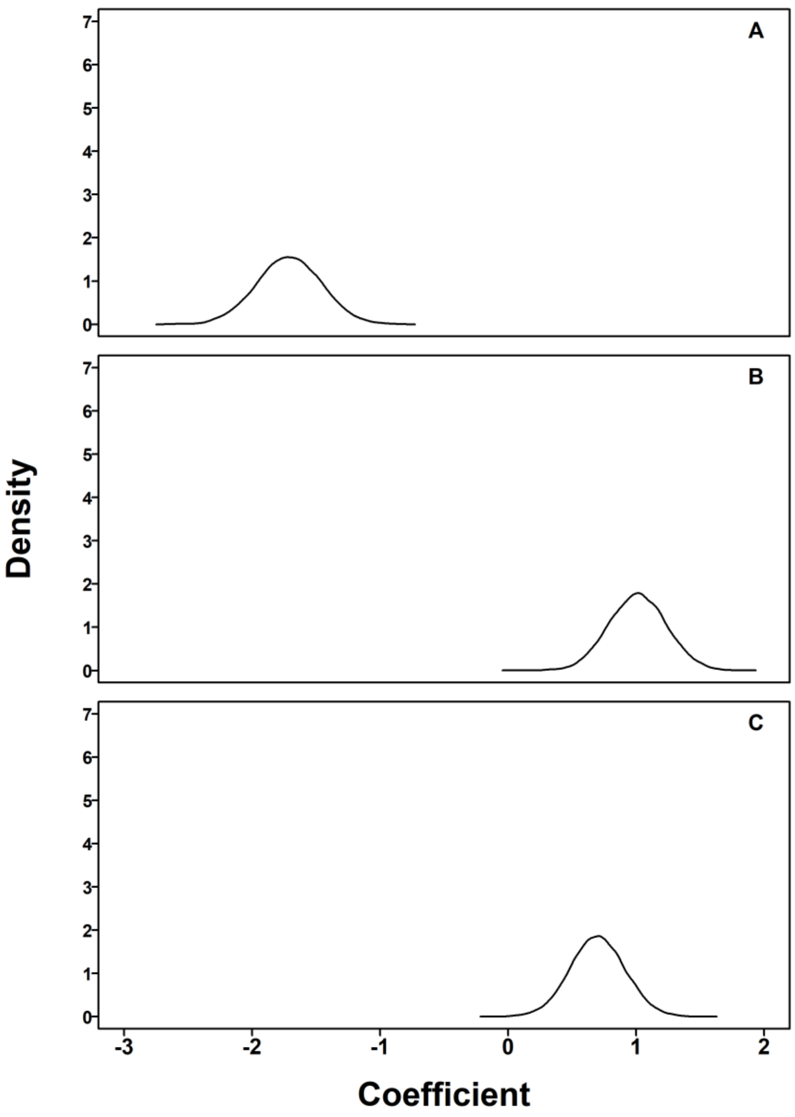

Supplementary Figure 163. Posterior densities of the ANOVA coefficients for the consumers' WTP for processed albacore tuna per educational level (from A to C: "Middle school degree", "High school degree", and "BSc degree or higher"), extracted after 10^4 MCMC draws.

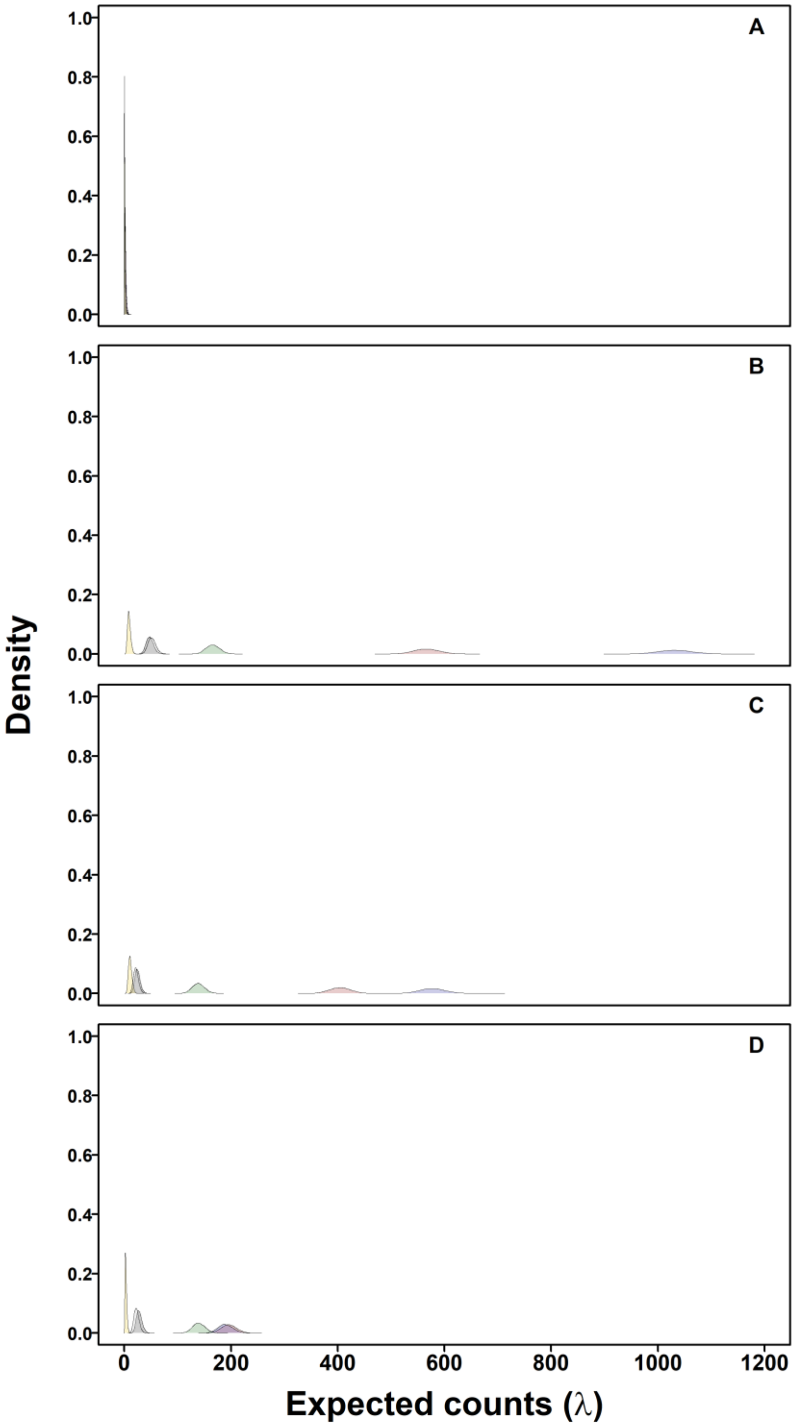

Supplementary Figure 164. Posterior densities of the expected counts (λ) for where consumers buy seafood (Red= “Fish market”, Blue= “Large retail”, Green= “Local market”, Dark gray= “Not buy”, Yellow= “Online”, and Light gray = “Other”) per monthly seafood consumption rate (from A to D: 0, 1–5, 6–10, and 10+), extracted after 10^4 MCMC draws.

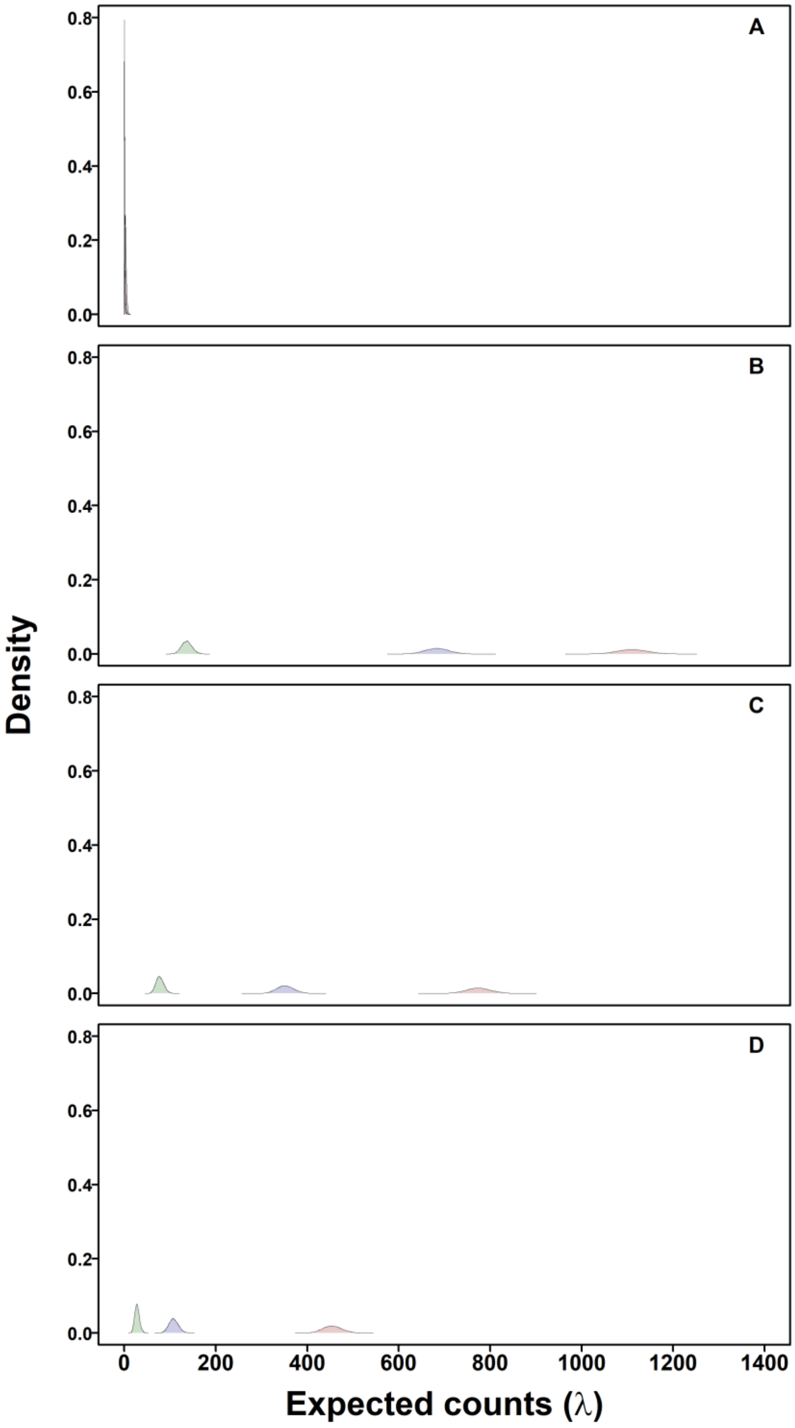

Supplementary Figure 165. Posterior densities of the expected counts (λ) for the degree of seafood processing consumers prefer (Red= "Fresh", Blue= "Frozen", and Green= "Processed") per monthly seafood consumption rate (from A to D: 0, 1–5, 6–10, and 10+), extracted after 10^4 MCMC draws.

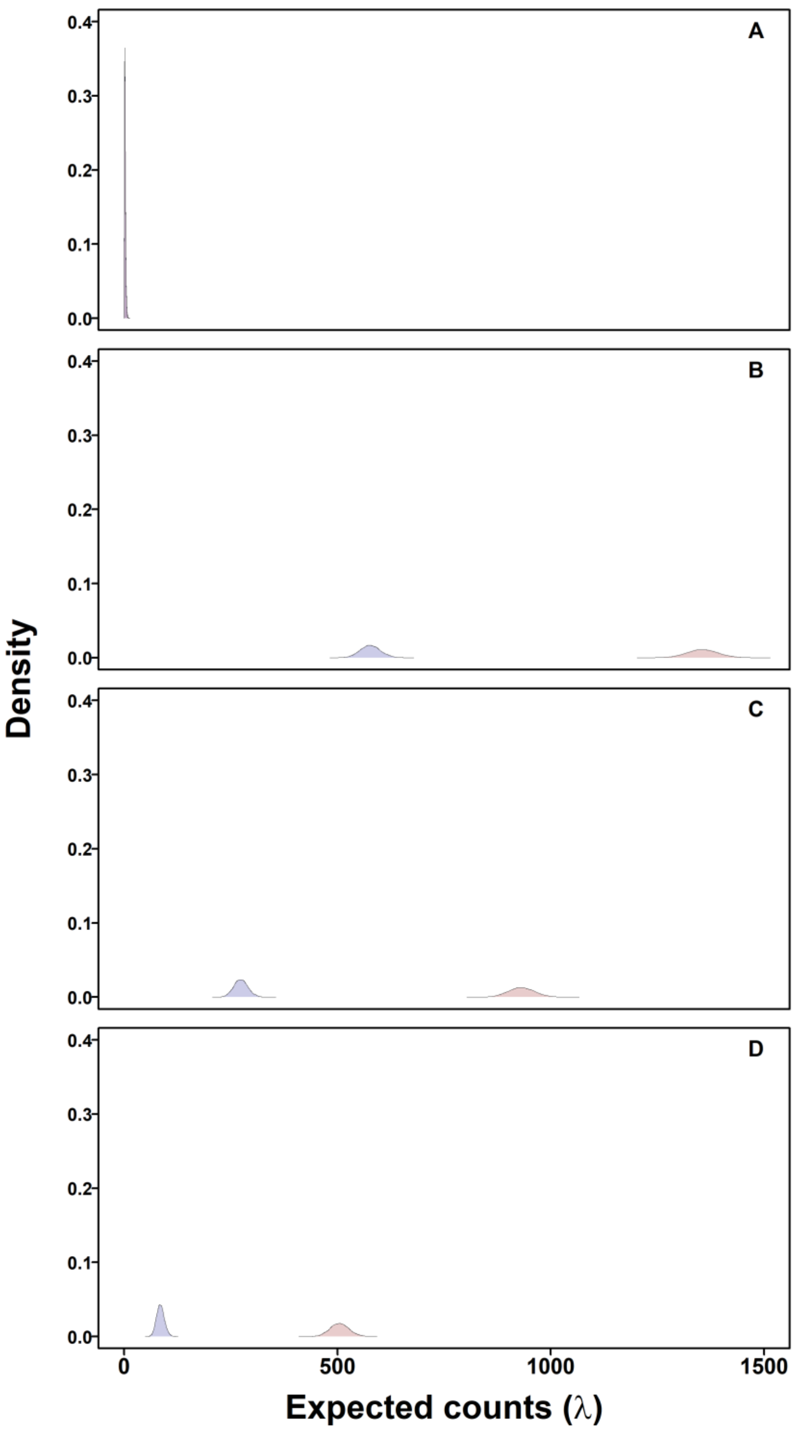

Supplementary Figure 166. Posterior densities of the expected counts (λ) for being informed on seafood origin (Red= "Yes" and Blue= "No") per monthly seafood consumption rate (from A to D: 0, 1–5, 6–10, and 10+), extracted after 10^4 MCMC draws.

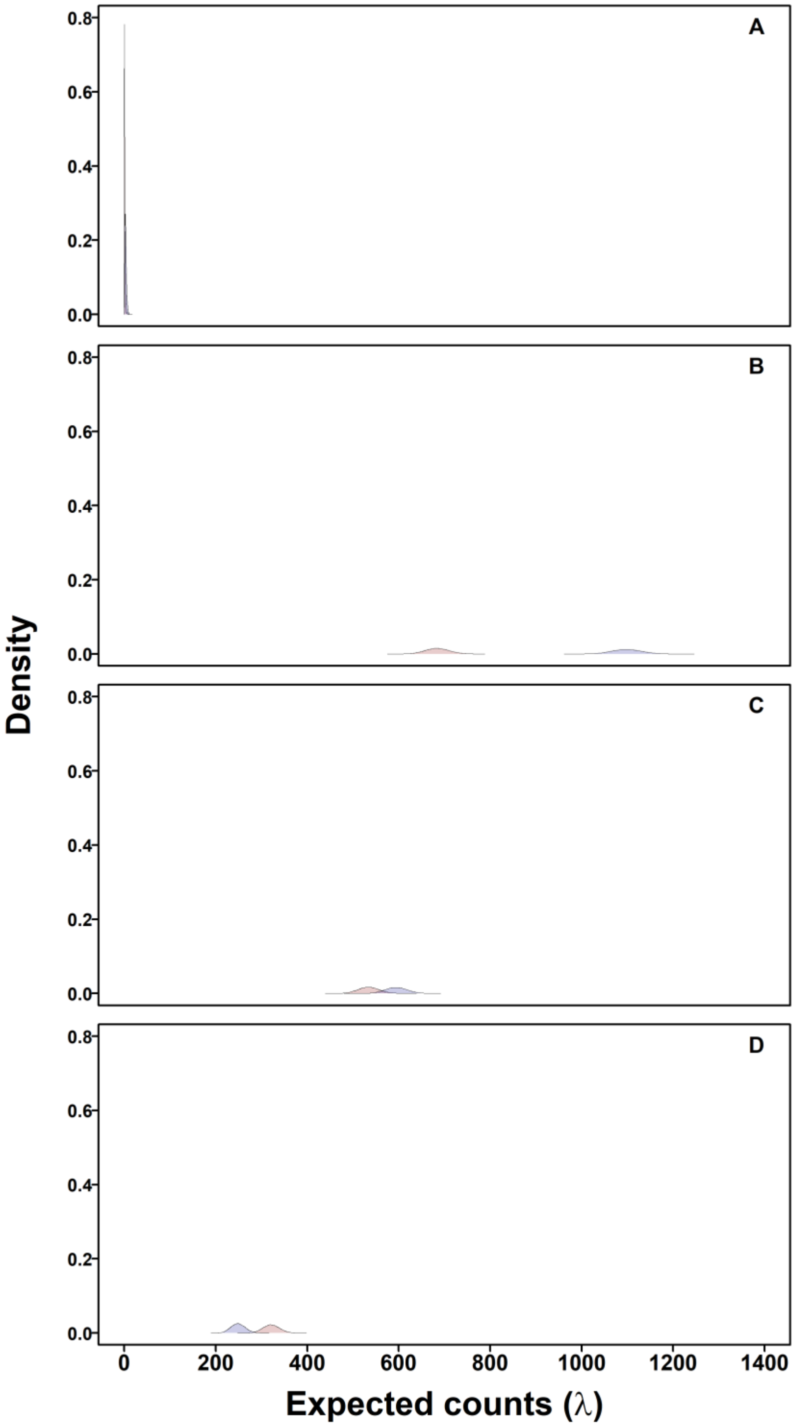

Supplementary Figure 167. Posterior densities of the expected counts (λ) for the consumption of Italian-farmed sea bass (Red= "Yes", and Blue= "No") per monthly seafood consumption rate (from A to D: 0, 1–5, 6–10, and 10+), extracted after 10^4 MCMC draws.

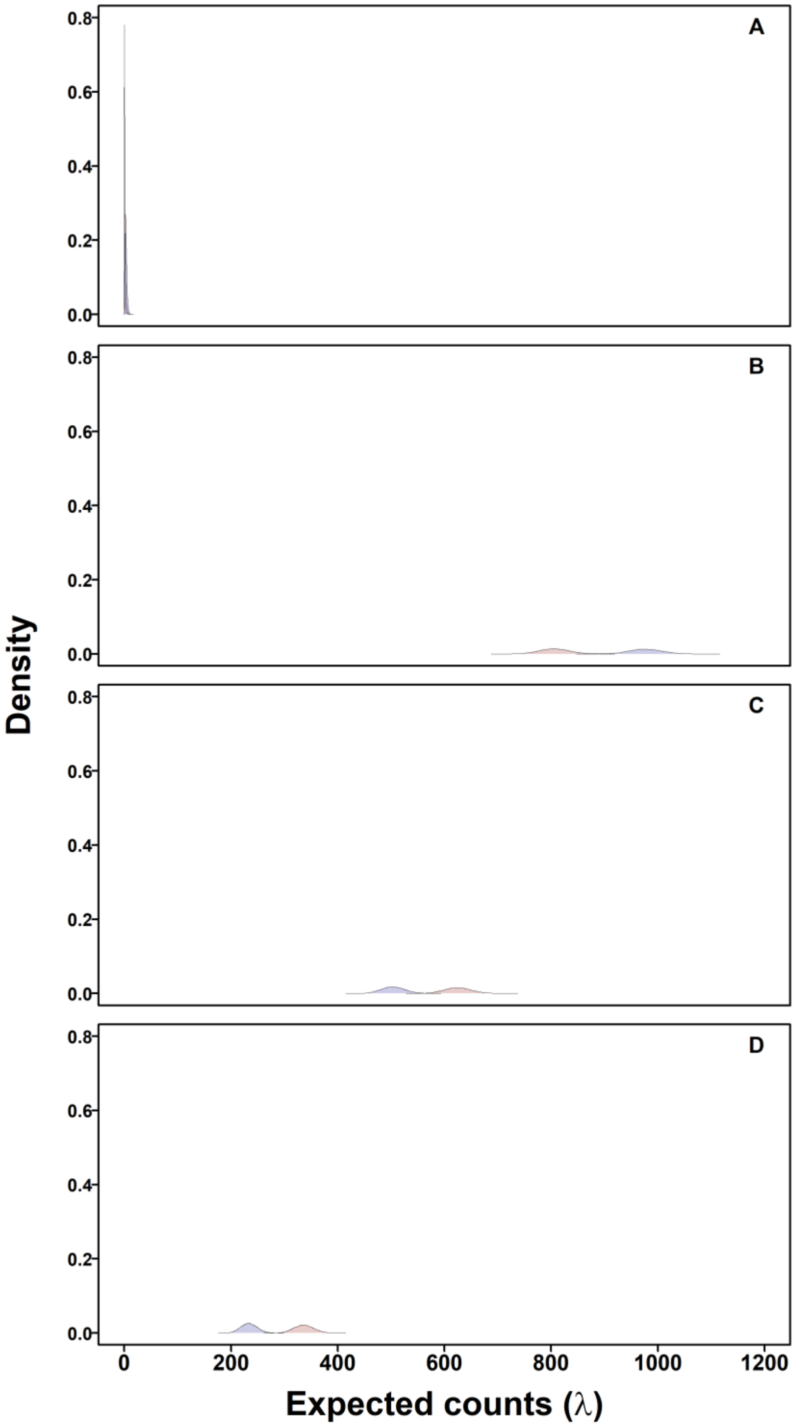

Supplementary Figure 168. Posterior densities of the expected counts (λ) for the consumption of striped venus clams (Red= "Yes", and Blue= "No") per monthly seafood consumption rate (from A to D: 0, 1–5, 6–10, and 10+), extracted after 10^4 MCMC draws.

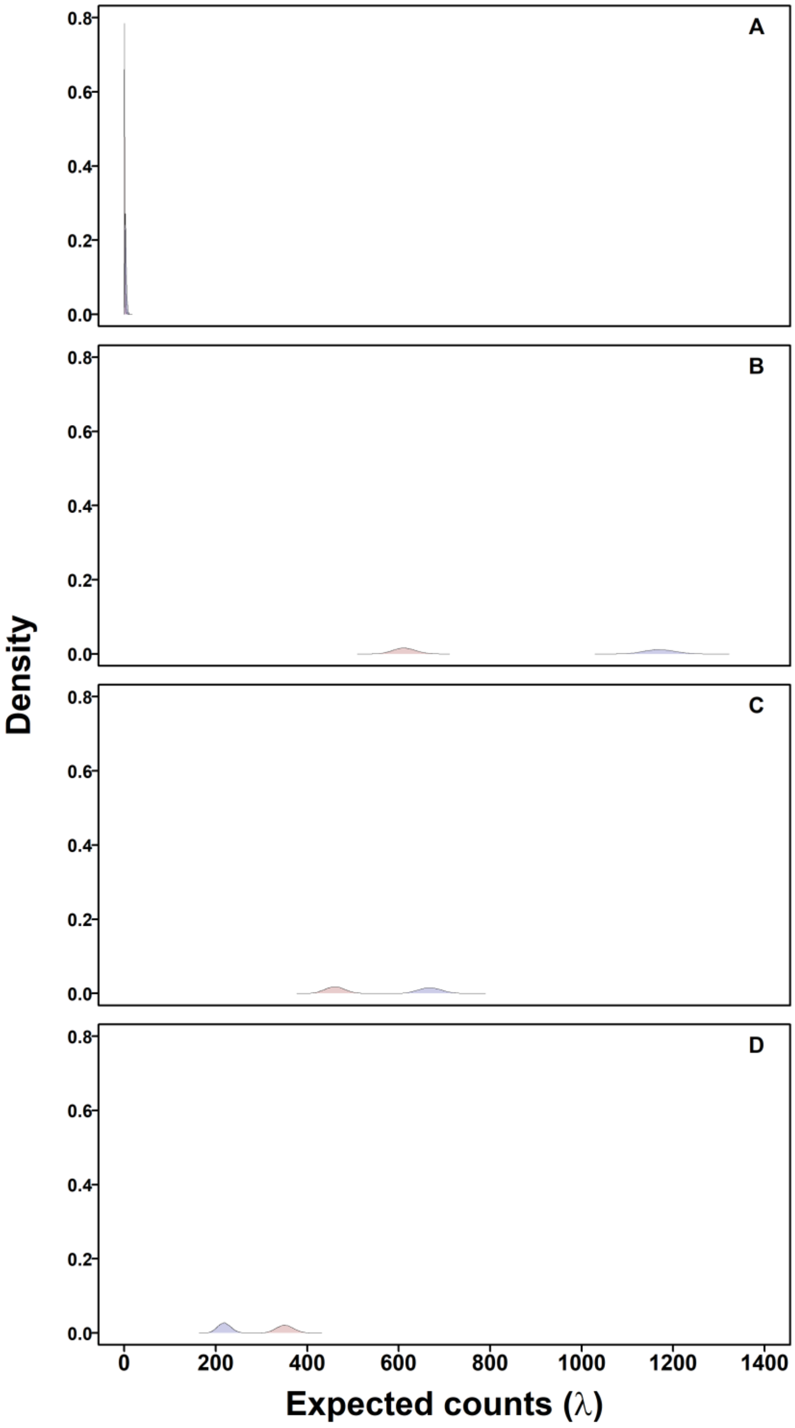

Supplementary Figure 169. Posterior densities of the expected counts (λ) for the consumption of giant red shrimp (Red= "Yes", and Blue= "No") per monthly seafood consumption rate (from A to D: 0, 1–5, 6–10, and 10+), extracted after 10^4 MCMC draws.

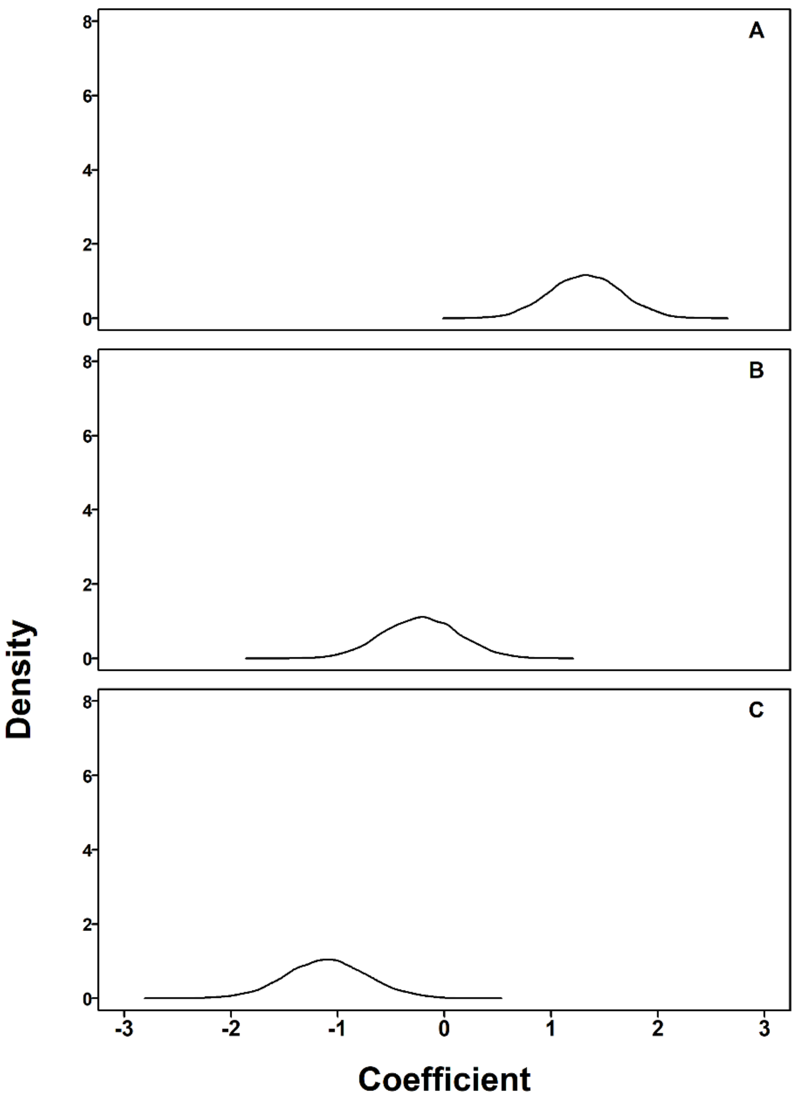

Supplementary Figure 170. Posterior densities of the ANOVA coefficients for the consumers' WTP for giant red shrimp per monthly seafood consumption rate (from A to C: 1–5, 6–10, and 10+), extracted after 10^4 MCMC draws. Note the absence of data for 0 monthly consumption.

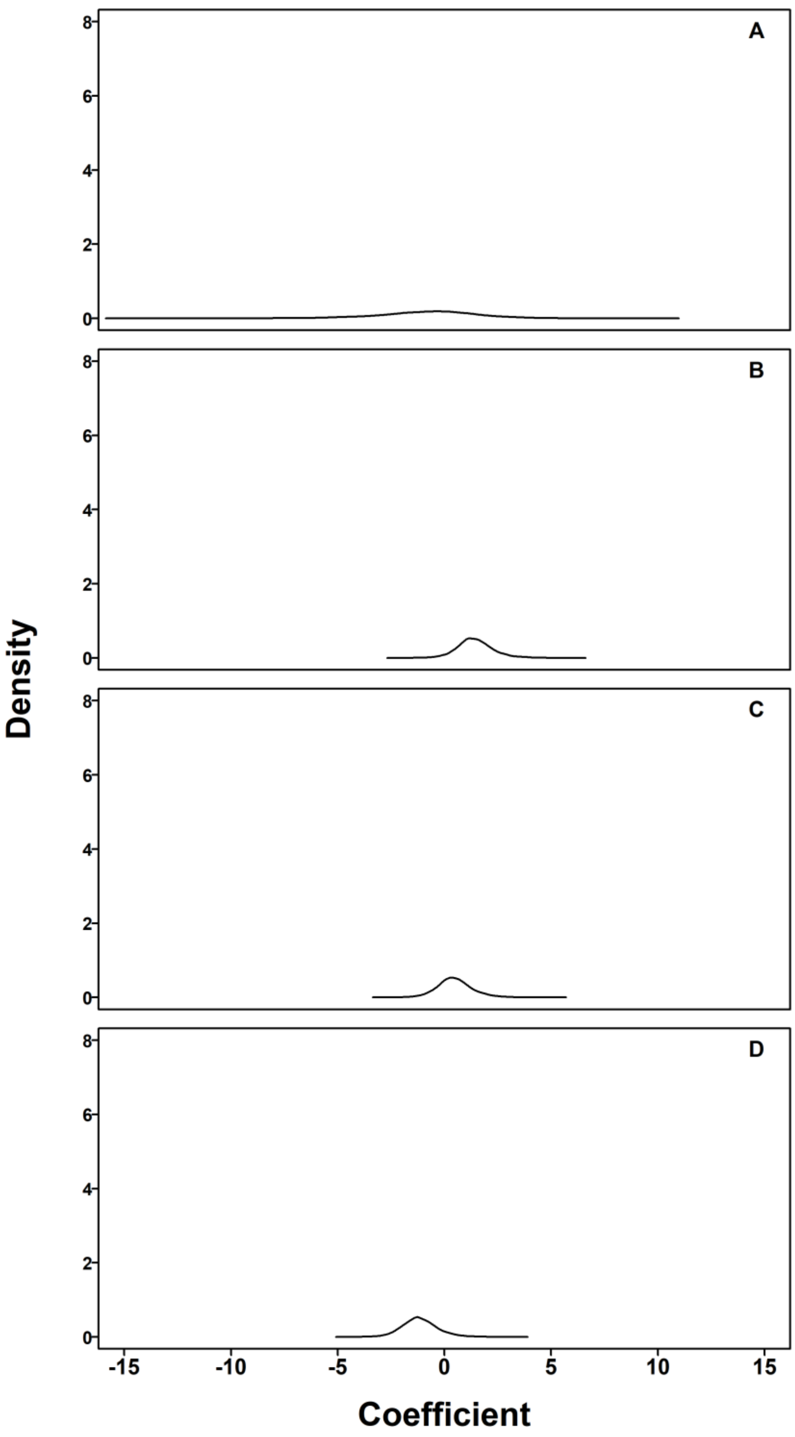

Supplementary Figure 171. Posterior densities of the ANOVA coefficients for the consumers' WTP for processed albacore tuna per monthly seafood consumption rate (from A to D: 0, 1–5, 6–10, and 10+), extracted after 10^4 MCMC draws.

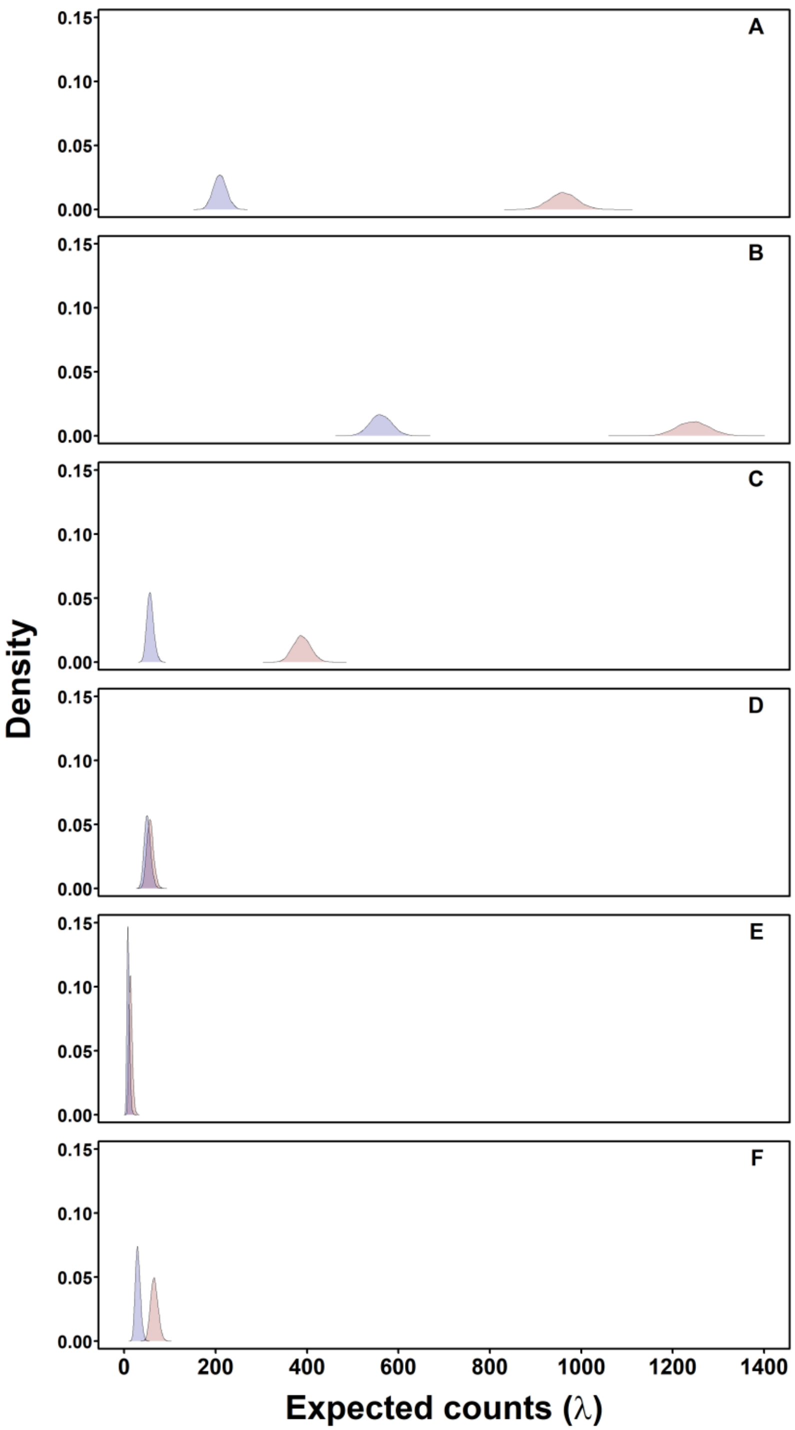

Supplementary Figure 172. Posterior densities of the expected counts (λ) for being informed on seafood origin (Red= "Yes" and Blue= "No") per where consumers buy seafood (from A to F: "Fish market", "Large retail", "Local market", "Not buy", "Online", and "Other"), extracted after 10^4 MCMC draws.

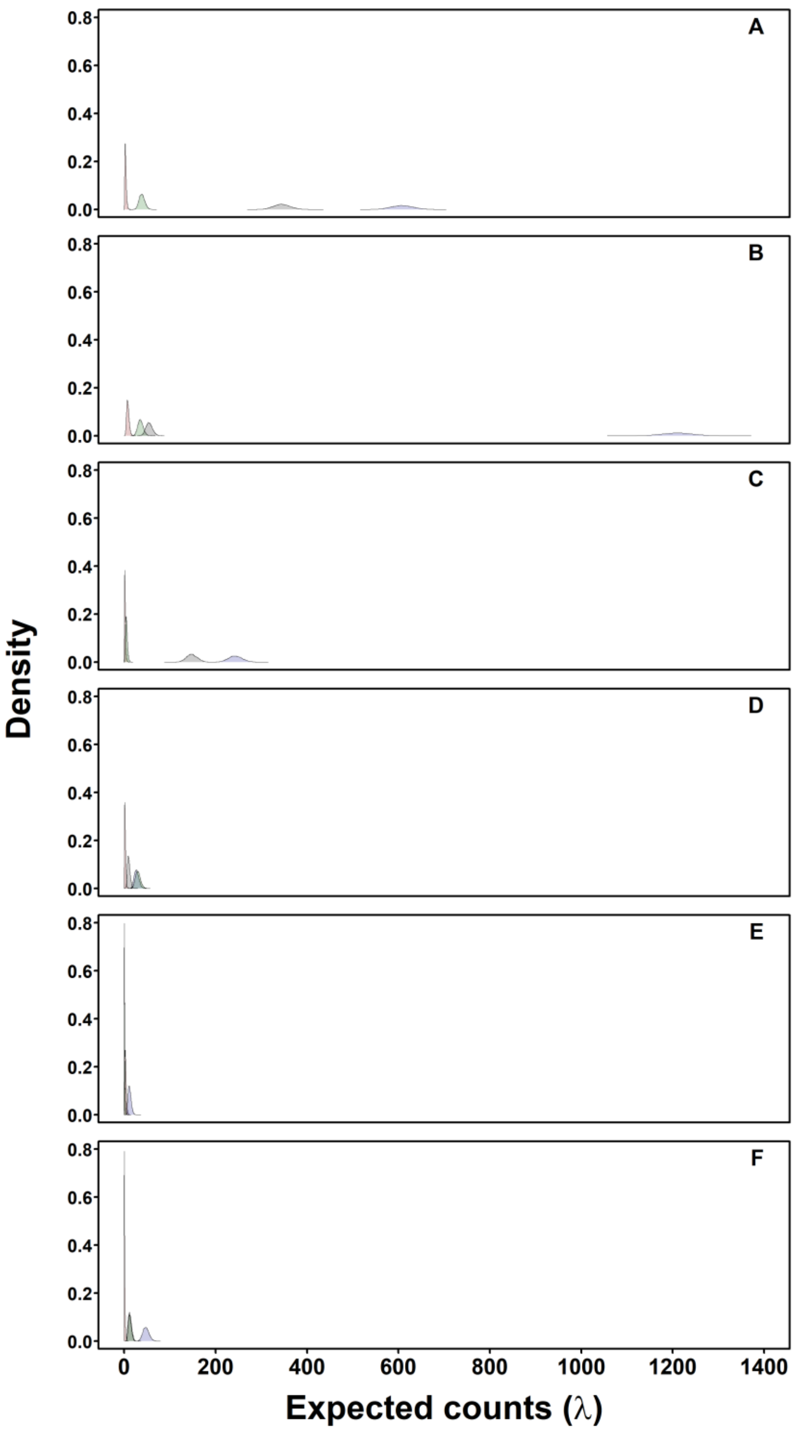

Supplementary Figure 173. Posterior densities of the expected counts (λ) for sources of information on seafood origin (Red= "Ads", Blue= "Label", Green= "Other", and Dark gray= "Retailer") per where consumers buy seafood (from A to F: "Fish market", "Large retail", "Local market", "Not buy", "Online", and "Other"), extracted after 10^4 MCMC draws.

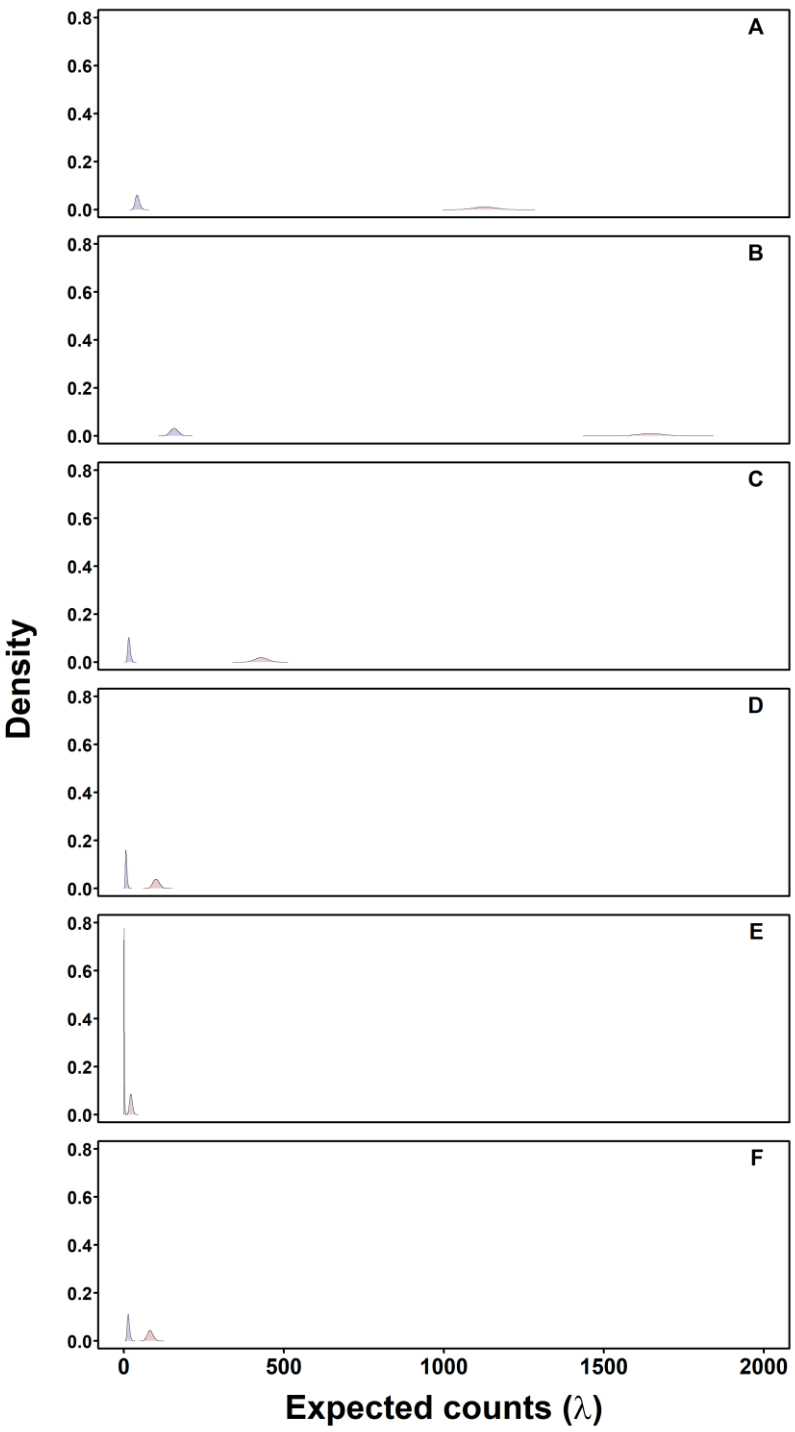

Supplementary Figure 174. Posterior densities of the expected counts (λ) for interest on seafood traceability (Red= “Yes” and Blue= “No”) per where consumers buy seafood (from A to F: “Fish market”, “Large retail”, “Local market”, “Not buy”, “Online”, and “Other”), extracted after 10^4 MCMC draws.

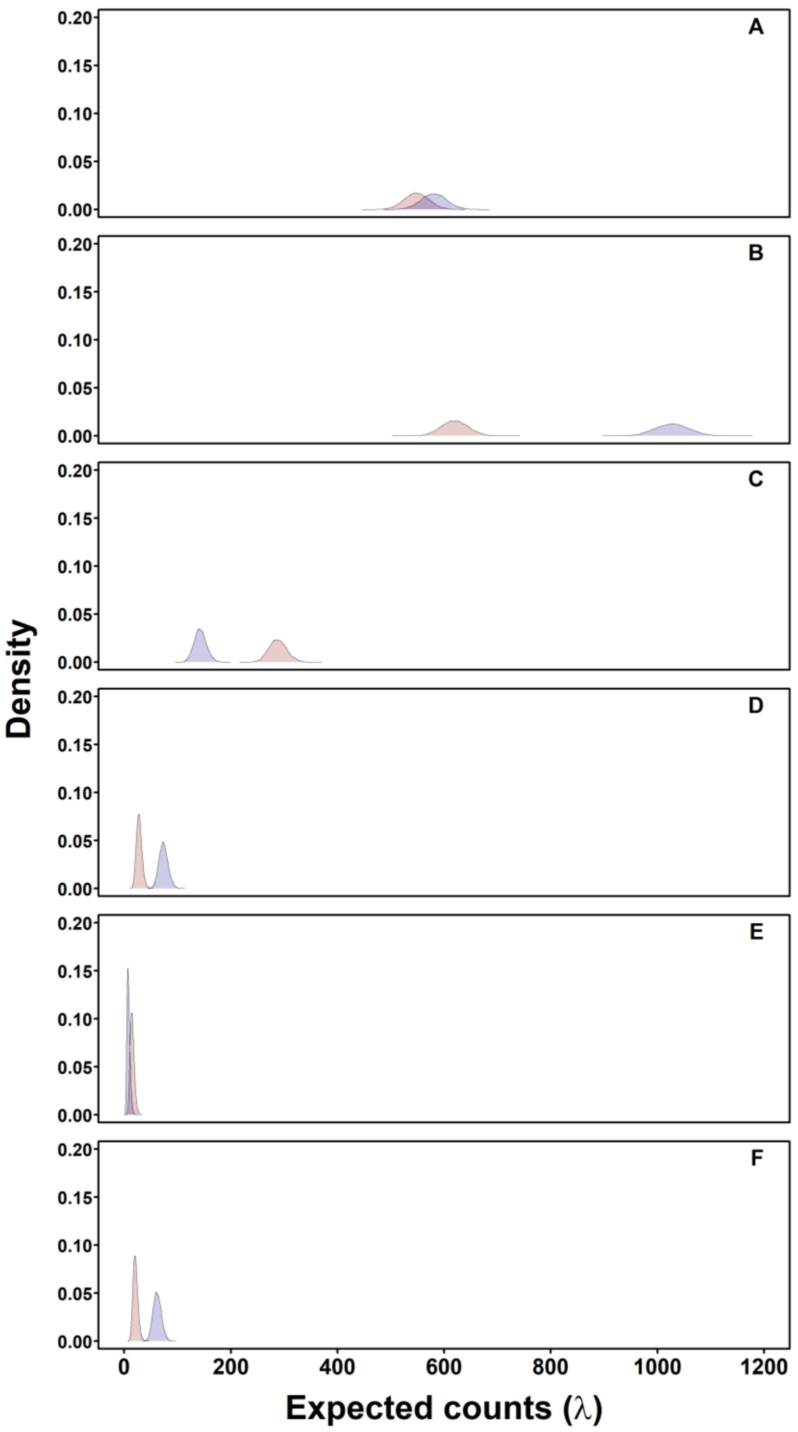

Supplementary Figure 175. Posterior densities of the expected counts (λ) for the consumption of Italian-farmed sea bass (Red= "Yes" and Blue= "No") per where consumers buy seafood (from A to F: "Fish market", "Large retail", "Local market", "Not buy", "Online", and "Other"), extracted after 10^4 MCMC draws.

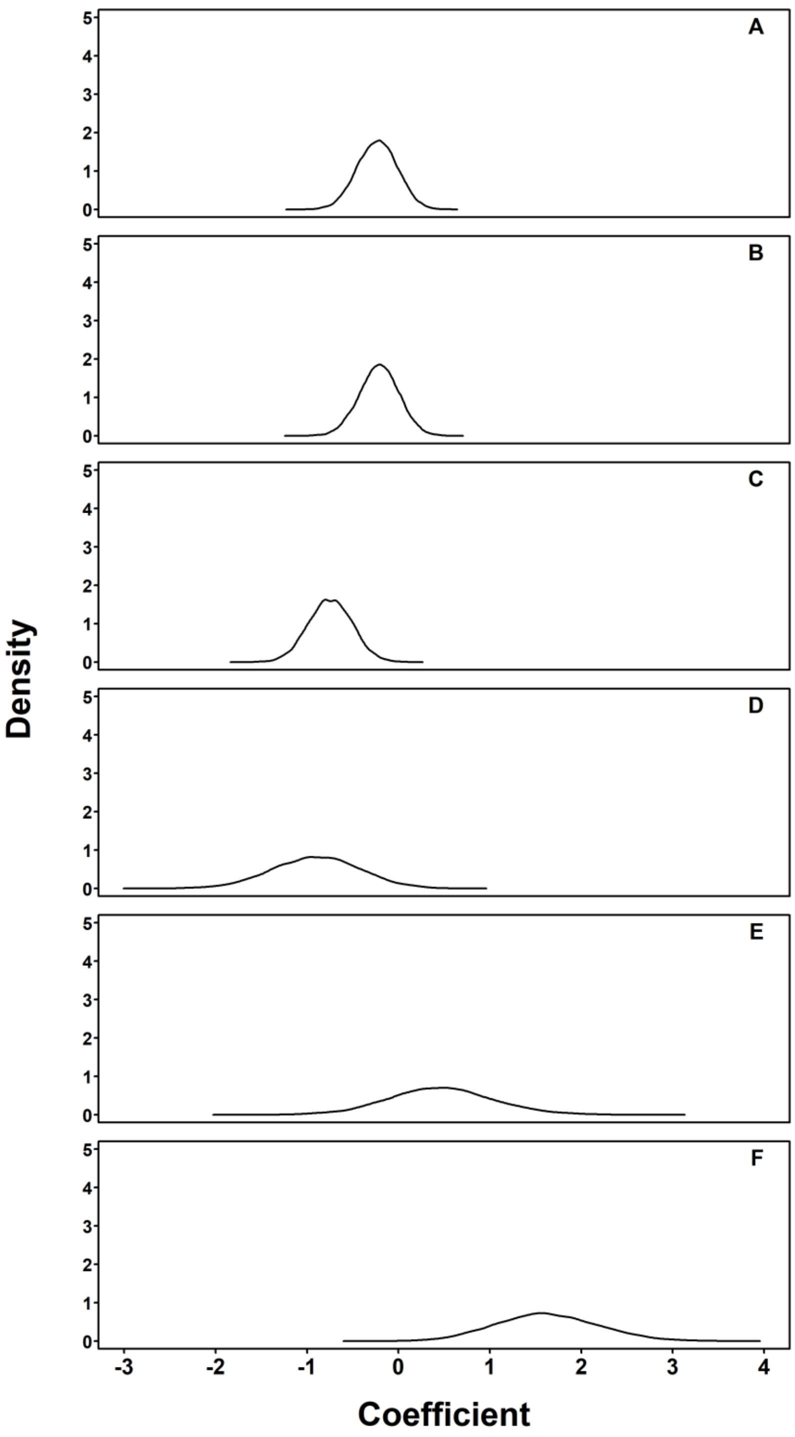

Supplementary Figure 176. Posterior densities of the ANOVA coefficients for the consumers' WTP for Italian-farmed sea bass per where consumers buy seafood (from A to F: "Fish market", "Large retail", "Local market", "Not buy", "Online", and "Other"), extracted after 10^4 MCMC draws.

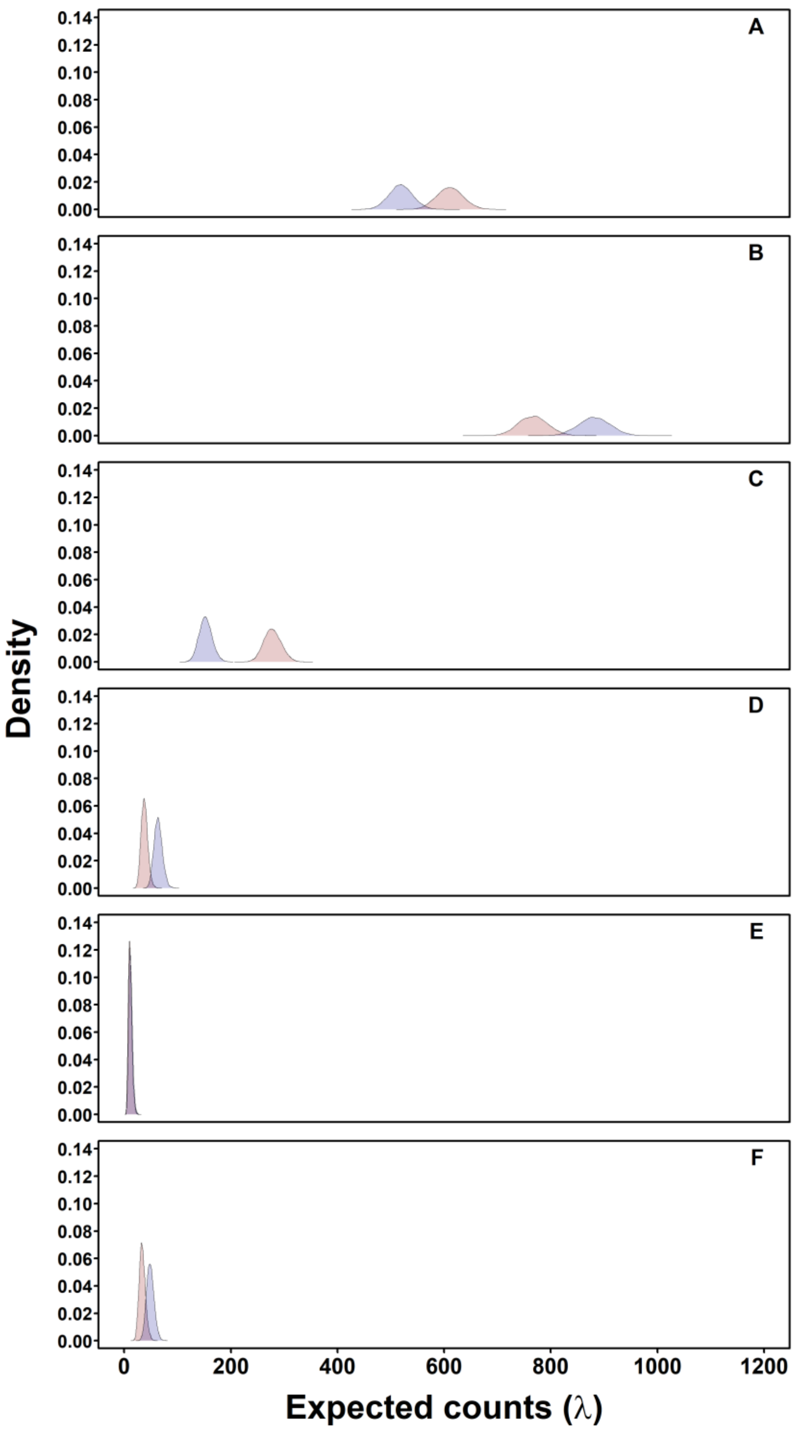

Supplementary Figure 177. Posterior densities of the expected counts (λ) for the consumption of striped venus clams (Red= "Yes" and Blue= "No") per where consumers buy seafood (from A to F: "Fish market", "Large retail", "Local market", "Not buy", "Online", and "Other"), extracted after 10^4 MCMC draws.

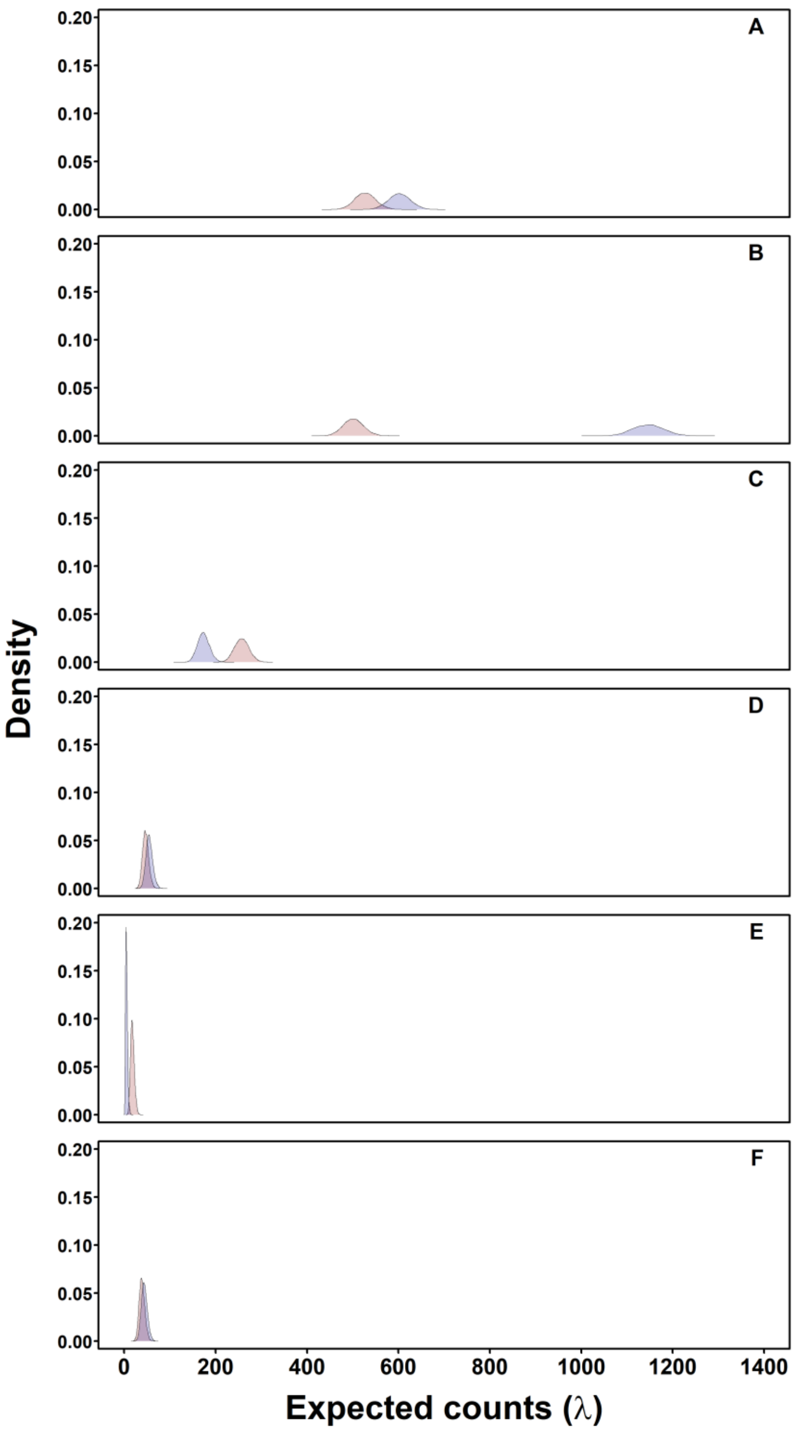

Supplementary Figure 178. Posterior densities of the expected counts (λ) for the consumption of giant red shrimp (Red= "Yes" and Blue= "No") per where consumers buy seafood (from A to F: "Fish market", "Large retail", "Local market", "Not buy", "Online", and "Other"), extracted after 10^4 MCMC draws.

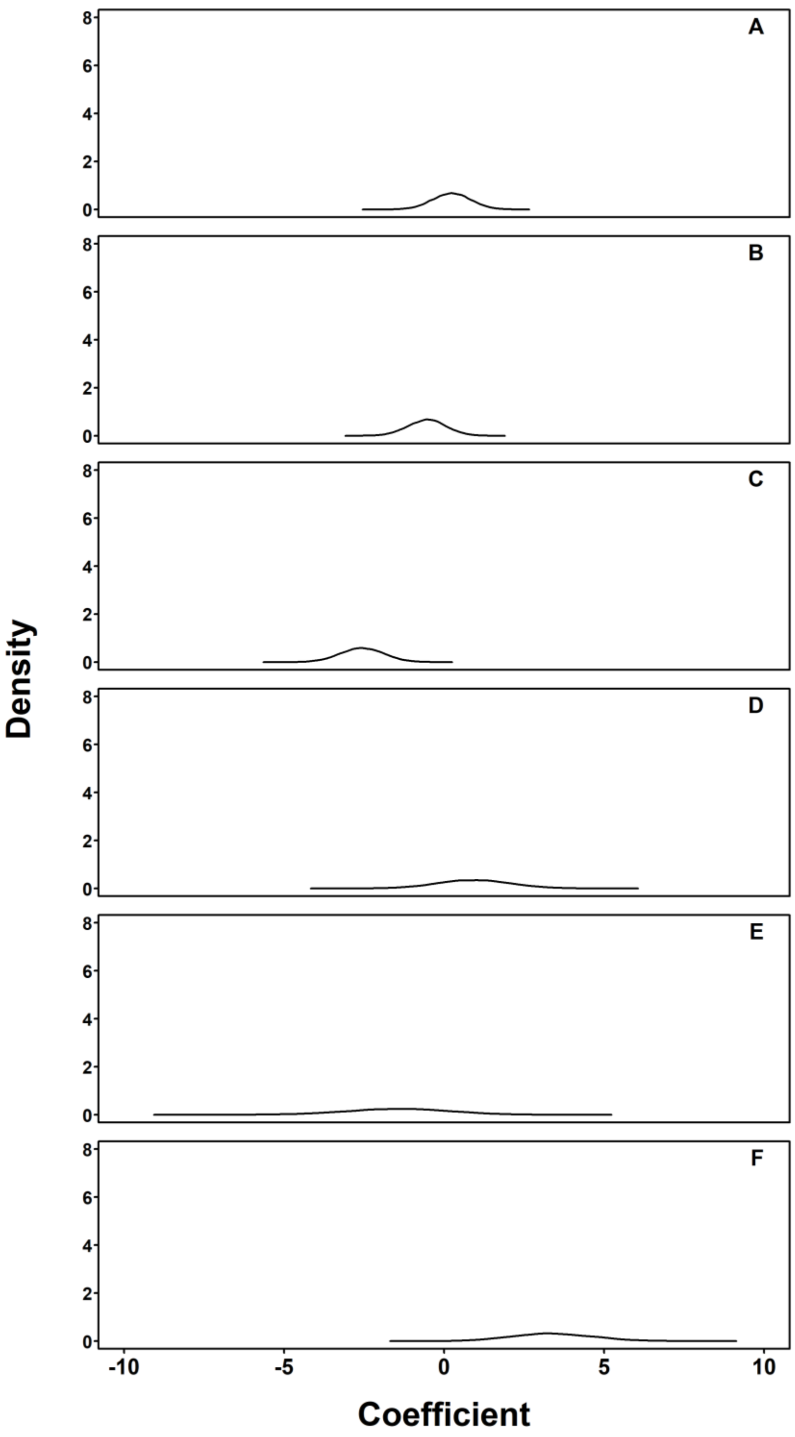

Supplementary Figure 179. Posterior densities of the ANOVA coefficients for the consumers' WTP for giant red shrimp per where consumers buy seafood (from A to F: "Fish market", "Large retail", "Local market", "Not buy", "Online", and "Other"), extracted after 10^4 MCMC draws.

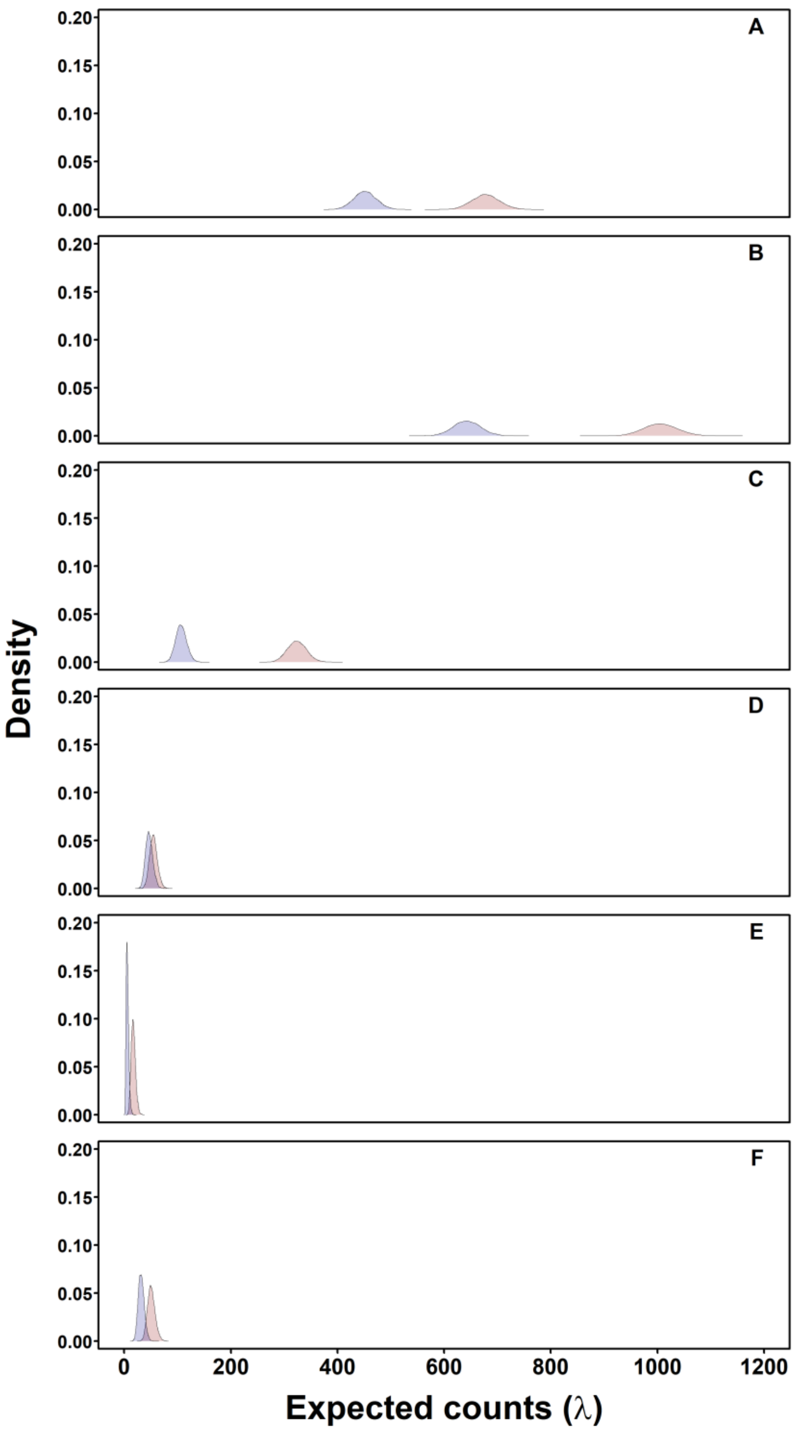

Supplementary Figure 180. Posterior densities of the expected counts (λ) for the consumption of processed albacore tuna (Red= "Yes" and Blue= "No") per where consumers buy seafood (from A to F: "Fish market", "Large retail", "Local market", "Not buy", "Online", and "Other"), extracted after 10^4 MCMC draws.

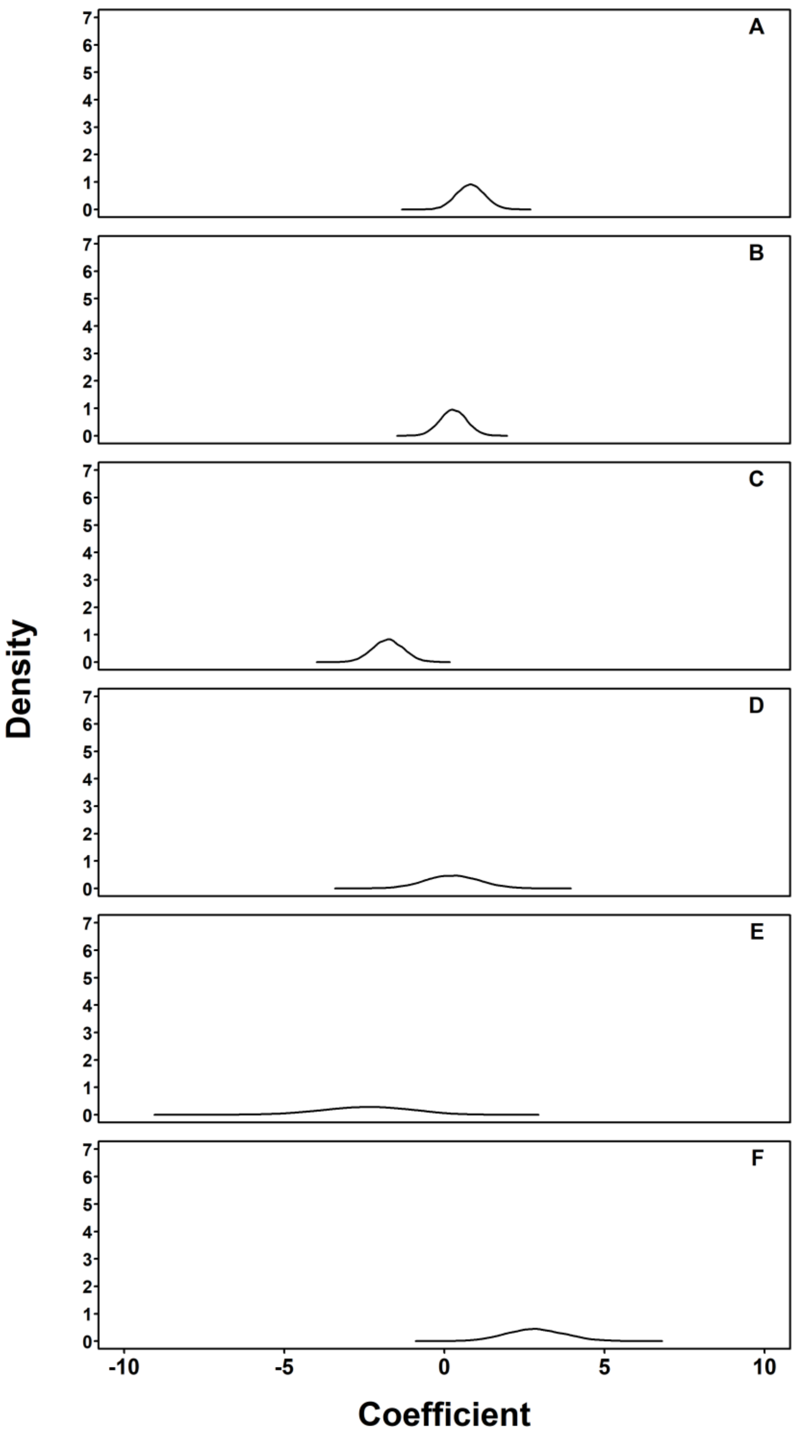

Supplementary Figure 181. Posterior densities of the ANOVA coefficients for the consumers' WTP for processed albacore tuna per where consumers buy seafood (from A to F: "Fish market", "Large retail", "Local market", "Not buy", "Online", and "Other"), extracted after 10^4 MCMC draws.

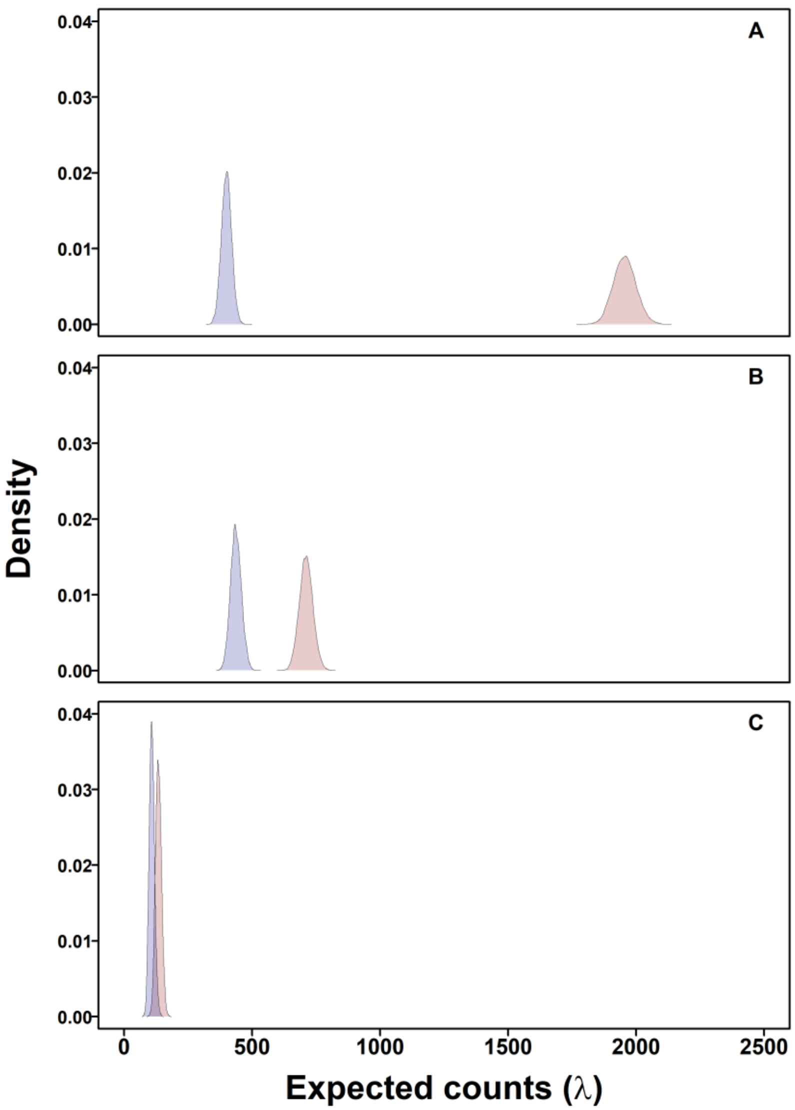

Supplementary Figure 182. Posterior densities of the expected counts (λ) for being informed on seafood origin (Red= "Yes" and Blue= "No") per degree of seafood processing consumers prefer (from A to C: "Fresh" "Frozen", and "Processed"), extracted after 10^4 MCMC draws.

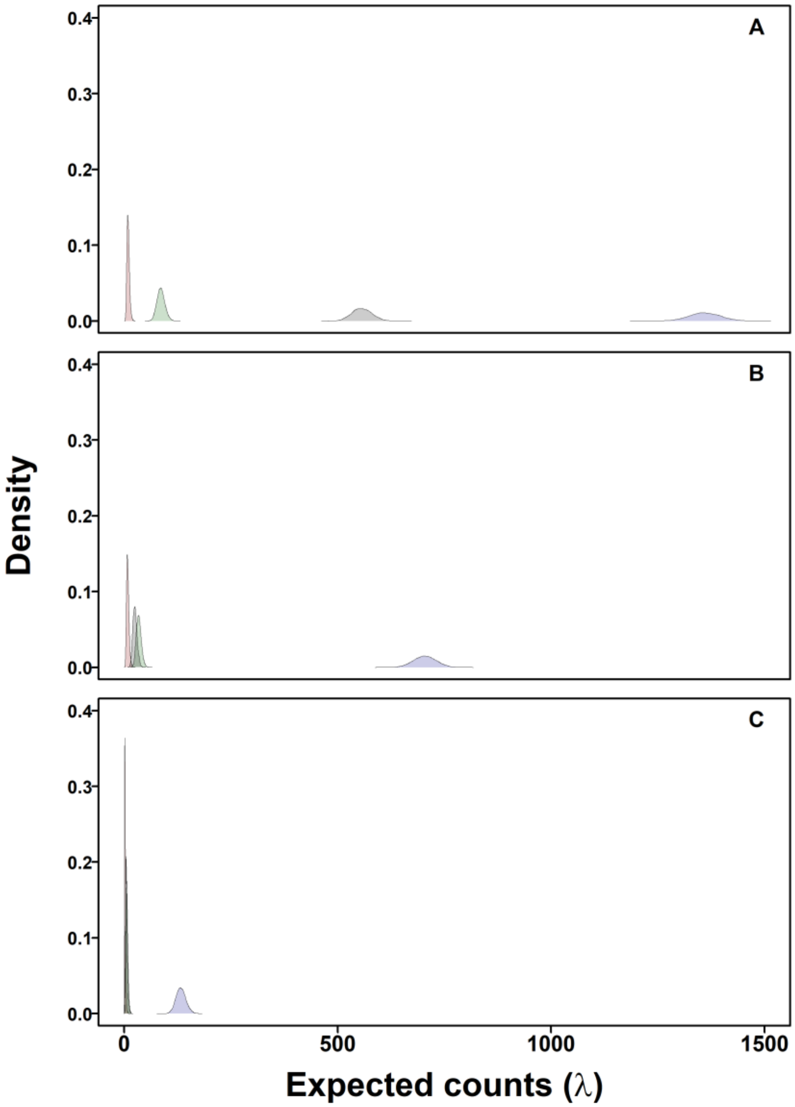

Supplementary Figure 183. Posterior densities of the expected counts (λ) for sources of information on seafood origin (Red= "Ads", Blue= "Label", Green= "Other", and Dark gray= "Retailer") per degree of seafood processing consumers prefer (from A to C: "Fresh" "Frozen", and "Processed"), extracted after 10^4 MCMC draws.

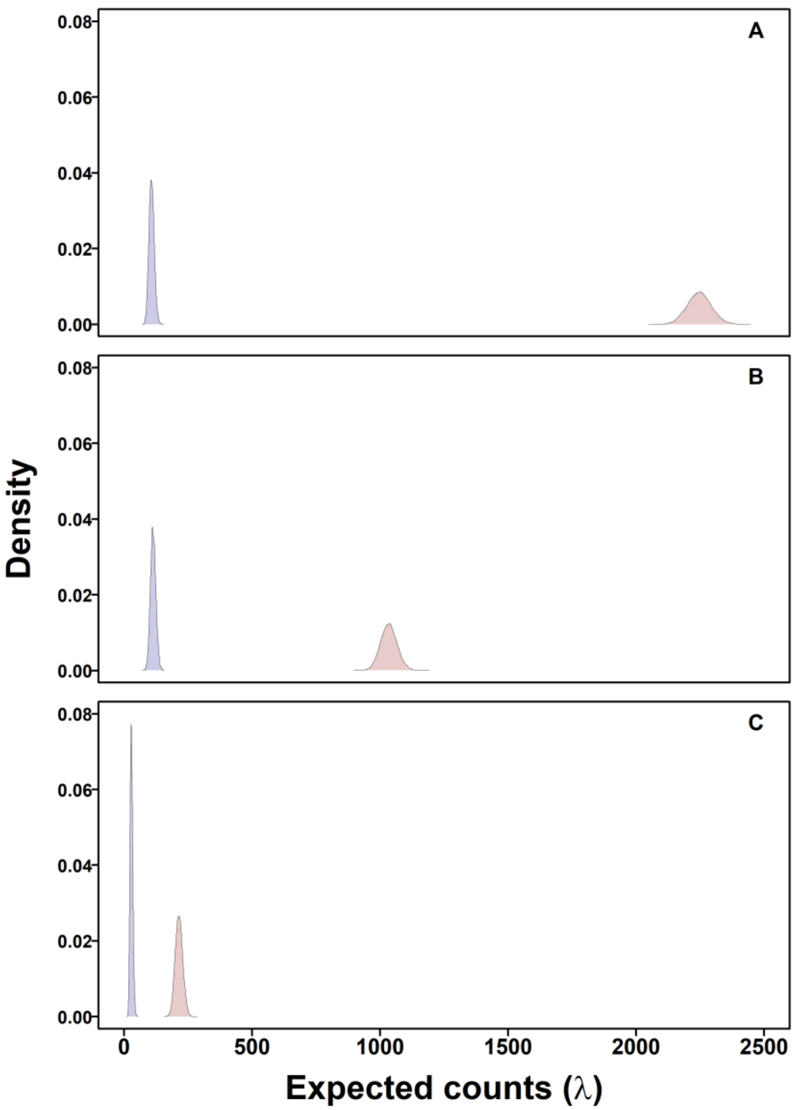

Supplementary Figure 184. Posterior densities of the expected counts (λ) for interest on seafood traceability (Red= "Yes", and Blue= "No") per degree of seafood processing consumers prefer (from A to C: "Fresh" "Frozen", and "Processed"), extracted after 10^4 MCMC draws.

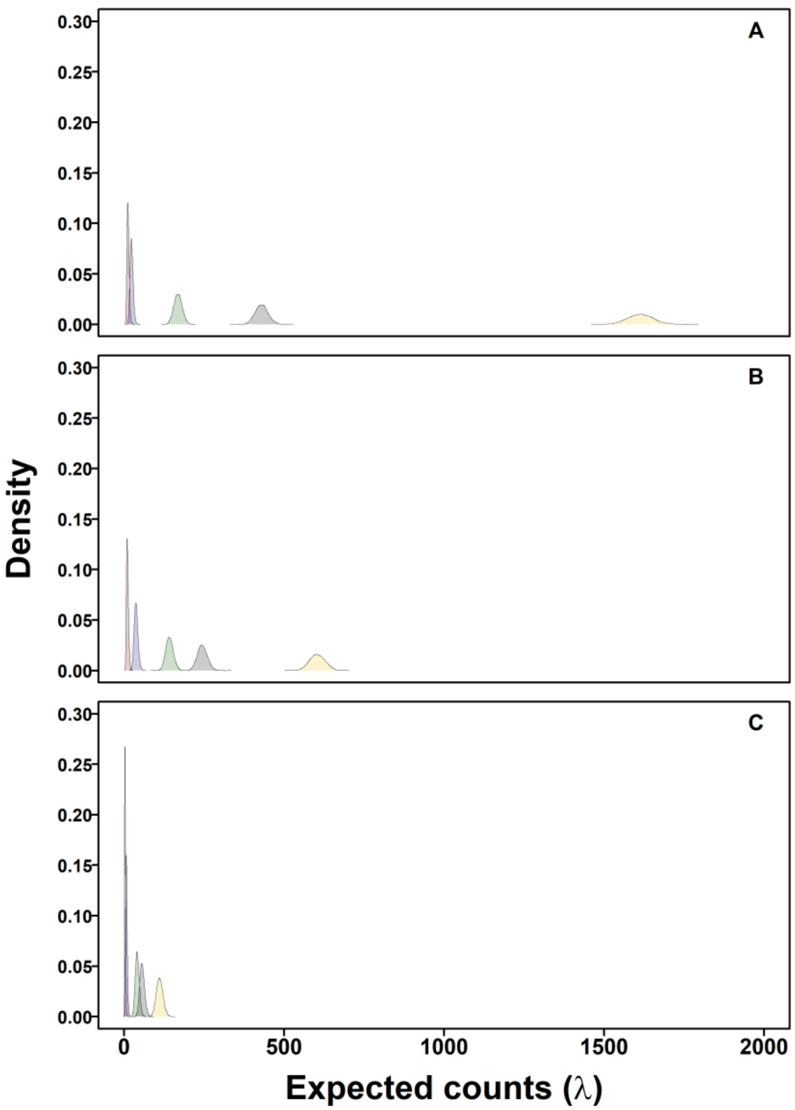

Supplementary Figure 185. Posterior densities of the expected counts (λ) for level of consumers' interest in seafood traceability (Red= 1, Blue= 2, Green= 3, Dark gray= 4, and Yellow= 5) per degree of seafood processing consumers prefer (from A to C: "Fresh" "Frozen", and "Processed"), extracted after 10^4 MCMC draws.

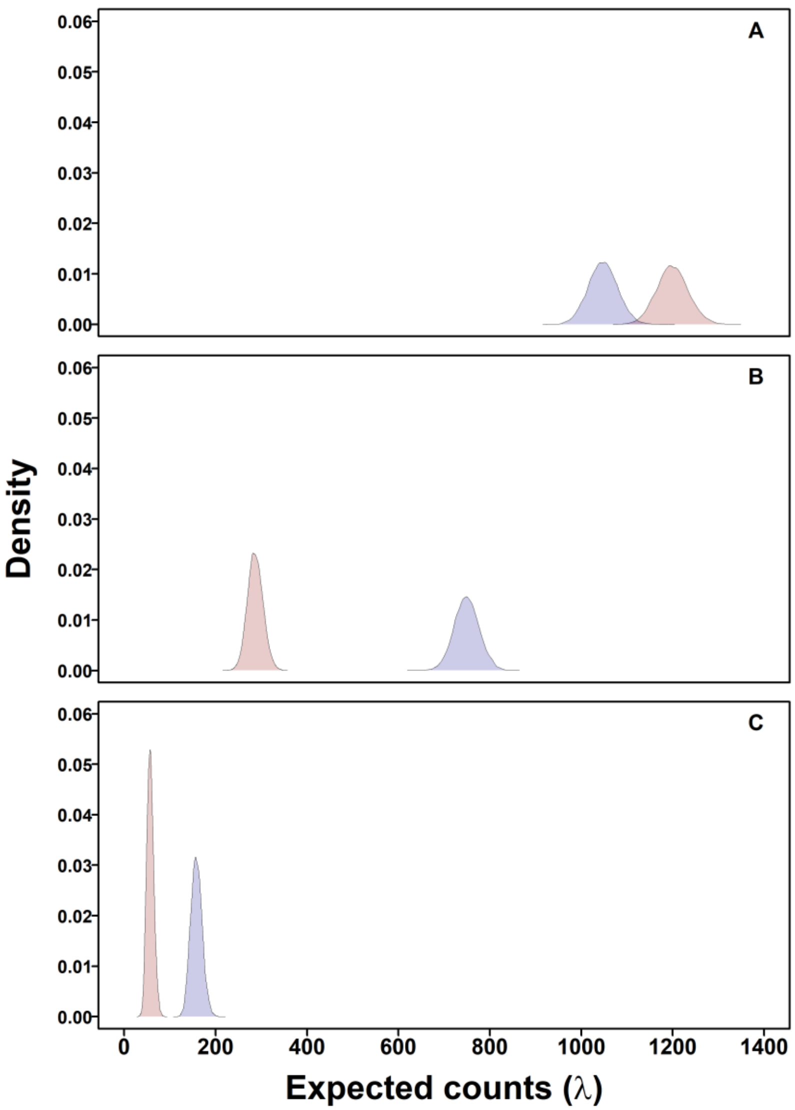

Supplementary Figure 186. Posterior densities of the expected counts (λ) for the consumption of Italian-farmed sea bass (Red= “Yes”, and Blue= “No”) per degree of seafood processing consumers prefer (from A to C: “Fresh” “Frozen”, and “Processed”), extracted after 10^4 MCMC draws.

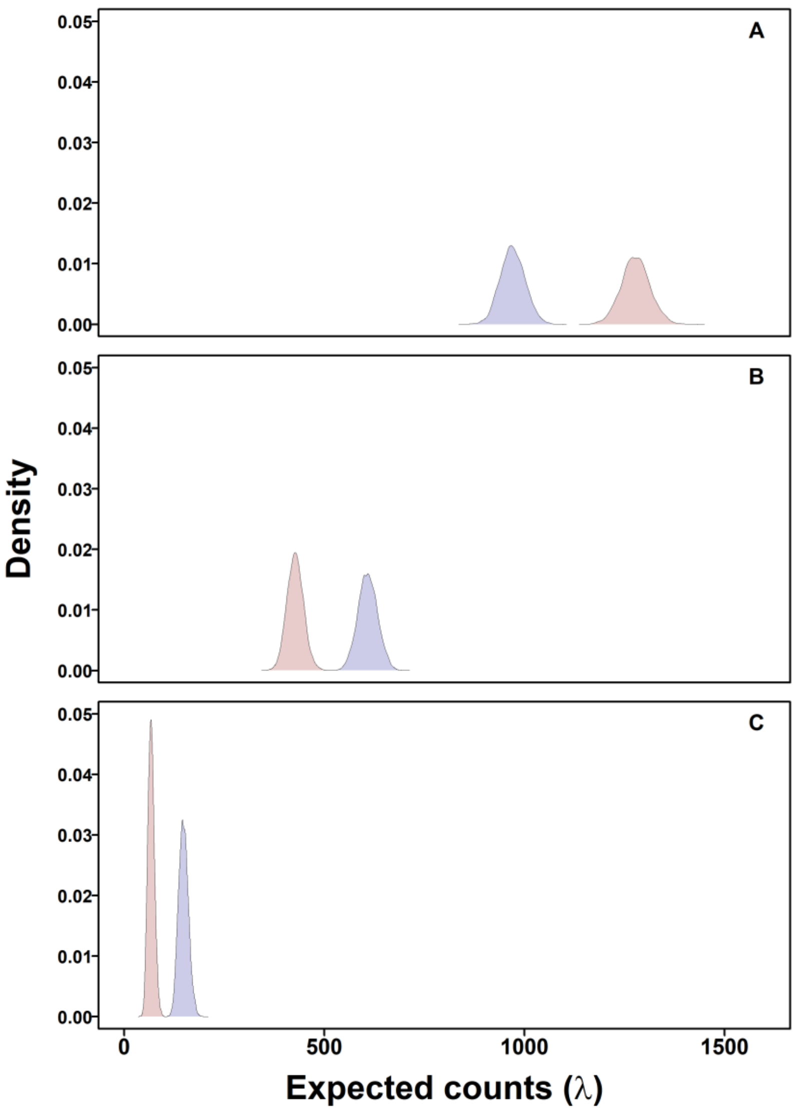

Supplementary Figure 187. Posterior densities of the expected counts (λ) for the consumption of striped venus clams (Red= “Yes”, and Blue= “No”) per degree of seafood processing consumers prefer (from A to C: “Fresh” “Frozen”, and “Processed”), extracted after 10^4 MCMC draws.

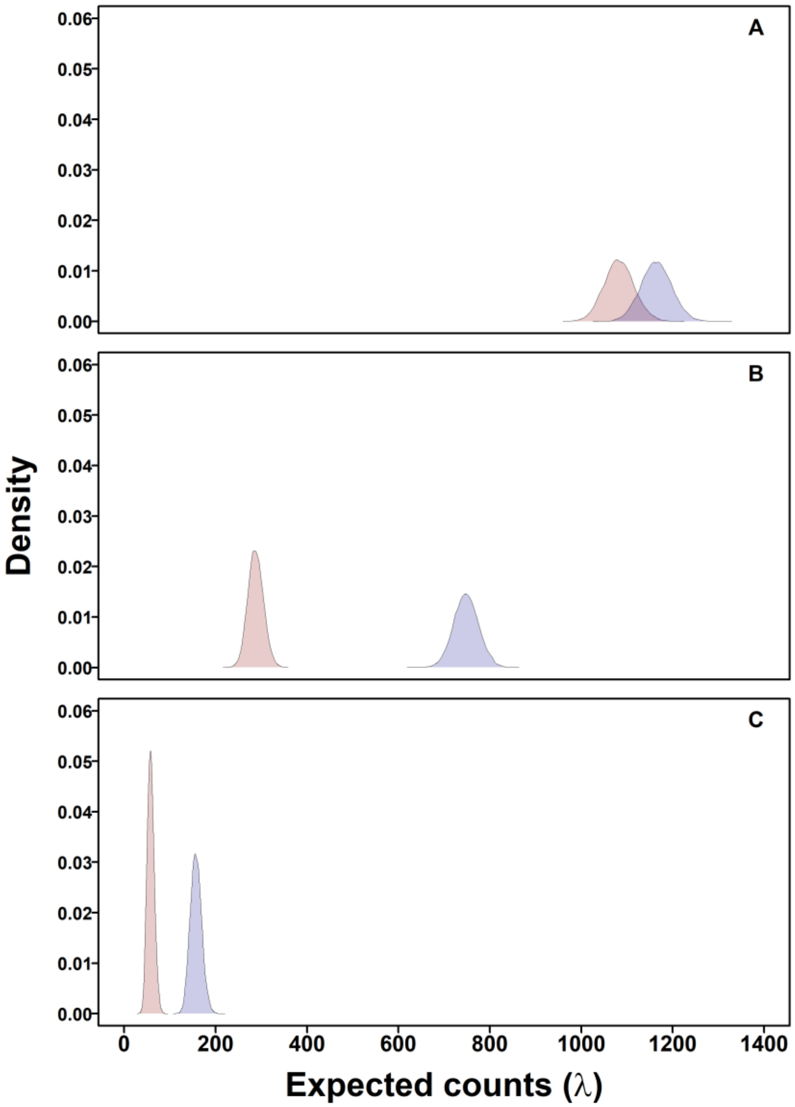

Supplementary Figure 188. Posterior densities of the expected counts (λ) for the consumption of giant red shrimp (Red= “Yes”, and Blue= “No”) per degree of seafood processing consumers prefer (from A to C: “Fresh” “Frozen”, and “Processed”), extracted after 10^4 MCMC draws.

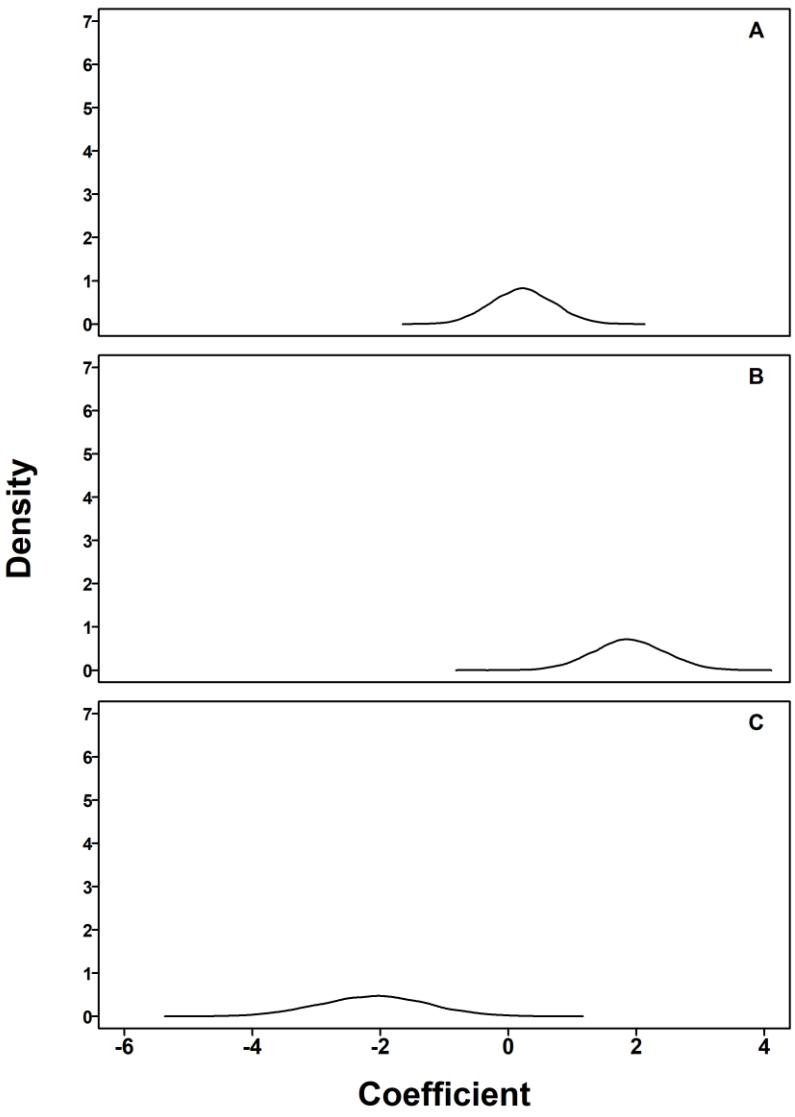

Supplementary Figure 189. Posterior densities of the ANOVA coefficients for the consumers' WTP for giant red shrimp per degree of seafood processing consumers prefer (from A to C: "Fresh" "Frozen", and "Processed"), extracted after 10^4 MCMC draws.

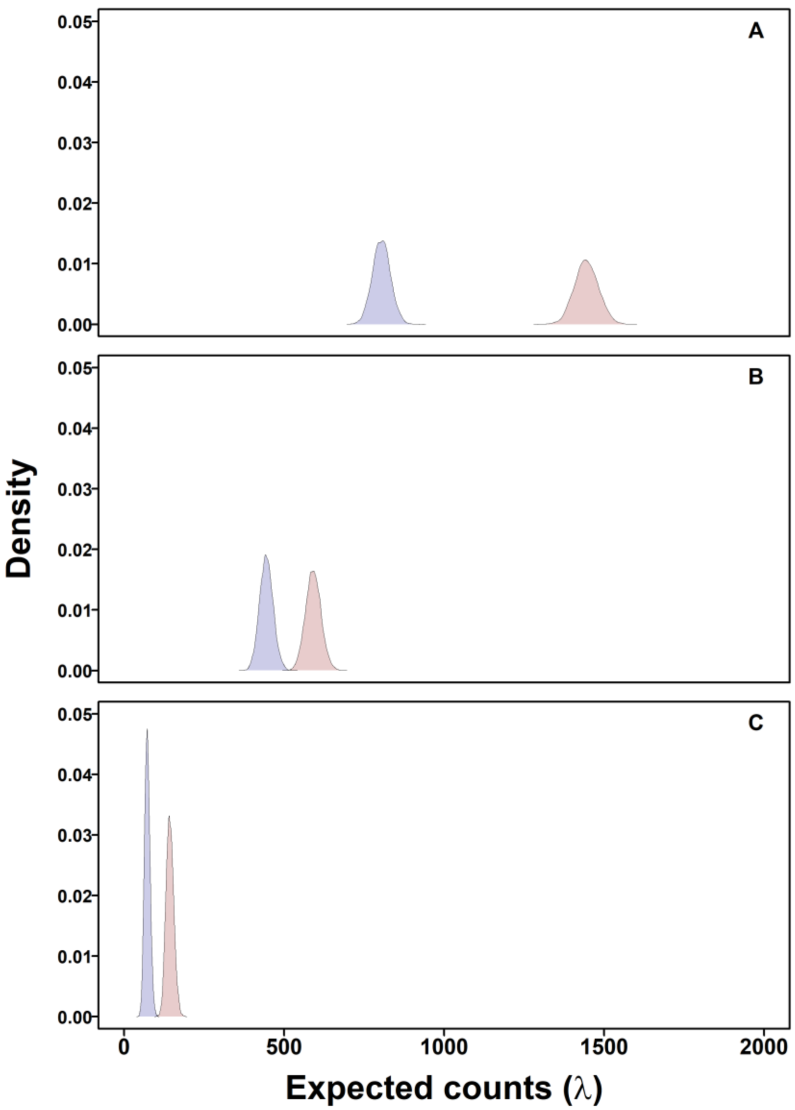

Supplementary Figure 190. Posterior densities of the expected counts (λ) for the consumption of processed albacore tuna (Red= “Yes”, and Blue= “No”) per degree of seafood processing consumers prefer (from A to C: “Fresh” “Frozen”, and “Processed”), extracted after 10^4 MCMC draws.

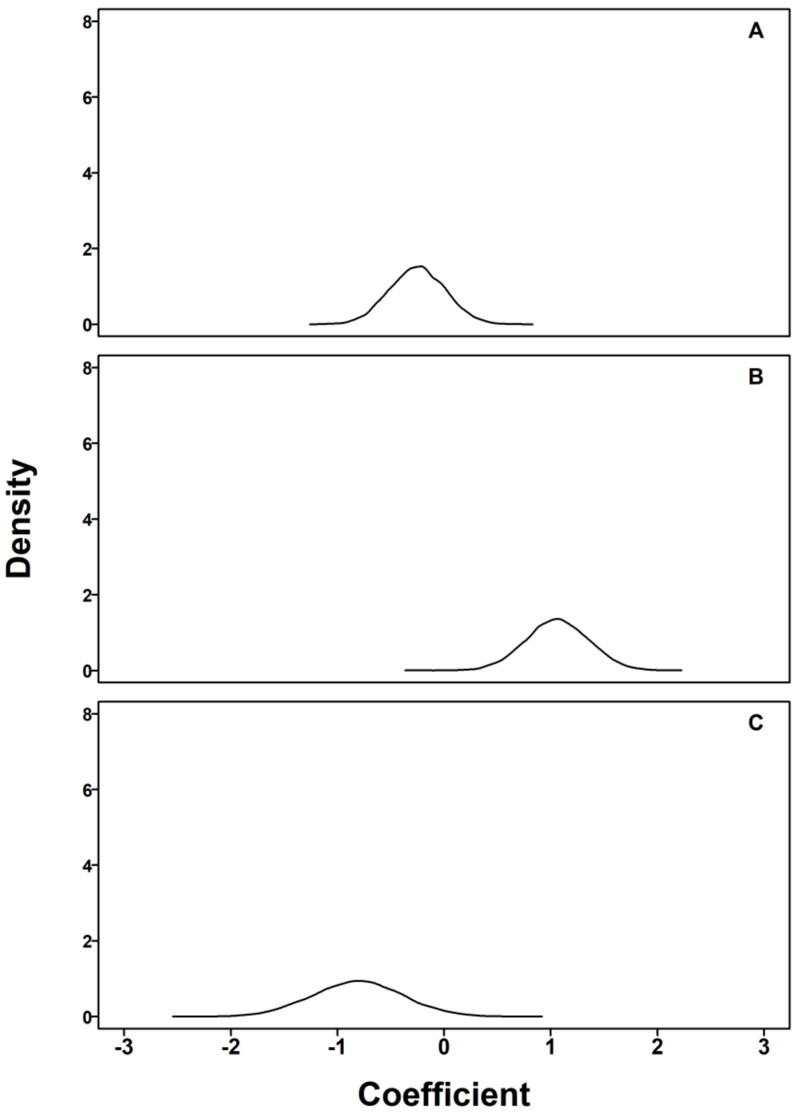

Supplementary Figure 191. Posterior densities of the ANOVA coefficients for the consumers' WTP for processed albacore tuna per degree of seafood processing consumers prefer (from A to C: "Fresh" "Frozen", and "Processed"), extracted after 10^4 MCMC draws.

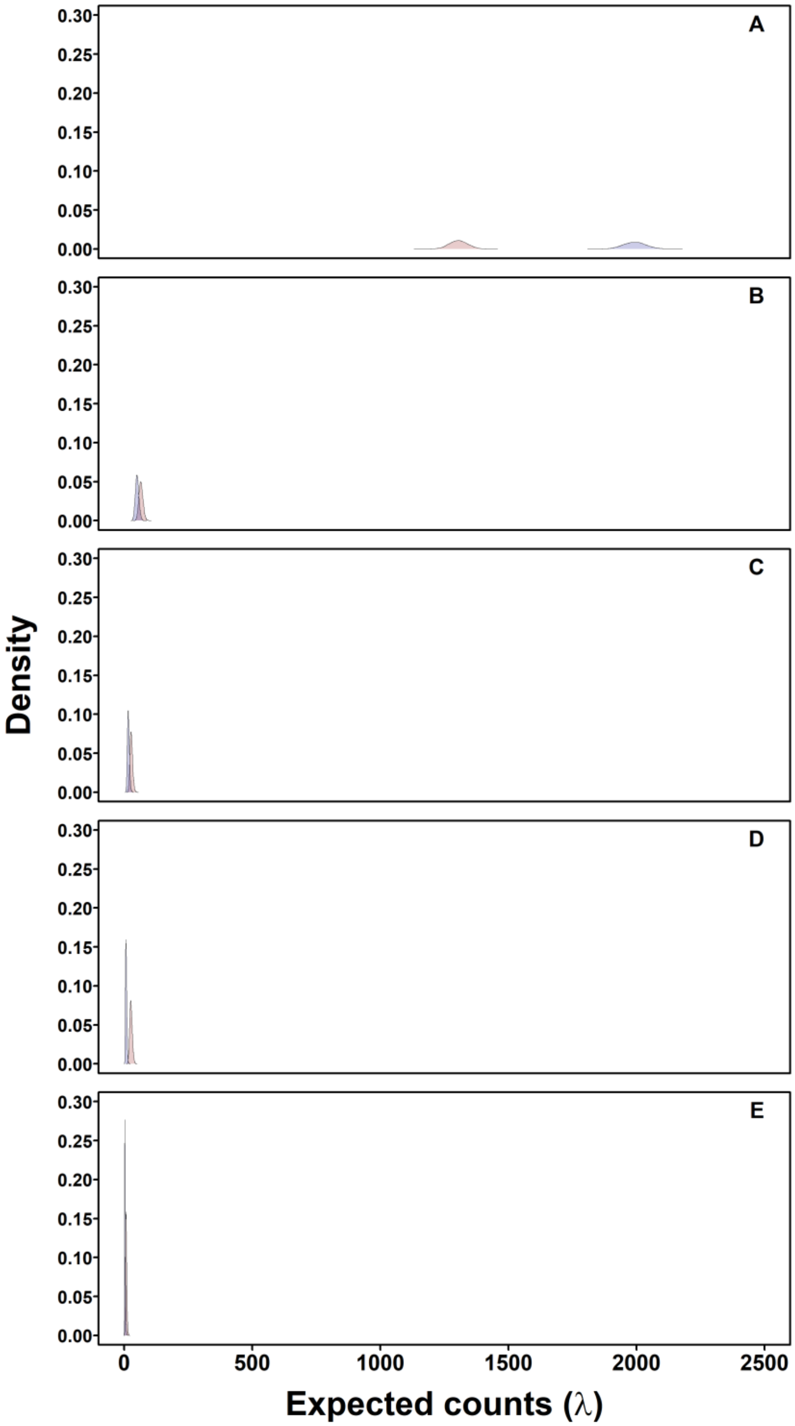

Supplementary Figure 192. Posterior densities of the expected counts (λ) for the consumption of giant red shrimp (Red= “Yes”, and Blue= “No”) per frequency of online seafood purchasing (from A to E: “Never”, “Rarely”, “Sometimes”, “Often”, and “Always”), extracted after 10^4 MCMC draws.

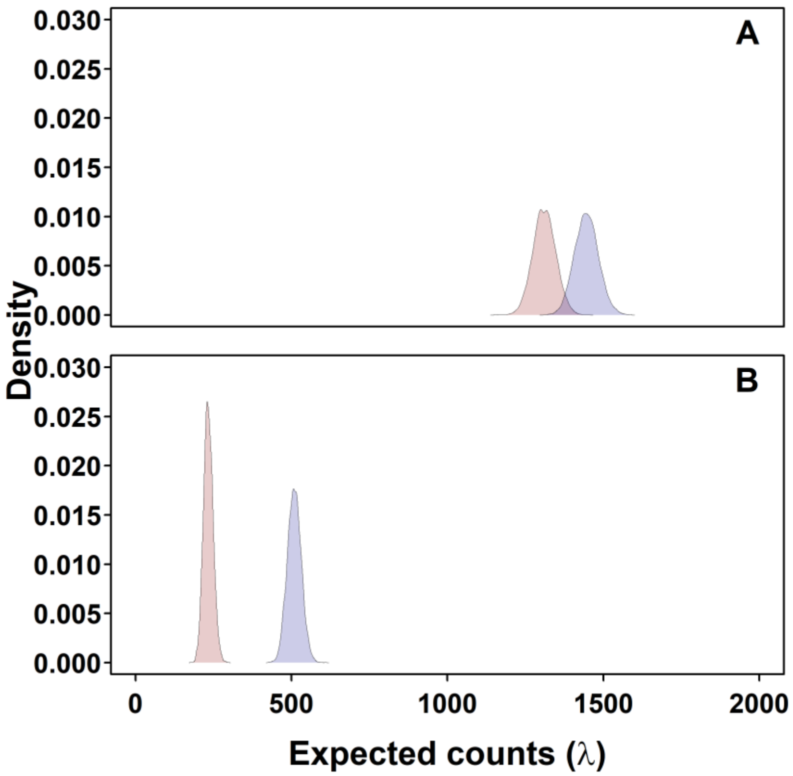

Supplementary Figure 193. Posterior densities of the expected counts (λ) for the consumption of Italian-farmed sea bass (Red= “Yes”, and Blue= “No”) per being informed on seafood origin (from A to B: “Yes”, and “No”), extracted after 10^4 MCMC draws.

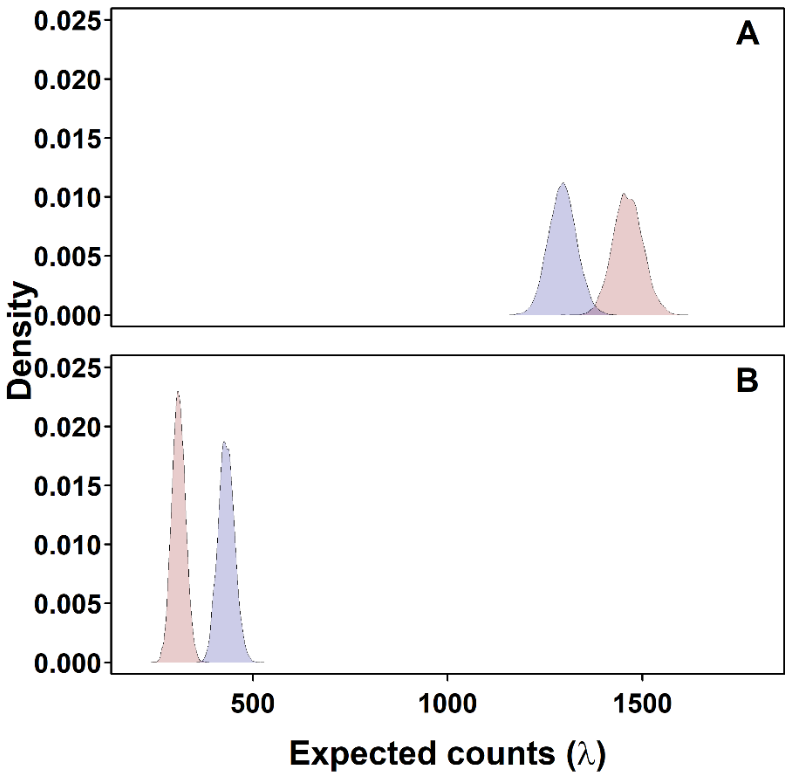

Supplementary Figure 194. Posterior densities of the expected counts (λ) for the consumption of striped venus clams (Red= "Yes", and Blue= "No") per being informed on seafood origin (from A to B: "Yes", and "No"), extracted after 10^4 MCMC draws.

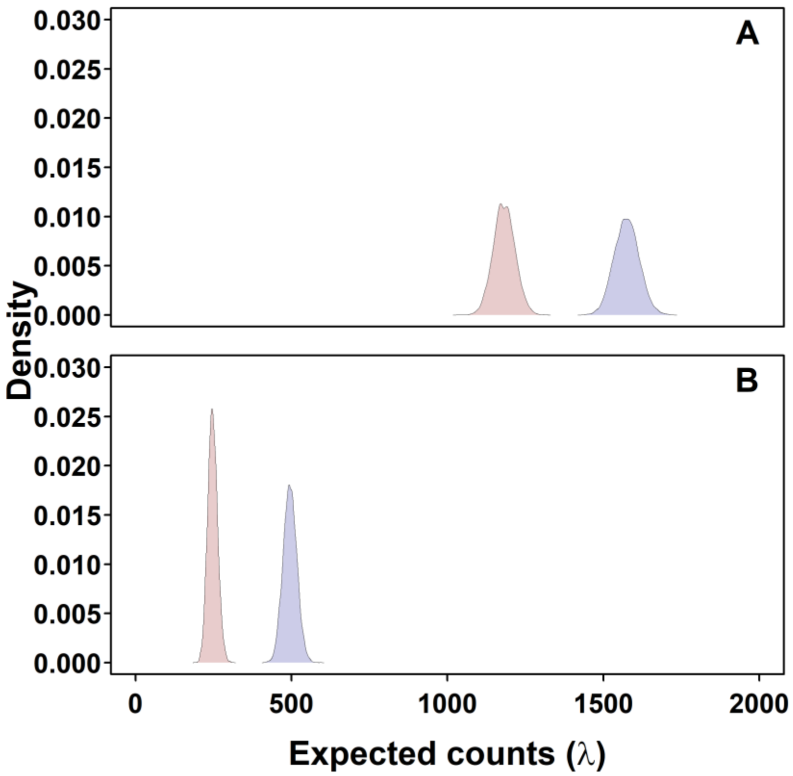

Supplementary Figure 195. Posterior densities of the expected counts (λ) for the consumption of giant red shrimp (Red= "Yes", and Blue= "No") per being informed on seafood origin (from A to B: "Yes", and "No"), extracted after 10^4 MCMC draws.

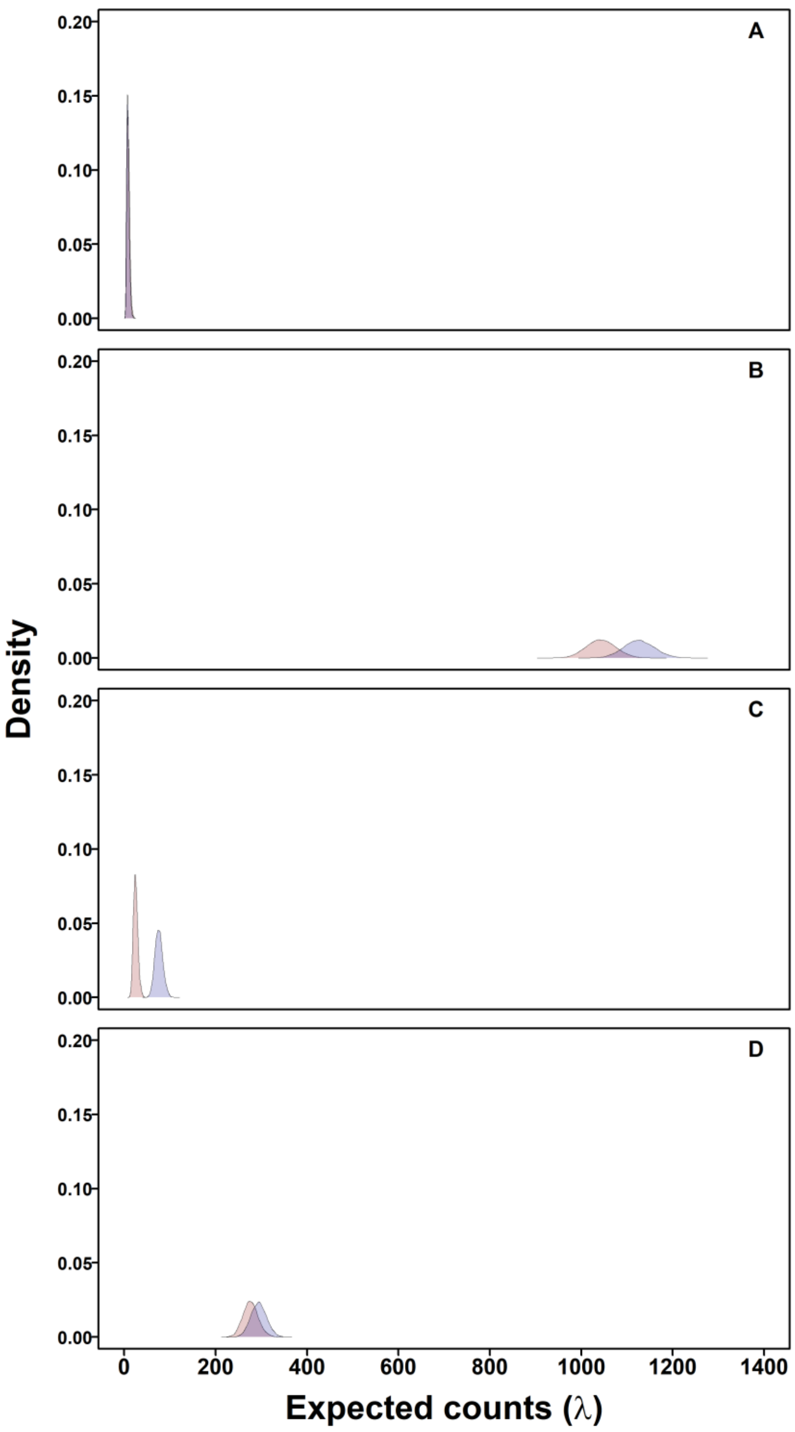

Supplementary Figure 196. Posterior densities of the expected counts (λ) for the consumption of Italian-farmed sea bass (Red= "Yes", and Blue= "No") per source of information on seafood origin (from A to D: "Ads", "Label", "Other", and "Retailer"), extracted after 10^4 MCMC draws.

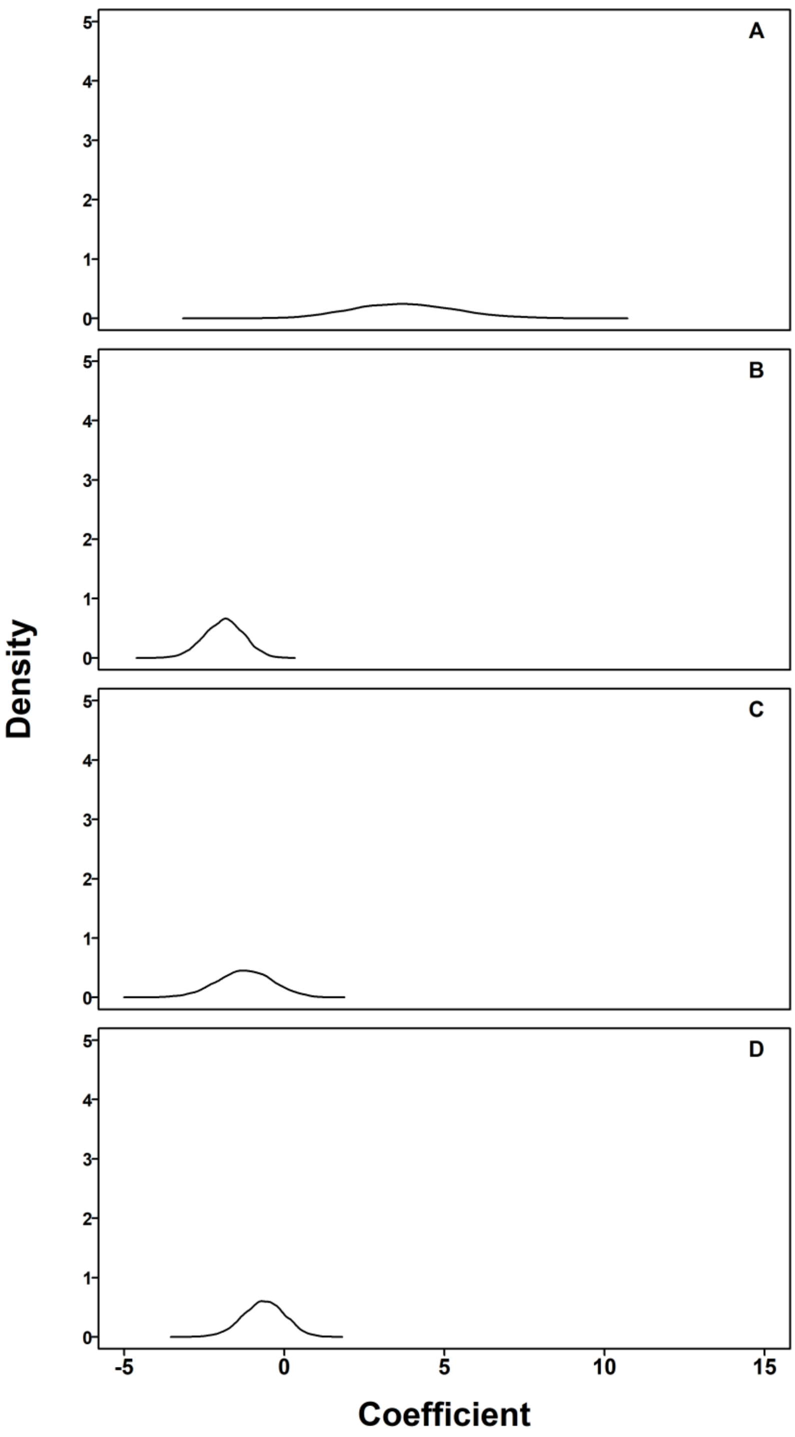

Supplementary Figure 197. Posterior densities of the ANOVA coefficients for the consumers' WTP for processed albacore tuna per source of information on seafood origin (from A to D: "Ads", "Label", "Other", and "Retailer"), extracted after 10^4 MCMC draws.

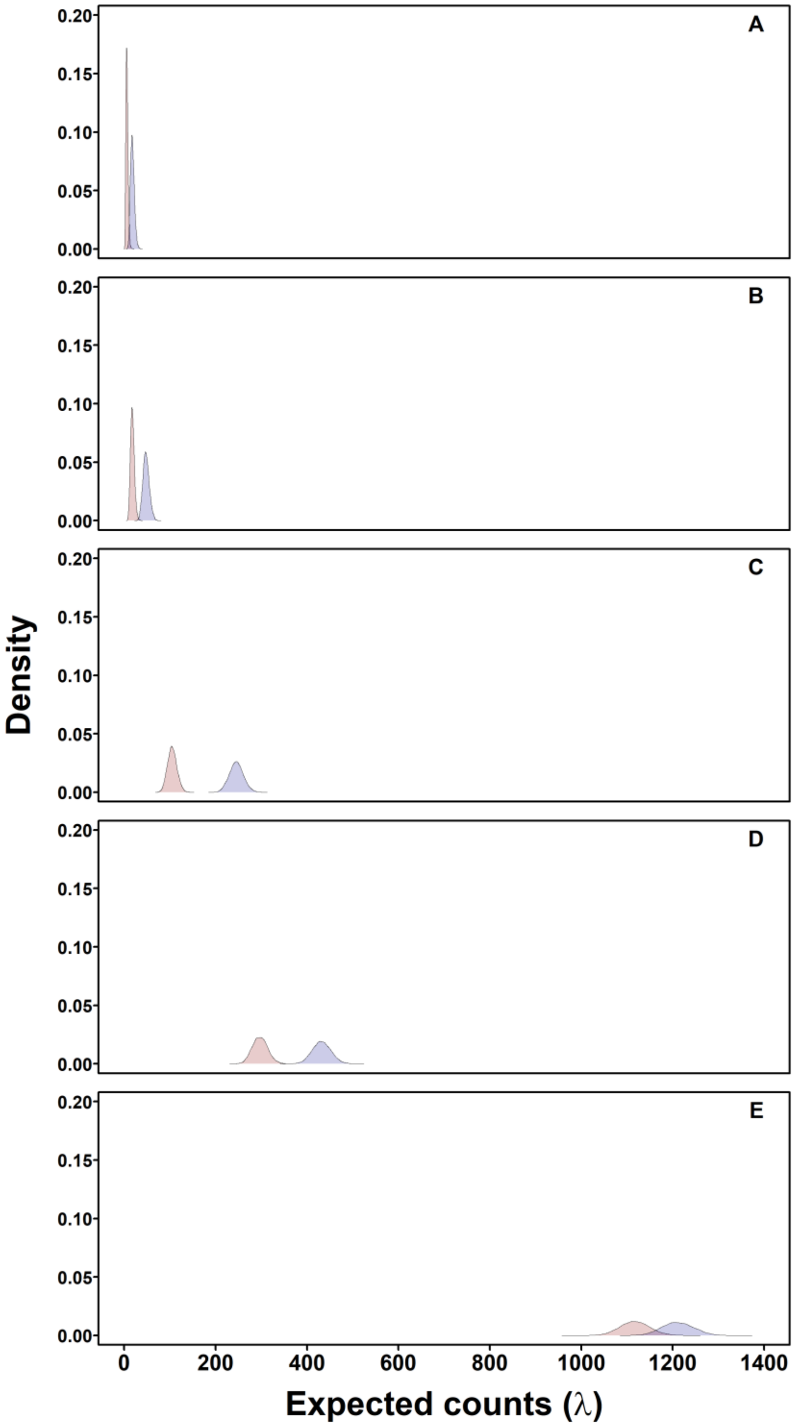

Supplementary Figure 198. Posterior densities of the expected counts (λ) for the consumption of Italian-farmed sea bass (Red= "Yes", and Blue= "No") per level of consumers' interest in seafood traceability (from A to E: 1, 2, 3, 4, and 5), extracted after 10^4 MCMC draws.
